# Evolution, Systematics and Classification of Commelinales (Commelinids, Monocots) Based on a Giant Morphological Taxon-Character Matrix

**DOI:** 10.3390/plants15111738

**Published:** 2026-06-03

**Authors:** Marco O. O. Pellegrini

**Affiliations:** 1 C.E. Moss Herbarium, School of Animal, Plant & Environmental Sciences, University of the Witwatersrand, Johannesburg 2050, Gauteng Province, South Africa; marco.pellegrini@wits.ac.za or m.pellegrini@kew.org; 2 Royal Botanic Gardens Kew, London TW9 3AB, Surrey, UK

**Keywords:** Commelinaceae, Haemodoraceae, Hanguanaceae, Philydraceae, Pontederiaceae, taxonomy, nomenclature

## Abstract

Despite being strongly recovered as monophyletic by molecular studies, Commelinales completely lacks any morphological support or circumscription. It is also the Monocot order that suffered the most striking changes across different classification systems, with its type family, Commelinaceae, being the only consistent member since its proposition. The order currently consists of Commelinaceae, Haemodoraceae, Hanguanaceae, Philydraceae and Pontederiaceae, presenting a Pantropical distribution and great ecological and morphological diversity. Based on extensive field, cultivation, ecological, herbarium, botanical illustration and literature research, I present the first morphological phylogeny for Commelinales, based on an extensive 600-character matrix, sampling almost a third of the species in the order. All five families are recovered as monophyletic, with 49 of the 59 currently recognised genera also recovered as monophyletic. The M P and BA topologies are greatly congruent with the available molecular hypotheses for Commelinales, highlighting the importance of morphology in understanding the systematics of plant groups. Almost all genera are morphologically supported by at least one exclusive synapomorphy. Thus, based on a combination of morphological and molecular data, *Aneilema*, *Callisia*, *Coleotrype*, *Elasis*, *Thyrsanthemum*, *Tricarpelema* and *Tripogandra* (Commelinaceae) are recircumscribed to represent monophyletic genera. Five new genera of Commelinaceae are described, in addition to the reestablishment of *Aploleia*, *Cuthbertia*, *Gibasoides* and *Hadrodemas* (Commelinaceae), and *Orthotylax* (Philydraceae). The circumscription of *Anigozanthos* (Haemodoraceae) is broadened to include *Macropidia*, *Conostylis* (Haemodoraceae) is broadened to include *Blancoa*, and *Wachendorfia* (Haemodoraceae) is broadened to include *Barberetta*. Finally, I propose an updated classification for Commelinales, recognising two suborders, one superfamily, five families, four subfamilies, 10 tribes, 13 subtribes (four of them newly described here), and 64 genera (five of them newly described here).

## 1. Introduction

Commelinales is a small and well-supported Monocot order, currently understood as belonging to the commelinid Monocots. This group also includes the Commelinales’ sister group, the order Zingiberales, together with Poales, Arecales, and Dasypogonales [1,2]. The order is composed of Commelinaceae, Haemodoraceae, Hanguanaceae, Philydraceae, and Pontederiaceae [1,2,3,4,5,6,7,8,9,10,11,12,13], and currently includes 59 genera and ca. 1000 species [14]. Despite its relatively reduced number of genera and species, Commelinales is geographically, ecologically, and morphologically very diverse [12,15]. The order is Pantropical in distribution, reaching temperate areas in some parts of the world [12,14,15,16,17,18], especially due to the wide distribution of Commelinaceae, Haemodoraceae, and Pontederiaceae. Regarding the individual families, Australasia represents the diversity centres of Haemodoraceae [19,20], Hanguanaceae [21], and Philydraceae [22,23]. Alternatively, Pontederiaceae has the Neotropics as its diversity centre, especially Brazil [24,25]. Finally, Commelinaceae possesses two Neotropical (i.e., Mexico and Brazil) and two Paleotropical (i.e., Africa and Asia) diversity centres, being the most widespread family [26,27,28,29,30,31,32,33,34].

**Figure 1:**
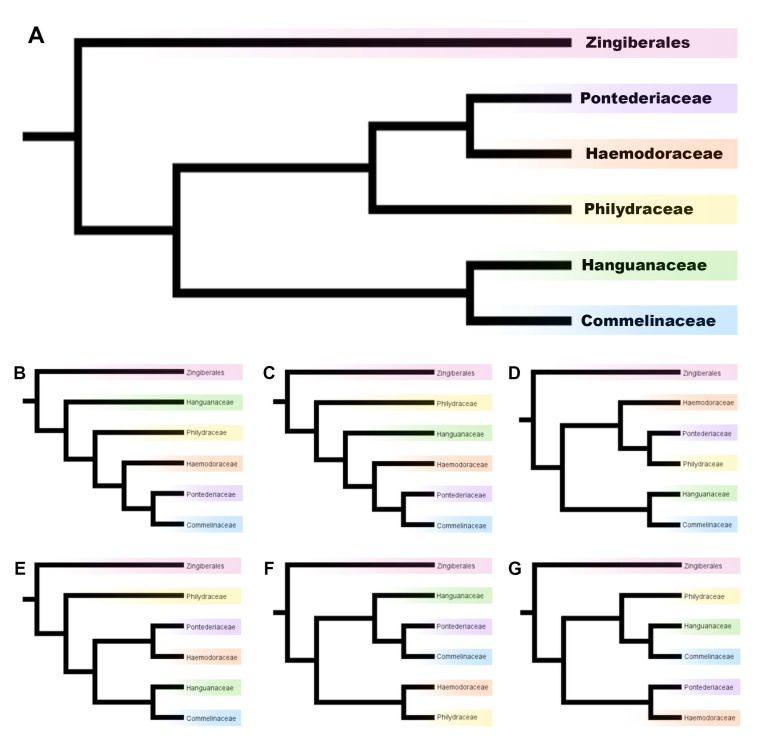
Comparison between the current and previous hypotheses for the phylogenetic relationships between the families of Commelinales. (**A**), Hanguanaceae as sister to Commelinaceae, which is in turn recovered as sister to the unifacial clade, with Haemodoraceae and Pontederiaceae as sister to Philydraceae, recovered based on morphology in the present study and using plastid markers [6,10,12,39,41,44] and using plastomes [2]. (**B**), Recovered using plastid markers [40]. (**C**), Recovered using plastid markers [55]. (**D**), Recovered using plastid markers [3,9]. (**E**), Recovered using plastid markers [1,4,45]. (**F**), Recovered using plastid markers [11]. (**G**), Recovered using nuclear genes [45], using plastomes [42,43], and using Angiosperms353 [44].

Commelinales is probably the least studied Monocot order from an evolutionary and taxonomic point of view, having suffered the most striking changes in its circumscription between different classification systems [3,12,35,36]. The only family consistently associated with Commelinales is its type family, Commelinaceae. Most families historically placed in emblematic previous circumscriptions of Commelinales (i.e., Eriocaulaceae, Flagellariaceae, Mayacaceae, Rapateaceae, Restionaceae, and Xyridaceae) are currently placed in Poales [3,13,35], while Dasypogonaceae is either placed in Arecales [1,13] or in Dasypogonales [2,3]. Alternatively, the families currently placed in Commelinales have rarely been considered to be closely related to Commelinaceae [3,35]. Haemodoraceae, Philydraceae, and Pontederiaceae have generally been considered to be more or less closely related to each other, despite being placed in different orders depending on the classification system. Hanguanaceae has consistently been considered a family of uncertain affinity amongst the Monocots [37,38]. Currently, despite the strong molecular support for the monophyly of Commelinales, the relationship between its families remains highly contentious [1,2,3,6,9,10,11,12,39,40,41,42,43,44,45], with very distinct topologies recovered depending on the dataset or even the type of analysis for the same dataset (Figure 1). Additionally, as currently accepted, Commelinales is circumscribed based exclusively on molecular characters, lacking any kind of morphological support [12,14,16,17,18,35]. Despite some systematic studies having recovered morphological synapomorphies for the individual families in Commelinales (Haemodoraceae: [19,20,46,47,48,49]; Philydraceae: [19,50]; Pontederiaceae: [19,25,51,52]), very few studies have proposed or recovered synapomorphies for suprafamilial groupings in the order [3,12,25,53,54]. In contrast, a single study has recovered a single putative morphological synapomorphy for the order [3]. The order as a whole has been superficially characterised by the absence of mycorrhizae, vessel elements with scalariform perforation plates, cuticle waxes not as aggregated rodlets, inflorescence thyrsoid, secondary branches of scorpioid cymes, flowers generally monosymmetric, generally few fertile stamens, seed coat testal and tegmic, endosperm abundant and helobial, and the presence of collar rhizoids in seedlings [3,12,14,16,17,18,35].

The current study aims to: (1) investigate the phylogenetic relationships within Commelinales (in order to try to provide a more robust hypothesis for its interfamilial relationships); (2) retrieve unambiguous morphological synapomorphies for the order, higher relationships, its families and infrafamilial taxa; (3) test the monophyly of all currently accepted supraspecific taxa and their circumscription; and (4) explore the congruence of morphological and molecular characters and their phylogenetic potential for Commelinales. In order to do so, I present a morphological phylogeny for Commelinales based on a giant character-taxon matrix (the largest morphological matrix ever compiled for plants), including macro- and micromorphological, ecological, palynological, anatomical, cytological, and phytochemical characters.

## 2. Results

**Figure 2:**
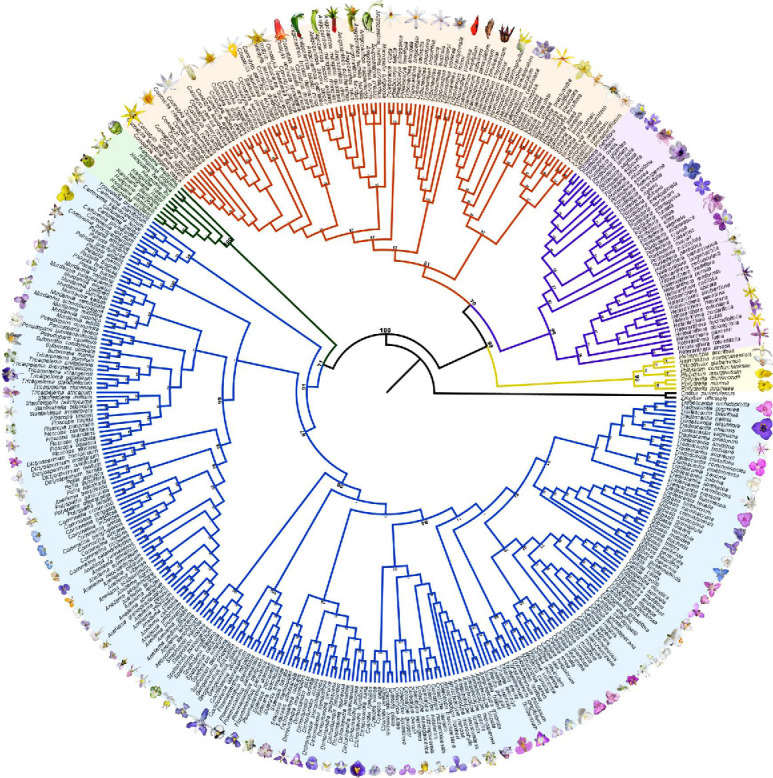
Maximum Parsimony consensus circular tree for Commelinales with flowers illustrating the diagnostic floral morphology for each genus in the order. Bootstrap values (BS) are presented above each node. Families coloured to facilitate visualisation, with Philydraceae in yellow, Pontederiaceae in purple, Haemodoraceae in orange, Hanguanaceae in green, and Commelinaceae in blue.

**Figure 3:**
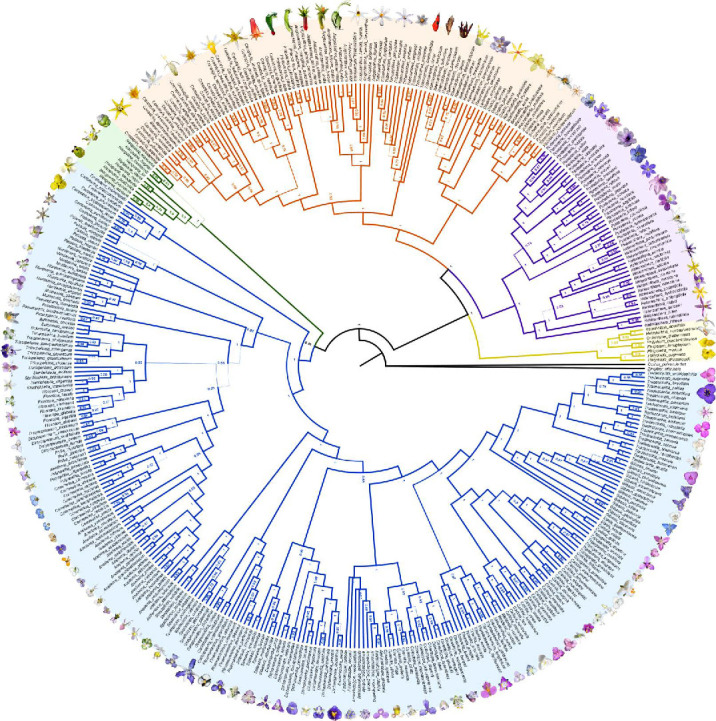
Bayesian Analysis consensus circular tree for Commelinales with diagnostic floral morphology arranged around it. Posterior Probability values (PP) are presented above each node. Families coloured to facilitate visualisation, with Philydraceae in yellow, Pontederiaceae in purple, Haemodoraceae in orange, Hanguanaceae in green, and Commelinaceae in blue.

The MP retrieved three equally parsimonious trees with 5323 steps, CI of 0.2241 (0.2216 when excluding uninformative characters), HI of 0.7759 (0.7784 when excluding uninformative characters), RI of 0.9013, and RC of 0.2020 (Figure 2). Little incongruence was observed between the MP and the BA topologies. The BA retrieved a consensus tree with all ranks ranging from family to subgenus as well-supported (Figure 3). Out of the 600 coded characters, 583 were parsimony informative, with 16 characters being variable but uninformative, and a single character being constant.

Due to the huge size of the present character matrix, listing, describing and discussing all of them would make this already lengthy article unnecessarily long. Thus, I have chosen to focus mainly on the exclusive synapomorphic characters and to just address selected homoplastic ones when these are systematically and taxonomically relevant to Commelinales. In an attempt to make this article a bit more concise, I also summarise synapomorphic (both exclusive and homoplastic) characters recovered in the present analysis in Table 1, Table 2, Table 3, Table 4 and Table 5. The tables are organised by taxonomic ranks (or equivalent clades), and taxa are presented in phylogenetic order and secondarily arranged alphabetically.

Because the families of Commelinales differ drastically in size, tables covering ranks below family and above genus (i.e., Table 2 and Table 4) do not include the monogeneric Hanguanaceae and the bigeneric Pontederiaceae, since these ranks do not exist. In the case of Philydraceae, Haemodoraceae and Commelinaceae, whenever clades have been formally named, that is the name that is used in the table. However, not all clades in a phylogeny are or have to be named (especially since there are not enough taxonomic ranks for such), so they are given informal names to facilitate the description and discussion of the results. Finally, Table 3 summarises the generic synapomorphies for Philydraceae, Pontederiaceae, Haemodoraceae and Hanguanaceae, while Table 5 does the same for Commelinaceae.

### 2.1. Suprafamilial and Familial Relationships

**Figure 4:**
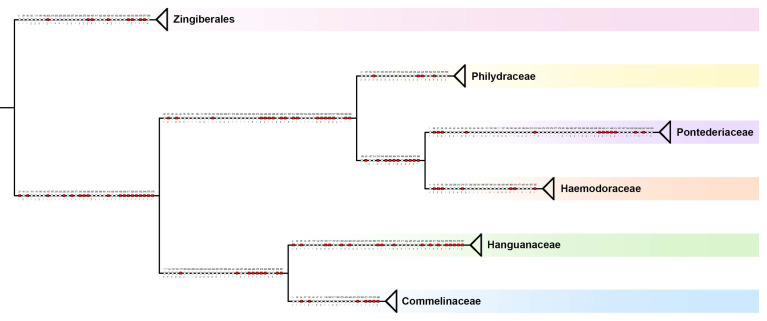
Simplified Maximum Parsimony consensus tree for Commelinales, showing the character optimisation mapped using WinClada (version 1.0000) over each branch of the cladogram. Homoplastic synapomorphies are shown as white circles, while exclusive synapomorphies are shown as red squares. The numbers above represent characters, while the ones below represent character states (as presented in Table S2).

In both the MP and BA topologies, Commelinales is recovered, organised in two well-supported clades (Figure 2 and Figure 3): (1) the bifacial clade, consisting of [Commelinaceae + Hanguanaceae] (BS = 71; PP = 0.99) and (2) the unifacial clade, consisting of [Philydraceae [Haemodoraceae + Pontederiaceae]] (BS = 99; PP = 1). The order as a whole is supported by 34 characters, of which 18 are exclusive (Figure 4; Table 1). The bifacial clade (i.e., [Commelinaceae + Hanguanaceae]) is supported by 30 characters, of which nine are exclusive (Figure 4; Table 1). The unifacial clade (i.e., [Philydraceae [Haemodoraceae + Pontederiaceae]]) is supported by 47 characters, of which 18 are exclusive (Figure 4; Table 1). Finally, the [Haemodoraceae + Pontederiaceae] clade has medium statistical support (BS = 71; PP = 1), morphologically supported by 15 characters, of which 10 are exclusive (Figure 4; Table 1).

All five families of Commelinales are recovered as monophyletic, with medium to high statistical support (Figure 2 and Figure 3). Philydraceae is statistically strongly supported as monophyletic (BS = 99; PP = 1) by 22 synapomorphic characters, four of which are exclusive (Figure 4; Table 1). Haemodoraceae recovered as monophyletic with high statistical support (BS = 81; PP = 1), morphologically supported by 27 synapomorphic characters, with seven of them being exclusive (Figure 4; Table 1). Pontederiaceae is statistically strongly supported as monophyletic (BS = 99; PP = 1), being morphologically supported by 56 synapomorphic characters, 12 of which are exclusive (Figure 4; Table 1). Hanguanaceae is recovered as monophyletic (BS = 100; PP = 1) and supported by 43 synapomorphic characters, 17 of which are exclusive (Figure 4; Table 1). Finally, Commelinaceae is statistically strongly supported as monophyletic (BS = 91; PP = 1) by 22 synapomorphic characters, seven of which are exclusive (Figure 4; Table 1).

### 2.2. Infrafamilial Relationships

For the sake of conciseness, I will only describe the synapomorphies between family and genus. I will not go into any infrageneric relationships, unless they relate to issues on current and widely accepted generic circumscriptions.

#### 2.2.1. Philydraceae

**Figure 5:**
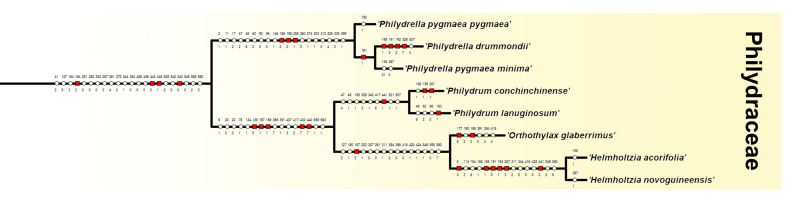
Maximum Parsimony consensus tree for Philydraceae, showing the character optimisation mapped using WinClada ver. 1.0000 over each branch of the cladogram. Homoplastic synapomorphies are shown as white circles, while exclusive synapomorphies are shown as red squares. The numbers above represent characters, while the ones below represent character states (as presented in Table S2).

*Philydrella* Caruel is recovered as sister to the rest of the family, followed by *Philydrum* Banks & Sol. ex Gaertn., with *Orthotylax* (Hook. f.) Skottsb. recovered as sister to *Helmholtzia* s.str. F.Muell. (Figure 2, Figure 3 and Figure 5). *Philydrella* is statistically strongly supported as monophyletic (BS = 100; PP = 1), being morphologically supported by 19 synapomorphies, three of them being exclusive (Figure 5; Table 3). The remaining genera in the family (i.e., [*Philydrum* [*Orthotylax* + *Helmholtzia* s.str.]]) are strongly statistically supported (BS = 82; PP = 0.53), being morphologically supported by 16 characters, of which five are exclusive (Figure 5; Table 2). *Philydrum* is statistically strongly supported as monophyletic (BS = 96; PP = 0.99), being morphologically supported by nine synapomorphies, with a single one being exclusive (Figure 5; Table 3). *Helmholtzia* s.lat. (i.e., [*Orthotylax* + *Helmholtzia* s.str.]) is statistically strongly supported (BS = 91; PP = 0.92), being morphologically supported by 15 synapomorphies, with only a single exclusive one (Figure 5; Table 2). *Orthotylax* is represented by its sole species and, thus, monophyletic by definition, being morphologically supported by five synapomorphies, two of which are exclusive (Figure 5; Table 3). Finally, *Helmholtzia* s.str. is strongly statistically supported as monophyletic (BS = 99; PP = 1), being morphologically supported by 15 synapomorphies, six of which are exclusive (Figure 5; Table 3).

#### 2.2.2. Pontederiaceae

**Figure 6:**
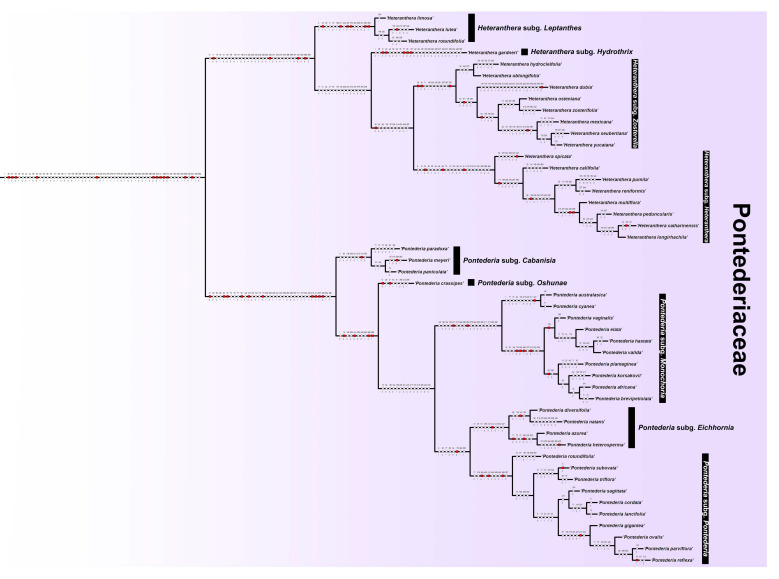
Maximum Parsimony consensus tree for Pontederiaceae, showing the character optimisation mapped using WinClada ver. 1.0000 over each branch of the cladogram. Homoplastic synapomorphies are shown as white circles, while exclusive synapomorphies are shown as red squares. The numbers above represent characters, while the ones below represent character states (as presented in Table S2).

*Heteranthera* Ruiz & Pav. sensu Pellegrini [56] is recovered as monophyletic with high statistical support (BS = 96; PP = 1). It is morphologically supported by 29 synapomorphies, of which four are exclusive (Figure 6; Table 3). The previously recognised *Eurystemon* Alexander, *Hydrothrix* Hook. f., *Scholleropsis* H.Perrier, and *Zosterella* Small are recovered nested in *Heteranthera*, supporting their synonymisation. *Pontederia* L. sensu Pellegrini et al. [25] is also recovered as monophyletic with robust statistical support (BS = 99; PP = 1), being morphologically supported by 35 synapomorphies, 10 of which are exclusive (Figure 6; Table 3). The previously recognised *Eichhornia* Kunth is recovered as polyphyletic, in three distinct lineages, while *Monochoria* C.Presl is recovered as monophyletic, but nested within the broadly circumscribed *Pontederia*.

#### 2.2.3. Haemodoraceae

**Figure 7:**
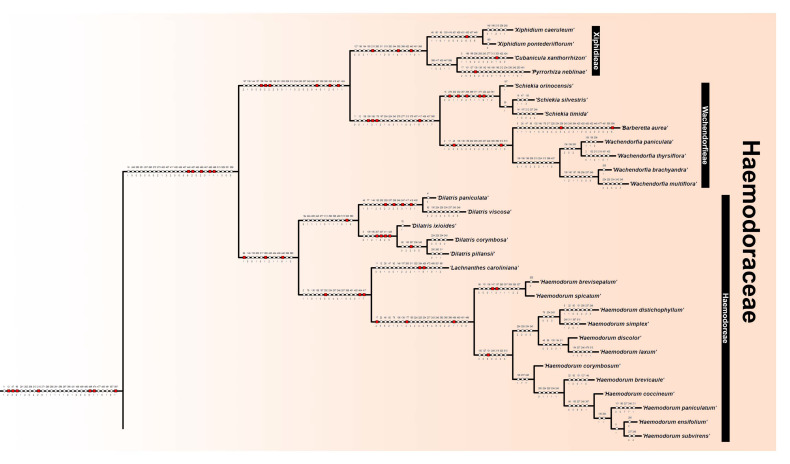
Maximum Parsimony consensus tree for Haemodoraceae (Haemodoroideae), showing the character optimisation mapped using WinClada ver. 1.0000 over each branch of the cladogram. Homoplastic synapomorphies are shown as white circles, while exclusive synapomorphies are shown as red squares. The numbers above represent characters, while the ones below represent character states (as presented in Table S2).

The family is divided into two main lineages, corresponding to its two accepted subfamilies (Figure 2 and Figure 3). Subfamily Haemodoroideae is statistically strongly supported (BS = 96; PP = 1), being morphologically supported by 25 synapomorphies, of which five are exclusive (Figure 7; Table 2). Haemodoroideae is recovered in the present analysis, arranged into three main clades (Figure 2, Figure 3 and Figure 7; Table 2): (1) the strongly supported (BS = 97; PP = 1) Haemodorum clade (i.e., tribe Haemodoreae); (2) the well-supported (BS = 88; PP = 1) Wachendorfia clade (i.e., tribe Wachendorfieae); and (3) the supported (BS = 87; PP = 1) Xiphidium clade (i.e., tribe Xiphidieae). Tribes Wachendorfieae and Xiphidieae are recovered as sister to each other, with high statistical support (BS = 87; PP = 1). This clade is morphologically supported by 24 synapomorphies, of which six are exclusive (Figure 7; Table 2). Tribe Xiphidieae is morphologically supported by 16 synapomorphies, three of them exclusive (Figure 7; Table 2). Inside Xiphidieae, *Xiphidium* Aubl. is strongly supported as monophyletic (BS = 98; PP = 1) by 11 synapomorphies (one of them exclusive), being recovered as sister to a clade consisting of *Cubanicula* Hopper et al. and *Pyrrorhiza* Maguire & Wurdack (Figure 7; Table 3). Both *Cubanicula* and *Pyrrorhiza* are monospecific and, thus, monophyletic by default. Their sister relationship is mildly supported (BS = 52; PP = 0.98) by five homoplastic synapomorphies, while *Cubanicula* is supported by 11 synapomorphies (one exclusive), and *Pyrrorhiza* is supported by 15 synapomorphies (one exclusive) (Figure 7; Table 3). Tribe Wachendorfieae is morphologically supported by 19 synapomorphies, of which four are exclusive (Figure 7; Table 2).

Inside Wachendorfieae, *Schiekia* Meisn. is strongly supported (BS = 88; PP = 1) as sister to *Barberetta* Harv. + *Wachendorfia* Burm. (or *Wachendorfia* s.lat.). *Schiekia* is recovered as monophyletic with high statistical support (BS = 99; PP = 1), supported by 12 synapomorphies, five of them being exclusive (Figure 7; Table 3). *Wachendorfia* s.lat. is strongly supported as monophyletic (BS = 91; PP = 1), supported by 15 synapomorphies, with two of them being exclusive (Figure 7; Table 3). *Barberetta* is monospecific and, thus, by default monophyletic, being morphologically supported by 23 synapomorphies, of which two are exclusive (Figure 7; Table 3). Nonetheless, *Wachendorfia* s.str. is inconsistently recovered as monophyletic, sometimes with *Barberetta* nested within it (Figure 2, Figure 3 and Figure 7), with no statistical support (BS = 0; PP = 0) and morphologically supported only by nine non-exclusive synapomorphies (Figure 7; Table 3). Tribe Haemodoreae is morphologically supported by 12 synapomorphies, of which three are exclusive (Figure 7; Table 3).

Inside Haemodoreae, *Dilatris* P.J.Bergius s.lat. is strongly recovered (BS = 98; PP = 1) as sister to *Haemodorum* Sm. and the monospecific *Lachnanthes* Elliott (Figure 2, Figure 3 and Figure 7). *Dilatris* s.lat. is supported by 12 synapomorphies, with only one being exclusive (Figure 7; Table 3). *Dilatris* s.lat. is recovered in the present study, arranged into two morphologically cohesive and statistically strongly supported clades (Figure 7; Table 3): (1) *Dilatris* s.str. (BS = 97; PP = 1), with mauve-coloured flowers, supported by seven synapomorphies (four exclusive), and (2) *Paradilatris* (Hopper ex J.C.Manning) Hopper (BS = 99; PP = 1), with yellow-coloured flowers, supported by 13 synapomorphies (four exclusive). The sister relationship between *Lachnanthes* and Haemodorum is strongly supported (BS = 95; PP = 1) by 15 synapomorphies, of which three are exclusive (Figure 7; Table 2). The monospecific *Lachnanthes* is morphologically supported by 16 synapomorphies, two of which are exclusive (Figure 7; Table 3). Finally, *Haemodorum* is strongly recovered as monophyletic (BS = 99; PP = 1), supported by 22 synapomorphies, of which three are exclusive (Figure 7; Table 3).

**Figure 8:**
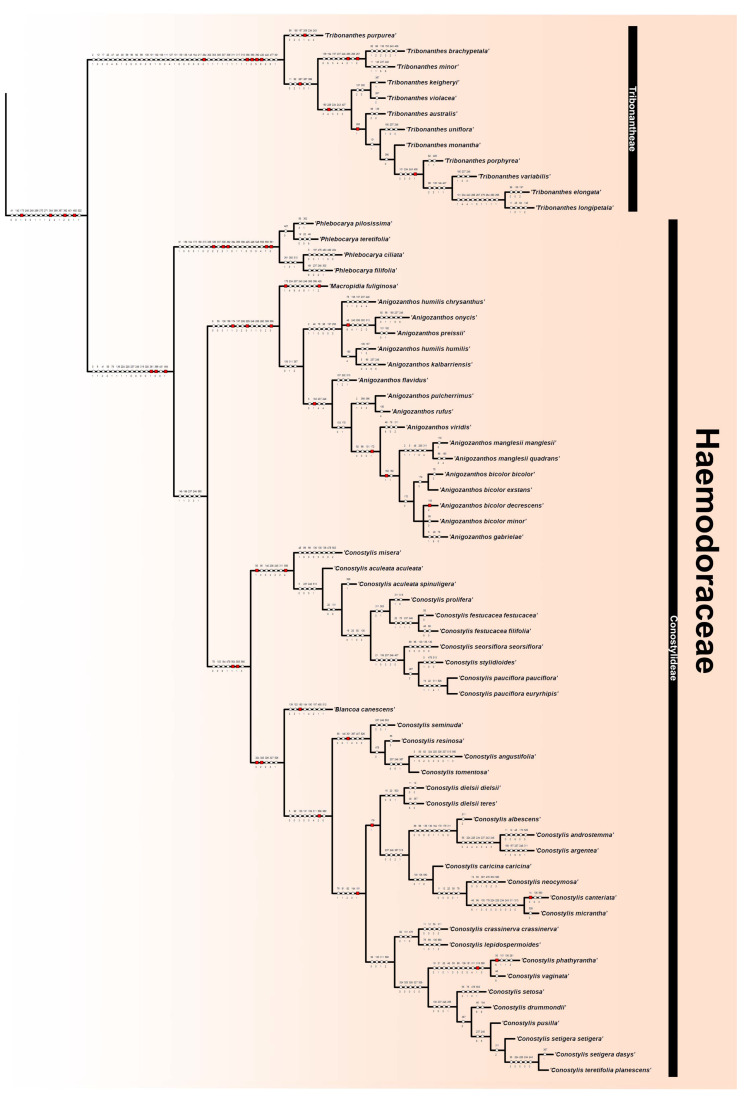
Maximum Parsimony consensus tree for Haemodoraceae (Conostylidoideae), showing the character optimisation mapped using WinClada ver. 1.0000 over each branch of the cladogram. Homoplastic synapomorphies are shown as white circles, while exclusive synapomorphies are shown as red squares. The numbers above represent characters, while the ones below represent character states (as presented in Table S2).

Subfamily Conostylidoideae is statistically strongly supported (BS = 82; PP = 1), being morphologically supported by 15 synapomorphies, four of them exclusive (Figure 7; Table 2). Conostylidoideae is recovered in the present analysis, organised into two main clades (Figure 2, Figure 3 and Figure 8; Table 2): (1) the very strongly supported (BS = 100; PP = 1) Tribonanthes clade (i.e., tribe Tribonantheae) and (2) the mildly supported (BS = 67; PP = 0.95) Conostylis clade (i.e., tribe Conostylideae). Tribe Tribonantheae consists solely of the genus *Tribonanthes* Endl., with both being morphologically supported by 39 synapomorphies, of which five are exclusive (Figure 8; Table 2 and Table 3). Tribe Conostylideae consists of *Phlebocarya* R.Br., *Anigozanthos* Labill. s.lat. (including the monospecific *Macropidia* J.Drumm. ex Harv.), and *Conostylis* R.Br. s.lat. (incl. the monospecific *Blancoa* Lindl.), being supported by 16 synapomorphies, of which three are exclusive (Figure 8; Table 2). *Phlebocarya* is strongly supported as monophyletic (BS = 99; PP = 1) by 20 synapomorphies, five of them being exclusive (Figure 8; Table 3). *Phlebocarya* is poorly statistically supported (BS = 63; PP = 0) as sister to *Anigozanthos* s.lat. and *Conostylis* s.lat., being morphologically supported by five non-exclusive synapomorphies (Figure 8; Table 2). *Anigozanthos* s.lat. is strongly recovered as monophyletic (BS = 95; PP = 1), being supported by 13 synapomorphies, with three being exclusive (Figure 8; Table 3). *Macropidia* is morphologically supported by eight synapomorphies, of which two are exclusive (Figure 8; Table 4). However, *Anigozanthos* s.str. is inconsistently recovered as monophyletic, sometimes with *Macropidia* nested within it (Figure 2, Figure 3 and Figure 8), with no statistical support (BS = 0; PP = 0) and morphologically supported only by three non-exclusive synapomorphies (Figure 8; Table 3). Finally, *Conostylis* s.lat. is well-supported as monophyletic (BS = 72; PP = 1), being supported by seven synapomorphies, with two being exclusive (Figure 8; Table 3). *Blancoa* is morphologically supported by eight synapomorphies with a single exclusive character (Figure 8; Table 3). However, *Conostylis* s.str. is inconsistently recovered as monophyletic, sometimes with *Blancoa* nested within it (Figure 2, Figure 3 and Figure 8), with no statistical support (BS = 0; PP = 0) and morphologically supported by eight synapomorphies, with only a single one of them being exclusive (Figure 8; Table 3).

#### 2.2.4. Hanguanaceae

**Figure 9:**
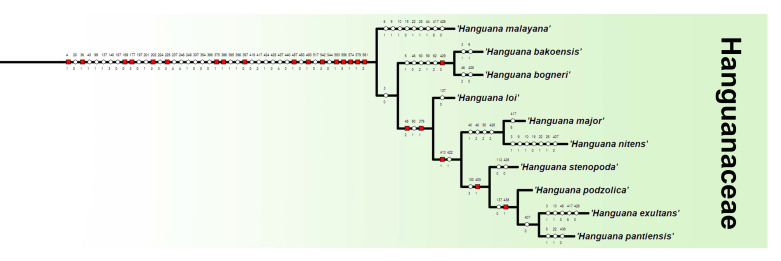
Maximum Parsimony consensus tree for Hanguanaceae, showing the character optimisation mapped using WinClada ver. 1.0000 over each branch of the cladogram. Homoplastic synapomorphies are shown as white circles, while exclusive synapomorphies are shown as red squares. The numbers above represent characters, while the ones below represent character states (as presented in Table S2).

Hanguanaceae is monogeneric and, thus, infrafamilial relationships in this group can be summarised into species-level relationships. For this reason, species-levels are briefly described below. The internal relationships in *Hanguana* Blume are mostly poorly supported, with *H. malayana* (Jack) Merr. being recovered sister to the rest of the genus, supported by a single non-exclusive synapomorphy (Figure 9; Table 3). The next lineage is a strongly supported (BS = 85; PP = 0.95) couplet consisting of *H. bakoënsis* Siti Nurfazilah et al. and *H. bogneri* Tillich & E.Sill, being supported by six synapomorphies, with a single one being exclusive (Figure 9). This couplet is sister to a statistically poorly supported clade (BS = 0; PP = 0.7), composed of *H. exultans* Siti Nurfazilah et al., *H. loi* Mohd Fahmi et al., *H. major* Airy Shaw, *H. nitens* Siti Nurfazilah et al., *H. pantiensis* Siti Nurfazilah et al., *H. podzolica* Siti Nurfazilah et al., and *H. stenopoda* Siti Nurfazilah et al. This clade is supported by two exclusive synapomorphies and a non-exclusive one (Figure 9). *Hanguana loi* is the first taxon in this clade, followed by a mildly supported (BS = 61; PP = 0.96) clade consisting of *H. major* and *H. nitens*, morphologically supported by four non-exclusive synapomorphies (Figure 9). This couplet is sister to the last statistically supported clade (BS = 55; PP = 0.8), consisting of *H. exultans*, *H. pantiensis*, *H. podzolica* and *H. stenopoda*, and morphologically supported by exclusive and non-exclusive synapomorphies (Figure 9).

#### 2.2.5. Commelinaceae

**Figure 10:**
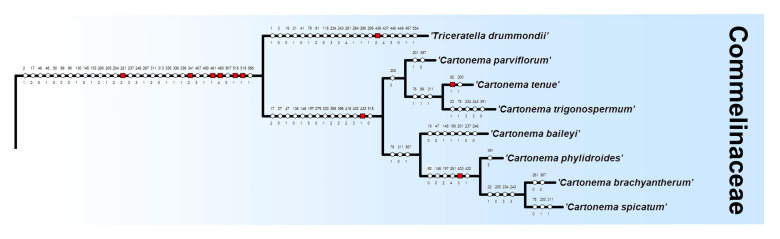
Maximum Parsimony consensus tree for Commelinaceae (Cartonematoideae), showing the character optimisation mapped using WinClada ver. 1.0000 over each branch of the cladogram. Homoplastic synapomorphies are shown as white circles, while exclusive synapomorphies are shown as red squares. The numbers above represent characters, while the ones below represent character states (as presented in Table S2).

**Figure 11:**
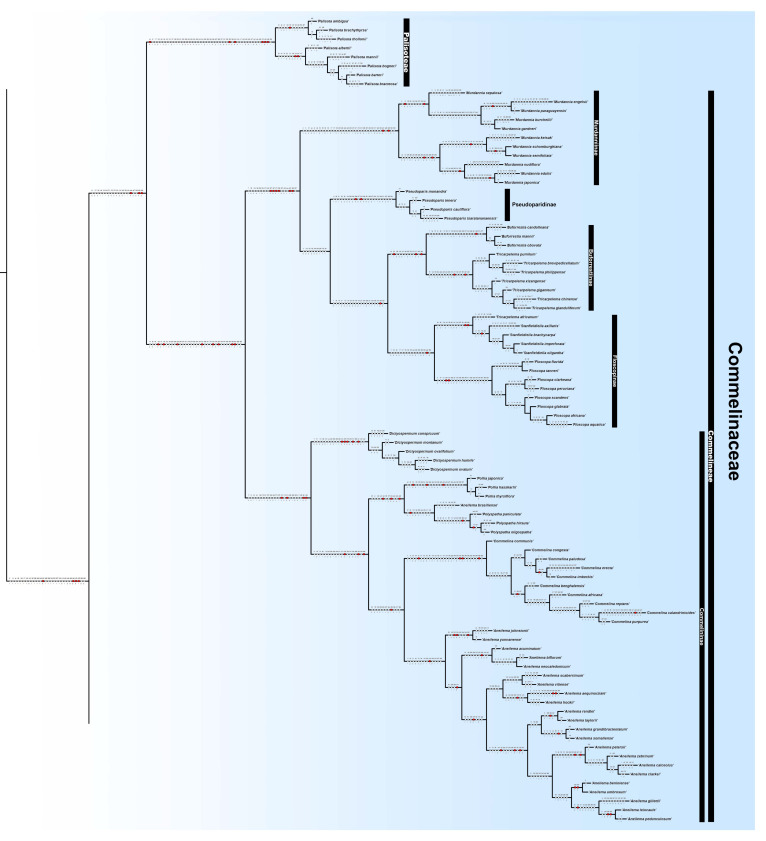
Maximum Parsimony consensus tree for Commelinaceae (Commelinoideae, Palisoteae and Commelineae), showing the character optimisation mapped using WinClada ver. 1.0000 over each branch of the cladogram. Homoplastic synapomorphies are shown as white circles, while exclusive synapomorphies are shown as red squares. The numbers above represent characters, while the ones below represent character states (as presented in Table S2).

The family is divided into four main lineages (Figure 2 and Figure 3; Table 4): (1) the strongly supported (BS = 99; PP = 1) subfamily Cartonematoideae; (2) the strongly supported (BS = 100; PP = 1) *Palisota* Rchb. *ex* Endl. (i.e., tribe Palisoteae); (3) the strongly supported (BS = 99; PP = 1) tribe Commelineae; and (4) the well-supported (BS = 58; PP = 1) tribe Tradescantieae, excluding *Palisota*. Subfamily Cartonematoideae is supported by 31 synapomorphies, with six of them being exclusive (Figure 10; Table 5). Internally, Cartonematoideae consists of *Cartonema* R.Br. and the monospecific *Triceratella* Brenan (and thus, the monogeneric tribes Cartonemateae and Triceratelleae). Consequently, *Triceratella* and Triceratelleae are monophyletic by default, being morphologically supported by 20 synapomorphies, with a single one being exclusive (Figure 10; Table 4 and Table 5). *Cartonema* is strongly supported (BS = 94; PP = 1) as monophyletic by 14 synapomorphies, one of which is exclusive (Figure 10; Table 4 and Table 5). Cartonematoideae is recovered as sister to the remaining three lineages, which represent subfamily Commelinoideae, which is well statistically supported (BS = 67; PP = 1) and morphologically supported by 28 synapomorphies, four of which are exclusive (Figure 10; Table 4). The relationships between the three main clades within Commelinoideae are poorly statistically supported. Palisoteae is inconsistently recovered as sister to Commelineae with some statistical support (BS = 0; PP = 0.92), but supported by 19 synapomorphies, of which three are exclusive (Figure 11; Table 4). Tribe Palisoteae (and as a consequence, *Palisota*) is morphologically supported by 45 synapomorphies, of which six are exclusive (Figure 11; Table 4 and Table 5).

Tribe Commelineae is morphologically supported by 34 synapomorphies, of which eight are exclusive (Figure 11; Table 4). Internally, Commelineae is arranged into two main groups (Figure 2, Figure 3 and Figure 10; Table 4): (1) the inconsistently monophyletic and statistically unsupported (BS = 0; PP = 0) Murdannia group (i.e., the non-monophyletic subtribe Murdanniinae s.lat.) and (2) the strongly monophyletic (BS = 98; PP = 1) subtribe Commelininae. The Murdannia group is represented by *Murdannia* Royle s.lat. (incl. *Anthericopsis* Engl.), *Buforrestia* C.B.Clarke, *Pseudoparis* H.Perrier, *Stanfieldiella* Brenan, and the non-monophyletic *Tricarpelema* J.K.Morton. Inside the Murdannia group, the lack of statistical support extends into its backbone (Table 4), with only the relationship between the Buforrestia (i.e., *Buforrestia* + *Tricarpelema* s.str.) and the Floscopa (i.e., [*Floscopa* + [*Stanfieldiella* + *Tricarpelema africanum* Faden]]) clades being weakly supported (BS = 53; PP = 0.58). *Murdannia* s.lat. is recovered as the first lineage, being strongly statistically supported (BS = 100; PP = 1), being morphologically supported by 34 synapomorphies, of which two are exclusive (Figure 11; Table 5). *Anthericopsis* is recovered nested within *Murdannia* s.lat., which prevents their recognition as distinct genera. The next lineage is *Pseudoparis*, which is strongly supported as monophyletic (BS = 100; PP = 1) by 22 synapomorphies, two of which are exclusive (Figure 11; Table 5). Despite the clade including *Pseudoparis* as sister to the Buforrestia clade (subtribe Buforrestiinae) and the Floscopa clade (subtribe Floscopinae) not being statistically supported, it is morphologically supported by nine non-exclusive synapomorphies (Figure 11; Table 4). The relationship between Buforrestiinae and Floscopinae is morphologically supported by 19 synapomorphies, with only a single one being exclusive (Figure 11; Table 4). Subtribe Buforrestiinae is well-supported as monophyletic (BS = 72; PP = 1) by 12 synapomorphies, of which three are exclusive (Figure 11; Table 4). Inside the Buforrestiinae, *Buforrestia* is strongly supported as monophyletic (BS = 100; PP = 1) by 20 synapomorphies, of which a single exclusive one (Figure 11; Table 5). *Tricarpelema* is recovered as non-monophyletic, due to *T. africanum* being recovered nested within subtribe Floscopinae. However, *Tricarpelema* s.str.is strongly supported (BS = 98; PP = 1) by 15 synapomorphies, with five being exclusive (Figure 11; Table 5). Floscopinae is strongly supported (BS = 95; PP = 1) by 15 synapomorphies, with only a single one being exclusive (Figure 11; Table 4). Inside subtribe Floscopinae, *Floscopa* is strongly supported as monophyletic (BS = 100; PP = 1) by 19 synapomorphies, of which two are exclusive (Figure 11; Table 5). *Floscopa* is recovered as sister to a strongly supported clade (BS = 96; PP = 1) consisting of *T. africanum* and *Stanfieldiella*, which is morphologically supported by 12 synapomorphies, of which two are exclusive (Figure 11; Table 4). *Tricarpelema africanum* is supported by six non-exclusive synapomorphies, while *Stanfieldiella* is well-supported as monophyletic (BS = 79; PP = 0.98) by three non-exclusive synapomorphies and a single exclusive synapomorphy (Figure 11; Table 5).

Subtribe Commelininae is morphologically supported by 22 synapomorphies, of which four are exclusive (Figure 11; Table 4). Internally, three main lineages are recovered (Figure 2, Figure 3 and Figure 10; Table 4): (1) *Dictyospermum* Wight; (2) the well-supported (BS = 79; PP = 1) Pollia clade; and (3) the mildly supported (BS = 66; PP = 0.99) Commelina clade. *Dictyospermum* is strongly supported as monophyletic (BS = 99; PP = 1) by 19 synapomorphies, with five of them being exclusive (Figure 11; Table 5). The Pollia and Commelina clades are strongly recovered (BS = 98; PP = 1) as sister to each other, supported by 19 synapomorphies, of which three are exclusive (Figure 11; Table 4). The Pollia clade consists of *Pollia* Thunb. s.str., *Polyspatha* Benth., and *Aneilema brasiliense* C.B.Clarke, being supported by 11 synapomorphies, with three of them being exclusive (Figure 11; Table 4). *Pollia* s.str. is highly supported (BS = 100; PP = 1) by 21 synapomorphies, with three of them being exclusive (Figure 11; Table 5). *Pollia* s.str. is recovered as sister to *Polyspatha* and *A. brasiliense*, being strongly supported (BS = 97; PP = 1) by nine synapomorphies, with a single one of them being exclusive (Figure 11; Table 4). *Aneilema brasiliense* is supported by seven non-exclusive synapomorphies, while *Polyspatha* is strongly supported (BS = 99; PP = 1) by 11 synapomorphies, with a single one of them being exclusive (Figure 11; Table 5). The Commelina clade consists of *Aneilema* R.Br. s.lat. (excl. *A. brasiliense* but incl. *Rhopalephora* Hassk.) and *Commelina* Plum. ex L. s.lat. (incl. the monospecific *Tapheocarpa* Conran), and is supported by 11 synapomorphies, of which one is exclusive (Figure 11; Table 4). *Commelina* s.lat. is highly supported as monophyletic (BS = 100; PP = 1) by 28 synapomorphies, with four of them being exclusive (Figure 11; Table 5). *Tapheocarpa calandrinioides* (F.Muell.) Conran is recovered nested deep within *Commelina*, which prevents its recognition as a distinct genus (Figure 11). Finally, *Aneilema* s.lat. is well-supported (BS = 75; PP = 1) by 13 synapomorphies, of which only a single one is exclusive (Figure 11; Table 5). *Rhopalephora* is recovered deeply nested within *Aneilema*, preventing its recognition as a distinct genus.

**Figure 12:**
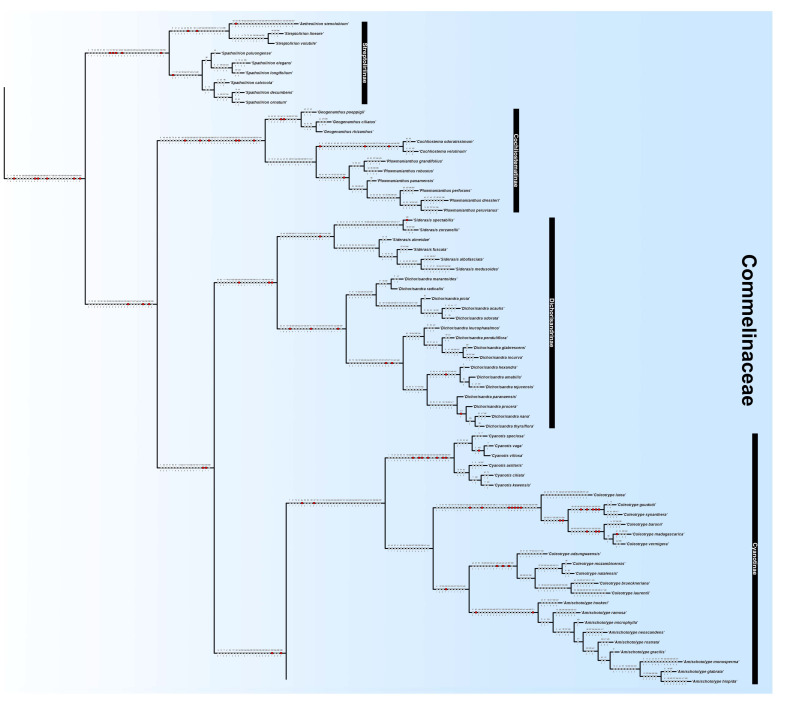
Maximum Parsimony consensus tree for Commelinaceae (Commelinoideae, Tradescantieae part 1), showing the character optimisation mapped using WinClada ver. 1.0000 over each branch of the cladogram. Homoplastic synapomorphies are shown as white circles, while exclusive synapomorphies are shown as red squares. The numbers above represent characters, while the ones below represent character states (as presented in Table S2).

Tribe Tradescantieae is morphologically supported by 25 synapomorphies, six of which are exclusive (Figure 12; Table 4). Internally, Tradescantieae is divided into five main lineages (Figure 2, Figure 3, Figure 11 and Figure 12; Table 4): (1) the very strongly supported (BS = 99; PP = 1) subtribe Streptoliriinae; (2) the very strongly supported (BS = 100; PP = 1) subtribe Cochliostematinae; (3) the mildly supported (BS = 58; PP = 1) subtribe Dichorisandrinae s.str.; (4) the very strongly supported (BS = 99; PP = 1) Cyanotinae s.lat.; and (5) the well-supported (BS = 71; PP = 1) Tradescantia alliance (subtribe Tradescantiinae s.lat.). Tradescantieae minus Streptoliriinae (i.e., [Cochliostematinae [Dichorisandrinae s.str. [Cyanotinae s.lat. + Tradescantia alliance]]]) is somewhat statistically supported (BS = 0; PP = 1), but morphologically supported by 22 synapomorphies, three of them being exclusive (Figure 12; Table 4). The [Dichorisandrinae [Cyanotinae s.lat. + Tradescantia alliance]] clade is also inconsistently statistically supported (BS = 0; PP = 0.83), but morphologically supported by 17 synapomorphies, two of them being exclusive (Figure 12; Table 4). Finally, the Cyanotinae s.lat. + Tradescantia alliance clade is statistically well-supported (BS = 84; PP = 1), being morphologically supported by 22 synapomorphies, of which two are exclusive (Figure 12; Table 4). Subtribe Streptoliriinae is morphologically supported by 26 synapomorphies, with five of them being exclusive (Figure 12; Table 4). Inside Streptoliriinae, *Spatholirion* Ridl. is well-supported as monophyletic (BS = 84; PP = 1) by nine synapomorphies, with a single one of them being exclusive (Figure 12; Table 5). *Spatholirion* is recovered as sister to a clade consisting of *Aëtheolirion* Forman + *Streptolirion* Edgew., which is strongly supported (BS = 94; PP = 1) by 188 synapomorphies, of which two are exclusive (Figure 12; Table 4). The monospecific *Aëtheolirion* is monophyletic by default, being supported by 21 characters, with a single one of them being exclusive, while *Streptolirion* is strongly supported as monophyletic (BS = 99; PP = 1) by 11 non-exclusive synapomorphies (Figure 12; Table 5).

Subtribe Cochliostematinae is supported by 34 synapomorphies, with six of them being exclusive (Figure 12; Table 4). *Geogenanthus* Ule is strongly supported as monophyletic (BS = 99; PP = 1) by 10 synapomorphies, of which two are exclusive (Figure 12; Table 5). *Geogenanthus* is recovered as sister to a strongly supported clade (BS = 95; PP = 1) consisting of *Cochliostema* Lem. + *Plowmanianthus* Faden & C.R.Hardy, morphologically supported by 15 non-exclusive synapomorphies (Figure 12; Table 4). *Cochliostema* is strongly supported as monophyletic (BS = 100; PP = 1) by 27 synapomorphies, of which three are exclusive (Figure 12; Table 5), while *Plowmanianthus* is strongly supported as monophyletic (BS = 95; PP = 1) by nine synapomorphies, with a single one being exclusive (Figure 12; Table 5). Subtribe Dichorisandrinae s.str. consists of *Dichorisandra* J.C.Mikan and *Siderasis* Raf. emend. M.Pell. & Faden, being morphologically supported by 19 synapomorphies, of which three are exclusive (Figure 12; Table 4). *Dichorisandra* is strongly supported as monophyletic (BS = 97; PP = 1) by 21 synapomorphies, with one of them being exclusive (Figure 12; Table 5), while *Siderasis* is also strongly supported as monophyletic (BS = 97; PP = 1) by 17 synapomorphies, also with a single one being exclusive (Figure 12; Table 5). Subtribe Cyanotinae s.lat. is morphologically supported by 31 synapomorphies, of which two are exclusive (Figure 12; Table 4). It consists of *Amischotolype* Hassk. s.lat. (incl. *Porandra* D.Y.Hong), *Cyanotis* D.Don s.lat. (incl. *Belosynapsis* Hassk.), and the non-monophyletic *Coleotrype* C.B.Clarke, which is recovered in two separate lineages (Figure 2, Figure 3 and Figure 11). *Cyanotis* s.lat. is recovered sister to the remaining genera, being very strongly supported as monophyletic (BS = 100; PP = 1) by 21 synapomorphies, with seven of them being exclusive (Figure 12; Table 5). Furthermore, *Belosynapsis* is recovered as non-monophyletic and nested within *Cyanotis*, preventing its recognition as an independent genus. The clade consisting of [*Coleotrype* pro parte + [*Coleotrype* s.str. + *Amischotolype* s.lat.]] (i.e., subtribe Coleotrypinae) is not statistically supported (BS = 0; PP = 0), but is morphologically supported by 14 non-exclusive synapomorphies (Figure 12; Table 4). *Coleotrype* pro parte (i.e., the Malagasy species of *Coleotrype*) is recovered as an inconsistently supported clade (BS = 100; PP = 0), morphologically supported by 34 synapomorphies, of which seven are exclusive (Figure 12; Table 5). The clade consisting of *Coleotrype* s.str. + *Amischotolype* s.lat. is not statistically supported (BS = 0; PP = 0), but is morphologically supported by 10 synapomorphies, with one of them being exclusive (Figure 12; Table 4). *Coleotrype* s.str. is statistically well-supported (BS = 83; PP = 1), being morphologically supported by 14 synapomorphies, of which three are exclusive (Figure 12; Table 5). *Amischotolype* s.lat. is strongly supported as monophyletic (BS = 94; PP = 1) by 21 synapomorphies, two of which are exclusive (Figure 12; Table 5). Furthermore, *Porandra* is recovered as non-monophyletic in a polytomy with the remaining species of *Amischotolype* s.str., which prevents their recognition as distinct genera.

**Figure 13:**
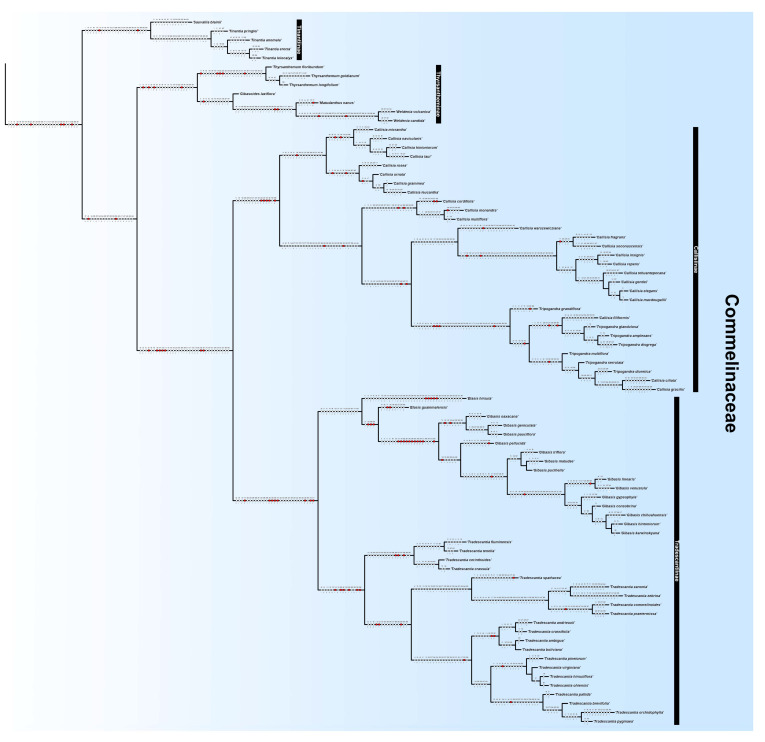
Maximum Parsimony consensus tree for Commelinaceae (Commelinoideae, Tradescantieae part 2), showing the character optimisation mapped using WinClada ver. 1.0000 over each branch of the cladogram. Homoplastic synapomorphies are shown as white circles, while exclusive synapomorphies are shown as red squares. The numbers above represent characters, while the ones below represent character states (as presented in Table S2).

Subtribe Tradescantiinae s.lat. sensu Pellegrini [28] (or the Tradescantia alliance sensu Hertweck & Pires [57]) is recovered as monophyletic, morphologically supported by 26 synapomorphies, with five of them being exclusive (Figure 13; Table 4). Internally, it is arranged into three main lineages (Figure 2, Figure 3 and Figure 12; Table 4): (1) the strongly supported (BS = 96; PP = 1) Tinantia clade; (2) the strongly supported (BS = 98; PP = 1) Thyrsanthemum clade (i.e., subtribe Thyrsantheminae s.str.); and (3) the strongly supported (BS = 99; PP = 1) Tradescantia clade. The Tinantia clade is morphologically supported by 23 characters, with two of them being exclusive (Figure 13; Table 4). However, due to the amount of missing data in the matrix for the poorly understood *Sauvallia* Wright ex Hassk., both characters are most accurately exclusive synapomorphies for *Tinantia* Scheidw. *Sauvallia* is monospecific and by default monophyletic, being morphologically supported by 13 non-exclusive synapomorphies (Figure 13; Table 5). *Tinantia* is strongly supported as monophyletic (BS = 95; PP = 1), morphologically supported by 20 non-exclusive synapomorphies, in addition to the two aforementioned exclusive ones (Figure 13; Table 5). The Thyrsanthemum clade represents a narrower circumscription of the old subtribe Thyrsantheminae (i.e., excl. *Elasis* D.R.Hunt and *Tinantia*), being morphologically supported by 20 synapomorphies, of which four are exclusive (Figure 13; Table 4). This clade consists of *Gibasoides* D.R.Hunt, *Matudanthus* D.R.Hunt, *Thyrsanthemum* Pichon s.str., and *Weldenia* Schult. f., with *Thyrsanthemum* s.str. being strongly supported as monophyletic (BS = 100; PP = 1) by 23 synapomorphic characters, five of which are exclusive (Figure 13; Table 5). Additionally, its expansion to include the monospecific *Gibasoides* is not supported by this analysis, as *Gibasoides laxiflora* (C.B.Clarke) D.R.Hunt is recovered as sister to *Matudanthus* + *Weldenia* (Figure 13; Table 4). Nonetheless, the present analysis and dataset have been unable to recover any synapomorphies for the genus (Figure 13; Table 5). The clade including [*Gibasoides* [*Matudanthus* + *Weldenia*]] is statistically well-supported (BS = 87; PP = 1), being morphologically supported by 11 synapomorphies, of which a single one is exclusive (Figure 13; Table 5). The clade consisting of *Matudanthus* + *Weldenia* is well-supported (BS = 89; PP = 1), being morphologically supported by 21 synapomorphies, of which two are exclusive (Figure 13; Table 4). *Matudanthus* is monospecific and monophyletic by default, being supported by six synapomorphies, with one of them being exclusive (Figure 13; Table 5), while *Weldenia* is very strongly supported as monophyletic (BS = 100; PP = 1), being supported by 28 synapomorphies, with two of them being exclusive (Figure 13; Table 5).

The Tradescantia clade is morphologically supported by 33 synapomorphies, with seven of them being exclusive (Figure 13; Table 4). The Tradescantia clade is arranged into two main lineages (Figure 2, Figure 3 and Figure 12; Table 4): (1) the well-supported (BS = 76; PP = 1) Callisia group (subtribe Callisiinae) and (2) the well-supported (BS = 91; PP = 1) Tradescantia group (subtribe Tradescantiinae s.str.). Subtribe Callisiinae corresponds to the very broadly circumscribed *Callisia* Loefl. (sensu Christenhusz et al. 2018 [58]), which also includes *Tripogandra* Raf. It is morphologically supported by 15 synapomorphic characters, with five of them being exclusive (Figure 13; Table 4). Internally, subtribe Callisiinae is further divided into six lineages (Figure 2, Figure 3 and Figure 12; Table 5): (1) the well-supported (BS = 72; PP = 1) Navicularis group; (2) the strongly supported (BS = 98; PP = 1) Rosea group (i.e., *Cuthbertia* Small); (3) the strongly supported (BS = 98; PP = 1) Monandra group (i.e., *Aploleia* Raf.); (4) the highly supported (BS = 100; PP = 1) Warszewicziana group (i.e., *Hadrodemas* H.E.Moore); (5) the strongly supported (BS = 99; PP = 1) Repens group (i.e., *Callisia* s.str.); and (6) the strongly supported (BS = 99; PP = 1) *Tripogandra* s.lat. [incl. *Tradescantia triandra* Kunth (≡ *Callisia ciliata* Kunth, nom. illeg.), *C. filiformis* (M.Martens & Galeotti) D.R.Hunt, and *C. gracilis* (Kunth) D.R.Hunt]. The Navicularis group and *Cuthbertia* are well-supported (BS = 86; PP = 1) as sister to each other, morphologically supported by 15 synapomorphies, with a single one of them being exclusive (Figure 13; Table 4). The Navicularis group is morphologically supported by eight synapomorphies (two of them exclusive), while *Cuthbertia* is supported by 10 synapomorphies (with two of them being exclusive) (Figure 13; Table 5). The Navicularis group + *Cuthbertia* clade is recovered as sister to the remaining lineages of subtribe Callisiinae, which are strongly supported (BS = 92; PP = 1) by 28 synapomorphies, with two of them being exclusive (Figure 13; Table 4). The next lineage to diverge is *Aploleia*, which is morphologically supported by 18 synapomorphies, two of which are exclusive (Figure 13; Table 5). *Aploleia* is sister to the clade consisting of [*Tripogandra* s.lat. [*Hadrodemas* + *Callisia* s.str.]], which is well-supported (BS = 71; PP = 1) by 16 synapomorphies, with two of them being exclusive (Figure 13; Table 4). *Tripogandra* s.lat. is morphologically supported by 34 synapomorphies, four of which are exclusive (Figure 13; Table 5). *Callisia ciliata* is recovered as sister to *C. gracilis*, nested within a clade with *T. multiflora* Raf. (the type species of *Tripogandra*), while *C. filiformis* is recovered in a clade with *T. disgrega* (Kunth) Woodson and other morphologically similar species. These relationships preclude the monophyly of *Tripogandra*, unless these three species are transferred to it. The *Hadrodemas* + *Callisia* s.str. clade is poorly supported (BS = 51; PP = 0) by 15 non-exclusive synapomorphies (Figure 13; Table 4). *Hadrodemas* is monospecific and monophyletic by default, being morphologically supported by 20 synapomorphies, with a single one being exclusive (Figure 13; Table 5). *Callisia* s.str. is morphologically supported by 34 synapomorphies, of which two are exclusive (Figure 13; Table 5).

Subtribe Tradescantiinae s.str. consists of *Elasis* D.R.Hunt s.lat., *Gibasis* Raf., and *Tradescantia* Ruppius ex L. emend. M.Pell., and is morphologically supported by 29 synapomorphies, of which eight are exclusive (Figure 13; Table 4). *Tradescantia* is well-supported as monophyletic (BS = 60; PP = 1) by 15 synapomorphies, with six of them being exclusive (Figure 13; Table 5). *Tradescantia* is poorly supported (BS = 60; PP = 0) as sister to a clade consisting of *Elasis* s.lat and *Gibasis*, morphologically supported by 14 non-exclusive synapomorphies (Figure 13; Table 4). *Elasis* s.lat. is recovered as non-monophyletic due to *E. guatemalensis* (C.B.Clarke ex Donn.Sm.) M.Pell. being recovered as sister to *Gibasis*, instead of in a clade with *E. hirsuta* (Kunth) D.R.Hunt. *Elasis hirsuta* (i.e., *Elasis* s.str.) is morphologically supported by 36 synapomorphies, of which five are exclusive (Figure 13; Table 5). The *Elasis guatemalensis* + *Gibasis* clade is well-supported (BS = 62; PP = 1) by three exclusive synapomorphies and one non-exclusive synapomorphy (Figure 13; Table 4). *Elasis guatemalensis* is morphologically supported by nine synapomorphies, with two of them being exclusive (Figure 13; Table 5). Finally, *Gibasis* is well-supported as monophyletic (BS = 69; PP = 1) by 20 synapomorphies, of which 11 are exclusive (Figure 13; Table 5).

### 2.3. Taxonomy

In the present study, I recognize two suborders, one superfamily, and five families in Commelinales. I adopt a phylogenetic classification for the order, in which only monophyletic groups are recognised [59,60,61], meaning that each recognised group should be: (1) consistent in all equally parsimonious trees; (2) defined by observable morphological synapomorphies; and (3) preferably statistically well-supported. Monospecific taxa have been avoided whenever possible, since they provide little to no information regarding phylogenetic relationships, making the classification redundant [59,60,61]. In the same way, infrafamilial taxa (e.g., subfamilies, tribes, subtribes, genera, etc.) are generally recognised if they represent stable and well-supported clades, composed of more than one subordinate taxon. This is done to facilitate the application and recognition of names above the species level, making the group’s taxonomy and classification more accessible to non-specialists.

Taxa are presented below in a phylogenetic sequence, being secondarily arranged in alphabetical order. The following taxonomic realignments are necessary to ensure Commelinales only recognises monophyletic taxa. Finally, several taxonomic changes are also provided here, in order to facilitate the application and conservation of several taxa by recognising them at the species rank. Thus, infraspecific taxa are not recognised in Commelinales whenever possible. A summary of the proposed classification is presented in Table 6. A complete linear classification for Commelinales, including full synonymy, is presented in List S1, summarising all accepted taxa down to genus.

#### 2.3.1. Philydraceae

For Philydraceae, two taxonomic updates are needed and are implemented below:

2.3.1.a. ***Philydrella*** Caruel, Nuovo Giorn. Bot. Ital. 10: 91. 1878. Type species. *Philydrella pygmaea* (R.Br.) Caruel.

Figure 14G

= *Hetaeria* Endl. Gen. Pl.: 133. 1836, nom, illeg. non *Hetaeria* Blume, Bijdr. Fl. Ned. Ind. 8: 409. 1825. Type species. *Hetaeria pygmaea* (R.Br.) Endl. [≡ *P. pygmaea* (R.Br.) Caruel].

= *Pritzelia* F.Muell. Descr. Notes Papuan Pl. 1: 13. 1875, nom. illeg. non *Pritzelia* Walp., Repert. 2: 428. 1843, nec *Pritzelia* Schauer Flora 26: 407. 1843. Type species. *Pritzelia pygmaea* (R.Br.) F.Muell. ex Benth. [≡ *P. pygmaea* (R.Br.) Caruel].

= *Philydrum* sect. *Pritzelia* Baill., Hist. Pl. 13: 234. 1894. Type species. *Philydrum pygmaeum* R.Br. [≡ *P. pygmaea* (R.Br.) Caruel].

**Distribution.** Endemic to Western Australia.

**Ecology.** Found growing in freshwater swamps, seasonally flooded white-sand formations, and seepage areas.

**Comments.** *Philydrella* can be easily differentiated from the other three genera of Philydraceae by its diminute cormose habit, rhomboid outer perianth lobes, thin filament, expanded connective, pollen grains with tectum verrucose, and seeds with reticulate testa.

**Accepted species and new combination.** A small genus represented by three morphologically similar species: *Philydrella drummondii* L.G.Adams, *P. minima* (L.G.Adams) M.Pell., and *P. pygmaea* (R.Br.) Caruel.

2.3.1.a.i. ***Philydrella minima*** (L.G.Adams) M.Pell., **stat. nov.** ≡ *Philydrella pygmaea* subsp. *minima* L.G.Adams, Fl. Australia 45: 454. 1987.

urn:lsid:ipni.org:names:77379616-1 (https://www.ipni.org/n/77379616-1)

2.3.1.b. ***Philydrum*** Banks & Sol. ex Gaertn., Fruct. Sem. Pl. 1: 62. 1788 ≡ *Philydrum* Banks & Sol. ex Gaertn. sect. *Philydrum*. Type species. *Philydrum lanuginosum* Banks & Sol. ex Gaertn.

Figure 14H

**Distribution.** Known to occur in Australia (New South Wales, Northern Territory, Queensland, Victoria, Western Australia), Southeast China, India (Andaman Islands), Japan (Ryukyu Islands), Malaysia, Myanmar, Micronesia, New Guinea, Palau, Taiwan, Thailand, and Vietnam.

**Ecology.** It grows in freshwater swamps and drainage areas.

**Comments.** *Philydrum* is unique in possessing spongy and fistulose leaves, plicate outer perianth lobes, filament stout and medially broadened, inconspicuous connective, pollen release in tetrads with foveolate tectum, and seed testa spirally striate.

**Accepted species and new combination.** A total of two species: *Philydrum cochinchinense* (Lour.) M.Pell. and *P. lanuginosum* Banks & Sol. ex Gaertn.

2.3.1.b.i. ***Philydrum cochinchinense*** (Lour.) M.Pell., **comb. nov.** ≡ *Garciana cochinchinensis* Lour., Fl. Cochinch.: 15. 1790.

urn:lsid:ipni.org:names:77379617-1 (https://www.ipni.org/n/77379617-1)

#### 2.3.2. Pontederiaceae

In and related to Pontederiaceae, taxonomic updates are needed above the family level and under *Heteranthera*. Above the family rank, it refers to the recognition of a superfamily that includes Pontederiaceae and Haemodoraceae. While under *Heteranthera*, it refers to the recognition of subgenera, in a similar fashion as previously proposed for *Pontederia*.

2.3.2.a. **Pontederiorae** M.Pell., **supfam. nov.** Type family. Pontederiaceae Kunth (*Pontederia* L.).

**Figure 14:**
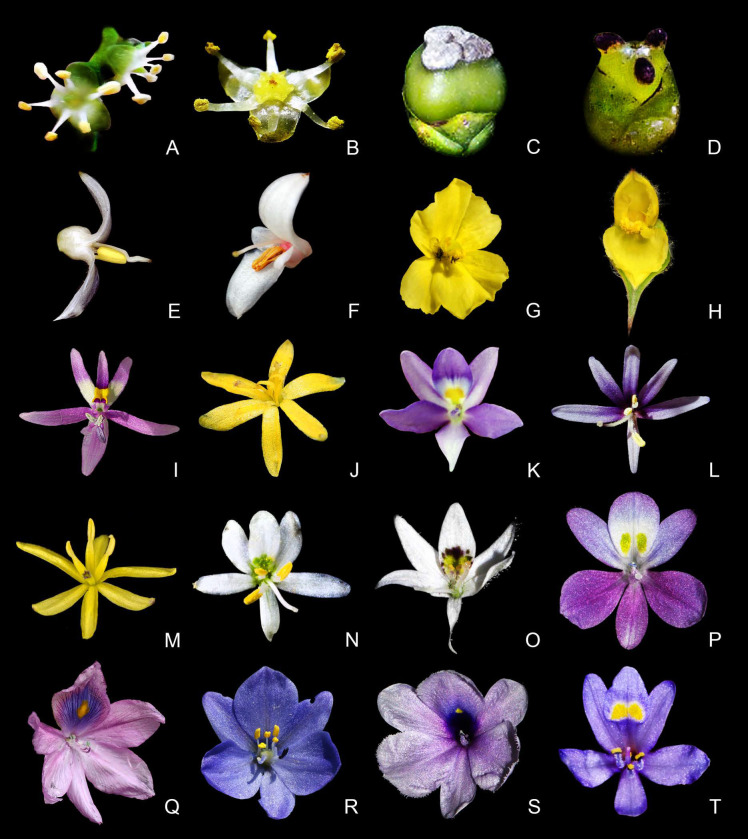
Floral morphology of Hanguanaceae, Philydraceae, and Pontederiaceae. (**A–D**), Hanguanaceae: (**A**), staminate flower of *Hanguana* sp. (**B**), pistillate flower of *H. anthelminthica* (Blume *ex* Schult. & Schult. f.) Masam. (**C**), pistillate flower of *H. anthelminthica* starting fruit development. (**D**), pistillate flower of *H. nitens* Siti Nurfazilah et al. (**E**–**H**), Philydraceae: (**E**), *Helmholtzia acorifolia* F.Muell. (**F**), *Orthothylax glaberrimus* (Hook. f.) Skottsb. (**G**), *Philydrella pygmaea* (R.Br.) Caruel. (**H**), *Philydrum cochinchinense* (Lour.) M.Pell. (**I**–**T**), Pontederiaceae: (**I**), *Heteranthera rotundifolia* (Kunth) Griseb.; (**J**), *H. gardneri* (Hook.) M.Pell.; (**K**), *H. oblongifolia* Mart. ex Schult & Schult. f.; (**L**), *H. zosterifolia* Mart.; (**M**), *H. dubia* (Jacq.) MacMill.; (**N**), *H. callifolia* Rchb. ex Kunth; (**O**), *H. reniformis* Ruiz & Pav.; (**P**), *Pontederia paniculata* Spreng.; (**Q**), *P. crassipes* Mart.; (**R**), *P. korsakowii* (Regel & Maack) M.Pell. & C.N.Horn; (**S**), *Pontederia azurea* Sw.; (**T**), *P. cordata* L. A by M. Niissalo, B & D by J. Leong-Škorničková, C by K.H. Ong, E by B. Jago, F by L. Lambrianides, G by B. Kin, H by J.-R. Lin, I by A. Popovkin, J by C.P. Bove, K by F.F.S. Silva, L by S.S. Oliveira, M by G. Carr, N by E. Bidault, O–Q and S–T by M.O.O. Pellegrini, and R by Ashitaka.

**Figure 15:**
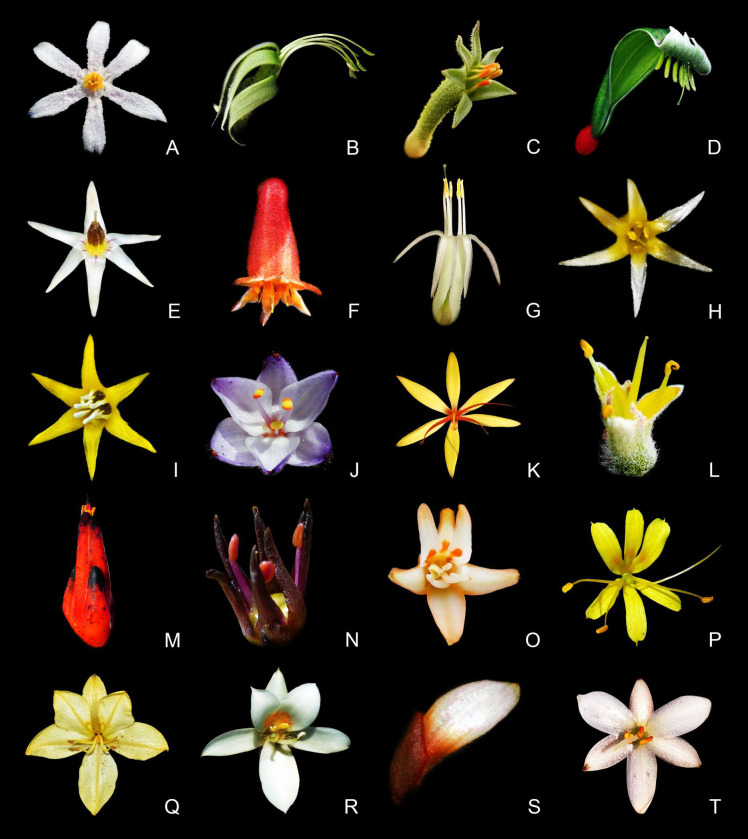
Floral morphology of Haemodoraceae. (**A**), *Tribonanthes longipetala* Lindl. (**B**), *Anigozanthos fuliginosus* Hook.; (**C**), *Anigozanthos flavidus* DC.; (**D**), *Anigozanthos manglesii* D.Don; (**E**), *Phlebocarya ciliata* R.Br.; (**F**), *Conostylis canescens* (Lindl.) F.Muell.; (**G**), *Conostylis androstemma* F.Muell.; (**H**), *Conostylis setigera* R.Br.; (**I**), *Conostylis vaginata* Endl. (**J**), *Dilatris ixioides* Lam.; (**K**), *Paradilatris viscosa* (L. f.) Hopper; (**L**), *Lachnanthes caroliniana* (Lam.) Dandy; (**M**), *Haemodorum coccineum* R.Br.; (**N**), *Haemodorum simplex* Lindl. (**O**), *Schiekia orinocensis* (Kunth) Meisn.; (**P**), *Wachendorfia aurea* (Harv.) M.Pell.; (**Q**), *Wachendorfia paniculata* Burm.; (**R**), *Cubanicula xanthorrhizos* (C.Wright ex Griseb.) Hopper et al.; (**S**), *Pyrrorhiza neblinae* Maguire & Wurdack; (**T**), *Xiphidium caeruleum* Aubl. A & F by J. Hort & F. Hort, B–D, H–K & Q by J.T. Johansson, E by M. Brundrett, G by K. Thiele, L by M. Keim, M by R. Cumming, N by J. Tann, O by E.J. Hickman, P by C. McMaster, R by R.J. Smith, S by A. Weitzman, and T by A. Yakovlev.

Figure 14I–T and Figure 15

**Validation.** Based on the Latin description associated with Pontederiaceae Kunth in F.W.H.A. von Humboldt, A.J.A. Bonpland & C.S. Kunth, Nov. Gen. Sp. 1, ed. qu.: 265. Mai 1816, *nom. cons*.

**Included families.** Haemodoraceae and Pontederiaceae.

**Notes.** Despite not being currently recognised by the Code [62] (Art. 41), superranks help formalise phylogenetic relationships and have also been used in the sister order, Zingiberales [40]. Thus, in anticipation that the Code might be updated in the future to recognise these taxonomic ranks, I am choosing to publish this name.

2.3.2.b. ***Heteranthera*** Ruiz & Pav., Fl. Peruv. Prodr.: 9. 1794. Type species. *Heteranthera reniformis* Ruiz & Pav.

Figure 14I–O

**Distribution.** *Heteranthera* has a Pantropical distribution, but most of its species are concentrated in the Neotropical region, especially in Brazil [24,56,63].

**Ecology.** Its species are found growing in permanent or seasonal freshwater environments, with some species also commonly found growing as weeds in rice fields and other flooded plantations.

**Comments.** *Heteranthera* is easily differentiated from *Pontederia* by leaf morphology, inflorescence architecture, and floral morphology.

**Infrageneric classification.** *Heteranthera* was traditionally divided into sections by previous authors. However, this classification was abandoned by Horn [63] in his taxonomic revision of the genus, in which he accepted *Hydrothrix* Hook. f., *Scholleropsis* H.Perrier, and *Zosterella* Small as distinct genera. Rosatti [64] disagreed with Horn’s circumscription and reduced *Zosterella* to a subgenus of *Heteranthera*. Pellegrini [56] reduced *Hydrothrix* and *Scholleropsis* to synonyms of *Heteranthera*, but made no combinations either for the subgeneric or sectional ranks. With the present study sampling all currently accepted species of *Heteranthera*, it seems convenient to propose a new infrageneric classification, based on monophyletic and morphologically well-defined groupings. Four subgenera are recognised.

**Accepted species.** *Heteranthera* is a small genus with ca. 30 species, several still undescribed.

2.3.2.b.i. ***Heteranthera* subg. *Leptanthus*** (Michx.) M.Pell. & C.N.Horn, **comb. & stat. nov.** ≡ *Heteranthera* sect. *Leptanthus* (Michx.) Solms. in A.L.P.P. de Candolle & A.C.P. de Candolle, Monogr. Phan. 4: 518. 1883 ≡ *Leptanthus* Michx. subg. *Leptanthus* ≡ *Leptanthus* Michx., Fl. Bor.-Amer. 1: 24. 1803. Type species (designated here). *Leptanthus ovalis* Michx., nom. superfl. [≡ *H. limosa* (Sw.) Willd.].

urn:lsid:ipni.org:names:77379618-1 (https://www.ipni.org/n/77379618-1)

Figure 14I

≡ *Lunania* Raf., Med. Fl. 2: 106. 1830, nom. rej. non *Lunania* Hook., nom. cons., London J. Bot. 3: 317. 1844. Type species. *Lunania uniflora* Raf., nom. superfl. [≡ *H. limosa* (Sw.) Willd.].

≡ *Triexastima* Raf., Fl. Tellur. 4: 121. 1836 [1838]. Type species. *Triexastima uniflora* (Raf.) Raf., nom. superfl. [≡ *H. limosa* (Sw.) Willd.].

≡ *Phrynium* Loefl. ex Kuntze, Revis. Gen. Pl. 3(3): 318. 1898, nom. illeg., non *Phrynium* Willd., Sp. Pl. Editio quarta 1: 1, 17. 1797. Type species (designated here). *Phrynium limosum* (Sw.) Kuntze [≡ *H. limosa* (Sw.) Willd.].

= *Scholleropsis* H.Perrier, Notul. Syst. (Paris) 5: 158. 1936. Type species. *Scholleropsis lutea* H.Perrier [≡ *H. lutea* (H.Perrier) M.Pell.].

**Included species.** *Heteranthera limosa* (Sw.) Willd., *H. lutea* (H.Perrier) M.Pell., and *H. rotundifolia* (Kunth) Griseb.

2.3.2.b.ii. ***Heteranthera* subg. *Hydrothrix*** (Hook. f.) M.Pell. & C.N.Horn, **comb. & stat. nov.** ≡ *Hydrothrix* Hook. f., Ann. Bot. (Oxford) 1: 89. 1887. Type species. *Hydrothrix gardneri* Hook. f. [≡ *Heteranthera gardneri* (Hook. f.) M.Pell.].

urn:lsid:ipni.org:names:77379619-1 (https://www.ipni.org/n/77379619-1)

Figure 14I

≡ *Hookerina* Kuntze, Revis. Gen. Pl. 2: 718. 1891, nom. superfl. Type species. *Hookerina gardneri* (Hook. f.) Kuntze [≡ *H. gardneri* (Hook. f.) M.Pell.].

**Included species.** *Heteranthera gardneri* (Hook. f.) M.Pell.

2.3.2.b.iii. ***Heteranthera* subg. *Zosterella*** (Small) Rosatti, J. Arnold Arbor. 68(1): 59. 1987 ≡ *Zosterella Small*, Fl. Lancaster Co.: 68. 1913. Type species. *Zosterella dubia* (Jacq.) Small [≡ *Heteranthera dubia* (Jacq.) MacMill.].

Figure 14K–M

= *Heteranthera* sect. *Schollera* Solms. in A.L.P.P. de Candolle & A.C.P. de Candolle, Monogr. Phan. 4: 517. 1883 ≡ *Schollera* Schreb., Gen. Pl. 785. 1791, nom. illeg., non *Schollera* Roth, Tent. Fl. Germ. 1: 165, 170. 1788. Type species (designated here). *Schollera graminea* (Michx.) Willd. ex A.Gray [= *H. dubia* (Jacq.) MacMill.].

= *Eurystemon* Alexander, N. Amer. Fl. 19: 55. 1937. Type species. *Eurystemon mexicanum* (S.Watson) Alexander (≡ *H. mexicana* S.Watson).

**Included species.** *Heteranthera dubia* (Jacq.) MacMill., *H. hydrocleifolia* Griseb., *H. mexicana* S.Watson, *H. oblongifolia* Mart. ex Schult. & Schult. f., *H. osteniana* Herter, *H. seubertiana* Solms, *H. yucatana* Carnevali et al., and *H. zosterifolia* Mart.

2.3.2.b.iv. ***Heteranthera*** Ruiz & Pav. **subg. *Heteranthera***. Type species. *Heteranthera reniformis* Ruiz & Pav.

Figure 14N,O

≡ *Leptanthus* subg. *Heteranthera* (Ruiz & Pav.) Pers., Syn. Pl. 1: 56. 1805. Type species. *Leptanthus peruvianus* Pers., nom. superfl. (≡ *Heteranthera reniformis* Ruiz & Pav.).

≡ *Heterandra* P.Beauv., Trans. Amer. Philos. Soc. 4: 175. 1799, nom. superfl. Type species. *Heterandra reniformis* (Ruiz & Pav.) P.Beauv. (≡ *Heteranthera reniformis* Ruiz & Pav.).

= *Buchosia* Vell., Fl. Flumin.: 33. 1829. Type species. *Buchosia aquatica* Vell. (= *Heteranthera reniformis* Ruiz & Pav.).

**Included species.** *Heteranthera callifolia* Rchb. ex Kunth, *H. catharinensis* C.N.Horn & M.Pell., *H. longirachilla* D.J.Sousa & Giul., *H. missouriensis* C.N.Horn, *H. multiflora* (Griseb.) C.N.Horn, *H. pauciflora* C.N.Horn, *H. peduncularis* Benth., *H. pumila* M.Pell. & C.N.Horn, *H. reniformis* Ruiz & Pav., and *H. spicata* C.Presl.

#### 2.3.3. Haemodoraceae

In Haemodoraceae, some updates are necessary to recognise monophyletic and morphologically diagnosable genera, in addition to formalising some hybrids and elevating taxa to the species rank.

2.3.3.a. ***Anigozanthos*** Labill., Voy. Rech. Pérouse 1: 410. 1800. Type species. *Anigozanthos rufus* Labill.

Figure 15B–D

**Distribution.** *Anigozanthos* is endemic to Western Australia.

**Ecology.** *Anigozanthos* is found growing in dry or seasonally wet sand.

**Comments.** *Anigozanthos* would greatly benefit from a modern taxonomic revision. Furthermore, based on available molecular and morphological data, all accepted infraspecific taxa should be recognised at the specific rank.

**Infrageneric classification.** The current infrageneric classification for *Anigozanthos* [63] is not supported by either the present study or previous molecular-based phylogenies [39,65,66,67]. However, because the molecular dataset does not greatly agree with the molecular topologies recovered so far, I refrain from proposing a full updated classification, and only propose the recognition of *Macropidia* as a subgenus.

**Accepted taxa and new combinations.** A total of 23 species, plus three formally recognised natural hybrids, including the new name and combinations below:

2.3.3.a.i. ***Anigozanthos* subg. *Macropidia*** (J.Drumm. ex Harv.) M.Pell., **comb. & stat. nov.** ≡ *Macropidia* J.Drumm. ex Harv., Hooker’s J. Bot. Kew Gard. Misc. 7: 57. 1855 ≡ *Anigozanthos* sect. *Macropidia* (J.Drumm. ex Harv.) Geerinck, Bull. Jard. Bot. Natl. Belg. 39: 63. 1969. Type species. *Macropidia fumosa* J.Drumm. ex Harv., nom. superfl. (≡ *A. fuliginosus* Hook.).

urn:lsid:ipni.org:names:77379621-1 (https://www.ipni.org/n/77379621-1)

Figure 15B

**Included species.** *Anigozanthos fuliginosus* Hook.

2.3.3.a.ii. ***Anigozanthos*** Labill. **subg. *Anigozanthos***, Voy. Rech. Pérouse 1: 410. 1800. Type species. *Anigozanthos rufus* Labill.

Figure 15C,D

**Included species.** The remaining 22 species and the three natural hybrids.

2.3.3.a.ii(a) ***Anigozanthos chrysanthus*** (Hopper) M.Pell., **stat. nov.** ≡ *Anigozanthos humilis* subsp. *chrysanthus* Hopper, Fl. Australia 45: 456. 1987.

urn:lsid:ipni.org:names:77379622-1 (https://www.ipni.org/n/77379622-1)

2.3.3.a.ii(b) ***Anigozanthos* × *angustifolius*** (Lindl.) M.Pell., **stat. nov.** ≡ *Anigozanthos manglesii* var. × *angustifolius* Lindl., Bot. Reg. 23: t. 2012. 1837.

urn:lsid:ipni.org:names:77379623-1 (https://www.ipni.org/n/77379623-1)

**Parentage.** *Anigozanthos manglesii* D.Don. × *A. viridis* Endl.

2.3.3.a.ii(c) ***Anigozanthos* × *virescens*** (Ostenf.) M.Pell., **stat. nov.** ≡ *Anigozanthos manglesii* var. × *virescens* Ostenf., Biol. Meddel. Kongel. Danske Vidensk. Selsk. 3(2): 35 (1921).

urn:lsid:ipni.org:names:77379625-1 (https://www.ipni.org/n/77379625-1)

**Parentage.** *Anigozanthos manglesii* D.Don. × ? *A. metallicus* (Hopper) M.Pell.

2.3.3.a.ii(d) ***Anigozanthos exstans*** (Hopper) M.Pell., **stat. nov.** ≡ *Anigozanthos bicolor* subsp. *exstans* Hopper, Fl. Australia 45: 455. 1987.

urn:lsid:ipni.org:names:77379626-1 (https://www.ipni.org/n/77379626-1)

2.3.3.a.ii(e) ***Anigozanthos grandis*** (Hopper) M.Pell., **stat. nov.** ≡ *Anigozanthos humilis* subsp. *grandis* Hopper in Hopper et al., Nuytsia 36: 188. 2025.

urn:lsid:ipni.org:names:77379627-1 (https://www.ipni.org/n/77379627-1)

2.3.3.a.ii(f) ***Anigozanthos* × *hopperii*** M.Pell., **noth. sp. nov.** Type. AUSTRALIA. Western Australia: Old Crossman River Bridge, Albany Highway, fl., 22 Oct. 1976, *S.D. Hopper 754* (holotype: PERTH no. 01962043!).

urn:lsid:ipni.org:names:77379629-1 (https://www.ipni.org/n/77379629-1)

**Parentage.** *Anigozanthos exstans* (Hopper) M.Pell. × *A. decrescens* (Hopper) Hopper & R.J.Sm.

**Diagnosis.** Hybrid intermediate between its putative parents. Perianth 48–64 mm long, constricted above the ovary for 9.3–17.2 mm, perianth when flattened 3.3–6.3 mm wide at the narrowest point in the upper half. Anthers 2.7–4.3 mm long.

**Notes.** For further information on morphology, distribution, ecology, reproduction, and affinity, see Hopper et al. [68].

2.3.3.a.ii(g) ***Anigozanthos metallicus*** (Hopper) M.Pell., **stat. nov.** ≡ *Anigozanthos viridis* subsp. *metallicus* Hopper in Hopper et al., Nuytsia 36: 193. 2025.

urn:lsid:ipni.org:names:77379630-1 (https://www.ipni.org/n/77379630-1)

2.3.3.a.ii(h) ***Anigozanthos minor*** (Benth.) M.Pell., **stat. nov.** ≡ *Anigozanthos bicolor* var. *minor* Benth., Fl. Austral. 6: 446. 1873 ≡ *Anigozanthos bicolor* subsp. *minor* (Benth.) Hopper, Fl. Australia 45: 455. 1987.

urn:lsid:ipni.org:names:77379631-1 (https://www.ipni.org/n/77379631-1)

2.3.3.a.ii(i) ***Anigozanthos quadrans*** (Hopper) M.Pell., **stat. nov.** ≡ *Anigozanthos manglesii* subsp. *quadrans* Hopper, Fl. Australia 45: 456. 1987.

urn:lsid:ipni.org:names:77379632-1 (https://www.ipni.org/n/77379632-1)

2.3.3.a.ii(j) ***Anigozanthos sophrosyne*** (Hopper) M.Pell., **stat. nov.** ≡ *Anigozanthos viridis* subsp. *sophrosyne* Hopper in Hopper et al., Nuytsia 36: 196. 2025.

urn:lsid:ipni.org:names:77379634-1 (https://www.ipni.org/n/77379634-1)

2.3.3.a.ii(k) ***Anigozanthos terraspectans*** (Hopper) M.Pell., **stat. nov.** ≡ *Anigozanthos viridis* subsp. *terraspectans* Hopper, Fl. Australia 45: 456. 1987.

urn:lsid:ipni.org:names:77379635-1 (https://www.ipni.org/n/77379635-1)

2.3.3.b. ***Phlebocarya*** R.Br., Prodr.: 301. 1810. Type species. *Phlebocarya ciliata* R.Br.

Figure 15E

**Distribution.** *Phlebocarya* is endemic to southern Western Australia.

**Ecology.** *Phlebocarya* grows in well-drained to swampy sandy soils, in heath and woodland.

**Accepted species and new combination.** A total of four species: *Phlebocarya ciliata* R.Br., *P. filifolia* (F.Muell.) Benth., *P. pilosissima* (F.Muell.) Benth., and *P. teretifolia* (T.D.Macfarl.) M.Pell.

2.3.2.b.i. ***Phlebocarya teretifolia*** (T.D.Macfarl.) M.Pell., **stat. nov.** ≡ *Phlebocarya pilosissima* subsp. *teretifolia* T.D.Macfarl., Fl. Australia 45: 465. 1987.

urn:lsid:ipni.org:names:77379636-1 (https://www.ipni.org/n/77379636-1)

2.3.3.c. ***Conostylis*** R.Br., Prodr.: 300. 1810. Type species. *Conostylis aculeata* R.Br.

Figure 15F–I

**Distribution.** *Conostylis* is endemic to Western Australia.

**Ecology.** *Conostylis* is found growing in dry or seasonally wet sand.

**Comments.** *Conostylis* needs a taxonomic revision focusing on poorly explored characters in the genus. As evidenced by our analyses, anatomical characters are promising and should be further explored.

**Infrageneric classification.** Hopper et al. [68] updated the infrageneric classification of *Conostylis*, based on molecular data [65], to ensure the recognition of monophyletic taxa.

**Accepted taxa and new combinations.** Molecular and morphological data do not support the monophyly of most species that recognise infraspecific taxa, as they do not coalesce in either molecular or morphological phylogenies ([39,44,65,66,67], this study). With the necessary changes, *Conostylis* has ca. 70 species. Additionally, I reduce *Blancoa* to a subgenus of *Conostylis* and provide the needed updates for the classification proposed by Hopper et al. [68].

2.3.3.c.i. ***Conostylis* subg. *Blancoa*** (Lindl.) M.Pell., **comb. & stat. nov.** ≡ *Blancoa* Lindl., Edwards’s Bot. Reg.: 45. 1840 ≡ *Conostylis* sect. *Blancoa* (Lindl.) Kuntze in von Post & Kuntze, Lex. Gen. Phan.: 1903. Type species. *Blancoa canescens* Lindl. [≡ *C. canescens* (Lindl.) F.Muell.].

urn:lsid:ipni.org:names:77379637-1 (https://www.ipni.org/n/77379637-1)

Figure 15F

≡ *Styloconus* Baill., Hist. Pl. 13: 75. 1894, nom. superfl. Type species. *Blancoa canescens* Lindl. [≡ *C. canescens* (Lindl.) F.Muell.].

**Included species.** *Conostylis canescens* (Lindl.) F.Muell.

2.3.3.c.ii. ***Conostylis* subg. *Appendicula*** (Geerinck) Hopper in Hopper et al., Nuytsia 36: 201. 2025 ≡ *Conostylis* sect. *Appendicula* Geerinck, Bull. Jard. Bot. Etat. 39: 64. 1969. Type species. *Conostylis aurea* Lindl.

**Included species.** *Conostylis angustifolia* Hopper, *C. aurea* Lindl., *C. hiemalis* Hopper, *C. resinosa* Hopper, *C. seminuda* Hopper, and *C. tomentosa* Hopper.

2.3.3.c.iii. ***Conostylis*** R.Br. **subg. *Conostylis***, Prodr.: 300. 1810. Type species. *Conostylis aculeata* R.Br.

**Included species.** See under *C.* sect. *Terraflora* and *C.* sect. *Conostylis*.

2.3.3.c.iii(a) ***Conostylis* sect. *Terraflora*** Hopper in Hopper et al., Nuytsia 36: 202. 2025. Type species. *Conostylis serrulata* R.Br.

**Included species.** *Conostylis glabra* Hopper, *C. juncea* Endl., *C. laxiflora* Benth., *C. magna* Hopper, and *C. serrulata* R.Br.

2.3.3.c.iii(b) ***Conostylis*** R.Br. **sect. *Conostylis***, Prodr.: 300. 1810. Type species. *Conostylis aculeata* R.Br.

**Included species.** *Conostylis aculeata* R.Br., *C. bolghinup* (Hopper) M.Pell., *C. breviflora* (Hopper) M.Pell., *C. bromelioides* Endl., *C. calcicola* (Hopper) M.Pell., *C. candicans* Endl., *C. cygnorum* (Hopper) M.Pell., *C. echinissima* (Hopper) M.Pell., *C. euryrhipis* (Hopper) M.Pell., *C. festucacea* Endl., *C. filifolia* F.Muell., *C. flavifolia* (Hopper) M.Pell., *C. gracilis* (Hopper) M.Pell., *C. hickmaniae* (Hopper) M.Pell., *C. longissima* (Hopper) M.Pell., *C. misera* Endl., *C. pauciflora* Hopper, *C. procumbens* (Hopper) M.Pell., *C. prolifera* Benth., *C. rhipidion* (J.W.Green) M.Pell., *C. robusta* Diels, *C. seorsiflora* F.Muell., *C. septentrionora* (Hopper) M.Pell., *C. stylidoides* F.Muell., and *C. trichophylla* (Hopper) M.Pell.

2.3.3.c.iii(b1) ***Conostylis bolghinup*** (Hopper) M.Pell., **stat. nov.** ≡ *Conostylis aculeata* subsp. *bolghinup* Hopper in Hopper et al., Nuytsia 36: 208. 2025.

urn:lsid:ipni.org:names:77379638-1 (https://www.ipni.org/n/77379638-1)

2.3.3.c.iii(b2) ***Conostylis breviflora*** (Hopper) M.Pell., **stat. nov.** ≡ *Conostylis aculeata* subsp. *breviflora* Hopper, Nuytsia 2: 261. 1978.

urn:lsid:ipni.org:names:77379639-1 (https://www.ipni.org/n/77379639-1)

2.3.3.c.iii(b3) ***Conostylis calcicola*** (Hopper) M.Pell., **stat. nov.** ≡ *Conostylis candicans* subsp. *calcicola* Hopper, Fl. Australia 45: 459. 1987.

urn:lsid:ipni.org:names:77379640-1 (https://www.ipni.org/n/77379640-1)

2.3.3.c.iii(b4) ***Conostylis cygnorum*** (Hopper) M.Pell., **stat. nov.** ≡ *Conostylis aculeata* subsp. *cygnorum* Hopper, Fl. Australia 45: 457. 1987.

urn:lsid:ipni.org:names:77379641-1 (https://www.ipni.org/n/77379641-1)

2.3.3.c.iii(b5) ***Conostylis echinissima*** (Hopper) M.Pell., **stat. nov.** ≡ *Conostylis aculeata* subsp. *echinissima* Hopper, Fl. Australia 45: 458. 1987.

urn:lsid:ipni.org:names:77379642-1 (https://www.ipni.org/n/77379642-1)

2.3.3.c.iii(b6) ***Conostylis euryrhipis*** (Hopper) M.Pell., **stat. nov.** ≡ *Conostylis pauciflora* subsp. *euryrhipis* Hopper, Fl. Australia 45: 461. 1987.

urn:lsid:ipni.org:names:77379643-1 (https://www.ipni.org/n/77379643-1)

2.3.3.c.iii(b7) ***Conostylis flavifolia*** (Hopper) M.Pell., **stat. nov.** ≡ *Conostylis candicans* subsp. *flavifolia* Hopper, Fl. Australia 45: 459. 1987.

urn:lsid:ipni.org:names:77379644-1 (https://www.ipni.org/n/77379644-1)

2.3.3.c.iii(b8) ***Conostylis gracilis*** (Hopper) M.Pell., **stat. nov.** ≡ *Conostylis aculeata* subsp. *gracilis* Hopper, Fl. Australia 45: 458. 1987.

urn:lsid:ipni.org:names:77379645-1 (https://www.ipni.org/n/77379645-1)

2.3.3.c.iii(b9) ***Conostylis hickmaniae*** (Hopper) M.Pell., **stat. nov.** ≡ *Conostylis robusta* subsp. *hickmaniae* Hopper Hopper et al., Nuytsia 36: 214. 2025.

urn:lsid:ipni.org:names:77379646-1 (https://www.ipni.org/n/77379646-1)

2.3.3.c.iii(b10) ***Conostylis longissima*** (Hopper) M.Pell., **stat. nov.** ≡ *Conostylis seorsiflora* subsp. *longissima* Hopper, Fl. Australia 45: 462. 1987.

urn:lsid:ipni.org:names:77379647-1 (https://www.ipni.org/n/77379647-1)

2.3.3.c.iii(b11) ***Conostylis procumbens*** (Hopper) M.Pell., **stat. nov.** ≡ *Conostylis candicans* subsp. *procumbens* Hopper, Fl. Australia 45: 459. 1987.

urn:lsid:ipni.org:names:77379648-1 (https://www.ipni.org/n/77379648-1)

2.3.3.c.iii(b12) ***Conostylis rhipidion*** (J.W.Green) M.Pell., **stat. nov.** ≡ *Conostylis aculeata* subsp. *rhipidion* J.W.Green, Proc. Linn. Soc. New South Wales 85: 348. 1961.

urn:lsid:ipni.org:names:77379649-1 (https://www.ipni.org/n/77379649-1)

2.3.3.c.iii(b13) ***Conostylis septentrionora*** (Hopper) M.Pell., **stat. nov.** ≡ *Conostylis aculeata* subsp. *septentrionora* Hopper, Fl. Australia 45: 458. 1987.

urn:lsid:ipni.org:names:77379650-1 (https://www.ipni.org/n/77379650-1)

2.3.3.c.iii(b14) ***Conostylis trichophylla*** (Hopper) M.Pell., **stat. nov.** ≡ *Conostylis seorsiflora* subsp. *trichophylla* Hopper, Fl. Australia 45: 462. 1987.

urn:lsid:ipni.org:names:77379651-1 (https://www.ipni.org/n/77379651-1)

2.3.3.c.iv. ***Conostylis* subg. *Brachycaulon*** (Benth.) Hopper, Fl. Australia 45: 457. 1987 ≡ *Conostylis* sect. *Brachycaulon* Benth., Fl. Austral. 6:428, 430. 1873. Type species. *Conostylis breviscapa* R.Br.

**Included species.** *Conostylis breviscapa* R.Br.

2.3.3.c.v. ***Conostylis* subg. *Bicolorata*** Hopper, Fl. Australia 45: 456. 1987. Type species. *Conostylis vaginata* Endl.

Figure 15I

**Included species.** *Conostylis vaginata* Endl.

2.3.3.c.vi. ***Conostylis* subg. *Divaricata*** (Hopper) Hopper in Hopper et al., Nuytsia 36: 202. 2025 ≡ *Conostylis* sect. *Divaricata* Hopper, Fl. Australia 45: 457. 1987. Type species. *Conostylis phathyrantha* Diels.

**Included species.** *Conostylis phathyrantha* Diels.

2.3.3.c.vii. ***Conostylis* subg. *Pendula*** Hopper, Fl. Australia 45: 457. 1987. Type: *Conostylis setigera* R.Br.

Figure 15G,H

≡ *Conostylis* sect. *Catospora* Benth., Fl. Austral. 6: 428. 1873, **syn. nov.** Type species. *Conostylis setigera* R.Br.

= *Androstemma* Lindl., Sketch Veg. Swan R.: xlvi. 1840 ≡ *Conostylis* subg. *Androstemma* (Lindl.) Hopper, Fl. Australia 45: 456. 1987 ≡ *Conostylis* subg. *Androstemma* (Lindl.) Hopper Fl. Australia 45: 456. 1987. Type species. *Conostylis androstemma* F.Muell.

= *Conostylis* subg. *Greenia* (Geerinck) Hopper, Fl. Australia 45: 457. 1987 ≡ *Conostylis* sect. *Greenia* Geer., Bull. Jard. Bot. État. 39: 65. 1969. Type: *Conostylis bealiana* F.Muell.

**Included species.** *Conostylis absens* (Hopper) M.Pell., *C. albescens* Hopper, *C. androstemma* F.Muell., *C. argentea* (J.W.Green) Hopper, *C. bealiana* F.Muell., *C. bungalbin* Hopper, *C. canteriata* Hopper, *C. caricina* Lindl., *C. crassinerva* J.W.Green, *C. dasys* (Hopper) Hopper, *C. deplexa* J.W.Green, *C. dielsii* W.Fitzg., *C. drummondii* Benth., *C. elachys* (Hopper) M.Pell., *C. latens* Hopper, *C. lepidospermoides* Hopper, *C. micrantha* Hopper, *C. neocymosa* Hopper, *C. petrophiloides* F.Muell. ex Benth., *C. planescens* (Hopper) M.Pell., *C. pusilla* Endl., *C. rogeri* Hopper, *C. setigera* R.Br., *C. setosa* Lindl., *C. teres* (Hopper) M.Pell., *C. teretifolia* J.W.Green, *C. teretiuscula* F.Muell., *C. villosa* Benth., and *C. wonganensis* Hopper.

2.3.3.c.vii(a) ***Conostylis absens*** (Hopper) M.Pell., **stat. nov.** ≡ *Conostylis crassinerva* subsp. *absens* Hopper, Fl. Australia 45: 460. 1987.

urn:lsid:ipni.org:names:77379652-1 (https://www.ipni.org/n/77379652-1)

2.3.3.c.vii(b) ***Conostylis elachys*** (Hopper) M.Pell., **stat. nov.** ≡ *Conostylis caricina* subsp. *elachys* Hopper, Fl. Australia 45: 460. 1987.

urn:lsid:ipni.org:names:77379653-1 (https://www.ipni.org/n/77379653-1)

2.3.3.c.vii(c) ***Conostylis planescens*** (Hopper) M.Pell., **stat. nov.** ≡ *Conostylis teretifolia* subsp. *planescens* Hopper, Fl. Australia 45: 463. 1987.

urn:lsid:ipni.org:names:77379654-1 (https://www.ipni.org/n/77379654-1)

2.3.3.c.vii(d) ***Conostylis teres*** (Hopper) M.Pell., **stat. nov.** ≡ *Conostylis dielsii* subsp. *teres* Hopper, Fl. Australia 45: 460. 1987.

urn:lsid:ipni.org:names:77379655-1 (https://www.ipni.org/n/77379655-1)

2.3.3.d. ***Paradilatris*** (Hopper ex J.C.Manning) Hopper in Hopper et al., Nuytsia 36: 169. 2025 ≡ *Dilatris* subg. *Paradilatris* Hopper ex J.C.Manning, S. African J. Bot. 113: 104. 2017. Type species. *Paradilatris viscosa* (L. f.) Hopper.

Figure 15K

**Distribution.** Endemic to the Western Cape Region, South Africa.

**Ecology.** Restricted to seasonally flooded areas, including montane seeps, marshes, and seasonally wet sandy soils.

**Accepted species and new combination.** A total of two species: *Paradilatris viscosa* (L. f.) Hopper and *P. paniculata* (L. f.) M.Pell.:

2.3.3.d.i. ***Paradilatris paniculata*** (L. f.) M.Pell., **comb. nov.** ≡ *Dilatris paniculata* L. f., Suppl. Pl.: 101. 1782.

urn:lsid:ipni.org:names:77379656-1 (https://www.ipni.org/n/77379656-1)

2.3.3.e. ***Haemodorum*** Sm., Trans. Linn. Soc. London 4: 213. 1798. Type. *Haemodorum corymbosum* Vahl.

Figure 15M,N

**Distribution.** Restricted to Australia, Tasmania, and Papua New Guinea.

**Ecology.** Species can be found growing in a myriad of open environments.

**Accepted species and new combination.** Ca. 40 species, including the new combination below:

2.3.3.e.i. ***Haemodorum gracilescens*** (Domin) M.Pell., **stat. nov.** ≡ *Haemodorum corymbosum* var. *gracilescens* Domin, Biblioth. Bot. 85: 527. 1915.

urn:lsid:ipni.org:names:77379657-1 (https://www.ipni.org/n/77379657-1)

**Note.** This name matches the morphologically distinct elements of *H. coccineum* R.Br. from the Northern Territory, northern Queensland and Papua New Guinea, with smaller and crimson to maroon flowers (vs. orange-red to red in *H. coccineum*), sepals shorter than the petals with obtuse apex (vs. subequal to equal, apex acute), petals erect (vs. reflexed at apex), anthers smaller and orange-red (vs. orange-yellow to yellowish-orange), connective minutely apiculate at apex (vs. truncate), style apex orange-red (vs. red to maroon), stigma apricot (vs. black), fruits burgundy to maroon, becoming dark brown when mature (vs. dark purple, atropurpureous to bluish-black when mature).

2.3.3.f. ***Wachendorfia*** Burm. ex L., Syst. Nat., ed. 10. 2: 864. 1759. Type species. *Wachendorfia paniculata* Burm.

Figure 15P,Q

**Distribution.** *Wachendorfia* is endemic to South Africa.

**Ecology.** *Wachendorfia* is found growing exclusively in seasonally wet areas in the Grassland and Fynbos biomes.

**Accepted species and new combination.** Ca. 10 species, with many still undescribed:

2.3.3.f.i. ***Wachendorfia* subg. *Barberetta*** (Harv.) M.Pell., **comb. & stat. nov.** ≡ *Barberetta* Harv., Gen. S. Afr. Fl. Pl. (ed. 2): 377. 1868. Type species. *Barberetta aurea* Harv. [≡ *W. aurea* (Harv.) M.Pell.].

urn:lsid:ipni.org:names:77379658-1 (https://www.ipni.org/n/77379658-1)

Figure 15P

**Included species.** *Wachendorfia aurea* (Harv.) M.Pell., plus an undescribed species.

2.3.3.f.i(a) ***Wachendorfia aurea*** (Harv.) M.Pell., **comb. nov.** ≡ *Barberetta aurea* Harv., Gen. S. Afr. Fl. Pl. (ed. 2): 377. 1868.

urn:lsid:ipni.org:names:77379659-1 (https://www.ipni.org/n/77379659-1)

2.3.3.f.ii. ***Wachendorfia*** Burm. ex L. **subg. *Wachendorfia***, Syst. Nat., ed. 10. 2: 864. 1759. Type species. *Wachendorfia paniculata* Burm.

Figure 15Q

**Included species.** *Wachendorfia brachyandra* W.F.Barker, *W. laxa* W.F.Barker ex Hopper, *W. multiflora* (Klatt) J.C.Manning & Goldblatt., *W. paniculata* L., and *W. thyrsiflora* L.

#### 2.3.4. Hanguanaceae

The taxonomic updates needed at this moment for Hanguanaceae (e.g., description of new species, standardisation of its morphological terminology, etc.) cannot be easily implemented here and, thus, will be implemented in several future studies.

#### 2.3.5. Commelinaceae

In Commelinaceae, four new subtribes, five new genera and several status changes are necessary and formalised below:

2.3.5.a. ***Aneilema*** R.Br., Prodr.: 270. 1810. Type species. *Aneilema biflorum* R.Br.

Figure 16O–Q

**Distribution.** Mainly Paleotropical and centred in Africa, but extending to Australasia, with a sole species reaching the Neotropics.

**Ecology.** Its species can be found growing from open and dry environments to shady and moist environments and forest understories.

**Comments.** With the exclusion of *Fadeniella brasiliensis* and the Australian species of *Pollia*, the reduction of *Rhopalephora* to the sectional rank, and the inclusion of *A. yunnanense*, *Aneilema* is finally rendered monophyletic.

**Accepted species and new combinations.** Ca. 80 species, including the new combination below:

2.3.5.a.i. ***Aneilema brevisepalum*** (Brenan) M.Pell., **stat. nov.** ≡ *Aneilema nyansense* var. *brevisepalum*, as “*brevisepala*”, Brenan, Kew Bull. 15: 214. 1961.

urn:lsid:ipni.org:names:77379669-1 (https://www.ipni.org/n/77379669-1)

2.3.5.a.ii. ***Aneilema gypsophilum*** (Faden) M.Pell., **stat. nov.** ≡ *Aneilema pusillum* subsp. *gypsophilum* Faden, Smithsonian Contr. Bot. 76: 88. 1991.

urn:lsid:ipni.org:names:77379670-1 (https://www.ipni.org/n/77379670-1)

2.3.5.a.iii. ***Aneilema keniense*** (Faden) M.Pell., **stat. nov.** ≡ *Aneilema indehiscens* subsp. *keniense* Faden, Smithsonian Contr. Bot. 76: 104. 1991.

urn:lsid:ipni.org:names:77379671-1 (https://www.ipni.org/n/77379671-1)

2.3.5.a.iv. ***Aneilema leonense*** (J.K.Morton) M.Pell., **stat. nov.** ≡ *Aneilema beniniense* subsp. *leonense* J.K.Morton, J. Linn. Soc., Bot. 60: 169. 1967.

urn:lsid:ipni.org:names:77379672-1 (https://www.ipni.org/n/77379672-1)

2.3.5.a.v. ***Aneilema lilacinum*** (Faden) M.Pell., **stat. nov.** ≡ *Aneilema indehiscens* subsp. *lilacinum* Faden, Bothalia 15(1–2): 97. 1984.

urn:lsid:ipni.org:names:77379673-1 (https://www.ipni.org/n/77379673-1)

2.3.5.a.vi. ***Aneilema longiaxis*** (Faden) M.Pell., **stat. nov.** ≡ *Aneilema hockii* subsp. *longiaxis* Faden, Fl. Trop. E. Africa, Commelin. 8. 2012.

urn:lsid:ipni.org:names:77379675-1 (https://www.ipni.org/n/77379675-1)

2.3.5.a.vii. ***Aneilema luteum*** (C.B.Clarke) M.Pell., **stat. nov.** ≡ *Aneilema pedunculosum* var. *luteum* C.B.Clarke in A.L.P.P. de Candolle & A.C.P. de Candolle, Monogr. Phan. 3: 228. 1881 ≡ *Aneilema angolense* var. *luteum* (C.B.Clarke) Faden in Figueiredo & Smith, Strelitzia 22: 176. 2008.

urn:lsid:ipni.org:names:77379676-1 (https://www.ipni.org/n/77379676-1)

2.3.5.a.viii. ***Aneilema mossambicense*** (Faden) M.Pell., **stat. nov.** ≡ *Aneilema dregeanum* subsp. *mossambicense* Faden, Bothalia 15(1–2): 90. 1984.

urn:lsid:ipni.org:names:77379674-1 (https://www.ipni.org/n/77379674-1)

2.3.5.a.ix. ***Aneilema pallidiciliatum*** (J.K.Morton) M.Pell., **stat. nov.** ≡ *Aneilema setiferum* subsp. *pallidiciliatum* J.K.Morton, J. Linn. Soc., Bot. 59: 451. 1966.

urn:lsid:ipni.org:names:77379677-1 (https://www.ipni.org/n/77379677-1)

2.3.5.a.x. ***Aneilema pallidiflorum*** (Faden) M.Pell., **stat. nov.** ≡ *Aneilema petersii* subsp. *pallidiflorum* Faden, Smithsonian Contr. Bot. 76: 97. 1991.

urn:lsid:ipni.org:names:77379678-1 (https://www.ipni.org/n/77379678-1)

2.3.5.a.xi. ***Aneilema pauciflorum*** (J.K.Morton) M.Pell., **stat. nov.** ≡ *Aneilema paludosum* subsp. *pauciflorum* J.K.Morton, J. Linn. Soc., Bot. 59: 447. 1966.

urn:lsid:ipni.org:names:77379679-1 (https://www.ipni.org/n/77379679-1)

2.3.5.a.xii. ***Aneilema pilosum*** (Brenan) M.Pell., **stat. nov.** ≡ *Aneilema lanceolatum* var. *pilosum* Brenan, Kew Bull. 15: 219. 1961.

urn:lsid:ipni.org:names:77379680-1 (https://www.ipni.org/n/77379680-1)

2.3.5.a.xiii. ***Aneilema pseudolanceolatum*** (J.K.Morton) M.Pell., **stat. nov.** ≡ *Aneilema paludosum* subsp. *pseudolanceolatum* J.K.Morton, J. Linn. Soc., Bot. 59: 448. 1966.

urn:lsid:ipni.org:names:77379681-1 (https://www.ipni.org/n/77379681-1)

2.3.5.a.xiv. ***Aneilema rhizomatosum*** (Faden) M.Pell., **stat. nov.** ≡ *Aneilema hockii* var. *rhizomatosum* Faden, Fl. Trop. E. Africa, Commelin. 80. 2012.

urn:lsid:ipni.org:names:77379682-1 (https://www.ipni.org/n/77379682-1)

2.3.5.a.xv. ***Aneilema sessiliflorum*** (Benth.) M.Pell., **stat. nov.** ≡ *Aneilema beniniense* var. *sessiliflorum* Benth., Niger Fl. 549. 1849 ≡ *Aneilema beniniense* subsp. *sessiliflorum* (Benth.) J.K.Morton, J. Linn. Soc., Bot. 59: 466. 1966.

urn:lsid:ipni.org:names:77379683-1 (https://www.ipni.org/n/77379683-1)

2.3.5.a.xvi. ***Aneilema thulinii*** (Faden) M.Pell., **stat. nov.** ≡ *Aneilema pusillum* subsp. *thulinii* Faden Smithsonian Contr. Bot. 76: 89. 1991.

urn:lsid:ipni.org:names:77379684-1 (https://www.ipni.org/n/77379684-1)

2.3.5.a.xvii. ***Aneilema variabile*** (Faden) M.Pell., **stat. nov.** ≡ *Aneilema pusillum* subsp. *variabile* Faden Smithsonian Contr. Bot. 76: 88. 1991.

urn:lsid:ipni.org:names:77379685-1 (https://www.ipni.org/n/77379685-1)

2.3.5.a.xviii. ***Aneilema yunnanense*** (D.Y.Hong) M.Pell., **comb. nov.** ≡ *Floscopa yunnanensis* D.Y.Hong, Acta Phytotax. Sin. 12(4): 464–465, pl. 93, f. 1. 1974.

urn:lsid:ipni.org:names:77379686-1 (https://www.ipni.org/n/77379686-1)

2.3.5.b. ***Commelina*** Plum. ex L., Sp. Pl. 1: 40. 1753. Type species. *Commelina communis* L.

Figure 16R–T

**Distribution.** Cosmopolitan, but centred in tropical Africa.

**Ecology.** Found growing in a myriad of environments, especially dry, but seldom in forests.

**Figure 16:**
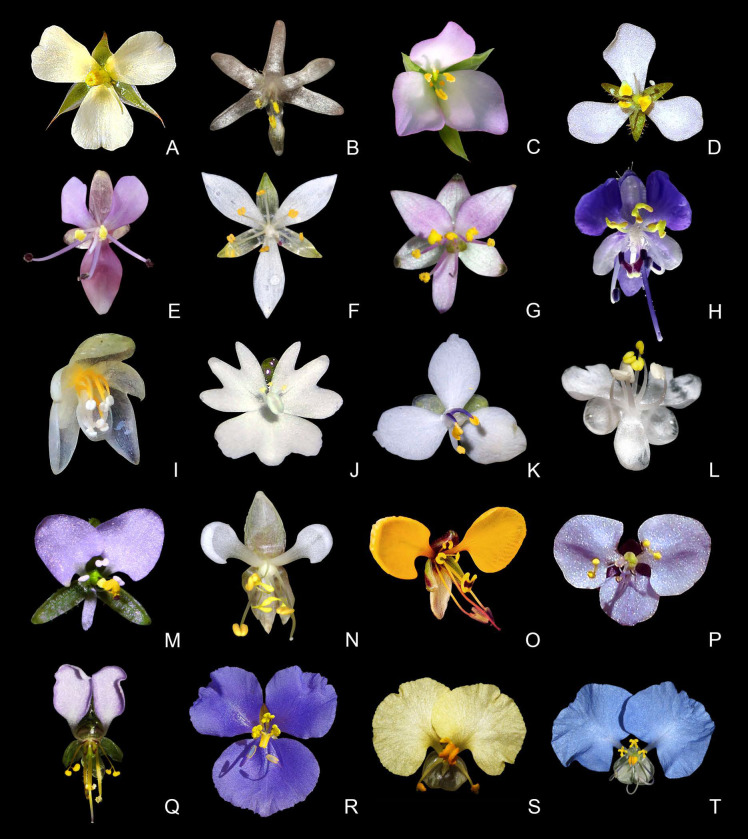
Floral morphology of Commelinaceae. (**A**), *Cartonema trigonospermum* C.B.Clarke. (**B**), *Palisota bracteosa* C.B.Clarke; (**C**), *Murdannia sepalosa* (C.B.Clarke) C.K.Lee et al.; (**D**), *M. engelsii* M.Pell. & Faden. (**E**), *Saxofloscopa africana* (Faden) M.Pell.; (**F**), *Stanfieldiella imperforata* (C.B.Clarke) Brenan; (**G**), *Floscopa hirsuta* (Kunth) Hassk. (**H**), *Tricarpelema chinense* D.Y.Hong; (**I**), *Buforrestia* sp.; (**J**), *Pseudoparis cauliflora* H.Perrier. (**K**), *Dictyospermum montanum* Wight; (**L**), *Pollia secundiflora* (Blume) Bakh. f.; (**M**), *Fadeniella brasiliensis* (C.B.Clarke) M.Pell.; (**N**), *Polyspatha paniculata* Benth.; (**O**), *Aneilema johnstonii* K.Schum.; (**P**), *A. protensum* (Wall. *ex* Wight) Thwaites; (**Q**), *A. beniniense* (P.Beauv.) Kunth; (**R**), *Commelina agrostophylla* F.Muell.; (**S**), *C. catharinensis* Hassemer et al.; (**T**), *C. erecta* L. A by B. Stuckey, B, G, Q & S–T by M.O.O. Pellegrini, C & E by L.E. Brothers, D by M.R. Engels, F & N by E. Bidault, H by R. Gogoi and S. Borah, I by L.A. Teixeira, J by C. Davidson, K by A.N. Sharma, L by R. Schneider, M by C.N. Fraga, O by B. Wursten, P by P. Awale, and R by R. Cumming.

**Comments.** *Tapheocarpa* was described to accommodate the peculiar *A. calandrinioides*, which clearly does not fit in *Aneilema* [29]. It was defined by its inflorescences reduced to a solitary flower, echinate, indehiscent, and geocarpic fruits. At the time of the description of the genus, its antherodes were misinterpreted as two-lobed. However, a careful dissection of flowers in herbarium specimens shows X-shaped antherodes, which can appear two-lobed when folded in half during pressing and drying of specimens. This character alone would be enough to safely place *Tapheocarpa* in *Commelina*. In light of the antherode morphology and phylogenetic placement, the inflorescence of *Tapheocarpa* can be easily reinterpreted as an extreme reduction of the Commelina-type inflorescence, which is already greatly reduced on its own merit. The inflorescences of typical *Commelina* already lack a developed main axis, cincinni bracts, with bracteoles generally so minute they are virtually invisible to the naked eye. Thus, the inflorescence of *Tapheocarpa* would only need to lose its basal bract, abort the upper cincinnus (which is commonly observed in several species of *Commelina*), and have the lower cincinnus reduced to become 1-flowered (also not uncommon in *Commelina*). Finally, inflorescences of *Commelina* are always leaf-opposed, even when they appear terminal (e.g., *C. erecta* L.). This can only be confirmed by dissecting the apex of the flowering branches or, if they posteriorly elongate, revealing such a pattern. Nonetheless, as it happens with several species of *Commelina* with apparently terminal inflorescences, that flowering branch does not continue its growth after flowering [69]. Based on morphological evidence, *C. calandrinioides* seems to be closely related to other aquatic Australian species with floating stems, linear and succulent leaves, reduced spathes, aborted upper cincinni, 1–few-flowered lower cincinni, and very broad and subequal petals (e.g., *C. agrostophylla* F.Muell.; Figure 16R).

**Accepted species and new combinations.** Ca. 250 species, including the new combinations below:

2.3.5.b.i. ***Commelina aggregata*** (Faden) M.Pell., **stat. nov.** ≡ *Commelina nigritana* subsp. *aggregata* Faden, Fl. Trop. E. Africa, Commelin. 234(–235). 2012.

urn:lsid:ipni.org:names:77379690-1 (https://www.ipni.org/n/77379690-1)

2.3.5.b.ii. ***Commelina amplexicaulis*** (Faden) M.Pell., **stat. nov.** ≡ *Commelina foliacea* subsp. *amplexicaulis* Faden, Novon 4(3): 232. 1994.

urn:lsid:ipni.org:names:77379695-1 (https://www.ipni.org/n/77379695-1)

2.3.5.b.iii. ***Commelina chacoensis*** (Slanis & Bulacio) M.Pell., **stat. nov.** ≡ *Commelina fasciculata* subsp. *chacoensis* Slanis & Bulacio, Darwiniana 45(1): 89 (88–91; fig. 1). 2007.

urn:lsid:ipni.org:names:77379696-1 (https://www.ipni.org/n/77379696-1)

2.3.5.b.iv. ***Commelina deamiana*** (Fernald) M.Pell., **stat. nov.** ≡ *Commelina erecta* var. *deamiana* Fernald, Rhodora 42: 440, tab. 631. 1940.

urn:lsid:ipni.org:names:77379697-1 (https://www.ipni.org/n/77379697-1)

2.3.5.b.v. ***Commelina gideonii*** (Nandikar) M.Pell., **stat. nov.** ≡ *Commelina attenuata* var. *gideonii* Nandikar, Commelin. India: 54. 2024.

urn:lsid:ipni.org:names:77379691-1 (https://www.ipni.org/n/77379691-1)

2.3.5.b.vi. ***Commelina indica*** (Nandikar) M.Pell., **stat. nov.** ≡ *Commelina kurzii* subsp. *indica* Nandikar, Commelin. India: 73. 2024.

urn:lsid:ipni.org:names:77379694-1 (https://www.ipni.org/n/77379694-1)

2.3.5.b.vii. ***Commelina jacobii*** C.E.C.Fisch., Bull. Misc. Inform. Kew 1928: 277. 1928.

= *Commelina alisagarensis* Kumar & Deodikar, Proc. Indian Acad. Sci., B 13(3): 168. 1941, **syn. nov.**

= *Commelina petersii* subsp. *geniculata* (C.B.Clarke) Nandikar, Commelin. India: 81. 2024 ≡ *Commelina persicariifolia* var. *geniculata* C.B.Clarke in A.L.P.P.de Candolle & A.C.P.de Candolle, Monogr. Phan. 3: 172. 1881, **syn. nov.**

= *Commelina petiolata* Abeyw., Ceylon J. Sci., Biol. Sci. 2: 82. 1959, **syn. nov.**

– *Commelina persicariifolia* Wight ex C.B.Clarke in A.L.P.P.de Candolle & A.C.P.de Candolle, Monogr. Phan. 3: 171 (1881), nom. illeg., **syn. nov.**

2.3.5.b.viii. ***Commelina kucharii*** (Faden) M.Pell., **stat. nov.** ≡ *Commelina polhillii* subsp. *kucharii* Faden, Fl. Trop. E. Africa, Commelin. 164 (166-167). 2012.

urn:lsid:ipni.org:names:77379689-1 (https://www.ipni.org/n/77379689-1)

2.3.5.b.ix. ***Commelina maritima*** (J.K.Morton) M.Pell., **stat. nov.** ≡ *Commelina gerrardii* subsp. *maritima* J.K.Morton, J. Linn. Soc., Bot. 55: 525. 1956 ≡ *Commelina erecta* subsp. *maritima* (J.K.Morton) J.K.Morton, J. Linn. Soc., Bot. 60: 184. 1967.

**Notes.** *Commelina erecta* L. is an exclusively Neotropical species, which all Paleotropical names conflated under its very broad sense, requiring reestablishment and formal recognition. *Commelina gerrardii* C.B.Clarke and *C. livingstonii* C.B.Clarke are also reestablished here for the remaining African plants previously treated under *C. erecta* slat.

urn:lsid:ipni.org:names:77379758-1 (https://www.ipni.org/n/77379758-1)

2.3.5.b.x. ***Commelina palkondensis*** (Sivaram., Yugandhar & L.J.Singh) M.Pell., **stat. nov.** ≡ *Commelina badamica* var. *palkondensis* Sivaram., Yugandhar & L.J.Singh, Nordic J. Bot. 2024(3)-e04152: 4. 2024.

urn:lsid:ipni.org:names:77379692-1 (https://www.ipni.org/n/77379692-1)

2.3.5.b.xi. ***Commelina rhizomifera*** (Faden) M.Pell., **stat. nov.** ≡ *Commelina bracteosa* subsp. *rhizomifera* Faden, Fl. Trop. E. Africa, Commelin. 215(–217). 2012.

urn:lsid:ipni.org:names:77379693-1 (https://www.ipni.org/n/77379693-1)

2.3.5.b.xii. ***Commelina thikaensis*** (Faden) M.Pell., **stat. nov.** ≡ *Commelina eckloniana* subsp. *thikaensis* Faden, Fl. Trop. E. Africa, Commelin. 184(–185). 2012.

urn:lsid:ipni.org:names:77379698-1 (https://www.ipni.org/n/77379698-1)

2.3.5.b.xiii. ***Commelina undulatifolia*** (Faden) M.Pell., **stat. nov.** ≡ *Commelina latifolia* var. *undulatifolia* Faden, Fl. Trop. E. Africa, Commelin. 181(–182). 2012.

urn:lsid:ipni.org:names:77379699-1 (https://www.ipni.org/n/77379699-1)

2.3.5.b.i. ***Commelina volomparasy*** M.Pell., **nom. nov.** ≡ *Commelina diffusa* subsp. *violacea* Faden, Taxon 52(4): 832. 2003.

urn:lsid:ipni.org:names:77379700-1 (https://www.ipni.org/n/77379700-1)

2.3.5.b.ii. ***Commelina zanzibarica*** (Faden) M.Pell., **stat. nov.** ≡ *Commelina africana* subsp. *zanzibarica* Faden, Fl. Trop. E. Africa, Commelin.: 148. 2012.

urn:lsid:ipni.org:names:77379757-1 (https://www.ipni.org/n/77379757-1)

2.3.5.c. ***Fadeniella*** M.Pell., **gen. nov.** Type species. *Fadeniella brasiliensis* (C.B.Clarke) M.Pell. (≡ *Aneilema brasiliense* C.B.Clarke).

urn:lsid:ipni.org:names:77379703-1 (https://www.ipni.org/n/77379703-1)

Figure 16M

**Description.** *Herbs* chamaephytes, base definite, solitary, perennial, terrestrial or rupicolous. *Roots* thin and fibrous. *Stolons* absent, rarely produced. *Stems* erect, unbranched to sparsely branched on the upper third, rooting at the basal nodes; internodes puberulous with hook hairs, the lower ones glabrescent at age. *Leaves* spirally alternate, congested at the apex of the stem, subpetiolate, becoming sessile towards the apex of the stem; ptyxis involute; sheaths puberulous with hook hairs, margins ciliate; blades flat, smaller towards the apex of the stems, membranous, sparsely to densely puberulous with hook hairs on both sides, abaxial side scabrid with prickle hairs near the margins, sometimes also with eglandular uniseriate hairs, base symmetrical, cuneate, margins scabrid with prickle hairs, apex acuminate; midvein conspicuous, adaxially impressed, abaxially very prominent, secondary veins inconspicuous. *Synflorescence* composed of a solitary main florescence or with 1–few coflorescences. *Main florescences (inflorescences)* terminal or axillary in the in the uppermost nodes, not perforating the leaf-sheaths; main florescence many-branched, pedunculate, lax thyrse; basal bract reduced to leaf-like; peduncle densely puberulous with hook hairs; peduncle bracts (sterile bracts) absent; main axis elongated, in zig-zag, puberulous with hook hairs; cincinni bracts persistent, flat, decreasing in size towards the apex of the main florescence, almost glabrous or puberulous with a mixture of hook hairs and uniseriate eglandular hairs; cincinni alternate, long-pedunculate, peduncles decreasing in length towards the apex, puberulous with hook hairs, axis elongated, ascending to erect, rarely patent, sinuate, puberulous with hook hairs; bracteoles cup-shaped, non-perfoliate, herbaceous, apex eglandular, persistent, puberulous with a mixture of hook hairs and uniseriate eglandular hairs. *Flowers* hermaphrodite or staminate (the staminate ones with a reduced or completely aborted gynoecium), zygomorphic, non-enantiostylous, chasmogamous, flat (not tubular); pedicels deflexed at pre-anthesis, geniculate at anthesis, erect at post-anthesis; pedicels stout, not gibbous at apex, slightly elongated and lignified at post-anthesis and in fruit; sepals 3, unequal, free, cucullate, membranous, dorsally not keeled, sparsely with a mixture of hook hairs and uniseriate eglandular hairs, margins hyaline, apex eglandular, slightly accrescent and persistent in fruit; petals 3, unequal, free, deliquescent, glabrous, medial one discolorous, light blue to pale lilac to lilac, paired petals clawed, held upwards to deflexed, claws glabrous, lighter than the limb, limb concave, the medial sessile, held downwards to strongly deflexed, white, limb linear, flat; staminodes 0–3, if present subequal, posterior, filaments glabrous, medial staminode sometimes with an unlobed antherode, lateral staminodes free from the stamens, antherodes bilobed, lobes sessile, lobes transversally ellipsoid, lilac; stamens 3, unequal, anterior, filaments free, glabrous, anthers versatile, dehiscence rimose, introrse, lateral filaments initially horizontal, straight but sharply recurved near apex, then arcuate-descending, anthers with an inconspicuous connective, medial filament initially arcuate-ascending, then arcuate-descending, anther with a conspicuous connective, of a different size, shape and color, its pollen also different in color; ovary sessile, glabrous, apex truncate, 3-locular, ovules uniseriate, dorsal locule reduced, 1-ovulate, ventral locules 2-ovulate, style elongate, gently arcuate-descending but sharply recurved at apex, then arcuate-descending, not spirally-coiled at post-anthesis, stigma capitate. *Capsules* loculicidal, 2-valved, sessile, smooth, glabrous, apex emarginate, valves slightly spreading, persistent, dorsal locule empty, ventral locules 2-seeded. *Seeds* monomorphic, exarillate, unappendaged, non-farinose, uniseriate, rectangular, not cleft towards the embryotega, ventrally flattened, testa rugose, tan, spotted with dark brown, especially on the bumps, with a very low, fine, irregular, colourless reticulum on the surface; hilum linear, prominent, raised in a shallow groove, slightly extended onto apical and basal surfaces; embryotega lateral.

**Etymology.** The generic name honours botanist, colleague and mentor, Dr Robert B. Faden, for his amazing contributions to the taxonomy, nomenclature and systematics of Commelinaceae, especially tribe Commelineae.

**Distribution.** *Fadeniella* is endemic to South America (in Brazil and Venezuela).

**Ecology.** *Fadeniella* is found growing in the understory of rainforests or seasonally dry forests.

**Notes.** *Fadeniella brasiliensis* has long been considered a species of uncertain generic placement, as well as of uncertain systematic affinity [29]. Due to the lack of obvious morphological differences from *Aneilema*, Faden [29] felt it was best to keep it in *Aneilema* and await further phylogenetic data. Kelly & Evans [70] recovered *F. brasiliensis* in an averagely supported clade with *Pollia*, and it is strongly supported as sister to *Polyspatha*. This relationship was deemed as morphologically surprising, but the present morphological dataset recovers this exact relationship, with strong statistical support. At this time, the only synapomorphy for the genus seems to be its geniculate pedicels. Nonetheless, *Fadeniella* is macromorphologically very different from its sister group, *Polyspatha*, which in turn is very distinct from *Pollia*. Thus, the recognition of a new genus is necessary.

**Accepted species and new combination.** Two species, including an undescribed one:

2.3.5.c.i. ***Fadeniella brasiliensis*** (C.B.Clarke) M.Pell., **comb. nov.** ≡ *Aneilema brasiliense* C.B.Clarke in A.L.P.P. de Candolle & A.C.P. de Candolle, Monogr. Phan. 3: 225. 1881.

urn:lsid:ipni.org:names:77379748-1 (https://www.ipni.org/n/77379748-1)

2.3.5.d. **Buforrestiinae** M.Pell., **subtrib. nov.** Type genus. *Buforrestia* C.B.Clarke.

urn:lsid:ipni.org:names:77379704-1 (https://www.ipni.org/n/77379704-1)

Figure 16H–I

**Description.** *Herbs* perennial, terrestrial. *Roots* thin, fibrous. *Rhizomes* present, short to elongate. *Stems* ascending to erect, sometimes prostrate, unbranched. *Leaves* spirally alternate, pseudopetiolate; epidermis lacking warts, with star-shaped idioblasts, mesophyll with tannin cells, marginal mechanical tissue absent. *Synflorescence* composed of a solitary main florescence; synflorescence leaves, if present, reduced to bladeless sheaths. *Main florescences (inflorescences)* terminal or axillary, sessile or pedunculate, many-branched or reduced to a solitary cincinnus; basal bract bracteose or leaf-like; cincinnus bracts persistent or not, bracteose to frondose, cup-shaped, perfoliate, membranous or herbaceous; cincinni alternate, sessile, rarely short-pedunculate, free from the inflorescence main axis, cincinnus peduncle and each other, few–many-flowered, flowers secund; bracteoles persistent or not, bracteose, canaliculate or cup-shaped, not perfoliate, membranous or herbaceous. *Flowers* bisexual and staminate, chasmogamous, enantiostylous, zygomorphic, flat to slightly campanulate; pedicel erect at post-anthesis and fruit; sepals 3, subequal to unequal, free, herbaceous; petals 3, sessile, rarely the laterals clawed, subequal to unequal, sometimes the medial cup-shaped, deliquescent, glabrous; androecium zygomorphic, dimorphic or heteromorphic, filaments free, stamens 3–6, sometimes the posterior modified into staminodes, posterior filaments shorter than the anteriors, glabrous, anthers dorsifixed, rimose, anterior filaments glabrous, anthers sub-basifixed or apicifixed, rimose, connective inconspicuous, anther sacs C-shaped or elliptic or round; staminodes if present (2–)3, posterior, filaments glabrous, antherodes dorsifixed, polliniferous, rimose; pollen exine tuberculate, micro-perforations sparse, pollen of the posterior anthers or antherodes sterile; ovary sessile, 3-locular, locules equal, style elongated, gently to strongly curved at apex, base tapering into the ovary, apex cylindrical, stylar canal present, stigma truncate. *Capsules* loculicidal, 3-valved, sometimes indehiscent. *Seeds* 1–many per locule, uniseriate; hilum linear; embryotega lateral to semilateral, concolourous with the testa.

**Distribution.** Pantropical but centred in Southeast Asia and the Malaysian Archipelago.

**Ecology.** Members of Buforrestiinae grow exclusively in the understory of tropical rainforests, sharing chartaceous to coriaceous and lustrous pseudopetiolate leaves, which are congested at the apex of the stems.

**Included genera.** *Buforrestia* and *Tricarpelema*.

**Notes.** *Buforrestia* has been traditionally associated with *Stanfieldiella*, but no morphological explanation was ever provided to support this hypothesis. Alternatively, *Tricarpelema* has been historically associated with *Aneilema* R.Br., *Dictyospermum* Wight, *Murdannia* Royle, and *Pollia* Thunb. based on a series of misinterpretations of these genera’s floral morphology.

2.3.5.e. **Floscopinae** M.Pell., **subtrib. nov.** Type genus. *Floscopa* Lour.

urn:lsid:ipni.org:names:77379705-1 (https://www.ipni.org/n/77379705-1)

Figure 16E–G

**Description.** *Herbs* perennial or annual, paludal to aquatic or terrestrial, rarely rupicolous. *Roots* thin, fibrous. *Rhizomes* absent. Stems prostrate to erect, branched or not. *Leaves* spirally alternate, rarely distichously alternate, sessile or pseudopetiolate; epidermis lacking warts, lacking star-shaped idioblasts, mesophyll with tannin cells, marginal mechanical tissue absent. *Synflorescence* composed of the main florescence plus 1–several coflorescences, rarely main florescence solitary; synflorescence leaves equal to the vegetative leaves, sometimes reduced in size. *Main florescences (inflorescences)* terminal or axillary, sessile or pedunculate, many-branched or reduced to a solitary cincinnus; basal bract bracteose; cincinnus bracts persistent, bracteose, canaliculate or cup-shaped, perfoliate or not, membranous or herbaceous; cincinni alternate, free from the inflorescence main axis, cincinnus peduncle and each other, (1–)many-flowered, flowers secund; bracteoles persistent or not, minute, flat or cup-shaped, not perfoliate, rarely perfoliate, membranous, scarious. *Flowers* bisexual or bisexual and staminate, chasmogamous, enantiostylous, zygomorphic, sometimes actinomorphic, bisexual, sometimes bisexual and staminate, flat; pedicel erect at post-anthesis and fruit; sepals 3, equal to subequal, free, membranous; petals 3, sessile, equal to subequal, deliquescent, glabrous; androecium zygomorphic, sometimes actinomorphic, dimorphic, sometimes monomorphic, filaments basally connate or free, stamens (5–)6, posterior filaments shorter than the anterior ones, sometimes equal to the anterior, glabrous, anthers sub-basifixed, rimose, anterior filaments, glabrous, anthers dorsifixed, rimose, connective inconspicuous to linear-tapered, anther sacs elliptic or round; staminode when present posterior (outer antesepalous stamen), antherode absent; pollen exine tuberculate, micro-perforations sparse, pollen of the posterior anthers sterile; ovary sessile or shortly stipitate, 2–3-locular, locules equal, gently curved at apex, base cylindrical, rarely tapering into the ovary, apex cylindrical, stylar canal present, stigma truncate to capitate. *Capsules* loculicidal, 2–3-valved. *Seeds* 1–many per locule, uniseriate; hilum linear; embryotega lateral or semilateral or dorsal, much lighter than the testa.

**Distribution.** Pantropical but centred in tropical Africa, with only *Floscopa* extending into the Neotropics.

**Ecology.** Ecologically diverse, with each genus occupying different environments. *Floscopa* is aquatic or paludal, while *Saxofloscopa* is rupicolous, and *Stanfieldiella* is terrestrial and limited to forest understories.

**Included genera.** *Floscopa*, *Saxofloscopa*, and *Stanfieldiella*.

**Notes.** Despite their striking similarity, the genera in Floscopineae have historically not been consistently considered to be closely related to each other. *Floscopa* has generally been treated as more or less systematically isolated, either included in its own informal group or a broader and generally polyphyletic concept of other tribes. *Stanfieldiella* was originally segregated from *Buforrestia*, causing these morphologically distinct genera to continue to be associated with each other. Finally, the herein-proposed *Saxofloscopa* had its sole species initially placed in *Tricarpelema* despite the lack of morphological and geographical evidence supporting that decision. However, *Floscopa*, *Saxofloscopa*, and Stanfieldiella share very peculiar seeds, with the embryotega being much lighter in colour than the testa, to the extent that some species have seeds with black testa and white embryotega. Furthermore, these genera all share dense synflorescences (which give them their characteristic broom-like appearance), glandular macrohairs generally covering the inflorescences, pedicels and sepals, minute and membranous bracteoles, enantiostylous flowers, posterior stamens producing sterile pollen, and stipitate capsules.

2.3.5.f. ***Saxofloscopa*** M.Pell., **gen. nov.** Type species: *Saxofloscopa africana* (Faden) M.Pell. (≡ *Tricarpelema africanum* Faden).

urn:lsid:ipni.org:names:77379733-1 (https://www.ipni.org/n/77379733-1)

Figure 16E

= *Tricarpelema* subg. *Keatingia* Faden, Novon 17: 166. 2007, **syn. nov.** Type species. *Tricarpelema africanum* Faden [≡ *Saxofloscopa africana* (Faden) M.Pell.].

**Description.** *Herbs* chamaephytes, perennial, base definite, tufted to mat-forming, rupicolous. *Roots* thin, fibrous. *Rhizome* absent. *Stems* prostrate, ascending to erect at apex, branched at base or unbranched, succulent, becoming fibrous and wiry with age, rooting at the lower nodes; internodes at base glabrous or becoming glabrous with age, ± covered by overlapping and persistent leaf-sheaths, at apex sparsely glandular-pubescent, not covered by the leaf-sheaths. *Leaves* distichously alternate, evenly distributed along the stems, slightly reduced towards the apex; ptyxis involute; sheaths glandular-pubescent, margin ciliate with glandular hairs; blades moderately succulent, falcate to strongly recurved, flat to slightly conduplicate when fresh, strongly conduplicate when dry, sometimes marcescent and persisting with the sheaths, adaxially glandular-pubescent, abaxially sparsely glandular-pubescent to glandular-pubescent, base symmetric, round-amplexicaulous, margin slightly revolute, scabrid with prickle hairs, at base ciliate with a mixture of glandular and prickle hairs, apex acuminate; midvein inconspicuous, adaxially very slightly impressed, abaxially inconspicuously acute, secondary veins 2–3 pairs, adaxially inconspicuous, abaxially inconspicuous, becoming more conspicuous on both sides when dry. *Synflorescence* composed of the main florescence plus 1–several coflorescences, rarely main florescence solitary, thyrsoid, sometimes forming a second-degree synflorescence; synflorescence leaves distichously alternate, sometimes spirally alternate, smaller than the vegetative leaves, becoming conspicuously much smaller towards the apex, bracteose and hyaline. *Main florescences (inflorescences)* terminal or axillary in the upper nodes, pedunculate, few–many-branched; basal bract leaf-like to bracteose; peduncle sparsely glandular-pubescent; cincinnus bracts bracteose, cup-shaped, perfoliate, membranous, hyaline; cincinni 2–7, alternate to sub-opposite, rarely verticillate, pedunculate, elongate, ascending, 3–7-flowered; bracteoles conspicuous, persistent, cup-shaped, perfoliate, membranous, scarious. *Flowers* chasmogamous, enantiostylous, zygomorphic due to the morphology of the sepals, petals and stamens, bisexual and staminate; floral buds obovoid; pedicels deflexed at pre-anthesis, erect at anthesis, post-anthesis and fruit, glabrous to sparsely glandular-pubescent; sepals 3, subequal, navicular, membranous, sparsely glandular-pubescent, the dorsal one slightly larger than the laterals, cucullate at apex; petals 3, subequal, sessile, free, strongly reflexed at anthesis, glabrous, the paired ones slightly narrower than the medial, flat to slightly convex, the medial concave; stamens 6, 3 posterior and 3 anterior, posterior stamens shorter than the anterior ones, unequal, filaments glabrous, straight or almost so, very gently deflexed at apex, anthers sub-basifixed, connective obdeltate to obcordate, base bilobed, yellow, anther sacs parallel, rimose, polliniferous, pollen sterile, central medial posterior stamen longer than the laterals, filament straight or almost so, apex abruptly deflexed, lacking the anther, anterior stamens unequal, lateral stamens divergent, filaments straight to very gently J-shaped, apex gently recurved, anthers dorsifixed, connective elliptic to ovate, marron to purplish-black, anther sacs divergent, rimose, polliniferous, pollen fertile, medial anterior stamen slightly longer than the laterals, filament gently J-shaped, apex recurved, anthers dorsifixed, connective widely elliptic to widely ovate, base cordate, apex emarginate, marron to purplish-black, anther sacs parallel, rimose, polliniferous, pollen fertile; ovary shortly-stipitate, 3-locular, ellipsoid to lanceoloid, smooth, glabrous, ovules 4–5 per locule, uniseriate, style glabrous, ca. the same length as the medial anterior stamen, apex gently recurved towards, cylindric, base tapering into the ovary, stigma truncate. *Capsules* oblongoid to ellipsoid, thin-walled, shortly-stipitate to stipitate, tan-coloured to light brown, shiny, sometimes with small dark brown specks, 3-valved, valves remaining oblique to almost upright after dehiscence, apex truncate, style base not persistent. *Seeds* 2–5 per locule, uniseriate, exarillate, terminal seeds triangular to triangular-ovate, medial seeds rectangular to trapezoidal, ventrally flattened, not cleft towards the embryotega, greyish-brown, testa scrobiculate, farinose, farinae white; hilum linear, ca. the same length as the seed; embryotega lateral, conspicuous, without a prominent apicule, white.

**Etymology.** From the Latin *saxō* (derived from the Proto-Italic *saksom, meaning “rock” or “stone”) + the generic name *Floscopa*, in reference to its similarities to *Floscopa*, but growing on rocky outcrops instead of aquatic environments. Furthermore, *Floscopa* comes from the Latin *flos* (derived from the Proto-Italic *flōs, meaning “flower”) + *scōpa* (derived from the Proto-Italic *skōpā, meaning “broom”), in reference to their broom-like synflorescences.

**Distribution.** *Saxofloscopa* is endemic to tropical central Africa, occurring in southeastern Cameroon, continental Equatorial Guinea, and northern Gabon.

**Ecology.** *Saxofloscopa* grows on inselbergs in the forest zone, in full sun in shallow soil, rocky slabs and cliffs, fringes of taller vegetation, and less often in thickets or shady, moist environments, between 500–950 m above sea level.

**Notes.** *Saxofloscopa* is superficially similar to the Asian *Tricarpelema*, being originally described as a member of this genus by Faden [71] due to its glandular-pubescent inflorescences, flowers with zygomorphic androecium (with distinct posterior and anterior stamens), 3-carpellate gynoecium, and 3-valvate capsules. Nonetheless, these are either plesiomorphic characters in tribe Commelineae (i.e., zygomorphic androecium with distinct posterior and anterior stamens) or at the family level (i.e., 3-carpellate gynoecium and 3-valvate capsules). Therefore, they are not good diagnostic characters. In the same publication, Faden [71] compares *Saxofloscopa africana* (as *Tricarpelema africanum*) to *Floscopa* and *Stanfieldiella* due to their “compound thyrsi” (i.e., dense and many-branched synflorescence) and glandular-pubescent inflorescences. However, the author failed to observe their shared erect pedicels at post-anthesis, stipitate ovary and capsules, and embryotega much lighter in colour than the seed testa, which characterise this clade.

*Saxofloscopa* is very peculiar in presenting a unique combination of morphological and ecological characters, aside from possessing some that are very uncommon in the family as a whole. It is morphologically intermediate between *Floscopa* and *Stanfieldiella*, but does not fit comfortably within any of them. Its overall inflorescence and flower morphology are more reminiscent of *Floscopa*. However, it readily differs from *Floscopa* by its cup-shaped bracteoles (vs. flat in *Floscopa*), gynoecium 3-carpellate (vs. 2-carpellate), capsules oblongoid to ellipsoid and 3-valved (vs. cordate to subcordate and 2-valved), and seeds rugose with lateral embryotega (vs. smooth to costate with dorsal embryotega). It is more closely related to *Stanfieldiella*, based on both morphology (Figure 2, Figure 3 and Figure 11) and molecular data (Pellegrini, unpubl. data), but can be readily differentiated due to its leaves being distichously alternate, sessile, succulent and glandular-pubescent (vs. spirally alternate, pseudopetiolate, membranous and glabrous to eglandular-pubescent in *Stanfieldiella*), flowers bisexual or staminate (vs. always bisexual), petals pink to mauve to purple (vs. white, sometimes with dark pink veins), androecium zygomorphic (vs. actinomorphic), style base tapering into the ovary (vs. style base cylindric), and embryotega lateral (vs. dorsal to semidorsal). However, we do agree with Faden [71] in that *Saxofloscopa* is clearly distinct from *Floscopa* and *Stanfieldiella*. Thus, based on its phylogenetic placement and unique combination of morphological characters, we propose that *T. africanum* is best positioned in its own genus, *Saxofloscopa*.

**Accepted species and new combination.** A single accepted species:

2.3.5.f.i. ***Saxofloscopa africana*** (Faden) M.Pell., **comb. nov.** ≡ *Tricarpelema africanum* Faden, Novon 17: 160–163, f. 1. 2007.

urn:lsid:ipni.org:names:77379747-1 (https://www.ipni.org/n/77379747-1)

2.3.5.g. ***Stanfieldiella*** Brenan, Kew Bull. 14: 283. 1960. Type species. *Stanfieldiella imperforata* (C.B.Clarke) Brenan.

Figure 16F

**Distribution and ecology.** Australasia, from northeastern India to the Philippines and Borneo.

**Ecology.** Usually in the forest understory.

**Comments.** *Stanfieldiella* is currently understood as a small genus of seven species. A new taxonomic revision is necessary due to the great number of new specimens collected since the genus was described, the inclusion of *S. africana*, and the general short diagnosis provided in its first revision.

**Accepted species and new combinations.** A total of six species, including the new combinations below:

2.3.5.g.i. ***Stanfieldiella glabrisepala*** (De Wild.) M.Pell., **comb. & stat. nov.** ≡ *Stanfieldiella imperforata* var. *glabrisepala* (De Wild.) Brenan, Kew Bull. 14: 285. 1960 ≡ *Buforrestia glabrisepala* De Wild., Pl. Bequaert. 5: 224. 1931.

urn:lsid:ipni.org:names:77379701-1 (https://www.ipni.org/n/77379701-1)

2.3.5.g.ii. ***Stanfieldiella hirsuta*** (Brenan) M.Pell., **comb. & stat. nov.** ≡ *Stanfieldiella brachycarpa* var. *hirsuta* (Brenan) Brenan, Kew Bull. 14: 286. 1960 ≡ *Buforrestia brachycarpa* var. *hirsuta* Brenan, Kew Bull. 7: 455. 1953.

urn:lsid:ipni.org:names:77379702-1 (https://www.ipni.org/n/77379702-1)

2.3.5.h. ***Floscopa*** Lour., Fl. Cochinch. 1: 189, 192. 1790. Type species. *Floscopa scandens* Lour.

Figure 16G

**Distribution.** Pantropical, found in Central and South America, Africa (incl. Madagascar), and Australasia. This is the only non-monospecific genus in the Commelinaceae, which is exclusively paludal, aquatic or wetland-dependent.

**Ecology.** Species of *Floscopa* can be found growing in open water bodies, shaded water bodies, or flooded forest understories.

**Comments.** With the recent transfer of *Floscopa yunnanensis* to *Aneilema*, *Floscopa* is now a morphologically well-circumscribed and easily recognisable genus. On the other hand, its species are of difficult identification and delimitation, due to their plasticity and the poorly understood variation in floral characters. Taxonomy of the genus has been historically exceedingly reliant on vegetative characters, and a reinterpretation of the group taxonomy is urgently necessary.

**Accepted species and new combinations.** Currently with ca. 25 accepted species, including the new combinations below:

2.3.5.h.i. ***Floscopa hirsuta*** Hassk., Commelin. Ind. 165. 1870 ≡ *Floscopa glabrata* var. *hirsuta* (Kunth) C.B.Clarke in A.L.P.P. de Candolle & A.C.P. de Candolle, Monogr. Phan. 3: 270. 1881.

2.3.5.h.ii. ***Floscopa majuscula*** (C.B.Clarke) M.Pell., **stat. nov.** ≡ *Floscopa africana* var. *majuscula* C.B.Clarke, Fl. Trop. Afr. 8: 85. 1901 ≡ *Floscopa africana* subsp. *majuscula* (C.B.Clarke) Brenan, Kew Bull. 22: 387. 1968.

urn:lsid:ipni.org:names:77379958-1 (https://www.ipni.org/n/77379958-1)

2.3.5.h.iii. ***Floscopa pauciflora*** C.B.Clarke, Fl. Trop. Afr. [Oliver et al.] 8(1): 88. 1901 ≡ *Floscopa glomerata* subsp. *pauciflora* (C.B.Clarke) J.K.Morton, J. Linn. Soc., Bot. 60: 200. 1967.

2.3.5.h.iv. ***Floscopa petrophila*** (Gilg & Ledermann ex J.K.Morton) M.Pell., **stat. nov.** ≡ *Floscopa africana* subsp. *petrophila* Gilg & Ledermann ex J.K.Morton, J. Linn. Soc., Bot. 60: 200. 1967.

urn:lsid:ipni.org:names:77379709-1 (https://www.ipni.org/n/77379709-1)

2.3.5.h.v. ***Floscopa sprucei*** (C.B.Clarke) M.Pell., **stat. nov.** ≡ *Floscopa robusta* var. *sprucei* C.B.Clarke in A.L.P.P. de Candolle & A.C.P. de Candolle, Monogr. Phan. 3: 271. 1881.

urn:lsid:ipni.org:names:77379708-1 (https://www.ipni.org/n/77379708-1)

2.3.5.i. ***Murdannia*** Royle, Ill. Bot. Himal. Mts. 1: 403, pl. 95, f. 3. 1839, nom. cons. Type species. *Murdannia scapiflora* (Roxb.) Royle [= *M. edulis* (Stokes) Faden].

Figure 16C,D

**Distribution.** *Murdannia* is Pantropical, but centred in Asia, with only a handful of species currently recognised for the Neotropics and Africa [34].

**Ecology.** Its species are mainly associated with permanently or seasonally flooded environments across the genus distribution range.

**Comments.** *Anthericopsis* has been historically recognised as distinct from *Murdannia* and more commonly associated with the distantly related *Aneilema* [30]. Nonetheless, *Anthericopsis* is undisputedly closely related to *Murdannia*, being either recovered as sister to one another or with *Anthericopsis* nested within *Murdannia*. In the case of retaining *Anthericopsis* as distinct from *Murdannia*, the latter would have no morphological synapomorphy. For these reasons, *Anthericopsis* is here reduced to a synonym of *Murdannia*.

**Accepted species and new combination.** Ca. 80 species, including several undescribed species and the new combinations below:

2.3.5.i.i. ***Murdannia ahuchawlensis*** (Pagag & Borthakur) M.Pell., **stat. nov.** ≡ *Murdannia triquetra* var. *ahuchawlensis* Pagag & Borthakur, as “*ahuchawlense*”, Phytotaxa 525(2): 163 2021 ≡ *Murdannia keisak* var. *ahuchawlensis* (Pagag & Borthakur) Nandikar, as “*ahuchawlense*”, Commelin. India 156. 2024.

urn:lsid:ipni.org:names:77379755-1 (https://www.ipni.org/n/77379755-1)

2.3.5.i.ii. ***Murdannia bodinieri*** (H.Lév. & Vaniot) M.Pell., **comb. nov.** ≡ *Aneilema bodinieri* H.Lév. & Vaniot, Mém. Soc. Sci. Nat. Math. Cherbourg 35: 389. 1896.

urn:lsid:ipni.org:names:77379753-1 (https://www.ipni.org/n/77379753-1)

**Notes.** This new combination is made to accommodate the morphologically distinct Chinese specimens previously placed under *M. hookeri* (C.B.Clarke) G.Brückn.

2.3.5.i.iii. ***Murdannia flavanthera*** (Nandikar & Gurav) M.Pell., **stat. nov.** ≡ *Murdannia spirata* subsp. *flavanthera* (Nandikar & Gurav) Nandikar, Fl. India 28: 351. 2024 ≡ *Murdannia spirata* var. *flavanthera* Nandikar & Gurav, Phytodiversity 2(1): 93. 2015.

urn:lsid:ipni.org:names:77379715-1 (https://www.ipni.org/n/77379715-1)

2.3.5.i.iv. ***Murdannia glabrisepala*** (Faden) M.Pell., **stat. nov.** ≡ *Murdannia vaginata* var. *glabrisepala* Faden, Novon 11(1): 27. 2001.

urn:lsid:ipni.org:names:77379713-1 (https://www.ipni.org/n/77379713-1)

2.3.5.i.v. ***Murdannia longifolia*** (Hook.) M.Pell., **comb. & nov.** ≡ *Aneilema longifolium* Hook., Exot. Fl.: t. 204. 1826 ≡ *Commelina longifolia* (Hook.) Spreng., Syst. Veg., ed. 16. 4(2): 25. 1827, nom. illeg. non *C. longifolia* Lam.

urn:lsid:ipni.org:names:77379711-1 (https://www.ipni.org/n/77379711-1)

2.3.5.i.vi. ***Murdannia neglecta*** (Nandikar) M.Pell., **stat. nov.** ≡ *Murdannia spirata* subsp. *neglecta* Nandikar, Commelin. India: 178. 2024.

urn:lsid:ipni.org:names:77379710-1 (https://www.ipni.org/n/77379710-1)

2.3.5.i.vii. ***Murdannia parviflora*** (Faden) M.Pell., **stat. nov.** ≡ *Murdannia spirata* var. *parviflora* Faden, Novon 11(1): 25. 2001 ≡ *Murdannia spirata* subsp. *parviflora* (Faden) Nandikar, Commelin. India: 180. 2024.

urn:lsid:ipni.org:names:77379714-1 (https://www.ipni.org/n/77379714-1)

2.3.5.i.viii. ***Murdannia perennis*** (Faden) M.Pell., **stat. nov.** ≡ *Murdannia dimorphoides* subsp. *perennis* Faden, Novon 11(1): 24. 2001.

urn:lsid:ipni.org:names:77379712-1 (https://www.ipni.org/n/77379712-1)

2.3.5.i. ix. ***Murdannia secunda*** (Wight) M.Pell., **comb. nov.** ≡ *Aneilema secundum* Wight, Icon. Pl. Ind. Orient.: t. 2074. 1853.

urn:lsid:ipni.org:names:77379716-1 (https://www.ipni.org/n/77379716-1)

**Notes.** *Murdannia simplex* (Vahl) Brenan s.lat. is recovered as non-monophyletic based on molecular data (Pellegrini, unpubl. data), with M. simplex s.str. being an African endemic. Thus, *M. sinica* (Ker Gawl.) G.Brückn. and *M. stictosperma* Brenan are here reestablished, together with both needed new combinations (i.e., *M. longifolia* and *M. secunda*), to accommodate all recovered lineages.

2.3.5.i.x. ***Murdannia tradescantioides*** (Chiov.) M.Pell., **comb. nov.** ≡ *Anthericopsis tradescantioides* Chiov., Webbia 8: 40. 1951.

urn:lsid:ipni.org:names:77379707-1 (https://www.ipni.org/n/77379707-1)

2.3.5.j. **Pseudoparidinae** M.Pell., **subtrib. nov.** Type genus. *Pseudoparis* H.Perrier.

urn:lsid:ipni.org:names:77379706-1 (https://www.ipni.org/n/77379706-1)

Figure 16J

≡ Pseudoparideae Pichon, Notul. Syst. (Paris) 12: 240. 1946. Type genus: *Pseudoparis* H.Perrier

**Description.** *Herbs* perennial, terrestrial. *Roots* thin, fibrous, with ellipsoid to fusiform tubers at apex. *Rhizomes* present, short. *Stems* ascending to erect, generally unbranched. *Leaves* spirally alternate, sessile or pseudopetiolate; epidermis lacking warts, lacking star-shaped idioblasts, mesophyll with tannin cells, marginal mechanical tissue absent. *Synflorescence* composed of a solitary main florescence, terminal or axillary to sub-basal; synflorescence leaves reduced to bladeless sheaths. *Main florescences (inflorescences)* terminal, sessile or pedunculate, few–many-branched; basal bract spathaceous; cincinnus bracts persistent, bracteose, canaliculate or cup-shaped, not perfoliate, herbaceous; cincinni alternate, sessile to short-pedunculate, free from the inflorescence main axis, cincinnus peduncle and each other, many-flowered, flowers secund; bracteoles conspicuous, persistent, bracteose, cup-shaped, not perfoliate, membranous. *Flowers* bisexual and staminate, chasmogamous, non-enantiostylous, zygomorphic, flat; pedicel deflexed at post-anthesis and fruit; sepals 3, subequal, free, herbaceous; petals 3, sessile, unequal, free, glabrous, trilobed; androecium zygomorphic, dimorphic, filaments free, stamens (3–)4, posterior filaments shorter than the anterior, glabrous, anthers basifixed, rimose, anterior filament glabrous, anther sub-basifixed, rimose, connective inconspicuous, anther sacs elliptic or round; pollen exine tuberculate, micro-perforations sparse, pollen of the posterior anthers sterile; ovary sessile, 3-locular, locules equal, style conspicuous, gently curved at apex, base tapering into the ovary, apex cylindrical, stigma truncate. *Capsules* loculicidal, 3-valved, (?) rarely indehiscent. *Seeds* many per locule, uniseriate; hilum linear; embryotega lateral to semilateral, concolourous with the testa.

**Distribution.** Endemic to Madagascar.

**Ecology.** Grows in the understory of seasonally dry forests.

**Included genus.** *Pseudoparis*.

**Notes.** *Pseudoparis* has been traditionally considered a genus of dubious association in the family. Indeed, *Pseudoparis* is morphologically peculiar in several aspects, including its peculiar trilobed petals, unique androecium morphology, and its mostly axillary to sub-basal synflorescences. However, it shares several characters with other members of tribe Commelineae, as its stomata with 6 subsidiary cells (where the terminal pair is smaller than the second lateral pair), and pollen grains perforate with acute exine elements that are closer to each other in the transitional zone. Due to its peculiarity and isolated position in the tribe, it seems logical to place *Pseudoparis* in its own subtribe.

2.3.5.k. ***Streptolirion*** Edgew., Proc. Linn. Soc. Lond. 1: 254. 1845. Type species. *Streptolirion volubile* Edgew.

Figure 17C

**Distribution.** Japan to the Himalayas and Indo-China, can be found growing in the understory of rainforests.

**Ecology.** It can be found growing in the understory of rainforests.

**Comments.** Despite its reduced size, *Streptolirion* needs a taxonomic revision since the taxonomic status of most of its species has historically been questioned. Based on the specimens seen during the development of this study, the reestablishment of *S. lineare* is needed, together with a new combination.

**Accepted species and new combinations.** A total of four species, including the combinations below:

2.3.5.k.i. ***Streptolirion khasianum*** (C.B.Clarke) M.Pell., **stat. nov.** ≡ *Streptolirion volubile* var. *khasianum* C.B.Clarke in A.L.P.P. de Candolle & A.C.P. de Candolle, Monogr. Phan. 3: 262. 1881 ≡ *Streptolirion volubile* subsp. *khasianum* (C.B.Clarke) D.Y.Hong, Acta Phytotax. Sin. 12(4): 463. 1974.

urn:lsid:ipni.org:names:77379797-1 (https://www.ipni.org/n/77379797-1)

2.3.5.k.ii. ***Streptolirion angustifolium*** (Aver.) M.Pell., **stat. nov.** ≡ *Streptolirion volubile* var. *angustifolium* Aver., as “*angustifolia*”, Pl. Diversity Fl. Veg. Bat Dai Son: 155. 2021.

urn:lsid:ipni.org:names:77379764-1 (https://www.ipni.org/n/77379764-1)

2.3.5.l. ***Plowmanianthus*** Faden & C.R.Hardy in Hardy & Faden, Syst. Bot. 29(2): 316–318. 2004. Type species. *Plowmanianthus perforans* Faden & C.R.Hardy.

Figure 17F

**Distribution.** Panama to Peru, Colombia, Ecuador and Northern Brazil.

**Ecology.** Found growing in the understory of rainforests.

**Accepted species and new combination.** A total of six species, including the new combination below:

2.3.5.l.i. ***Plowmanianthus robustus*** (C.R.Hardy & Faden) M.Pell., **stat. nov.** ≡ *Plowmanianthus grandifolius* subsp. *robustus* C.R.Hardy & Faden, Syst. Bot. 29(2): 323–324, f. 4C–D, 6B, D–F, 11B, 12E. 2004.

urn:lsid:ipni.org:names:77379766-1 (https://www.ipni.org/n/77379766-1)

2.3.5.m. ***Cyanotis*** D.Don, Prodr. Fl. Nepal.: 45. 1825, nom. cons. Type species*. Cyanotis barbata* D.Don. [≡ *C. vaga* (Lour.) Schult. & Schult. f.].

Figure 17S,T

**Distribution.** Africa (incl. Madagascar), Asia and Oceania.

**Ecology.** It can be found generally growing in open areas, but also in the understory of rainforests, in dry or flooded environments.

**Comments.** Historically, *Belosynapsis* and *Cyanotis* were differentiated by cincinni elongation, petal connation, inflation of the filaments, and anther dehiscence. Nonetheless, molecular and morphological studies have shown *Belosynapsis* to be nested within *Cyanotis*. The genus needs a taxonomic revision, uniting the information gathered in the past years by floristic treatments and other local studies. The evolution and morphology of underground and storage organs in *Cyanotis* seem to be of systematic and taxonomic interest and should be studied soon. Finally, seed testa ornamentation is traditionally used in the taxonomy of Commelinaceae. Nonetheless, the complexity of the ornamentation patterns in *Cyanotis* has precluded its proper use. Thus, studies focusing on these patterns might shed some much-needed light on the taxonomy of this complex group.

**Accepted species and new combinations.** A total of ca. 70 species, including several undescribed species and the new combinations below:

2.3.5.m.i. ***Cyanotis cormosa*** M.Pell., **nom. nov.** ≡ *Cyanotis paludosa* subsp. *bulbifera* Faden, Fl. Trop. E. Africa, Commelin.: 19. 2012, non *Cyanotis bulbifera* Hutch.

urn:lsid:ipni.org:names:77379762-1 (https://www.ipni.org/n/77379762-1)

2.3.5.m.ii. ***Cyanotis gracilis*** (Schnell) M.Pell., **stat. nov.** ≡ *Cyanotis lanata* var. *gracilis* Schnell, Rev. Gén. Bot. 57: 287 1950 ≡ *Cyanotis longifolia* var. *gracilis* (Schnell) Schnell, Bull. Inst. Fondam. Afrique Noire, Sér. A, Sci. Nat. 19: 733. 1957.

urn:lsid:ipni.org:names:77379761-1 (https://www.ipni.org/n/77379761-1)

2.3.5.m.iii. ***Cyanotis madagascarica*** (C.B.Clarke) M.Pell., **stat. nov.** ≡ *Cyanotis nodiflora* var. *madagascarica* C.B.Clarke Monogr. Phan. 3: 258. 1881 ≡ *Cyanotis nodiflora* subsp. *madagascarica* (C.B.Clarke) H.Perrier, Fl. Madagasc. 37: 35. 1938 ≡ *Cyanotis speciosa* subsp. *madagascarica* (C.B.Clarke) Faden, Kew Bulletin 62(1): 140. 2007.

urn:lsid:ipni.org:names:77379767-1 (https://www.ipni.org/n/77379767-1)

2.3.5.m.iv. ***Cyanotis papyracea*** M.Pell., **nom. nov**. ≡ *Cyanotis speciosa* subsp. *bulbosa* Faden, Fl. Trop. E. Africa, Commelin.: 31. 2012, non *Cyanotis bulbosa* H.Lév.

urn:lsid:ipni.org:names:77379763-1 (https://www.ipni.org/n/77379763-1)

2.3.5.m.v. ***Cyanotis uda*** (C.B.Clarke) M.Pell., **stat. nov.** ≡ *Cyanotis somaliensis* var. *uda* C.B.Clarke, Ann. Mus. Congo Belge, Bot. sér. 1, V, 1: 223. 1906.

urn:lsid:ipni.org:names:77379765-1 (https://www.ipni.org/n/77379765-1)

2.3.5.m.vi. ***Cyanotis vinacea*** M.Pell., **nom. nov.** ≡ *Cyanotis repens* subsp. *robusta* Faden & D.M.Cameron, Novon 15(1): 113–116, figs. 1–2. 2005, non *Cyanotis robusta* Oberm.

urn:lsid:ipni.org:names:77379768-1 (https://www.ipni.org/n/77379768-1)

2.3.5.n. ***Nivoanthus*** M.Pell., **gen. nov.** Type species. *Nivoanthus madagascaricus* (C.B.Clarke) M.Pell. (≡ *Coleotrype madagascarica* C.B.Clarke).

urn:lsid:ipni.org:names:77379777-1 (https://www.ipni.org/n/77379777-1)

Figure 17O

= *Coleotrype* sect. *Madecassae* H.Perrier, Notul. Syst. (Paris) 5(3): 198. 1936, **syn. nov.** Type species. *Coleotrype madagascarica* C.B.Clarke [≡ *Nivoanthus madagascaricus* (C.B.Clarke) M.Pell.].

**Description.** *Herbs* geophytes, base definite, solitary or tufted, perennial, terrestrial. *Roots* tuberous, fusiform. *Rhizome* absent. *Stems* ascending to erect, unbranched to branched at upper half, herbaceous to succulent, rooting only at the lower nodes. *Leaves* spirally alternate, evenly distributed along the stems, slightly reduced towards the apex, subpetiolate; ptyxis involute; sheaths glandular-pubescent, margin ciliate with glandular hairs; blades herbaceous to moderately succulent, flat to slightly conduplicate, adaxially glabrous to pubescent, abaxially glabrous to pubescent, base symmetric, cuneate, margin flat, glabrous to ciliate, apex acuminate to caudate; midvein conspicuous, adaxially impressed to canulate, abaxially obtuse, secondary veins 3–5 pairs, adaxially inconspicuous, abaxially conspicuous, becoming more conspicuous on both sides when dry. *Synflorescence* composed of the main florescence plus several coflorescences, thyrsoid; synflorescence leaves sometimes pink to magenta in the basal 1/3 to 2/3, slightly smaller than the vegetative leaves, becoming smaller towards the apex. *Main florescences (inflorescences)* axillary in the upper nodes, sessile, perforating the subtending leaf-sheath, few-branched; basal bract bracteose, bicarinate, herbaceous; peduncle inconspicuous; cincinnus bracts bracteose, unequal, herbaceous; cincinni 2–4, glomerulate, sessile, contracted, 1–2-flowered; bracteoles conspicuous, bracteose, persistent, herbaceous. *Flowers* chasmogamous, enantiostylous, zygomorphic due to the morphology of the sepals, petals and stamens, bisexual; floral buds ellipsoid; pedicels inconspicuous, upright to erect at pre-anthesis, at anthesis, post-anthesis and fruit; sepals 3, subequal, connate at base, cannulate, chartaceous, sparsely pubescent, the dorsal one slightly larger than the laterals, apex cucullate; petals 3, subequal to unequal, clawed, connate, tubular, hypocrateriform, glabrous or margin barbate at base with moniliform hairs, the paired ones longer and broader than the medial, blade patent, flat, medial free or fused to the posterior filaments, blade flat to cup-shaped; stamens 6, subequal to unequal, 5 posterior and 1 anterior, posterior stamens connate, fused to the medial petal blade, filaments inserted in the corolla tube at 2 levels, straight to gently sinuate, glabrous or densely barbate with moniliform throughout, anthers basifixed, connective conspicuous or not, sagittate to quadrate, anther sacs parallel but divergent at base or parallel but divergent at base and apex or parallel but connivent at base and apex, anther sacs symmetric or not, poricidal, polliniferous, pollen fertile, anterior stamen free, straight to gently sinuate, glabrous or densely barbate with moniliform throughout, anthers basifixed, connective conspicuous or not, sagittate to quadrate, anther sacs parallel but divergent at base or parallel but divergent at base and apex or parallel but connivent at base and apex, anther sacs symmetric or not, poricidal, polliniferous, pollen fertile; ovary sessile, 3-locular, ellipsoid to lanceoloid, smooth, pubescent, dorsal locule (0–1–)2-ovulate, ventral locules 2-ovulate, ovules uniseriate, style glabrous, J-shaped, longer than the stamens, apex gently recurved towards, cylindric, base tapering into the ovary, stigma truncate. *Capsules* lanceoloid to ovoid, thin-walled, sessile, tan-coloured to light brown, opaque, 3-valved, dorsal locule (0–1–)2-seeded, ventral locules 2-seeded, apex acute, style base not persistent. *Seeds* (0–1–)2 per locule, exarillate, ovate to reniform, ventrally flattened, not cleft towards the embryotega, reddish-brown to maroon, testa rugose, farinose, farinae white; hilum C-shaped, ca. the same length as the seed; embryotega lateral, inconspicuous, without a prominent apicule, concolourous to the testa.

**Etymology.** The generic name honours botanist and friend Dr Nivohenintsoa Rakotonirina, a prominent Malagasy botanist and Biodiversity Team Leader for the Kew Madagascar Conservation Centre, who has dedicated her life to the study of the Malagasy flora, especially Fabaceae, Rubiaceae, Rutaceae and Xyridaceae.

**Distribution.** *Nivoanthus* is endemic to Madagascar.

**Ecology.** *Nivoanthus* can be found growing in the understory and edges of rainforests, seasonally dry forests, and savannahs in Madagascar.

**Notes.** *Nivoanthus* is superficially similar to the Continental Africa *Coleotrype*, where its species were originally placed. Nonetheless, *Coleotrype* s.str. has ejaculatory capsules, with seeds being mechanically squeezed out of the fruit with the assistance of the papery aril that covers the seeds, but remain inside the fruit at maturity. Additionally, *Nivoanthus* has sympodial growth, terminal and thyrsoid synflorescences, zygomorphic flowers, ventral sepals basally connate, petals subequal to unequal, medial petal fused to the posterior stamens, stamens unequal, posterior filaments fused, anthers generally held inside a deltoid hood-like structure (which gives its flowers a peculiar *Impatiens*-like morphology), and seeds rugose.

*Coleotrype* s.lat. was heterogeneous since its description, with the Malagasy species differing to such a degree that they were placed in their own section early on [72]. However, the indication that it might not represent a monophyletic group was highlighted by the lack of a synapomorphic character for it. In addition to the present analysis, molecular data also indicate that *Coleotrype* s.str. is more closely related to *Amischotolype* (incl. *Porandra*) than to *Nivoanthus*. One option would be merging both genera together, which would cause the otherwise morphologically cohesive *Amischotolype* to become heterogeneous and difficult to diagnose. Thus, the description of a new genus for the Malagasy species of *Coleotrype* is needed.

**Accepted species and new combination.** *Nivoanthus* is in dire need of a taxonomic revision. Currently, six species are accepted. However, they are very plastic and of difficult application, in addition to many infraspecific taxa and at least one undescribed species.

2.3.5.n.i. ***Nivoanthus baronii*** (Baker) M.Pell., **comb. nov.** ≡ *Coleotrype baronii* Baker, J. Linn. Soc., Bot. 22: 530. 1887.

urn:lsid:ipni.org:names:77379779-1 (https://www.ipni.org/n/77379779-1)

2.3.5.n.ii. ***Nivoanthus goudotii*** (C.B.Clarke) M.Pell., **comb. nov.** ≡ *Coleotrype goudotii* C.B.Clarke, in A.L.P.P. de Candolle & A.C.P. de Candolle, Monogr. Phan. 3: 238. 1881.

urn:lsid:ipni.org:names:77379780-1 (https://www.ipni.org/n/77379780-1)

2.3.5.n.iii. ***Nivoanthus luteus*** (H.Perrier) M.Pell., **comb. nov.** ≡ *Coleotrype lutea* H.Perrier, Notul. Syst. (Paris) 5: 199. 1936.

urn:lsid:ipni.org:names:77379781-1 (https://www.ipni.org/n/77379781-1)

2.3.5.n.iv. ***Nivoanthus madagascaricus*** (C.B.Clarke) M.Pell., **comb. nov.** ≡ *Coleotrype madagascarica* C.B.Clarke, in A.L.P.P. de Candolle & A.C.P. de Candolle, Monogr. Phan. 3: 238. 1881.

urn:lsid:ipni.org:names:77379782-1 (https://www.ipni.org/n/77379782-1)

2.3.5.n.v. ***Nivoanthus synantherus*** (H.Perrier) M.Pell., **comb. nov.** ≡ *Coleotrype synanthera* H.Perrier, Notul. Syst. (Paris) 5: 200. 1936.

urn:lsid:ipni.org:names:77379783-1 (https://www.ipni.org/n/77379783-1)

2.3.5.n.vi. ***Nivoanthus vermigerus*** (H.Perrier) M.Pell., **comb. nov.** ≡ *Coleotrype vermigera* H.Perrier, Notul. Syst. (Paris) 5: 206. 1936.

urn:lsid:ipni.org:names:77379778-1 (https://www.ipni.org/n/77379778-1)

2.3.5.o. ***Ivoniella*** M.Pell., **gen. nov.** Type species. *Ivoniella guatemalensis* (C.B.Clarke ex Donn.Sm.) M.Pell. [≡ *Tradescantia guatemalensis* C.B.Clarke ex Donn.Sm.].

urn:lsid:ipni.org:names:77339026-1 (https://www.ipni.org/n/77339026-1)

Figure 18G

= *Tradescantia* sect. *Coholomia* D.R.Hunt, Kew Bull. 35(2): 440. 1980, **syn. nov.** Type species. *Tradescantia guatemalensis* C.B.Clarke ex Donn.Sm. [≡ *Ivoniella guatemalensis* (C.B.Clarke ex Donn.Sm.) M.Pell.].

**Description.** *Herbs* chamaephytes, base definite, solitary, perennial, terrestrial or rupicolous. *Roots* thin and fibrous. *Rhizome* absent. *Stems* ascending or scrambling, generally leaning on bushes, sometimes trailing at base, herbaceous to slightly fibrous, branched only in the upper 1/3 or 2/3, rooting from basalmost nodes. *Leaves* distichously alternate, evenly distributed or congested at the apex of the stem, sessile or subpetiolate; ptyxis involute; sheaths remaining closed at maturity; ptyxis involute; blades flat, base symmetrical or asymmetrical, midvein conspicuous, secondary veins conspicuous. *Synflorescences* terminal, composed of a solitary main florescence partially concealed by a pair of leaves. *Inflorescences (main florescences)* consisting of a shortly pedunculate thyrse reduced to (1–)2 cincinni arranged side-by-side; inflorescence bract hyaline, tubular, inconspicuous; peduncle bracts absent; supernumerary bracts absent; main axis inconspicuous; cincinnus bracts reduced (bracteose), membranous, more or less unequal to each other, flat, straight, not saccate at base, free; cincinni sessile to very short-pedunculate, free from each other, not geniculate, contracted; bracteoles inconspicuous, triangular to ovate, hyaline, membranous. *Flowers* bisexual, actinomorphic, chasmogamous, flat; pedicel non-gibbous at apex, straight at anthesis and pre-anthesis, oblique at post-anthesis; floral buds oblongoid to ellipsoid or ovoid to widely ovoid; sepals equal, free, narrowly elliptic to elliptic, membranous, hyaline, navicular, dorsally not keeled, apex acute, persistent in fruit; petals sessile, equal, free, flat, white, sometimes light blue to blue to lilac-blue; stamens 6, arranged in two whorls, equal, filaments free from each other, free from the petals, straight at anthesis and post-anthesis, basally densely bearded with moniliform hairs, hairs ca, ½ the length of the filaments, white, anthers dorsifixed, rimose, connective rhombic, anther sacs elliptic, divergent, yellow; pollen yellow in vivo, released as monads, without adhering raphides, 1-sulcate, tectate-perforate to semitectate, endexine lacking ornamentation, sexine rugulate-fossulate, sulcal membrane irregularly verrucate-granulate; ovary sessile, subglobose to globose, smooth, entirely glandular-pubescent, 3-locular, locules equal, locules 2-ovulate, ovules uniseriate, style straight at anthesis and post-anthesis, cylindrical throughout, stigma truncate, pistil ca. the same length as the stamens. *Capsules* subglobose, light brown when mature, loculicidal, 3-valved, sometimes apiculate due to persistent style base. *Seeds* exarillate, 1–2 per locule, triangular to round-triangular, ventrally keeled, not cleft towards the embryotega, testa costate or foveolate, ridges or pits radiating from the embryotega, farinose or not, when present, farinae cream-coloured; embryotega dorsal, prominent but inside a deep cavity, apex truncate, generally covered by cream-coloured farinae; hilum linear.

**Etymology.** The generic name honours botanist and friend Dr Ivón Mercedes Ramírez Morillo (Centro de Investigación Científica de Yucatán), a prominent Venezuelan-born Mexican botanist who has dedicated her life to the study of Neotropical Monocots, especially Bromeliaceae and Orchidaceae.

**Distribution.** *Ivoniella* extends from Mexico to Nicaragua, and is disjunctively found in Venezuela (Pellegrini, in prep.).

**Ecology.** *Ivoniella* is found growing in the understory of seasonally dry forests.

**Notes.** Despite being recognised as an independent genus for the first time here, *Ivoniella* was previously highlighted to morphologically stand out from the remaining genera of Tradescantiinae s.str. [28].

**Accepted species.** A total of three species, including two undescribed species (Pellegrini & Hunt, in prep.):

2.3.5.o.i. ***Ivoniella guatemalensis*** (C.B.Clarke ex Donn.Sm.) M.Pell., **comb. nov.** ≡ *Tradescantia guatemalensis* C.B.Clarke ex Donn.Sm., Bot. Gaz. 18: 210. 1893 ≡ *Elasis guatemalensis* (C.B.Clarke ex Donn.Sm.) M.Pell., PhytoKeys 89: 42. 2017.

urn:lsid:ipni.org:names:77339027-1 (https://www.ipni.org/n/77339027-1)

2.3.5.p. **Callisiinae** M.Pell., **subtrib. nov.** Type genus. *Callisia* Loefl.

urn:lsid:ipni.org:names:77379775-1 (https://www.ipni.org/n/77379775-1)

Figure 18N–T

≡ Callisieae Pichon, Notul. Syst. (Paris) 12: 236. 1946. Type genus. *Callisia* Loefl.

**Description.** *Herbs* annual or perennial, terrestrial, rupicolous, paludal or aquatic, sometimes epiphytic. *Roots* thin and fibrous or tuberous. *Rhizomes* absent. *Stems* ascending to erect, branched or not. *Leaves* distichously or spirally alternate, sessile or pseudopetiolate; epidermis with silica crystals in specialized thick-walled cells, warted, lacking star-shaped idioblasts, mesophyll with tannin cells, marginal mechanical tissue absent. *Synflorescence* composed of a main florescence plus 1–many coflorescences, terminal or axillary; synflorescence leaves equal or subequal to the leaves or reduced to bladeless sheaths. *Main florescences (inflorescences)* terminal or axillary, sessile or pedunculate, 2(–3)-branched; basal bract tubular and hyaline; cincinnus bracts persistent, vestigial or bracteose, flat or canaliculate or both fused into a cup, not perfoliate, membranous or herbaceous or chartaceous; cincinni subopposite, sessile, rarely short-pedunculate, fused to the inflorescence main axis, fused to the cincinnus peduncle, fused to each other back to back, rarely fused to each other only at base, many-flowered, flowers secund; bracteoles conspicuous or not, persistent, bracteose, flat, not perfoliate, membranous or chartaceous or succulent or paleaceous. *Flowers* all bisexual or bisexual and staminate, rarely bisexual, staminate and pistillate, chasmogamous, non-enantiostylous, zygomorphic, rarely actinomorphic, flat or tubular, when tubular hypocrateriform or infundibuliform; pedicel reflexed at post-anthesis and fruit, rarely erect; sepals (2–)3, subequal, free, membranous or chartaceous or paleaceous, rarely succulent; petals (2–)3, sessile to clawed, equal, free, glabrous, entire; androecium actinomorphic or zygomorphic, dimorphic, filaments free, stamens (1–2–)3–6, antesepalous filaments shorter than the antepetalous, glabrous or bearded with moniliform hairs, anthers basifixed, rimose, antepetalous filaments glabrous or bearded with moniliform hairs, anther basifixed, rimose, connective quadrangular or sagittate or rhombic or flabellate, rarely inconspicuous, anther sacs C-shaped or elliptic or round; pollen exine microclavate or irregularly microclavate or insulate-cerebroid or verrucose-granulose, micro-perforations absent or sparse, sometimes pollen of the antepetalous anthers sterile, sulcal membrane ornamentation only slightly different from the exine; ovary sessile, (2–)3-locular, locules equal, style short to long, rarely inconspicuous, base cylindrical, straight to gently curved, apex cylindrical or obconic, stylar canal present, rarely absent, stigma truncate or capitulate or capitate, sometimes flabellate. *Capsules* loculicidal, (2–)3-valved. *Seeds* (1–)2 per locule, uniseriate, generally triangular, round-triangular or tetrahedral; hilum punctate to elliptic, rarely linear; embryotega dorsal, generally inside a deep cavity, concolourous with the testa.

**Distribution.** Neotropical, extending from the USA to Argentina, but centred in Mexico.

**Ecology.** Members of this subtribe are generally well adapted to very seasonal and open environments, with most species being slightly to greatly succulent, and sometimes presenting tuberous roots. However, *Aploleia* is found in seasonally dry forests, while several *Tripogandra* grow exclusively in the understory of rainforests.

**Included genera.** *Aploleia*, *Callisia*, *Cuthbertia*, *Hadrodemas*, *Huntiella*, and *Tripogandra*.

**Notes.** Members of Callisiinae have been traditionally considered to be closely related, with all of them included under a very broad and morphologically diverse *Callisia* s.lat. [24,58,73]. Despite being monophyletic and well-supported by morphology (see Discussion), the genera in this subtribe are readily differentiated based on floral morphology and reproductive biology (especially pollination syndromes). The recognition of six genera not only facilitates taxonomy (both at genus and species level), but also represents the least taxonomically and nomenclaturally disruptive option for this group.

2.3.5.q. ***Cuthbertia*** Small, Fl. S.E. U.S. 237, 1328. 1903 ≡ *Callisia* sect. *Cuthbertia* (Small) D.R.Hunt., Kew Bull. 41(2): 409. 1986. Type species. *Callisia rosea* (Vent.) D.R.Hunt [≡ *Cuthbertia rosea* (Vent.) Small].

Figure 18O

**Distribution.** Endemic to the USA, from Florida to Virginia.

**Ecology.** *Cuthbertia* is found growing in grasslands.

**Comments.** *Cuthbertia* was reduced to a section of *Callisia* by Hunt [73] with the aim of making the genus monophyletic. Nonetheless, as shown by several molecular and morphological phylogenies ([24,44,57], this study), Hunt’s [73] expansion of *Callisia* made the genus non-monophyletic, without the inclusion of the morphologically cohesive and well-defined *Tripogandra*. *Cuthbertia* is morphologically easily distinguished from the remaining genera of the *Callisia*/*Tripogandra* complex by its tuberous lanate roots, grass-like habit and leaves, leaf-blades with acicular hairs along the margin, much-reduced cincinnus bracts with a cleft or lobed apex, and flowers that mimic those of members of *Tradescantia* subg. *Tradescantia*, petals with crenulate margins, connectives quadrangular, anther sacs C-shaped, glandular microhairs with the distal cell wider at apex, leaf epidermis with striate thickenings and lacking warts, and stomata 2-celled.

**Accepted species and new combination.** A total of four species: *Cuthbertia graminea* Small, *C. leucantha* (Lakela) M.Pell., *C. ornata* Small, and *C. rosea* (Vent.) Small.

2.3.5.q.i. ***Cuthbertia leucantha*** (Lakela) M.Pell., **comb. & stat. nov.** ≡ *Cuthbertia graminea* fo. *leucantha* Lakela, Sida 5: 28. 1972 ≡ *Callisia graminea* fo. *leucantha* (Lakela) G.C.Tucker, J. Arnold Arbor. 70(1): 118. 1989.

urn:lsid:ipni.org:names:77379771-1 (https://www.ipni.org/n/77379771-1)

2.3.5.r. ***Huntiella*** M.Pell., **gen. nov.** Type species. *Huntiella navicularis* (Ortgies) M.Pell. [≡ *Callisia navicularis* (Ortgies) D.R.Hunt].

urn:lsid:ipni.org:names:77339030-1 (https://www.ipni.org/n/77339030-1)

Figure 18N

= *Callisia* sect. *Brachyphylla* D.R.Hunt., Kew Bull. 41(2): 409. 1986, **syn. nov.** Type species. *Callisia navicularis* (Ortgies) D.R.Hunt [≡ *H. navicularis* (Ortgies) M.Pell.].

= *Callisia* sect. *Lauia* D.R.Hunt., Kew Bull. 41(2): 409. 1986, **syn. nov.** Type species. *Callisia laui* (D.R.Hunt) D.R.Hunt [≡ *H. laui* (D.R.Hunt) M.Pell.].

**Description.** *Herbs* chamaephytes or geophytes, base definite or indefinite, solitary to tufted or mat-forming, perennial, frequently succulent, terrestrial or rupicolous. *Roots* thin, fibrous or tuberous, fusiform. *Rhizome* absent. *Stems* prostrate, ascending to erect at apex, succulent, unbranched to branched only at base or branched throughout, rooting only at the basal nodes or sometimes also along the distal ones when they touch the substrate. *Leaves* distichously or spirally alternate, evenly distributed along the stem or congested at the apex of the stem, sessile; ptyxis convolute; sheaths closed at maturity; blades conduplicate, navicular or strongly cannulate to semi-terete, base symmetric, margin glabrous or ciliate with acicular hairs, midvein conspicuous or not, secondary veins inconspicuous. *Synflorescences* terminal or restricted to the apex of the stem, composed of a solitary main florescence. *Inflorescences (main florescences)* consisting of a sessile to pedunculate double-cincinni fused back-to-back, inflorescence main axis with a 90° torsion; inflorescence bract hyaline, tubular, inconspicuous; peduncle bracts absent; supernumerary bracts absent; cincinnus bracts decussate in relation to the leaves, reduced (bracteose), crass, more or less unequal to each other, navicular, not saccate at base, fused back-to-back to each other; cincinni sessile, fused back-to-back, not geniculate, contracted; bracteoles expanded, imbricate, similar to the cincinnus bracts, opaque, crass. *Flowers* bisexual, actinomorphic, chasmogamous, flat; pedicel not gibbous at apex, upright at anthesis and pre-anthesis, reflexed at post-anthesis; sepals equal, free, chartaceous, navicular, dorsally keeled margin hyaline, apex acute, persistent in fruit; petals sessile, equal, free, flat; stamens 6, arranged in two series, equal to subequal, filaments free from each other, free from the petals, straight at anthesis and post-anthesis, bearded for 2/3 of their length with moniliform hairs, hair sparse, shorter than 1/2 the length of the filament, anthers basifixed, rimose, connective sagittate to triangular, yellow, anther sacs ellipsoid, divergent, yellow; pollen white in vivo, released as monads, without adhering raphides, 1-sulcate, tectate-perforate to semitectate, endexine lacking ornamentation, sexine rugulate-fossulate, sulcal membrane irregularly verrucate-granulate; ovary sessile, subglobose, white, glabrous, 3-locular, locules equal, locules 2-ovulate, ovules uniseriate, style straight at anthesis and post-anthesis, white, cylindrical at base and length, obconical at the apex, rarely slightly inflated at apex, stigma truncate or capitate, pistil ca. the same length as the stamens. *Capsules* subglobose to globose, light to medium brown when mature, loculicidal, 3-valved, sometimes apiculate due to the persistent style base. *Seeds* exarillate, 1–2 per locule, triangular to round-triangular, ventrally keeled, not cleft towards the embryotega, testa costate with ridges radiating from the embryotega, hilum punctate, embryotega dorsal, conspicuous, inside a deep cavity, covered by a cream farinae, apex acute.

**Etymology.** The generic name honours the late botanist and friend Dr David Richard Hunt (1938–2019), a prominent British botanist who specialised in Commelinaceae and Cactaceae. David greatly supported and contributed to my studies in Commelinaceae, especially with prolific discussions on the Tradescantia alliance and its members.

**Distribution.** *Huntiella* extends from Mexico to the US (Texas), growing in open grasslands and rocky environments.

**Ecology.** *Huntiella* is found growing in open grasslands and rocky environments.

**Notes.** *Huntiella* is here proposed to accommodate the lineage sister to *Cuthbertia*. A close relationship between both genera is supported by their tuberous roots, leaf blades with acicular hairs along the margin, reduced and apically lobed cincinnus bracts, anther sacs C-shaped, connectives quadrangular, glandular microhairs with distal cell wider at apex, and leaf-blade epidermis lacking warts. This is further supported by *H. hintoniorum* (as *Callisia hintoniorum*) being described as a member of *Callisia* sect. *Cuthbertia*, and not of *C.* sect. *Brachyphylla* [74]. Despite a generic name having never been previously proposed for this lineage, Pichon [75] had already indicated in his identification key the need to recognise *Tradescantia navicularis* as an independent genus. Nonetheless, the author never formalised this. *Huntiella* can be recognised by its very fleshy and generally navicular leaves, bracts decussate to the leaves (i.e., inflorescence with a 90° torsion in relation to the axis), fused back-to-back, bracteoles succulent, flowers that mimic those of *Tradescantia* subg. *Setcreasea*, petals with entire margins, connectives sagittate, apex acute, anther sacs divergent, leaf epidermis smooth and lacking warts, and stomata 4-celled.

**Accepted species and new combinations.** A total of four species:

2.3.5.r.i. ***Huntiella hintoniorum*** (B.L.Turner) M.Pell., **comb. nov.** ≡ *Callisia hintoniorum* B.L.Turner, Phytologia 75(4): 277–279, f. 1. 1993.

urn:lsid:ipni.org:names:77339034-1 (https://www.ipni.org/n/77339034-1)

2.3.5.r.ii. ***Huntiella laui*** (D.R.Hunt) M.Pell., **comb. nov.** ≡ *Phyodina laui* D.R.Hunt, Kew Bull. 33(3): 404. 1979 ≡ *Callisia laui* (D.R.Hunt) D.R.Hunt, Kew Bull. 38(1): 131. 1983.

urn:lsid:ipni.org:names:77339033-1 (https://www.ipni.org/n/77339033-1)

2.3.5.r.iii. ***Huntiella micrantha*** (Torr.) M.Pell., **comb. nov.** ≡ *Tradescantia micrantha* Torr., Rep. U.S. Mex. Bound. 2(1): 224. 1859 ≡ *Callisia micrantha* (Torr.) D.R.Hunt, Kew Bull. 38(1): 131. 1983 ≡ *Phyodina micrantha* (Torr.) D.R.Hunt, Kew Bull. 33: 404. 1979.

urn:lsid:ipni.org:names:77339032-1 (https://www.ipni.org/n/77339032-1)

2.3.5.r.iv. ***Huntiella navicularis*** (Ortgies) M.Pell., **comb. nov.** ≡ *Tradescantia navicularis* Ortgies, Gartenflora 26: 130, t. 901. 1877 ≡ *Phyodina navicularis* (Ortgies) Rohweder, Abh. Auslandsk., Reihe C, Naturwiss. 61(18): 151 1956 ≡ *Callisia navicularis* (Ortgies) D.R.Hunt, Kew Bull. 38(1): 132. 1983.

urn:lsid:ipni.org:names:77339031-1 (https://www.ipni.org/n/77339031-1)

2.3.5.s. ***Aploleia*** Raf., Fl. Tellur. 2: 17. 1836[1837]. Type species. *Aploleia diffusa* Raf., nom. superfl. [≡ *Aploleia monandra* (Sw.) H.E.Moore].

Figure 18P

**Distribution.** *Aploleia* extends from the US to Argentina and Brazil, but with *A. cordifolia* and *A. multiflora* restricted to North America, Central America, and the Antilles. It can be found growing in the understory of seasonally dry forests or rainforests, sometimes also in open environments.

**Ecology.** *Aploleia* can be found growing in the understory of seasonally dry forests or rainforests, sometimes also in open environments.

**Accepted species and new combination.** A total of three species: *Aploleia cordifolia* (Sw.) M.Pell., *A. monandra* (Sw.) H.E.Moore, and *A. multiflora* (M.Martens & Galeotti) H.E.Moore:

2.3.5.s.i. ***Aploleia cordifolia*** (Sw.) M.Pell., **comb. nov.** ≡ *Tradescantia cordifolia* Sw., Prodr.: 57. 1788 ≡ *Leiandra cordifolia* (Sw.) Raf., Fl. Tellur. 2: 17. 1836[1837]≡ *Callisia cordifolia* (Sw.) E.S.Anderson & Woodson, Contr. Arnold Arbor. 9: 117. 1935 ≡ *Phyodina cordifolia* (Sw.) Rohweder, Abh. Auslandsk., Reihe C, Naturwiss. 18: 151. 1956 ≡ *Tripogandra cordifolia* (Sw.) Aristeg., Bol. Acad. Ci. Fís. 25(68): 125. 1965.

urn:lsid:ipni.org:names:77379769-1 (https://www.ipni.org/n/77379769-1)

2.3.5.t. ***Tripogandra*** Raf., Fl. Tellur. 2: 16. 1836[1837], **emend. nov.** M.Pell. & Handlos. Type species. *Tripogandra multiflora* (Sw.) Raf.

Figure 18Q,R

**Distribution.** *Tripogandra* is widely distributed in the Neotropics, extending from Mexico to Argentina and Brazil.

**Ecology.** *Tripogandra* can be found growing in open, dry or flooded environments, but it is sometimes also found in the understory of seasonally dry forests or rainforests.

**Comments.** Christenhusz et al. [58] proposed the inclusion of *Tripogandra* in *Callisia* s.lat. due to it being nested in the latter. Nonetheless, this decision dramatically hampers the taxonomy of this unnecessarily complicated group. As stated by Pellegrini [28] and reinforced here, *Tripogandra* is easily diagnosable, even with the inclusion of *Callisia filiformis*, *C. gracilis*, and *Tradescantia triandra* Kunth (≡ *C. ciliata* Kunth, nom. illeg.). Furthermore, the remaining lineages of the *Callisia*/*Tripogandra* generic complex are also easily diagnosable and thus recognised by us as distinct genera. If *Callisia* sensu Christenhusz et al. [58] is accepted, the genus would only be differentiated based on three seed and two anatomical characters, which are most commonly absent in several herbarium specimens or very difficult to observe without proper anatomical methods. This way, recognising this unnecessarily broad and morphologically hyper-variable *Callisia* goes against the “practical and usable” requirements that the authors themselves affirmed as the base of their changes. With the circumscription accepted here for the lineages of the *Callisia*/*Tripogandra* generic complex, all six genera are easily differentiated from one another and other genera of Commelinaceae and are also supported by at least one easily observable synapomorphy.

**Accepted species and new combinations.** Over 30 species, including some still undescribed and the new combinations below:

2.3.5.t.i. ***Tripogandra australis*** (Handlos) M.Pell. & Handlos, **stat. nov.** ≡ *Tripogandra purpurascens* subsp. *australis* Handlos, Rhodora 77(810): 297–298. 1975.

urn:lsid:ipni.org:names:77379770-1 (https://www.ipni.org/n/77379770-1)

2.3.5.t.ii. ***Tripogandra filiformis*** (M.Martens & Galeotti) M.Pell. & Handlos, **comb. nov.** ≡ *Tradescantia filiformis* M.Martens & Galeotti, Bull. Acad. Roy. Sci. Bruxelles 9(2): 376. 1842 ≡ *Leptorhoeo filiformis* (M.Martens & Galeotti) C.B.Clarke, Diagn. Pl. Nov. Mexic. 3: 55. 1880 ≡ *Callisia filiformis* (M.Martens & Galeotti) D.R.Hunt, Kew Bull. 41(2): 410. 1986.

urn:lsid:ipni.org:names:77379772-1 (https://www.ipni.org/n/77379772-1)

2.3.5.t.iii. ***Tripogandra gracilis*** (Kunth) M.Pell. & Handlos, **comb. nov.** ≡ *Tradescantia gracilis* Kunth, Nov. Gen. Sp. (quarto ed.) 1: 261–262. 1815[1816] ≡ *Phyodina gracilis* (Kunth) Raf., Fl. Tellur. 2: 16. 1836[1837] ≡ *Aneilema gracile* (Kunth) Steyerm., Fieldiana, Bot. 28(1): 152. 1951, nom. illeg., as “*gracilis*” ≡ *Callisia gracilis* (Kunth) D.R.Hunt, Kew Bull. 38(1): 131. 1983.

urn:lsid:ipni.org:names:77379773-1 (https://www.ipni.org/n/77379773-1)

2.3.5.t.iv. ***Tripogandra procumbens*** (Willd.) M.Pell. & Handlos, **comb. nov.** ≡ *Tradescantia procumbens* Willd., Sp. Pl., ed. 4. 2: 19. 1799 ≡ *Descantaria procumbens* (Willd.) Schltdl., Linnaea 26: 141. 1853.

urn:lsid:ipni.org:names:77379774-1 (https://www.ipni.org/n/77379774-1)

**Notes.** These represent the northern South American and Central American specimens currently treated as *Tripogandra multiflora* (Sw.) Raf., while the southern South American ones refer to *Tripogandra parviflora* (Ruiz & Pav.) Steyerm., and the Antilles and North American ones represent *Tripogandra multiflora* s.str.

2.3.5.t.v. ***Tripogandra triandra*** (Kunth) M.Pell. & Handlos, **comb. nov.** ≡ *Tradescantia triandra* Kunth, Enum. Pl. 4: 93. 1843 ≡ *Tradescantia elongata* var. *triandra* (Kunth) C.B.Clarke in A.L.P.P. de Candolle & A.C.P. de Candolle, Monogr. Phan. 3: 304. 1881 ≡ *Callisia ciliata* Kunth, Nov. Gen. Sp. (quarto ed.) 1: 261. 1815[1816], nom. illeg., non *Callisia ciliata* Pers.

urn:lsid:ipni.org:names:77379776-1 (https://www.ipni.org/n/77379776-1)

## 3. Discussion

Due to the size and complexity of the present study, I organise the discussion into topics. The first one will provide a much-needed overview of the history and classification of Commelinales, covering previous and current ordinal and familial circumscriptions of plant families historically associated with Commelinales. The second will provide a morphological circumscription and will discuss the putative synapomorphies for Commelinales. The third one will discuss the relationship between the five families included in Commelinales. The fourth will cover the monophyly and circumscription of the families of Commelinales. The fifth will discuss the infrafamilial relationships in each family of Commelinales. The sixth will address generic limits in Commelinales in light of a combination of molecular and morphological data. The subsequent topics will discuss ecological and morphological matters relevant to the evolution and diversification of Commelinales. The final topic will discuss the congruence between molecular and morphological data in Commelinales, highlighting the importance of a combined approach.

### 3.1. Classification History of Commelinales

Depending on the author, the circumscription and characterisation of Commelinales have varied dramatically throughout the years, including their interpretation of morphology and, more recently, the data used to define it. In some instances, aside from its inconsistent circumscription, the order has also been treated under different descriptive ordinal names. The number of included families has also varied greatly, ranging from one to 11, depending on the author. Furthermore, the order has been historically associated with several Monocot orders (e.g., Bromeliales, Typhales, Velloziales, etc.; Table S4) but is currently placed in the commelinids, sister to Zingiberales [1,2,3,4,5,6,7,8,9,10,11,12,13].

Commelinales was originally described by Berchtold & Presl [76], based on Mirbel’s concept of Commelinaceae [77], composed solely by this family, which at the time also included the Mayacaceae (Table S4). Only a few years later, Reichenbach [78] proposed that Commelinaceae (which included under the tribe, Restioneae, the families Anarthriaceae, Centrolepidaceae, Rapateaceae, and Restionaceae; under Xyrideae the families Eriocaulaceae *pro parte*, Xyridaceae; and under Commelineae the families Commelinaceae, Eriocaulaceae *pro parte*, Flagellariaceae, Haemodoraceae *pro parte*, Mayacaceae, Philydraceae, and Pontederiaceae) was to be included in his order Caulo-Acroblastae, suborder Glumaceae (Tables S1 and S2). This order also included several other broadly circumscribed families, such as Bromeliaceae, Cyperaceae, Iridaceae, and Poaceae. However, the author provided no characterisation or rationale for either the order or the circumscription of each family. For these reasons, this work has been greatly ignored by most botanists throughout the years. In the following year, Dumortier [79] provided a rearrangement of Commelinales (as “Commelinariae”), characterising it as containing families with deliquescent petals, a superior ovary, and dehiscent fruits. It included the families Commelinaceae (incl. Mayacaceae), Dasypogonaceae, Eriocaulaceae, and Xyridaceae (Tables S1 and S2). Dumortier’s publication is considered the most relevant of the previous circumscriptions of the order since it was, until very recently, believed to be the original place of publication of the name Commelinales [80,81]. Endlicher [82] described the “class” Enantioblastae (which had its rank posteriorly corrected to order; the Code [62], Art. 16.3 & 32.3) as characterised by its heterochlamydeous flowers, dehiscent fruits, and orthotropous ovules. The order was composed of Centrolepidaceae, Commelinaceae, Eriocaulaceae, Restionaceae, and Xyridaceae (incl. Mayacaceae) (Tables S1 and S2). The circumscription and composition of Endlicher’s Enantioblastae were considerably similar to the concept of Commelinales adopted by Dumortier [79], which was faithfully followed by Eichler [83] in his classification system. Lindley [84] deviated from previous authors in adopting the name Xyridales instead of Commelinales. His new order was composed of Commelinaceae, Mayacaceae, Philydraceae, and Xyridaceae (incl. Dasypogonaceae and Rapateaceae) (Tables S1 and S2), being characterised by presenting trimerous and heterochlamydeous flowers, superior ovaries, and copious albumen. Furthermore, the author mentioned the similarity between Eriocaulaceae and Xyridaceae despite treating them in different orders. Engler [85] proposed the order Farinosae, which was characterised by plants that presented a mealy endosperm, as the name suggests. Aside from that, the Farinosae were further characterised by presenting a deliquescent corolla, a superior ovary, and dehiscent fruits [85]. The order was composed of 11 families, some of which were rarely considered to be closely related: Bromeliaceae, Centrolepidaceae, Commelinaceae, Eriocaulaceae, Flagellariaceae, Mayacaceae, Philydraceae, Pontederiaceae, Rapateaceae, Restionaceae, and Xyridaceae (Tables S1 and S2). This concept of Farinosae was also followed in detail by Rendle [86].

Between 1930 and 2000, several classification systems were proposed, and consequently, a significant disagreement erupted regarding the circumscription of Commelinales, as well as several other plant families and orders. Hutchinson [87] proposed the reduction of Commelinales, accepting only three families: Commelinaceae, Flagellariaceae, and Mayacaceae (Tables S1 and S2). In this circumscription, Dasypogonaceae are sunk into Xanthorrhoeaceae and placed in Agavales, and Eriocaulaceae are placed in the monofamilial Eriocaulales, while Xyridaceae and Rapateaceae are placed in Xyridales (Table S5). After a hiatus of over three decades, a myriad of classification systems and their versions were proposed by different authors (e.g., [88,89,90,91,92,93,94,95,96,97,98,99]), (Tables S1 and S2). According to the author and version of each classification system, Commelinales was composed of (Tables S1 and S2): (1) Commelinaceae and Mayacaceae [89,90,91]; (2) Commelinaceae, Mayacaceae, Rapateaceae, and Xyridaceae [88,95,96,97,100]; or (3) Commelinaceae, Eriocaulaceae, Mayacaceae, Rapateaceae, and Xyridaceae [92,93,94,95,96,98,99]. Cronquist [88,100] considered Commelinales to be closely related to Bromeliales and Liliales (the latter including Haemodoraceae, Philydraceae, and Pontederiaceae). He characterised Commelinales as possessing paracytic stomata with four or six subsidiary cells, nuclear endosperm generally starchy with some protein, and embryo capped at the micropylar end. This circumscription of Commelinales, including Commelinaceae, Mayacaceae, Rapateaceae, and Xyridaceae, was followed by Takhtajan [95,96] since it appeared to him to represent a “morphologically cohesive group”. Nonetheless, the morphological similarities between Eriocaulaceae, Mayacaceae and Xyridaceae were observed by other systems at the time [89,90,91], questioning their naturalness. The last exclusively morphological circumscription for Commelinales was proposed by Dahlgren et al. [92], which was updated by G. Dahlgren [93] and modified by Goldberg [94] and Thorne [98,99]. It was characterised by a tendency to a rosette habit, closed leaf sheaths, showy and insect-pollinated flowers, deliquescent petals (generally yellow, pink, purple or blue), and the absence of septal nectaries. Commelinales sensu Dahlgren et al. [92] included Commelinaceae, Eriocaulaceae, Mayacaceae, Rapateaceae, and Xyridaceae, being accepted by most botanists until the advent of molecular phylogenetics [3].

With the dawn of the molecular era, our knowledge and understanding of the phylogenetic relationships in plants drastically changed and increased [13,81,101,102]. Similarly, the concept of Commelinales was changed entirely, grouping families that were rarely considered to be closely related [3]. All families previously placed, at some point, in Commelinales (i.e., Anarthriaceae, Bromeliaceae, Centrolepidaceae, Cyperaceae, Eriocaulaceae, Flagellariaceae, Joinvilleaceae, Mayacaceae, Poaceae, Rapateaceae, Restionaceae, and Xyridaceae are currently placed in different lineages of Poales sensu APG; Table S4) [1,2,3,13,35,103]. The only exceptions are its type family (i.e., Commelinaceae), Iridaceae, and Dasypogonaceae. Iridaceae was only once placed in the same order as Commelinales [84], being posteriorly associated with other Lilioid Monocots in all morphological systems, and currently placed in Asparagales sensu APG [13,81,101,102]. On the other hand, Dasypogonales was generally associated with Juncaceae and/or Xyridaceae (Table S4) or sometimes sunk into Xanthorrhoeaceae and placed with other Lilioid Monocots (Table S4). Currently, it is either placed in Arecales [1,13] or in its own order, Dasypogonales (Table S4) [2,3]. Especially surprising was the placement of Mayacaceae in Poales (Table S4) [1,2,3,13,35,104] since it was always considered to be either sister to or included in Commelinaceae [105].

Commelinales sensu APG is monophyletic and composed of Commelinaceae, Haemodoraceae, Hanguanaceae, Philydraceae, and Pontederiaceae (Table S5) [1,2,3,4,5,6,7,8,9,10,11,12,13]. Out of these families, Haemodoraceae, Philydraceae, and Pontederiaceae were generally considered closely related to each other [19]. They were generally placed in (Table S5): (1) Liliales (only Pontederiaceae [84,88,94], all of them [93,94,102,103]); (2) each in its monofamilial order [95,96,98,99]; (3) Haemodorales (only Haemodoraceae and Philydraceae) [88]; (4) Bromeliales [99]; (5) Iridales (only Haemodoraceae and Philydraceae) [94]; or (6) all in Philydrales [98]. Rarely, Haemodoraceae was placed in Narcissales, while Philydraceae was placed in Xyridales [84]. On the other hand, Hanguanaceae was always considered a family of uncertain affinity [38] and has commonly been left unplaced in several classification systems (Table S5). It was historically considered to be morphologically similar to several genera and families of Monocots [37], such as Amaryllidaceae (i.e., *Crinum* L.), Arecaceae (i.e., *Chamaedorea* Liebm. ex Mart., *Pinanga* Blume, and *Morenia* Ruiz & Pav.) [106], Asparagaceae (i.e., *Dracaena* L. and *Lomandra* Labill.), Asteliaceae (i.e., *Astelia* Banks & Sol. ex R.Br.), Cyclanthaceae (i.e., *Carludovica* Ruiz & Pav.), Juncaceae, Melanthiaceae (i.e., *Veratrum* L.), Rapateaceae, and Smilacaceae (i.e., *Smilax* L.), and finally as part of the exceedingly broad concept of Liliaceae (as several other Monocot genera and families have; Table S5). Nonetheless, the most widely accepted grouping was proposed by Backer [107], in which *Hanguana* Blume was placed as a genus of Flagellariaceae (also including Joinvilleaceae). Regarding ordinal placement, Hanguanaceae has been tentatively placed in: (1) Coronariae [82,83]; (2) Juncales [84]; (3) Commelinales [85]; (4) Liliales [86,87,88,95,96,100]; (5) Asparagales [89,90,91,92,98,99]; (6) Poales (as an alternative placement) [90,91]; and (7) Hanguanales [93]. *Hanguana* has also been associated with Arales, Arecales, Asparagales, Xyridales, and Zingiberales, but has never been formally placed in these orders. The final ordinal association, based solely on morphology, was made by Rudall et al. [38]. These authors suggested an association with Zingiberales despite Hanguanceae presenting unisexual and (questionably) actinomorphic flowers, and a superior ovary, and lacking septal nectaries. The morphological hypothesis that seemed to be the least likely to be natural was the one proposed by Tillich [108] and Tillich & Sill [109]. They suggested, based on fruit, seed and seedling characters, that Hanguanaceae might be closely related to Commelinaceae, with a basal position [sic] in Commelinales. This hypothesis has been confirmed over and over and over by different molecular studies, with Hanguanaceae most commonly recovered in a sister position to Commelinaceae [1,2,3,4,5,6,7,8,9,10,11,12,13].

### 3.2. Monophyly, Synapomorphies and Characterisation of Commelinales

The monophyly and composition of Commelinales have been the focus of much debate since its recognition [1,2,3,4,5,6,7,8,9,10,11,12,13,35,38,39,40,41,42,43,44,45,55,76]. Until now, the current circumscription of Commelinales was supported exclusively by molecular data and, thus, was the cause of much criticism by traditional botanists. Despite the order being statistically robustly supported [1,2,3,4,5,6,7,8,9,10,11,12,13,35,38,39,40,41,42,43,44,45,55], its generally poorly resolved internal relationships (Figure 1) have also prevented any acceptable subdivision of the order. Out of the 34 synapomorphies recovered in the present study for Commelinales, 22 of them are clearly artefacts resulting from our sampling not including all of Angiosperms (i.e., characters 44, 101, 116, 136, 197, 234, 243, **250**, 251, **253**, 271, **354**, **366**, 387, 390, 401, **416**, 436, 440, **458**, **517**, 570, and **578**). These can be arranged into two groups, based on the reason why they are considered artefacts: (1) widely distributed in Angiosperms (i.e., characters 44—leaf-blade posture, 101—peduncle development of inflorescence secondary branches, 116—similarity between inflorescence secondary branch bracts, 136—number of flowers per inflorescence secondary branch, 197—perianth lobe posture, 234 and 243—petal blade shape, 366—pollen tectum elements rounded, 387—pistil length relative to the stamens, 401—style curvature, 436—seed dorsal side shape, 440—seed testa ornamentation, 458—seedlings with rhizoids, 517—calcium oxalate raphides in the leaf mesophyll, 570—seed coat bitegmic, and 578—copious endosperm), thus never being really expected to represent true synapomorphies; and (2) symplesiomorphies in Monocots (i.e., characters 250, 251 and 253—stamen absence, 271—filaments all equal, 354—sulcate pollen grains, 390—superior ovary, and 416—dry fruits) that are recovered as synapomorphies in the present analysis due to their actual synapomorphic state in Zingiberales (the outgroup).

In contrast, two (i.e., characters **37**—leaf-sheath suture scar, and **157**—number of septal nectaries) are the result of gaps/non-applicable data in the matrix. Additionally, character **157** also relates to the presence of septal nectaries, which is synapomorphic to Monocots, but has been lost independently countless times. This leaves only eight characters (i.e., a single non-exclusive—142; and seven exclusive—**91**, **343**, **508**, **529**, **531**, **542**, and **544**). Due to their relevance, characters 142, **343**, **529**, **531**, **542** and **544** will be discussed in detail further below, under their own subtopic. The development of synflorescence/inflorescence buds (character **91**) is a character that, based on an extensive literature review, has never been investigated in Monocots. Thus, I cannot affirm with certainty that it is indeed an exclusive synapomorphy to Commelinales and further studies on Monocot lineages are needed to investigate this character. At present, I recognise it as a putative synapomorphy for Commelinales. Stomata are incredibly variable structures, found in most land plants. Their morphology and evolution were recently reviewed for the Monocots [110], emphasising the number of neighbouring cells and the type of cell division. Nonetheless, the size, shape and general morphology of the neighbouring cells were not addressed. Thus, for the time being, stomata with terminal and lateral neighbouring cells equal in size (character **508**) are to be considered restricted to Commelinales.

### 3.3. Interfamilial Relationships in Commelinales

Following the Code [62] (Art. 3.1), five taxonomic ranks can be recognised between order and genus: (1) suborder; (2) family; (3) subfamily; (4) tribe; and (5) subtribe. Since the publication of the APG I [108], several taxonomic ranks (i.e., division, subdivision, class, subclass, and suborder) have been widely abandoned by the botanical community. However, based on the visible stability of the molecular framework for the Angiosperms in the past several decades [13,81,101,102], the need for higher classifications of important and large biological groups, it seems warranted that these ranks are recognised once again. Following this rationale, I propose the recognition of suborder Commelinineae (incl. Commelinaceae and Hanguanaceae) and suborder Pontederiineae (incl. Philydraceae, Haemodoraceae and Pontederiaceae) to formally recognise the two molecularly, morphologically and ecologically supported major clades within Commelinales.

The present study plays a very important role in understanding the phylogenetic relationships between the five families of Commelinales. First and foremost, the current interfamilial relationship of [[Hanguanaceae + Commelinaceae] + [Philydraceae [Pontederiaceae + Haemodoraceae]]] is the more consistently and well-supported hypothesis for the order (Figure 1A). So, despite the high statistical support for some alternate phylogenetic hypotheses (e.g., Figure 1E,G), the amount of morphological, ecological, and molecular data that supports the current hypothesis (Figure 1A) [2,6,10,12,39,41,44] makes it the most robust and likely to reflect the true phylogenetic relationship in Commelinales. Despite gross morphological differences, the Commelinaceae + Hanguanaceae clade (i.e., suborder Commelinineae) is supported by 30 synapomorphies (of which nine are exclusive; Figure 4; Table 1), being also extensively supported by molecular data [1,2,3,5,12,13,81,102]. Of the nine exclusive synapomorphies, only three are truly exclusive (i.e., characters **462**, **468** and **572**). Seedling morphology has only been superficially studied in the Monocots as a whole by Tillich [105]. Thus, the true systematic relevance of its morphology is still not well-understood. Nonetheless, Tillich [105,108] clearly states the uniqueness of the complete absence of chlorophyll in the cotyledons of Commelinaceae and Hanguanaceae (character **462**) and the fact that at least the first primary leaf is modified into a cataphyll (character **468**). Further studies on the seedling morphology of the Monocots are essential to investigate the putative exclusiveness of these characters and their phylogenetic relevance for the Commelinaceae + Hanguanaceae clade. Additionally, Tillich [108] highlights the peculiar seed coat structure of both families, pointing out the uniqueness of their bitegmic and sclerified structure (character **572**). Thus, this is the first study to empirically recover any kind of morphological synapomorphies for this clade. Finally, six characters require a more detailed explanation, since they represent four different types of artefacts: (1) optimisation method—characters **177** (inner perianth whorl deliquescent at post-anthesis) and **518** (calcium oxalate raphides inside canals) actually represent exclusive synapomorphies for Commelinaceae, but are recovered as synapomorphic for Commelinineae due to optimisation method; (2) non-applicable data/gaps—character **435** (seed wing morphology) represents an artefact caused by this character being extrapolated for all taxa, and it is non-applicable, since it is dependent on the presence of wing(s) in the seed; (3) coding—character **581** (chromosome number x > 19) represents a character coding artefact, since character 580 had to be split into two characters due to analysis limitations regarding the number of possible/accepted character-states); and (4) outgroup sampling—characters **470** (silica bodies in the leaves) and **540** (origin of the stamens primordia) are widely distributed in the Angiosperms and Monocots.

The [Philydraceae [Haemodoraceae + Pontederiaceae]] clade (i.e., suborder Pontederiineae) is morphologically and ecologically much more easily recognisable than the Commelinaceae + Hanguanaceae clade, having been recovered or suggested based on morphological data alone by several authors [19,108,111], and heavily supported by molecular data (Figure 1A) [2,6,10,12,39,41,44]. Of the 18 exclusive synapomorphies recovered as supporting this clade, 15 (i.e., characters **27**, **40**, **165**, **432**, **456**, **463**, **466**, **469**, **514**, **554**, **557**, **577**, **576**, **580**, **588**, and **600**) represent artefacts due to the limited outgroup. Conduplicate ptyxis (character **27**), equitant leaves (character **40**), and xylem and phloem alternate or circular phloem with central xylem or xylem abaxial (character **514**) are interconnected and widely observed in several Monocot families, including Acoraceae and Iridaceae. The fusion between both perianth whorls (character **165**) is widely distributed in the Monocots, being well known in several families of Asparagales, Liliales and Pandanales. Ovoid or fusiform to barrel-shaped seeds (character **432**), seedling primary root sinuate to spirally coiled (character **456**), cotyledon hyperphyll bifacial (character **466**), and seedling primary leaf bifacial and ribbon-like (character **469**) are widespread in aquatic plant species, representing a convergent evolution as part of the aquatic syndrome. Assimilating cotyledon (character **463**), ovules multiseriate (character **554**), 2-flanged placenta (character **557**), embryo ca. as long as the seed (character **576**), haploid chromosome number x = 7 (character **580**), and the presence of diferulic (character **588**) and p-coumaric acids (character **600**) seem to be widespread in Angiosperms and Monocots. This leaves only three (i.e., characters **471**, **520**, and **558**) putative exclusive synapomorphies for this clade. The presence of bean-shaped starch grains (character **471**) was suggested by Givnish et al. [3] as an exclusive synapomorphy for this clade. I have not been able to find any evidence that would indicate otherwise, and this is considered a putative synapomorphy for this clade. The morphology and distribution of calcium oxalate crystals in Monocots were reviewed by Prychid & Rudall [54]. They showed that styloid crystals are widespread in Monocots, but are notably absent in almost all Commelinids, except for this clade and a few records in Zingiberaceae. However, their presence in the endothecium (character **520**) seems to be exclusive to this clade [19], being a putative synapomorphy for this clade. Prychid et al. [53] investigated the systematic significance of cell inclusions in Commelinales. Still, they did not investigate the presence/absence of placental sclereids, in addition to not sampling any representatives of Hanguanaceae. Nonetheless, Simpson [19] reports the presence of placental sclereids (character **558**) for Haemodoraceae, Philydraceae, and Pontederiaceae, based on ample anatomical studies. Thus, this character is also considered another putative synapomorphy for this clade.

Finally, the Haemodoraceae + Pontederiaceae clade recovered in the present study is consistent with several morphological [19,25,48,51,52] and molecular studies [1,5,6,10,12,13,25,39,41,44,45]. Out of the 10 exclusive synapomorphies recovered in the present study, six (i.e., characters **161**, **215**, **363**, **374**, **440**, and **506**) represent artefacts due to the limited outgroup. The presence of a hypanthium (character **161**) is widespread in Angiosperms and Monocots, being well-known in many Eudicots (e.g., Fabaceae, Grossulariaceae, Lythraceae, Myrtaceae, and Rosaceae) and Monocot (e.g., Amaryllidaceae, Asparagaceae, Iridaceae, Liliaceae, Orchidaceae, and Velloziaceae) families. Nectar guides are widely distributed in Angiosperms and Monocots, also being variously coloured, sometimes even within the same family. Yellow (character **215**) seems to be the most widespread colouration of nectar guides in Angiosperms, being known in several Eudicot (e.g., Asteraceae, Brassicaceae, Fabaceae, Malvaceae, Onagraceae, Ranunculaceae, Rosaceae, and Solanaceae) and Monocot (e.g., Amaryllidaceae, Iridaceae, Liliaceae, Orchidaceae, and Zingiberaceae) families. Pollen grains lacking an infratectum (character **363**), pollen grains with a verrucate apertural membrane (character **374**), and stomata neighbouring cells with oblique division (character **506**) are also widespread in Angiosperms. Finally, seeds with testa longitudinally crested or winged (character **440**) are widespread in aquatic plant species, representing a convergent evolution as part of the aquatic syndrome. This leaves only four (i.e., characters **359**, **364**, **573**, and **590**) potentially exclusive characters. The palynological characters reported by Simpson [19,46,51], i.e., pollen inner layer papillate or baculate (character **359**) and tectum baculate (character **364**), are confirmed to be exclusive to the Haemodoraceae + Pontederiaceae clade. The present analysis also recovers the seed coat with calcium oxalate (character **573**) as an exclusive synapomorphy for this clade. Nonetheless, studies focusing on the macro- and micromorphology of Monocot seeds are sparse and further studies are much needed to test the exclusivity of this feature. Finally, the presence of phenylphenalenones (character **590**) was originally regarded by Simpson [19] as a synapomorphy for Haemodoraceae. Nonetheless, further phytotaxonomic studies have shown phenylphenalenones to be also present in Pontederiaceae and the distantly related Musaceae and Strelitziaceae [112,113]. Thus, this character should be regarded as a non-exclusive synapomorphy for the Haemodoraceae + Pontederiaceae clade.

### 3.4. Familial Circumscription in Commelinales

Regarding the five currently recognised families of Commelinales (i.e., Commelinaceae, Hanguanaceae, Philydraceae, Haemodoraceae, and Pontederiaceae), they have been consistently recovered as monophyletic based on molecular data [1,2,3,4,5,6,7,8,9,10,11,12,13,25,35,39,41,42,43,44,45,55,65,66,67,101,102]. However, morphological investigations on their monophyly, and thus their potential synapomorphies, have been limited to Haemodoraceae [19] and Pontederiaceae [25]. Commelinaceae also has a morphology-based phylogenetic hypothesis available [114]. However, the dataset is very small, sampling is suboptimal, and the dataset is not very congruent with the molecular dataset [115]. Finally, the authors failed to discuss or propose putative synapomorphies for the family. Thus, this study represents the first attempt to empirically investigate the monophyly and circumscription of Commelinaceae, Hanguanaceae, and Philydraceae.

#### 3.4.1. Philydraceae

Out of the 22 synapomorphies supporting the monophyly of Philydraceae, 10 of them clearly represent artefacts. These can be arranged into two groups, based on the reason why they are considered artefacts: (1) sampling—widely distributed in Monocots and/or Angiosperms, thus resulting from our limited outgroup sampling (i.e., characters 41—equitant leaf distribution, 272—antepetalous stamens longer than the antesepalous, 344—pollen yellow to orange in vivo, 394—ovary 1-locular, 436—seeds dorsally flattened, 548—ovary aposeptalous, 555—placentation intrusive-parietal, and 580—haploid chromosome count x = 8) and (2) gaps/non-applicable data—characters that are dependent on another, causing the optimisation to infer their condition for all taxa (i.e., characters 428—fleshy fruit colouration when mature and **443**—dactyliform projections arranged along longitudinal striations). This leaves 12 characters, three exclusive (i.e., characters **184**, **445**, and **543**) and nine non-exclusive (i.e., characters 127, 164, 251, 252, 253, 257, 261, 535, and 542) as potential synapomorphies.

The non-exclusive synapomorphies (i.e., characters 127, 164, 251, 252, 253, 257, 261, 535, and 542) merit only brief discussion. Spathaceous bracteoles equal to the cincinnus bracts (character 127) are an uncommon but widely distributed character in Angiosperms. More importantly, it is widespread in Commelinales, also being known in *Pyrrorhiza* (Haemodoraceae), with spathaceous bracteoles but distinct from the cincinnus bracts known in *Cubanicula*, *Tribonanthes* and *Xiphidium* (Haemodoraceae), and spathaceous and generally leaf-like known for *Amischotolype* s.lat., *Coleotrype* s.str., *Nivoanthus* (i.e., the Malagasy *Coleotrype*), and *Cyanotis* s.lat. (Commelinaceae). A campanulate perianth (character 164) is also a widely distributed character in Angiosperms, also being known in Commelinales in *Cubanicula*, *Pyrrorhiza* and *Xiphidium* (Haemodoraceae), and *Pontederia* subg. *Monochoria* (Pontederiaceae). When analysed separately, characters 251, 252 and 253 are clearly non-exclusive, being widespread in Angiosperms and Commelinales. However, when analysed together, they do represent an exclusive synapomorphy for Philydraceae. These characters refer to the absence of the three antesepalous (characters 251 and 252) and the inner antepetalous (character 253) stamens. Flowers with only the outer antesepalous stamen are exclusive to Philydraceae, with the few Monocot families with a single stamen (i.e., monandrous flowers) achieving this in very different ways: (1) Alismatales (e.g., Araceae, Hydrocharitaceae, Potamogetonaceae, and Zosteraceae) have truly dimerous flowers, with the few cases of monomerous flowers being ontogenetically different, in addition to several pseudomonomerous taxa; (2) Orchidaceae (i.e., subfamilies Vanilloideae, Orchidoideae, and Epidendroideae) have three stamens, with two of them highly modified into petaloid staminodes variously fused to the gynostemium; and (3) core Zingiberales (i.e., Cannaceae, Costaceae, Marantaceae, and Zingiberaceae) have one or a half fertile stamen, where the other stamens or partial stamen are variously modified into petaloid staminodes. Similar to the previous characters, filament fused to the petals/inner tepals (character 257) is recovered as non-exclusive, due to the way it was coded. However, the specific state of Philydraceae is unique in Angiosperms, as well, since the upper perianth lobe is a labellum-like structure of mixed origin (see below discussion for character **184**) and the single stamen is only fused with the two inner lobes (i.e., outer petals). Flattened filaments (character 261) are symplesiomorphic for Angiosperm, being almost omnipresent in members of the ANA Grade (i.e., Amborellaceae, Nymphaeaceae, Austrobaileyaceae, Schisandraceae, and Trimeniaceae), Magnoliales, and Laurales. However, they are rather uncommon in the Monocots and, thus, their occurrence in Philydraceae does stand out. As aforementioned (see Monophyly, synapomorphies and characterisation of Commelinales), perianth tannins are considered synapomorphic for Commelinales. However, their being densely distributed throughout the perianth (character 535) seems to have evolved independently within the order, in both Philydraceae and *Pontederia* (Pontederiaceae). Finally, the not basally thickened endothecium (character 542) represents a reversion.

Regarding the exclusive synapomorphies, these represent very obviously unique characters for the family. The pseudotetramerous perianth (character **184**) is truly unique among the Angiosperms. Other types of perianth fusion and/or specialisations are known to occur in several Monocots, with labellum-like structures also known for Orchidaceae, Corsiaceae, and all families of Zingiberales. Nonetheless, their origin differs among all families, resulting from conspicuously different-shaped perianth lobes (i.e., Orchidaceae, Corsiaceae, Lowiaceae, and Strelitziaceae), the fusion of different perianth lobes (i.e., Philydraceae, Heliconiaceae, Musaceae, and Strelitziaceae), or petaloid staminodes (i.e., Cannaceae, Costaceae, Marantaceae, and Zingiberaceae). Even when considering only the families where the labellum-like structure is the result of the fusion of different perianth lobes, their ontogenetic origin remains distinct, with Philydraceae being the result of the fusion between the two inner sepals and the outer petal, Heliconiaceae being the result of the fusion between the two inner sepals and all sepals, Musaceae being the result of the fusion between all sepals and the inner petals, and Strelitziaceae being the result of the fusion between the two inner petals. Thus, the pseudotetramerous perianth of Philydraceae, resulting from its unique labellum-like structure, is unique in the Angiosperms and represents an exclusive synapomorphy for the family. The enlarged chalazal cap of Philydraceae (character **445**) is also unique among Angiosperms, due to a combination of associated features, such as the enlarged cap obscuring the funiculum, being conspicuously discolourous from the rest of the seed, and lipid-rich, enabling seed floatation and water dispersal. Thus, the enlarged chalazal cap is here confirmed as an exclusive synapomorphy for Philydraceae. Another chalazal character, unfortunately, not included in the present study, is the development of a chalazal haustorium during germination, which is suggested by Stevens [15] as synapomorphic for the family. However, this character is widespread in Angiosperms and should be treated as a non-exclusive synapomorphy for Philydraceae. Finally, the glandular tapetum of Philydraceae (character **543**) seems to represent a reversal synapomorphic to this family. Glandular (or secretory) tapetum is symplesiomorphic Tracheophyta [15,116], but was lost several times inside the Monocots, with an amoeboid (or plasmodial) tapetum being synapomorphic for Commelinales and invasive (or non-syncytial) tapetum synapomorphic for the [[Costaceae + Zingiberaceae] [Cannaceae + Marantaceae]] clade of Zingiberales, while the remaining families of Zingiberales (i.e., Heliconiaceae, Lowiaceae, Musaceae and Strelitziaceae) have the symplesiomorphic glandular tapetum ([116,117]; this study).

#### 3.4.2. Pontederiaceae

As aforementioned, synapomorphies for Pontederiaceae have already been previously investigated and discussed by Pellegrini et al. [25]. Out of the 56 synapomorphies recovered in the present study, 18 (i.e., characters **26**—leaves dimorphic, **27**—ptyxis conduplicate-involute, **28**—leaves late bifacial, 29—ligule present, 40—immature leaves distichously-alternate, 83—inflorescence deflexed at post-anthesis and in fruit, 166—perianth whorls fused into a conspicuous tube, 180—flowers blue/lilac/purple, 212—nectar guide present, **213**—nectar guide on the posterior lobes, **355**—pollen grains bisulcate, **411**—superior ovary anthocarp, **514**—xylem abaxial and phloem adaxial near the margin of the blades plus xylem and phloem alternate near the centre of the blade, 521—mesophyll aerenchymatous, 527—receptacle aerenchymatous, 534—perianth lacking fibrillar tannins, **536**—perianth aerenchymatous, and **547**—ovary walls aerenchymatous) have been previously discussed by Pellegrini et al. (2018) [25] and will not be discussed in the present study. Out of the remaining 38 synapomorphies, 14 of them (i.e., 1—plants annual, 44—immature leaves patent to slightly recurved, 59—immature blades apex round to obtuse, 101 & 102—secondary branches long-pedunculate, 136—secondary branches 1-flowered, 198—outer sepal longer than the laterals, 204—persistent perianth longer than the fruit, 234—paired petals spatulate to obovate, and 274–277—filaments curved, and 548—ovary hemiseptalous) represent artefacts resulting from the limited outgroup sampling not including all Angiosperms. These characters are all widespread in Angiosperms, as well as Monocots. This leaves 24 undiscussed synapomorphies (i.e., characters 41, **63**, 84, 86, 90, 106, 111, 127, 158, 279, 281, 284, 286, 287, 290, 292, 293, 295, 298, **403**, **465**, 535, 546, and 552).

Out of the non-exclusive synapomorphies, the submerged immature leaves (character 42) are related to the strongly aquatic life form of the family, and can be observed in almost all lineages with aquatic species among the Angiosperms. Spathaceous (character 84) and connate basal bracts (character 86), accessory bracts absent (character 90), secondary branch axis contracted (character 106), and secondary branch ebracteate (character 111) are widespread in Commelinales, having evolved independently within its other four families. Bracteoles absent (character 127), interlocular septal nectaries (character 158) and perianth with moderately distributed tannin cells (character 535) are interesting cases, since they are almost omnipresent in Pontederiaceae and, thus, characterise the family but are also recorded for other members of the Commelinales (i.e., character 127—*Cochliostema*, *Commelina*, *Plowmanianthus*, *Siderasis* and *Spatholirion* in Commelinaceae; character 158—*Anigozanthos* s.lat., *Haemodorum* and *Lachnanthes* in Haemodoraceae; character 535—across Commelinaceae). Tannin absent in the ovary walls (character 546) and ovary septae (character 552) represent reversions to two synapomorphies for Pontederiineae. Filaments pubescent (characters 279, 284, 290 and 295) with glandular macrohairs (characters 281, 286 and 292) at the apex (characters 287, 293 and 298) stand out since filament pubescence is only otherwise recorded in Commelinales in Commelinaceae (i.e., subfamily Commelinoideae).

Of the exclusive synapomorphies, mature (i.e., petiolate) leaves being always produced (character **63**) is obviously related to the family’s synapomorphic presence of leaf dimorphism (character **26**). Interestingly enough, this character is posteriorly independently lost in two lineages of *Heteranthera* (Figure 6). Pubescent styles are uncommon in Commelinales, being recorded only for Commelinaceae and Pontederiaceae. However, in Commelinaceae, hairs are mostly restricted to the style base, being only apical within *Cyanotis* s.lat. On the other hand, stylar hairs in Pontederiaceae are either evenly distributed along the style or restricted to the apex. Regardless, glandular-pubescent styles (character **403**) are exclusive in the Commelinales + Zingiberales clade to Pontederiaceae, and represent a true exclusive synapomorphy for the family. Finally, the ligulate cotyledonary sheath (character **465**) was first reported by Tillich (1995) [105] in his overview of seedling characters in the Monocots. However, seedling characters are understudied and should only be treated as putative exclusive synapomorphies until further studies are carried out.

#### 3.4.3. Haemodoraceae

Only three (i.e., characters **95**, **390**, and 431) of the 27 currently recovered synapomorphies for Haemodoraceae were also recovered and discussed by Simpson [19]. Secondary inflorescence branches consisting of branched (i.e. bifurcate or trifurcate) cymes (character **95**) are confirmed as exclusive to Haemodoraceae. The inferior ovary of Haemodoraceae (character 390) is reported by Simpson [19], but is further investigated by Simpson [49], who confirms its distinct ontogenetic origin. The present greatly expanded matrix (both regarding sampling and characters) was the perfect opportunity to further test Simpson’s [19,20] homology hypothesis for the inferior ovary of Haemodoraceae. Unsurprisingly, I have also recovered it as synapomorphic for the family, supporting Simpson’s [19,20] hypothesis that the inferior ovary is, in fact, not homologous to the inferior ovary of other Monocots (e.g., the outgroup, Zingiberales). Thus, the inferior ovary of Haemodoraceae is treated here as “late-inferior” and coded a posteriori as a third character state (**390:3**) as a truly exclusive synapomorphic for the family. Seeds wider than long or as wide as long (character 431) were coded by Simpson [19] as “seeds discoid, flattened, or ovoid-globose, i.e., not ellipsoid and ridged” for their character 49. However, I have chosen to break this character up, since it conflated several other characters (e.g., characters 432—seed outline, 433—seed compression, 436—seed dorsal side shape, 437—seed ventral side shape, 439—seed lateral side cleft, and 440—testa ornamentation). This is an interesting character as it relates to the aquatic life form, with longer than wide seeds being more commonly observed in water-dispersed seeds, as observed in several aquatic Commelinales (e.g., all Philydraceae and almost all species of Pontederiaceae) and other distantly related aquatic Angiosperms (e.g., almost all Nymphaeales, Eriocaulaceae, Rapateaceae and Xyridaceae, all Typhaceae, etc.).

Of the remaining 24 synapomorphies, filaments densely pubescent (characters 280, 285, 291 and 296) represent optimisation artefacts, caused by the analysis extrapolating their coding since all species of Haemodoraceae have glabrous filaments. Thus, all characters associated with the presence of filament hairs (i.e., characters 280–283, 285–289, 291–294, and 296–300) recovered as any kind of synapomorphy in this family are, by definition, artefacts, since they are non-applicable for all its species. Out of the remaining 20 synapomorphies, 14 are non-exclusive (i.e., characters 11, 201, 202, 208, 215, 271, 458, 466, 477, 483, 491, and 557) with six being exclusive (i.e., characters **12**, **47**, **216**, **469**, **474**, and **587**). Perianth whorls equal or subequal to each other (character 201), outer perianth whorl succulent or fleshy (character 202), perianth lobes equal or subequal between whorls (character 208), inner whorl filaments longer than outer ones (character 271), and unflanged placenta (character 557) are widely distributed characters among Angiosperms, but more importantly, the Monocots. The loss of uniseriate macrohairs (character 483) seems to be correlated with the evolution of multiseriate tapering hairs (character 491) in Haemodoraceae. Interestingly, despite these being recorded in the present dataset exclusively for taxa within Haemodoraceae, the optimisation recovers it as a non-exclusive synapomorphy for the family. However, I argue that this specific hair type does represent a true exclusive synapomorphy for Haemodoraceae.

The following synapomorphies form groups of associated characters and will be discussed in that way. Sand-binding roots (character 11) and the presence of arachnoid hairs in the roots (character **12**) are clearly associated, even though sand-binding roots are widely recorded within Commelinales (e.g., Commelinaceae and Philydraceae) and throughout the Monocots. However, the arachnoid root hairs reported by Smith et al. [67] seem to be exclusive to Haemodoraceae, and are here treated as a putative exclusive synapomorphy to Haemodoraceae. Another characteristic feature of Haemodoraceae is the very sclerified nature of its vegetative organs, with leaf-blade fibrous and coriaceous (character **47**), root pith sclerified (character **474**), and vascular bundles in the stems with a fibrous layer (character 477). The latter, vascular bundles in the stems with a fibrous layer (character 477), is recovered as non-exclusive in this study due to its known occurrence in *Tradescantia zanonia* (L.) Sw. It is very likely that this feature is also shared with other members of Commelinales (e.g., *Hanguana*, *Helmholtzia*, *Orthotylax*, *Cartonema*, large-sized *Palisota*, etc.). The presence of yellow nectar guides is synapomorphic for the Haemodoraceae + Pontederiaceae clade. However, in Haemodoraceae, orange to red nectar guides (character 215) are recovered as synapomorphic, with the guide consisting of three distinct spots (character **216**) being exclusive. As aforementioned, nectar guide colouration and morphology are not thoroughly documented in Angiosperms, making it difficult to assess how exclusive this character-state truly is. Given that phenylphenalenones (character **590**), which are synapomorphic for this clade and very rarely reported in plants, are responsible for the orange to red colouration, it seems these nectar spots represent a strong candidate for putative exclusive synapomorphy for Haemodoraceae. Some seedling characters have already been discussed for other clades in the phylogeny, which has highlighted how poorly investigated these are in Angiosperms. The presence of rhizoids (character 458), a cylindrical cotyledon hyperphyll (character 466), and seedlings with unifacial primary leaves (character **469**) seems to be widely distributed in the Monocots and, thus, represents artefacts resulting from the limited outgroup sampling in the present study. Finally, the presence of chelidonic (character **587**) acid is widespread in the Monocots, but conspicuously absent in the Commelinids, with the only exception being Haemodoraceae. This makes their occurrence in Haemodoraceae a very peculiar independent origin of this compound, and an exclusive synapomorphy for the family.

#### 3.4.4. Hanguanaceae

Of the 43 synapomorphies recovered for Hanguanaceae, 26 of them are widely distributed in the Monocots. Fibrous stems (character 20), leaf sheaths with scarious margins (character **36**), two to several secondary branches per inflorescence node (character 99), flowers clustered (character 137), perianth homochlamydeous (character 167), inner perianth whorl sepaloid (character **169**), perianth remaining herbaceous at post-anthesis (character **177**), perianth lobes erect (character 197), sepals equal to subequal (character 201), outer perianth whorl herbaceous (character 202), medial and lateral sepals rhomboid to orbicular (characters 224 & 225), petals green (characters 237 & 246), stamens dimorphic (character 249), anther latrorse (character 337), pollen inaperturate (character 354), pollen grains with tectal elements acute (character 366), ovary locules 1-ovulate (characters 395 & 396), fruits fleshy (character 416), subglobose to globose (character 417), lustrous (character 424) and indehiscent (character 425), seed testa smooth (character 440), and seeds tenuinucellate (character **579**) are not only widespread in the Monocots but, more precisely, shared with members of Arecales, Dasypogonales, Pandanales and some Asparagales. These shared features could help explain Hanguanaceae having been previously associated with these orders and their families [38].

Rudall et al. [38] proposed Hanguanaceae as sister to Zingiberales based on spinulose and inaperturate pollen, plasmodial tapetum, mucilage-secreting intraovarian trichomes, and modified septal nectaries. Nonetheless, the pollen in Zingiberales is more correctly classified as ulcerate, due to their huge and more-or-less circular apertures with irregular margins. At the same time, spinulose exine is widely distributed not only in the Monocots, but also in the Angiosperms as a whole. The plasmodial tapetum was recovered by Furness and Rudall [116,117] as synapomorphic for Commelinales, albeit they excluded Hanguanaceae from the order based on the assumptions of Rudall et al. [38], with Zingiberales most likely possessing an ancestrally glandular tapetum ([116,117], this study). The ovarian mucilage-secreting colleter hairs (character **553**) are also reported for *Astelia* Banks & Sol. ex R.Br. (Asteliaceae, Asparagales), in addition to Hanguanaceae and Zingiberales. However, Rudall et al. [38] failed to investigate and compare the morphology of these hairs in said groups, rushing to assume the homology of these hairs between Hanguanaceae and Zingiberales. These hairs are conservatively considered to be ontogenetically distinct and, thus, an exclusive synapomorphy for Hanguanaceae and not a shared character between the family and Zingiberales. Finally, Rudall et al. [38] mention the presence of modified septal nectaries in Hanguanaceae. However, this was yet another rushed assumption, based on the presence of nectariferous staminal scales (character **375**) and nectariferous pistilode lobes (character **386**). Based on their own analysis, the nectariferous scales are very obviously not homologous to septal nectaries and represent an exclusive synapomorphy for Hanguanaceae. In contrast, the nectariferous pistilode lobes could indeed be homologous, but developmental studies are necessary to confirm this hypothesis. Regardless, Rudall et al. [38] also ignore the fact that septal nectaries are also present in Commelinales (i.e., Haemodoraceae and Pontederiaceae). Thus, based on the present morphological data and analysis, but also in combination with all available molecular data, I refute the hypotheses by Rudall et al. [38] that the nectariferous staminal scales and nectariferous pistilode lobes are homologous to the septal nectaries of Zingiberales, but also that Hanguanaceae is best positioned inside Zingiberales.

Out of the remaining 19 synapomorphies, six of them are recovered as non-exclusive. Subpetiolate leaves (character 43) are peculiar in that their leaf blades are conspicuously narrowed at the base, which is superficially similar but serves the same purpose of enabling leaf-blade movement. Subpetiolate leaves were suggested by Givnish et al. [3] to be synapomorphic to the Commelinaceae + Hanguanaceae clade. However, subpetiolate leaves seem to have evolved several times independently within Commelinales, being very common in Commelinaceae subfamily Commelinoideae, but conspicuously absent from subfamily Cartonematoideae. Subpetiolate leaves are also observed in *Wachendorfia* s.lat., despite clearly being ontogenetically distinct. Additionally, subpetiolate leaves are also sparsely widespread in the Monocots, being recorded for families such as Amaryllidaceae, Costaceae, Cyperaceae, Flagellariaceae, Hypoxidaceae, Poaceae, and Velloziaceae. The seeds of Hanguanaceae are very peculiar due to their bowl shape, which is complemented by the ventral side being concave to wedge-shaped (character 437). However, the concave ventral side is also shared with *Triceratella* (Commelinaceae), serving as a potential morphological link between both families. Associated with the bowl-shaped seeds of Hanguanaceae is the blanket-like placenta (character **556**), which completely fills the concave to wedge-shaped cavity on the ventral side of the seed. The loss of uniseriate macrohairs (character 483) in Hanguanaceae is relevant, as these hairs are almost omnipresent in Commelinales, being also independently lost in Haemodoraceae. These are replaced in Hanguanaceae by multiseriate fruticose hairs (character **493**) and by several other types of multiseriate hairs in Haemodoraceae (characters 491, 492, 494, 495, and 496). The loss of calcium oxalate raphides in their leaf’s mesophyll (character 517) is conspicuous in Hanguanaceae, as it is consistently present in other members of Commelinales. However, the family has been very scarcely anatomically investigated, which could mean that these crystals have just been overlooked, instead of being actually absent. Thus, I do not consider it a potential synapomorphy for the family, as I believe further studies might reveal their presence in Hanguanaceae. The presence of tapetal raphides (character 544) is suggested in the present study as a synapomorphy for Commelinales. However, these have not been confirmed for Hanguanaceae, and are coded in the present study as “?” (i.e., missing data). However, the optimisation analysis extrapolates their absence in the family, which represents an analysis artefact.

The remaining six synapomorphies are recovered as potentially exclusive to Hanguanaceae. Dioecy (character **4**) has independently evolved a few times in the Angiosperms, representing a rather uncommon character [118]. In the Monocots, dioecy is reported for Alismatales (i.e., Cymodoceaceae, Hydrocharitaceae, Posidoniaceae, and Zosteraceae), Asparagales (i.e., several genera of Asparagaceae), Dioscoreales (i.e., Dioscoreaceae), Liliales (i.e., Smilacaceae), Pandanales (i.e., Pandanaceae), Arecales (i.e., several genera of Arecaceae), and Poales (i.e., *Hechtia* Klotzsch in Bromeliaceae and Restionaceae), in addition to Hanguanaceae. The overall distribution of this character in the Angiosperms, but more specifically in the Monocots, suggests it indeed evolved independently in more distantly related lineages and, thus, should be treated as an exclusive synapomorphy for Hanguanaceae. The loss of the style in (character **397**) Hanguanaceae is an interesting case. The pistillate flowers on Hanguanaceae seem to truly lack a style, while the staminate ones have a short and stout style. Because of this variation between flower morphs, I have chosen to code the species as lacking a style, based on the pistillate flower, since it is a gynoecium/pistil character. This directly causes it to be recovered as a synapomorphy for the family, since this character is not observed in any other taxon in the morphological dataset. Additionally, flowers with sessile stigmas (or lacking a style) are interestingly shared between Arecales (i.e., Arecaceae) and Pandanales (i.e., Cyclanthaceae and Pandanaceae), which were suggested as possibly being closely related to Hanguanaceae, in addition to Alismatales (i.e., almost all families) and Poales (e.g., Cyperaceae and Poaceae).

The seed coat of both Commelinaceae and Hanguanaceae is bitegmic, with the outer layer sloughing off in Commelinaceae but persisting in Hanguanaceae. Additionally, in Hanguanaceae, the seed coat presents two layers of crossing fibres (character **574**), which is otherwise unknown in the Angiosperms. Tillich [108] studies the seed and seedling morphology and anatomy for Commelinaceae and Hanguanaceae. The author describes the peculiarity of the primary roots of Hanguanaceae seedlings being brown (character **457**). However, the author does not provide further information on the distribution or rarity of this character in Angiosperms or even the Monocots. Thus, because seedling characters have been poorly studied, the brown primary roots are tentatively treated as an exclusive synapomorphy for Hanguanaceae. As previously discussed (see the Philydraceae circumscription discussion above), the medially thickened endothecium (character **542**) is considered exclusive to Hanguanaceae. Finally, the haploid chromosome count x = ca. 85 (character **581**) is also tentatively considered an exclusive synapomorphy for Hanguanaceae. The reason being that no reliable chromosome counts have ever been produced for Hanguanaceae, with the approximated ca. 85 being, unfortunately, the best one. Detailed cytological studies in Hanguanaceae are drastically needed to test this synapomorphy hypothesis.

#### 3.4.5. Commelinaceae

Commelinaceae has been consistently considered to be monophyletic, based on both morphological and molecular data, and a morphologically cohesive family. Paradoxically, all previously phylogenetic studies for the family [44,114,115,119,120,121,122,123,124] have failed to propose or recover a single morphological synapomorphy for the family. The present study recovers 25 synapomorphies for Commelinaceae, seven of which are anatomical (characters 472, **475**, **518**, 535, **571**, **573**, and **575**). This greatly supports the importance of defining past anatomical studies [53,105,125,126,127,128,129,130,131,132,133,134,135,136,137,138,139]. The presence of vessels in both the roots and stems (character 472) seems to be related to the consistently less aquatic life form of Commelinaceae (and Haemodoraceae), when compared to the other families. The presence of a nodal vascular plexus in the stems of Commelinaceae (character **475**) was investigated in detail for the family by Vita et al. [138]. However, due to the complexity of such a study, it was limited to Commelinaceae and did not investigate any of the other four families in the order. A nodal vascular plexus has not been reported for Haemodoraceae, Hanguanaceae, Philydraceae and Pontederiaceae in the available literature. Nonetheless, further studies focusing on this character are imperative in Commelinales to investigate its potential distribution and evolution in the order. Thus, for the time being, the presence of a cauline nodal vascular plexus is treated as a putative synapomorphy for Commelinales. Raphide canals throughout the mesophyll (character **518**) are widely reported for subfamily Commelinoideae, but are exclusively found along the veins of the leaves of *Triceratella* (subfamily Cartonematoideae). Nonetheless, these are completely absent from the leaves of *Cartonema* (subfamily Cartonematoideae). The present analysis reconstructs these canals as having evolved a single time in the family, being posteriorly lost in *Cartonema* (a synapomorphic reversion), and their distribution restricted in *Triceratella* to the mesophyll along the veins. Thus, these are confirmed as synapomorphic to Commelinaceae. The moderate presence of tannin cells in the perianth (character 535) is shared with Pontederiaceae and hypothesized here to have evolved independently in both families. However, as studies on the presence, distribution, morphology and ontogeny of tannin cells are very few in Monocots (see floral anatomy and its systematic and phylogenetic importance in Commelinales below), I refrain from making further phylogenetic assessments at this time. As aforementioned, Hanguanaceae and Commelinaceae share a very characteristic type of bitegmic seed coat. In Hanguanaceae, each seed tegmen presents a layer of crossing fibres (character **574**). Alternatively, in Commelinaceae, the outer tegmen is ephemeral and thin, sloughing off (character **571**) during development and maturation, with the inner tegmen also presenting silica crystals (character **573**). These two characters seem to be unique in the Angiosperms and are, thus, considered synapomorphic for Commelinaceae. The final anatomical synapomorphy for Commelinaceae (i.e., embryo of the Xyris-Scirpus or grass type; character **575**) represents a reversion, with this character being widespread throughout the Monocots.

Of the remaining 18 synapomorphies, two stand out as being related to seedling morphology (characters 459 and 465). The presence of a seedling collar (character 459) and a coleoptile (character 465) is widespread in the Monocots, even based on the very limited data available on seedling morphology. However, as aforementioned, further studies on this topic are much needed. The distribution and specific structure of anthocyanins have been surprisingly poorly studied in Monocots [140,141,142]. However, Stirton & Harborne [140] reported that Commelinaceae and two members of Zingiberales (Marantaceae and Zingiberaceae) shared some anthocyanin patterns, such as the complete absence of pelargonins and high concentrations of cyanidins and delphinines (the latter apparently absent in Zingiberaceae). Additionally, these authors reported that acylation is very common in Commelinaceae, while being absent or rare in all other investigated Monocot families, with Commelinaceae being the only family to present anthocyanins with 3,7,3′-triglycosides. Thus, the presence of acylated cyanidin 3,7,3′-triglycoside anthocyanins (character **586**) is considered here as a putative synapomorphy for Commelinaceae.

The final 15 synapomorphies are macromorphological and also aid in the field recognition of Commelinaceae. Of these, a handful represent overall trends and are reconstructed in the present analysis as potentially ancestral in the family. Thus, the leaves spirally alternate or arranged into rosettes (character 40), leaf blades membranous (character 47) and adaxially variegated with brown to vinaceous blotches (character 61), floral buds ovoid (character 139), perianth lobes equal or subequal in the same whorl (character 208), and sepals free (character 217) are widespread in the family and uncommon in the other members of Commelinales. However, they are by no means consistently present in Commelinales, and cannot be reliably used to characterise or circumscribe the family. Thus, they are considered non-exclusive synapomorphies for the family. The thin filaments (character 260) and the outer antesepalous filament pubescent (character 279) are similar in that they are not consistently observed in Commelinaceae. However, these are far less common in the other families of Commelinales and, thus, more reliable in the characterisation of Commelinales. Thin filaments are widespread in the Angiosperms and several lineages of Monocots. However, they are far less common in the Monocots than in the Eudicots. Thus, their wide and consistent occurrence in Commelinaceae does indeed stand out. Filament pubescence is restricted in Commelinales to Commelinaceae and Pontederiaceae. These are recovered as ancestrally glandular-pubescent in Pontederiaceae, a state that is very rare in Commelinaceae. Sand-binding roots (character 11) have already been discussed under the Haemodoraceae. Deliquescent perianth at post-anthesis is a rather rare character in the Angiosperms, being recorded for the Magnoliids (e.g., Aristolochiaceae), Monocots (e.g., Afrothismiaceae, Alismataceae, Amaryllidaceae, Asparagaceae, Butomaceae, Commelinaceae, Hydrocharitaceae, Iridaceae, Pontederiaceae, and Thismiaceae), and Eudicots (e.g., Aizoaceae, Amaranthaceae, Anacampserotaceae, Barbeuiaceae, Basellaceae, Cactaceae, Corbichoniaceae, Didiereaceae, Gisekiaceae, Halophytaceae, Kewaceae, Lophiocarpaceae, Limeaceae, Molluginaceae, Montiaceae, Phytolaccaceae, Portulacaceae, and Talinaceae). In the Magnoliids and Eudicots, a deliquescent perianth is restricted to monophyletic lineages, while in the Monocots, it seems to be more widespread and most likely evolved several times independently. In Alismatales and Dioscoreales, a single origin is more likely within each order, while in Asparagales, it could have evolved once and lost several times (i.e., Xanthorrhoeaceae and Xeronemataceae, and several times within Asparagaceae) or have evolved independently a few to many times. In Commelinales, deliquescent perianth is widely distributed in Commelinaceae and in *Pontederia* (Pontederiaceae). Nonetheless, even when comparing it to most Monocots, the deliquescence of Commelinaceae is only similar to that of Alismatales, in which only the inner perianth whorl (i.e., the petals) deliquesces at post-anthesis (character **177**). Thus, based on the distant relationship between Commelinaceae and Alismatales, petals deliquescing at post-anthesis should be considered an exclusive synapomorphy for the family.

The final set of four synapomorphies is interesting in that they relate to node, internode and leaf-sheath characters. The swollen nodes of Commelinaceae (character **24**) are widely known and used as a diagnostic feature of the family. Similarly, swollen nodes are only observed in the more distantly related Poaceae, which interestingly also share the presence of a leaf-opposed line of uniseriate hairs (character **23**) with Commelinaceae. I am unaware of these characters being reported in other Monocot families, and thus, they should be considered to have evolved independently in both families and as exclusive synapomorphies for Commelinaceae (and in turn, also for Poaceae). The asymmetric leaf sheaths of Commelinaceae are similar to those of Poaceae, Costaceae and Zingiberaceae, in that they are long, tubular and asymmetric. However, only in Costaceae are they closed (i.e., fused) and thus equal to the ones in Commelinaceae. Nonetheless, the leaf sheaths of Commelinaceae and Costaceae still differ in a key aspect, which relates to the presence of an obvious suture scar opposite the blade. Thus, despite their superficial similarities, the leaf sheaths of Commelinaceae and Costaceae are in fact distinct, and closed and asymmetric leaf sheaths with a conspicuous suture scar (characters 35 and **37**) are to be considered as exclusive synapomorphies of Commelinaceae.

### 3.5. Infrafamilial Classification of Commelinales

Due to the reduced size of Hanguanaceae, Philydraceae and Pontederiaceae, the recognition of any taxonomic ranks between family and genus (i.e., subfamily, tribe and subtribe) is not warranted. Names in these ranks have been published in the past for some of these families, but are treated as synonyms in the present study (see Supplementary List S1). Alternatively, Commelinaceae and Haemodoraceae are much larger families, and names in these ranks have been more consistently recognised throughout the years. These ranks are discussed here for both families, especially regarding their monophyly and molecular and morphological support.

#### 3.5.1. Haemodoraceae

The present topology for Haemodoraceae is greatly consistent with previous phylogenetic hypotheses for the family, based on either morphological [19] or molecular data [39,44,65,66,67]. The primary subdivision of Haemodoraceae into two subfamilies (i.e., Conostylidoideae and Haemodoroideae) has been consistently supported by all previous studies and is further supported here. The smaller Haemodoroideae is recovered in the present study, arranged into three main lineages, representing tribes Haemodoreae, Wachendorfieae and Xiphidieae. Hopper et al. [66] propose a broadly circumscribed Wachendorfieae, including Xiphidieae. However, based on the accumulated data and the reciprocal monophyly, the many recovered synapomorphies, and the statistical support for both tribes, the recognition of Xiphidieae as distinct from Wachendorfieae is warranted. If recognised in their narrower sense, both tribes are more morphologically cohesive and easily distinguishable, on top of being more biogeographically cohesive, with Xiphidieae restricted to the Neotropics, and Wachendorfieae being Afro-American. Haemodoreae is circumscribed here the same way as proposed by Hopper et al. [66]. In Conostylidoideae, the position of Phlebocarya is inconsistent, being either recovered as sister to *Conostylis* s.lat. or [*Anigozanthos* s.lat. + *Conostylis* s.lat.], depending on the analysis. In the present study, this situation repeats itself, and I consider that the recognition of a monogeneric Phlebocaryeae is both unnecessary and potentially jeopardises the monophyly of a more narrowly circumscribed Conostylideae. Thus, I propose the recognition of two tribes within Conostylidoideae: (1) Tribonantheae and (2) Conostylideae. The minor amendments to Hopper et al. [66] proposed here highlight the importance of phylogenetic studies (both morphological and molecular) in ensuring the taxonomic and systematic stability of classification systems.

#### 3.5.2. Commelinaceae

Commelinaceae has recently shifted from a taxonomically neglected family to the surprising focus of several studies, including two recent and competing infrafamilial classifications [44,124]. Both classifications only provide minor changes to the original classification of Faden & Hunt [32], based on unreliable molecular data and no morphological backing. Interestingly, the systematic changes proposed by Lee et al. [124], Zuntini et al. [44], and Feng [143] are mostly taken from my own unpublished PhD thesis (i.e., Pellegrini 2019 [144]), for which the present study represents a much improved and published version. Therefore, some of the proposed classification changes are followed here (i.e., tribe Palisoteae by Zuntini et al. [44], subtribes Cochliostematinae, Commelininae and Murdanniinae by Lee et al. [124], and subtribe Tinantiinae by Feng [143]), with the appropriate author(s) added using “ex”, to ensure credit is given to the original proponents of such changes. Nonetheless, some of the changes proposed by these infrafamilial classifications for Commelinaceae (i.e., subfamily Triceratelloideae by Zuntini et al. [44] and tribe Streptolirieae by Lee et al. [124]) are not followed here since they are not statistically supported (e.g., Zuntini et al. [44] only recover 5 regions out of the 353 expected for their Angiosperm 353 bait kit) or result from low-quality and potentially contaminated sequences (e.g., Zuntini et al. [44] recovers *Aëtheolirion* as sister to the remaining members of subfamily Commelinoideae, while Lee et al. [124] removed this questionable sample from their analysis but still chose to elevate Streptoliriinae to a tribe).

The topology recovered in the present study is pleasantly but unsurprisingly congruent with all previous molecular hypotheses for the family [44,114,115,119,120,121,122,123,124] (Figure 1). It is, however, greatly different from the only published phylogenetic hypothesis for the family based on morphological data [115]. This can be easily explained by a series of factors: (1) analysis not rooted in Zingiberales; (2) outgroup not including Hanguanaceae; (3) small number of characters; (4) unsatisfactory character coding; (5) small sampling, combined with the artificial OTUs that assume the a priori monophyly of all genera; and (6) overdependence on macromorphological characters and the consequent overlook of micromorphological, ecological, cytological, and phytochemical characters. The topology recovered by Evans et al. [115] greatly reflects early macromorphology-only classification attempts for the family, which were generally based on a few and “very important” characters, such as floral symmetry and petal connation. The present phylogenetic hypothesis results from an integrated approach and highlights the importance of not allowing biases and not allowing biases and preconceptions to limit what characters are added to your phylogenetic matrix.

The present analysis recovers Commelinaceae arranged into two major lineages (i.e., subfamilies Cartonematoideae and Commelinoideae), which is congruent with all previous molecular hypotheses, except for Zuntini et al. [44], who questionably recover *Triceratella* as sister to Commelinoideae instead of as sister to *Cartonema* (albeit with no statistical support and almost 99% missing data for *Triceratella*). In the present analysis, Cartonematoideae is recovered as monophyletic with full statistical support. Thus, added to the morphological similarities between *Cartonema* and *Triceratella*, I reduce Triceratelloideae to a synonym of Cartonematoideae. In Commelinoideae, the subfamily is recovered into three major lineages: (1) Tradescantieae; (2) Commelineae; and (3) Palisoteae. Aside from the placement of *Palisota* as sister to tribe Commelineae, these groups precisely match the classification of Faden & Hunt [32]. Thus, the elevation of Palisotinae to the tribal rank is supported here. Within Commelineae, subtribes were tentatively recognised by Pellegrini [144], with two of these being later proposed by Lee et al. [124], but without any morphological support. Subtribe Commelininae equates to the “hook-hair group” of Faden & Hunt [32], and forms a clearly natural group, which is recognised here. However, subtribe Murdanniinae, as proposed by Lee et al. [124], is not only not consistently monophyletic [44,114,115,119,120,121,122,123,124,144]. Despite having some exclusive characters that support this grouping (i.e., character **357**—exine elements closer to each other in the transitional zone, **367**—exine tuberculate, **372**—exine densely microperforate, **467**—cotyledon with middle portion short, and **480**—glandular microhairs with basal cell lenticular and not wedged between the epidermal cells), these actually seem to be plesiomorphic for Commelineae and further studies are need to shed light on this. Nonetheless, the lineages within Murdanniinae (sensu Lee et al. [124]) are consistently and well statistically supported by molecular data [44,114,115,119,120,121,122,123,124,144], in addition to being morphologically supported as well (this study). Thus, I believe the best approach is to recognise these lineages as consistently monophyletic, statistically supported, and morphologically cohesive subtribes: (1) Murdanniinae s.str. (monogeneric); (2) Pseudoparidinae M.Pell. (monogeneric); (3) Buforrestiinae M.Pell. (incl. *Buforrestia* and *Tricarpelema* s.str.); and (4) Floscopinae M.Pell. (incl. *Floscopa* s.str., *Saxofloscopa* M.Pell., and *Stanfieldiella*).

Inside tribe Tradescantieae, the present analysis recovers eight main lineages: (1) Streptoliriinae; (2) Cochliostematinae; (3) Dichorisandrinae s.str.; (4) Cyanotinae s.lat.; (5) Tinantiinae; (6) Thyrsantheminae; (7) Tradescantiinae s.str.; and (8) Callisiinae M.Pell. The relationship between these lineages is more or less consistent throughout phylogenetic studies, with Streptoliriinae being generally recovered as the first lineage, followed either by a clade consisting of Cochliostematinae and Dichorisandrinae s.str. or these two in sequence. Streptoliriinae is morphologically very cohesive, with inflorescence architecture, floral morphology and leaf morphology being characteristic of this group. Cochliostematinae was first indicated by Pellegrini & Faden [145] as morphologically distinct from Dichorisandrinae s.str. Both subtribes are not consistently recovered forming a clade (making Dichorisandrinae s.lat. non-monophyletic), and there are no exclusive morphological characters that support this broader subtribe. Nonetheless, Cochliostematinae is very morphologically and biogeographically cohesive, which merits its recognition. Alternatively, Dichorisandrinae seems to be morphologically less obviously diagnosable, being mostly circumscribed by micromorphological characters. This prompts further studies to be carried out in this group, both on its phylogeny and morphology, to allow us to better understand its systematics. Cyanotinae s.lat. is the next lineage, normally (but not always) with Coleotrypinae recovered as non-monophyletic and nested within it. The relationships between the genera of Cyanotinae s.lat. are still poorly understood, changing greatly depending on the analysis. In some analyses, Coleotrypinae emerges as monophyletic, but lacking statistical support. Nonetheless, the broader circumscription of Cyanotinae is very strongly statistically supported in all analyses, being also morphologically cohesive.

Finally, the Tradescantia alliance (or Tradescantiinae s.lat.) includes the remaining four lineages. Tinantiinae is mostly recovered as part of this clade as the first lineage or as sister to Thyrsantheminae. However, it is also sometimes recovered sister to or before Cyanotinae s.lat. Tinantiinae is recovered in the present study as consisting of *Sauvallia* and *Tinantia*. *Sauvallia* is problematic in that it is potentially extinct, known from a single collection from Cuba consisting of poorly preserved specimens. Thus, no successful DNA has been extracted from these specimens, and the morphological data gathered are mostly tentative. Thus, its placement as sister to *Tinantia* represents the first and only phylogenetic hypothesis for this genus, but needs to be treated as tentative. Thyrsantheminae is morphologically superficially incoherent, but all its members share very peculiar ecological and morphological features. This explains the subtribe being consistently and strongly supported as monophyletic in previous studies, given the exclusion of *Tinantia* and *Elasis*. The final two lineages mostly match the concept of Tradescantiinae proposed by Faden & Hunt [32], except for the inclusion of *Elasis* (originally proposed as a member of Thyrsantheminae). Callisiinae equates to *Callisia* s.lat. proposed by Christenhusz et al. [58] and manages to be simultaneously morphologically cohesive (based on inflorescence, seed, micromorphological, chromosome, and phytochemical characters) and incoherent (based on macromorphological and reproductive biology characters). Finally, Tradescantiinae s.str. (as recognised here) represents the narrowest circumscription for this subtribe ever, finally rendering it not only consistently monophyletic but also morphologically cohesive.

### 3.6. Generic Limits in Commelinales

Out of all currently recognised genera of Commelinales, 49 out of 59 were consistently recovered as monophyletic with strong statistical support, further supported by at least one exclusive synapomorphy (Figure 4, Figure 5 and Figure 6; Table 4 and Table 6). Striking exceptions were: (1) *Wachendorfia* without the inclusion of *Barberetta* (Haemodoraceae); (2) *Anigozanthos* without the inclusion of *Macropidia* (Haemodoraceae); (3) *Conostylis* without the inclusion of *Blancoa* (Haemodoraceae); (4) *Murdannia* without the inclusion of *Anthericopsis* (Commelinaceae); (5) *Tricarpelema* without the exclusion of *T. africanum* (Commelinaceae); (6) *Floscopa* without the exclusion of *F. yunnanensis* (Commelinaceae); (7) *Aneilema* without the exclusion of *A. brasiliense* and the inclusion of *F. yunnanensis* and *Rhopalephora* (Commelinaceae); (8) *Commelina* without the inclusion of *Tapheocarpa* (Commelinaceae); (9) *Coleotrype* without the exclusion of the Malagasy species; (10) *Thyrsanthemum* without the exclusion of *Gibasoides* (Commelinaceae); (11) *Elasis* without the exclusion of *E. guatemalensis* (Commelinaceae); and (12) the hopelessly polyphyletic *Callisia*, which has the morphologically cohesive *Tripogandra* nested within it (Commelinaceae). Additionally, *Aneilema brasiliense*, *Gibasoides*, *Streptolirion*, and *Tricarpelema africanum* (Commelinaceae) are recovered as monophyletic, but are not supported by any exclusive synapomorphies (with *Gibasoides* also not supported by any non-exclusive synapomorphies). These results highlight an interesting historical issue in Commelinales (and several other plant groups), which is the excessive recognition of monospecific taxa (especially genera).

The historical recognition of *Hydrothrix* Hook. f., *Eurystemon* Small, *Scholleropsis* H.Perrier, and *Zosterella* Small made *Heteranthera* (Pontederiaceae) hopelessly polyphyletic [24,25]. The successive pulverisation of *Tradescantia* into a myriad of small or monospecific genera has also caused the paraphyly of the latter, and the recognition of a broader sense was necessary to maintain its monophyly and to facilitate the generic circumscription [28]. Similarly, *Pontederia* was successively split into several genera (e.g., *Cabanisia* Klotzsch ex Schltdl., *Eichhornia* Kunth, *Monochoria* C.Presl, and *Reussia* Endl.), causing the polyphyly of *Eichhornia* [25]. Thus, the recognition of monospecific genera should be carefully contemplated in Commelinales (and throughout plant families), only in cases where recognising these genera meets the following criteria: (1) it does not cause the non-monophyly of other taxa, especially more species-rich ones; (2) the sister taxon(a) is/are also supported by morphological synapomorphies and obviously diagnosable; (3) the alternative option(s) is/are more taxonomically and nomenclaturally disruptive (e.g., broadening the circumscription of a genus to include monospecific taxon/taxa would cause the large genus to become undiagnosable); and (4) the recognition of a monospecific genus will improve and facilitate taxonomy and not make it unnecessarily complicated. In cases where there is a clear incongruence between morphological and molecular data regarding the recognition of such genera, the most conservative approach should be taken to ensure that the larger genus will remain monophyletic, regardless of the dataset, approach or method used. Many historically recognised monospecific genera of Commelinales do not meet those criteria and should not be recognised.

In Philydraceae, *Orthotylax* has been recognised in the past as a monospecific genus distinct from *Helmholtzia*. Given the small size of the whole family (i.e., eight spp. worldwide), one could argue that three genera are more than enough to accommodate all its species. However, the broadly circumscribed *Helmholtzia* is only supported by a single exclusive synapomorphy (vs. two supporting *Orthotylax* and six supporting *Helmholtzia* s.str.), and the total number of morphological characters and genetic divergence between both lineages is equivalent to the number differentiating the other two genera in Philydraceae (Figure 5) [12]. Finally, no additional combinations or taxonomic changes are needed to recognise *Orthotylax* as an independent genus. Thus, *Orthotylax* is presently reestablished as a genus of Philydraceae distinct from *Helmholtzia* s.str. In Pontederiaceae, generic realignments were already carried out [24,25], and no further changes are needed in order to recognise monophyletic and morphologically cohesive genera. In Hanguanaceae, the family is currently considered monogeneric, with its sole genus (i.e., *Hanguana*) being also monophyletic and morphologically cohesive, and the recognition of additional genera would be unnecessary and taxonomically disruptive.

In Haemodoraceae, *Barberetta*, *Blancoa*, and *Macropidia* are indeed highly autapomorphic and can be seen as superficially “very different” from their sister taxa (i.e., *Wachendorfia*, *Conostylis*, and *Anigozanthos*, respectively). However, *Wachendorfia* and *Anigozanthos* in their current and narrower senses are weakly statistically supported and not consistently monophyletic, in addition to not being supported by a single exclusive synapomorphy. With *Conostylis*, the situation is very similar, with the exception that in its current circumscription, it is supported by a single exclusive synapomorphy. Thus, these three genera are currently made unnecessarily difficult to circumscribe and diagnose, due to the historical recognition of their sister monospecific genera. Interestingly, other monospecific genera in Haemodoraceae do meet these criteria, and their recognition is way less taxonomically disruptive than combining them with other genera. As explained by Pellegrini et al. [146], *Cubanicula* is morphologically distinct from *Xiphidium* s.str. due to a combination of floral, fruit, and seed characters. The same features that differentiate it from *Xiphidium* bring it closer to the also monospecific *Pyrrorhiza*, which could have resulted in its sole species being transferred to the latter. However, not only are these two species not consistently recovered as sisters, but they also do not share any exclusive synapomorphies and occur in very different environments in different hemispheres of the world. Finally, *Pyrrorhiza* and *Xiphidium* are also very morphologically different to each other, and combining these three genera into a much broader circumscription of *Xiphidium* would overcomplicate the group’s taxonomy and, consequently, be very taxonomically disruptive. Thus, the recognition of *Cubanicula* and *Pyrrorhiza* as distinct from *Xiphidium* is warranted and followed here. The final example in Haemodoraceae is the monospecific *Lachnanthes*, which is consistently and strongly supported as sister to *Haemodorum*. Both genera are indeed morphologically similar, and one could argue that they could be combined into a broadly circumscribed *Haemodorum*. However, *Lachnanthes* is endemic to North America, while *Haemodorum* is endemic to Oceania, in addition to being individually supported by many exclusive synapomorphies. Thus, they meet all the aforementioned criteria, and their recognition is less taxonomically disruptive than combining both genera.

In Commelinaceae, the situation is somewhat similar to that in Haemodoraceae, as some monospecific genera meet the aforementioned criteria, while many others do not. The currently monospecific *Triceratella* is strongly recovered in the present study as sister to *Cartonema* based on morphology (this study) and molecular data (Zuntini et al. [44], using chloroplast markers). However, both *Cartonema* and *Triceratella* are widely morphologically supported as distinct, each supported by one exclusive synapomorphy, with *Triceratella* supported by 19 non-exclusive synapomorphies and *Cartonema* is supported by 13 non-exclusive synapomorphies. Additionally, both genera are strongly supported as monophyletic, easily diagnosable, and are native to different continents (i.e., *Cartonema* to Oceania, and *Triceratella* to Africa). Finally, merging both genera would require a new combination and could potentially cause the non-monophyly of the genus (in case additional molecular data ever confirm the relationship recovered by Zuntini et al. [44] using Angiosperm 353).

*Anthericopsis* was long recognised as a monospecific genus, closely related to *Murdannia*. However, *Anthericopsis* has been consistently recovered as sister to *Murdannia* [44,115] based on limited sampling or as sister to the Paleotropical *Murdannia juncoides* group or the Neotropical *M. gardneri* group. Additionally, a second species of *Anthericopsis* (i.e., *A. tradescantioides* A.Chiov.) was described and does represent a distinct species. Thus, *Anthericopsis* is reduced as a synonym of *Murdannia*. *Tricarpelema africanum* is recovered as sister to *Stanfieldiella*, which are together sister to *Floscopa*. This clade is morphologically cohesive and statistically strongly supported, as are the two currently recognised genera (i.e., *Floscopa* and *Stanfieldiella*). Both genera and *T. africanum* are also ecologically very distinct, with *Floscopa* being exclusively aquatic, *Stanfieldiella* being restricted to rainforest understories, and *T. africanum* being restricted to inselberg formations. Based on floral, fruit and seed morphology, *T. africanum* shares some features with *Floscopa*, while others are shared with *Stanfieldiella*. Thus, three options exist: (1) transferring *T. africanum* to a broadly circumscribed and morphologically incoherent *Stanfieldiella* (requiring a single new combination); (2) transferring *T. africanum* and all *Stanfieldiella* to a very broad and morphologically incoherent *Floscopa* (requiring eight new combinations); or (3) describing a new monospecific genus for *T. africanum* (requiring the new genus and one new combination). Option three is surprisingly the most conservative and the least taxonomically disruptive one, thus being followed in this study. *Saxofloscopa* M.Pell. is described below (see Taxonomy) to include *T. africanum*. However, a considerable degree of variation is observed for this species and could indicate the need to recognise additional species. *Aneilema* is a peculiar case, with its circumscription being the one requiring the greatest number of changes. First of all, the satellite genus *Rhopalephora* is clearly nested within *Aneilema* and needs to be returned to it as a distinct section. *Floscopa yunnanensis* clearly does not belong in Floscopa s.str., sharing with *Aneilema* all its synapomorphies. This transfer not only makes *Floscopa* finally monophyletic, but also brings *Aneilema* another step closer to its monophyly. This leaves *A. brasiliense*, which is recovered as sister to the African and morphologically distinct *Polyspatha*. On top of the continental disjunction, *A. brasiliense* and *Polyspatha* are morphologically very distinct, and transferring *A. brasiliense* to a broader *Polyspatha* would be very disruptive. The second option, transferring *A. brasiliense* and all *Polyspatha* to a much broader *Pollia*, would be even more disruptive, given that *Pollia* already is the genus in the order with the most contentious circumscription. Due to the limited sampling of the present and previous studies, we still do not know the true relationship between the species with three fertile stamens and the ones with six fertile stamens. Aside from the gem-like fruits, which have evolved several times independently in the family (e.g., *Aneilema*, *Commelina*, *Dictyospermum*, and *Pollia*), these two groups seem to share very few features. Thus, adding even more morphologically incoherent elements to *Pollia* is the last thing we should do. Based on this discussion, the last and most viable option is to recognise a new genus (i.e., *Fadeniella* M.Pell.; see Taxonomy) to accommodate *A. brasiliense*. Luckily, a second species for this new genus will be described shortly, which will prevent it from becoming yet another monospecific genus of Commelinales. The monospecific *Tapheocarpa* is again recovered nested deep inside *Commelina*, and is recognised as a member of the latter.

In tribe Tradescantieae, few similar situations exist. Firstly, as aforementioned, based on the current limited data available, retaining *Sauvallia* as a monospecific genus tentatively positioned as sister to *Tinantia* seems to be the only option. *Tinantia* is morphologically distinct and strongly supported as monophyletic, being supported by at least two exclusive synapomorphies. The monospecific *Aëtheolirion* is strongly recovered (this study; Zuntini et al. [44], using chloroplast markers) as sister to the small *Streptolirion* (four spp.). Additionally, *Streptolirion* is not supported by a single exclusive synapomorphy, while a single exclusive synapomorphy supports the single species of *Aëtheolirion*. When compared to *Aëtheolirion* and *Spatholirion*, *Streptolirion* is easily and clearly distinct, which is also supported by molecular data [44,124]. Thus, combining these genera in any way would disrupt their taxonomy and reverse important past taxonomic studies that highlighted and clarified the differences between them. Recognising all three genera as distinct seems to be the best option, which will hopefully highlight the importance of further taxonomic and systematic studies in this subtribe. *Gibasoides* is a peculiar case in the order. I have previously sunk it under *Thyrsanthemum*, based on morphological data [144,147]. However, the re-examination of further specimens, combined with newly available molecular data, indicates *Gibasoides* is more closely related to *Matudanthus* (currently monospecific, but a second species is also in the course of being published) and *Weldenia*. In that clade, it is easily and undoubtedly differentiated from the other genera, being also easily differentiated from *Thyrsanthemum* s.str. based on inflorescence architecture. Surprisingly, despite the current and gigantic morphological dataset, I have been unable to recover a single character that supports it as independently distinct from *Matudanthus* and *Weldenia*. Based on a combination of morphological and molecular data, it seems indisputable that *Gibasoides* must be reinstated as a distinct monospecific genus, and that further studies are desperately needed to shed some light on its systematics and taxonomy. Finally, *Elasis* was originally described as a monospecific genus. It was later broadened to include *E. guatemalensis* [28], based on morphological data. However, recent molecular data highlight that the many differences between the two species are greater than previously thought. *Elasis guatemalensis* is more closely related to the clearly distinct *Gibasis* than to *E. hirsuta*. With additional new species being described for *Elasis* s.lat. (Pellegrini & Hunt, in prep.), the aforementioned differences became even more apparent, reinforcing the need to recognise a new genus to accommodate *E. guatemalensis* and two additional undescribed species, and for *Elasis* to be restricted to include only *E. hirsuta* and two undescribed species. The new genus, *Ivoniella* M.Pell. (see Taxonomy), is published in the present study, making it available for the publication of a taxonomic revision for both genera (Pellegrini & Hunt, in prep.).

The final generic circumscription issues to be discussed involve *Coleotrype* and the members of subtribe Callisiinae. In its current sense, *Coleotrype* represents a collection of morphologically dispar species. The Malagasy members of the genus have been consistently highlighted for their dissimilarity when compared to the Continental Africa members. Seven exclusive characters support the Malagasy species, while three exclusive synapomorphies support the Continental African species. Additionally, both species groups are not consistently recovered forming a clade, with each clade being variously related to each other and *Amischotolype* s.lat. Thus, the formal recognition of the Malagasy species of *Coleotrype* as a distinct genus is not only necessary but also feels logical. *Nivoanthus* M.Pell. is formally described below (see Taxonomy) to include all Malagasy species formally placed in *Coleotrype*. Numerous taxonomic issues persist in this group, and further studies are essential. Finally, the hopelessly polyphyletic *Callisia* is finally addressed and its circumscription sorted out. *Tripogandra* is morphologically well circumscribed, with its unique inflorescence, androecium arrangement and pollen morphology [28]. However, three species currently placed in *Callisia* sensu Hunt [73] have been consistently recovered nested within *Tripogandra* [28,44,114,115,119,120,121,122,123,124,144,148]. This was used by Christenhusz et al. [58] to justify sinking *Tripogandra* under the synonymy of the polyphyletic, already taxonomically complicated and morphologically incoherent *Callisia*. This decision was extremely disruptive, since it required almost 30 new combinations or new names and made *Callisia* even harder to circumscribe. However, the three aforementioned *Callisia* species recovered within *Tripogandra* do not deviate greatly from other species in the latter genus. Actually, two of them (i.e., *Callisia gracilis* and *Tradescantia triandra*) are consistently misidentified as *Tripogandra* sp. in all herbaria, due to their obvious similarity to *Tripogandra* s.str. The third species, *C. filiformis*, is the only one never formally or informally associated with *Tripogandra*. However, herbarium specimens of this species look remarkably similar to other minute aquatic species of *Tripogandra* [i.e., *T. angustifolia* (B.L.Rob.) Woodson and *T. kruseana* Matuda]. With these species transferred to *Tripogandra*, the remaining lineages of Callisiinae are simple to solve. *Callisia* s.str. consists of xerophytic and very succulent plants with reduced flowers and a tendency towards anemophily. *Callisia warszewicziana* stands out due to its bromeliform habit, main florescences consisting of 2–3 basally fused cincinni, showy and insect-pollinated flowers, and several anatomical peculiarities. The generic name *Hadrodemas* H.E.Moore is already available for this species. *Callisia monandra* and *C. multiflora* have been consistently associated, and even placed in their own genus, *Aploleia* Raf. The only necessary change in circumscription for *Aploleia* would be the inclusion of *C. cordifolia*, which shares all synapomorphies with the other two species. *Callisia rosea* and related species have been consistently associated with each other and placed in the past under the genus *Cuthbertia*. The only lineage lacking a generic name consists of *Callisia navicularis*, *C. hintoniorum*, *C. laui*, and *C. micrantha*. The species in this clade are very succulent xerophytes, commonly presenting tuberous roots, and presenting large and very showy, bright pink to magenta flowers. Thus, the generic name *Huntiella* M.Pell. is proposed here (see Taxonomy), tying the final taxonomic loose end in the family and Commelinales as a whole.

### 3.7. Aquatic Origin of Commelinales

Angiosperms are the most diversified group of green plants on the planet, showing unparalleled diversity in habitats, life forms, and morphology [98,99]. Even though the majority of flowering plants are terrestrial and woody, the origin and diversification of land plants seem to have occurred initially in freshwater environments [35,98,99,149,150]. Two divergent hypotheses on the habit of the first flowering plants are currently accepted [149,150]: (1) woody and terrestrial or (2) herbaceous and aquatic. The hypothesis that the earliest flowering plants were woody seems more likely, which is widely supported by the evidence that most Basal Angiosperms and all extant/extinct Gymnosperms are woody, as are the fossil lineages that are currently considered as most closely related to Angiosperms (e.g., Caytoniales and Bennettitales) [149,150,151].

Alternatively, an aquatic origin of the Angiosperms is tentatively supported by the fact that several of the earliest known fossil flowering plants were aquatic, and the second group of extant flowering plants to arise was the waterlilies (i.e., Nymphaeales) [149,150,151,152]. Nevertheless, the extensive fossil record of aquatic angiosperms certainly indicates that the aquatic habit arose early in the evolution of the flowering plants and might have been crucial in the diversification of several groups [35,153,154]. This view is further supported by the aquatic origin of the Monocots (reinforced by the aquatic habit of its first and second groups to diverge—Acorales and Alismatales—plus several unique anatomical features) [35,152], the sister placement of the aquatic group Ceratophyllales to the remaining Eudicots, and several independent origins of the aquatic habit in the Eudicots (e.g., in families such as Elatinaceae, Haloragaceae, Menyanthaceae, Nelumbonaceae, Onagraceae, Podostemaceae, Scrophulariaceae, Ranunculaceae, etc.) [35,149,152]. Therefore, it is evident that the evolution of the aquatic life form has occurred independently several times in the evolution of flowering plants, in unrelated groups of flowering plants, and during different geological periods. It is also clear that the aquatic life form has played a vital role in flowering plant evolution and diversification, allowing the establishment of important lineages and different lineages of flowering plants to colonise extreme environments such as brackish (e.g., Hydrocharitaceae), salt (i.e., seagrasses; Posidoniaceae, Zosteraceae, Hydrocharitaceae, and Cymodoceaceae), and rapidly moving waters (i.e., Hydrostachyaceae and Podostemaceae) [35,149,150,155].

Despite the aforementioned evolutionary relevance of the aquatic life form in Angiosperms and Monocots, this ecological trait has never been investigated in any other order of Monocots, aside from Acorales and Alismatales. However, as shown in the present study, several species of Commelinales are paludal, aquatic, or at the very least wetland-dependent (character 5). More precisely, all species in Pontederiaceae are aquatic, with several of them most commonly paludal in waterlogged soils. In Philydraceae, *Philydrum* grows exclusively in still to slow-moving freshwater environments (most commonly lakes), while *Helmholtzia* and *Orthotylax* are rheophytes, and *Philydrella* grow in seasonally flooded white sand environments. In *Hanguana*, species like *H. malayana* are aquatic, even forming floating vegetation islands. At the same time, other species in the genus (e.g., *H. exultans*, *H. loi*, *H. major*, *H. nitens*, *H. podzolica*, and *H. pantiensis*) are paludal and grow in waterlogged soils. In Haemodoraceae, all recognised genera have at least one wetland-dependent species, with over 50% of the accepted species growing in seasonally flooded environments. Finally, in Commelinaceae, the aquatic life form is slightly less common, but still omnipresent. *Cartonema* and *Triceratella* (subfamily Cartonematoideae) grow in seasonally flooded white sand environments, similar to the ones occupied by *Philydrella* and several Haemodoraceae. *Floscopa* and *Murdannia* are wholly or mostly aquatic, while aquatic and paludal species are rather common in other genera (e.g., *Commelina* and *Tripogandra*).

In addition to the current omnipresence of the aquatic life form in extant members of Commelinales, several taxa share a series of morphological characters commonly associated with the aquatic syndrome. For instance, seedlings with ribbon-like primary leaves, reported for Philydraceae and Pontederiaceae, were also reported by Tillich (1995) [105,108,109] in other Monocot families (e.g., Cyperaceae, Eriocaulaceae, some Juncaceae, Mayacaceae, Poaceae, Rapateaceae, Restionaceae s.lat., Thurniaceae, Typhaceae, and Xyridaceae) with primarily paludal and aquatic species. In addition to that, the seed morphology of Commelinales is very reminiscent of several other aquatic groups. The presence of an embryotega in all Commelinales seeds (i.e., an operculum that facilitates embryo germination) is shared with countless mostly to completely aquatic groups, such as Alismatales (all families), Lamiales (e.g., Byblidaceae, Lentibulariaceae, Linderniaceae, Schlegeliaceae, and Scrophulariaceae), Malpighiales (i.e., Elatinaceae and Podostemaceae), Nymphaeales (all families), and Poales (Bromeliaceae, Cyperaceae, Eriocaulaceae, Juncaceae, Mayacaceae, Rapateaceae, Restionaceae s.lat., Thurniaceae, Typhaceae, and Xyridaceae). The longitudinally striated/crested/winged seed testa ornamentation, which is widespread in Commelinales (especially Haemodoraceae, Philydraceae and Pontederiaceae), is found in many but not all mostly to completely aquatic groups, such as Alismatales (all families), Lamiales (widespread in paludal and aquatic Scrophulariaceae), Malpighiales (Podostemaceae), Nymphaeales (all families), and Poales (Bromeliaceae, Cyperaceae, Eriocaulaceae, Juncaceae, Mayacaceae, Rapateaceae, Restionaceae s.lat., Thurniaceae, Typhaceae, and Xyridaceae). Additional characters that are very common in aquatic plants observed in Commelinales include [156]: (1) unifacial, terete or subterete leaves (present in Pontederiineae and a handful of Commelinaceae species); (2) heteroblasty/heterophylly (present in Pontederiaceae in the form of sessile/immature and petiolate/mature leaves, but also present in some facultative submerged aquatic species of Commelinaceae with leaves clearly adapted to underwater); (3) production of mucilage (widespread in Commelinales); (4) thin cuticle (widespread in Commelinales); (5) stomatal development suppression upon submergence (widespread in Commelinales); and (6) roots with aerenchyma and air canals (widespread in Commelinales).

**Figure 19:**
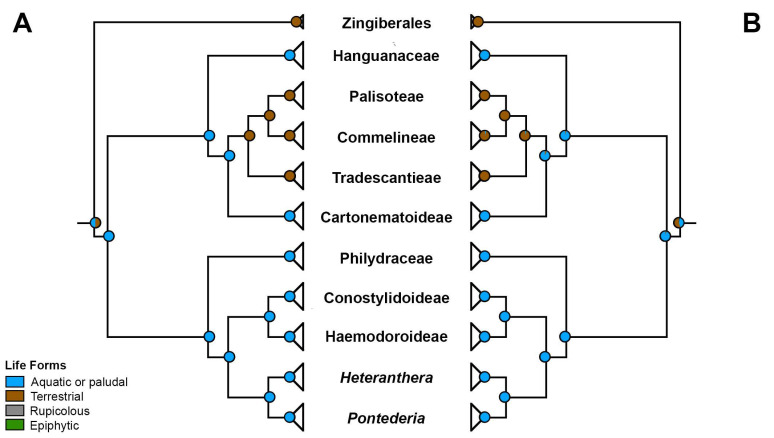
Simplified consensus tree for Commelinales showing the ancestral state reconstruction of the aquatic life form. (**A**), Parsimony reconstruction. (**B**), Maximum Likelihood reconstruction.

Despite the aquatic life form (character 5) not being recovered in the present analysis as synapomorphic for Commelinales (Figure 4), this seems to be an analysis artefact. In order to test this hypothesis, I have constructed the ancestral character state for Commelinales, using both Parsimony and Maximum Likelihood (Figure 19). The Parsimony approach reconstructs the last common ancestor for Commelinales as being undoubtedly aquatic, with the same being true for the last common ancestors for all five families, Commeliniineae, and Pontederiineae (Figure 19A). However, when reconstructed using Maximum Likelihood, only the last common ancestors for Hanguanaceae, Haemodoraceae, Pontederiaceae and Pontederiineae are recovered as undoubtedly aquatic. In contrast, the last common ancestors for Commelinaceae, Commeliniineae, and Commelinales are recovered as most likely aquatic, with a statistically irrelevant chance of the ancestor being terrestrial (Figure 19B). Interestingly, the last common ancestor for Commelinales and Zingiberales is reconstructed using Parsimony as being ambiguously either aquatic or terrestrial (Figure 19A). In contrast, it is reconstructed as most likely terrestrial (slightly over 50%) using Maximum Likelihood (Figure 19B). These reconstructions not only support the aquatic life form as synapomorphic and ancestral in Commelinales but also highlight the potential evolutionary importance of this character in the evolution and diversification of the order.

### 3.8. Evolution of Enantiostyly in Commelinales

Enantiostyly (character **142**) is a type of floral asymmetry, more precisely, a type of stylar polymorphism that is caused by the reciprocal deflection of the style either to the left (left-handed) or right (right-handed) side of a bisexual flower. Like several other types of stylar polymorphisms, enantiostyly has the primary function of reducing geitonogamous selfing, and thus, improving proficient cross-pollination [157,158]. Enantiostyly has evolved independently in 11 Angiosperm families, being reported for both Monocots and Eudicots [41,159]. However, in the Monocots, this feature is currently only known to be restricted to two distantly related lineages [41]: (1) *Cyanella* D.Royen (Tecophilaeaceae, Asparagales) and (2) Commelinaceae, Haemodoraceae, Philydraceae, and Pontederiaceae (Commelinales). The term was first coined by Knuth [160], but only in the past 30 years have the evolution and reproductive relevance of this feature been studied [161,162]. Based on field observations, Dulberger [163] suggested a set of floral features that circumscribe the enantiostylous flower syndrome: (1) nectarless flowers; (2) heteranthery (i.e., labour division between anthers); (3) poricidal anthers; (4) food pollen deposition on the ventral side of pollinators and reproductive pollen deposition on the dorsal or lateral side of the pollinators; (5) curved style; and (6) a small stigma. Nonetheless, several enantiostylous taxa do not present many of these features, highlighting our still incomplete understanding of enantiostyly.

Knuth [160] initially recognised three types of enantiostylous flowers: (1) with monomorphic anthers; (2) heterantherous but without preferential intermorph crosses; and (3) heterantherous with preferential intermorph crosses. The current classification of enantiostyly has oversimplified this system, recognising only two subtypes, based on the distribution of floral morphs per species [161]: (1) monomorphic, where species exhibit both floral morphs that are randomly distributed (random enantiostyly), alternately distributed in the inflorescence (non-random enantiostyly), or exclusive in the inflorescence (monomorphic individual has right- and left-handed inflorescences), and (2) dimorphic, where species have only one floral morph (i.e., the population has either right- and left-handed individuals). Monomorphic enantiostyly is the most widespread one, with dimorphic being restricted to Tecophilaeaceae, and the sister families Haemodoraceae and Pontederiaceae, representing a non-exclusive synapomorphy for them. In addition to the distribution of floral morphs, enantiostylous species are further classified based on the deflection of the androecium in relation to the gynoecium [164]: (1) reciprocal, where the androecium (or at least the reproductive anther/s) and gynoecium are deflected in opposite directions as the gynoecium, and (2) non-reciprocal, where the androecium is not deflexed, while only the gynoecium is deflected to either side of the flower. A third type is known exclusively to *Murdannia* (Commelinaceae), where the androecium (or at least the reproductive anther/s) is deflected to either side of the flower, while the gynoecium remains straight ([41,114,115], this study). Thus, enantiostylous species can be classified by a combination of the aforementioned features as reciprocal monomorphic, reciprocal dimorphic, non-reciprocal monomorphic, and non-reciprocal dimorphic. Non-reciprocal monomorphic enantiostyly is the most widespread of the types, being observed in all families with enantiostylous taxa [159]. In case the enantiostyly is reciprocal, it will produce mirror-image flowers, where the left- and right-styled morphs are a perfect reflection of each other [41,165]. In enantiostylous species, the timing of stylar deflection from the floral axis either happens while in the bud (as in Commelinaceae, Haemodoraceae and Philydraceae, but also Tecophilaeaceae) or at the beginning of anthesis (as in Pontederiaceae) ([159], this study), which would make the former a non-exclusive synapomorphy for Commelinales. At the same time, the latter would represent another exclusive synapomorphy of Pontederiaceae.

As aforementioned, enantiostyly has previously been widely reported for Haemodoraceae (subfamily Haemodoroideae), Philydraceae, and Pontederiaceae (*Heteranthera* and *Pontederia* subg. *Monochoria*). Alternatively, reports of enantiostyly in Commelinaceae have been sparse and inconsistent. This feature has been previously recorded for *Aneilema*, *Amischotolype* s.lat., the Malagasy *Coleotrype*, *Cochliostema*, *Commelina*, *Murdannia*, and *Tinantia*. Nonetheless, previous studies have failed to observe the enantiostyly in the flowers of *Cartonema*, *Dictyospermum*, *Floscopa*, *Pollia*, *Polyspatha*, *Pseudoparis*, *Stanfieldiella*, and *Tricarpelema africanum*, which are reported here for the first time. The only family of Commelinales never investigated for this feature is the poorly understood Hanguanaceae.

Hanguanaceae are peculiar in that they are dioecious, with functionally unisexual flowers, where the pistillate ones lack a style, and the pistilode of the staminate ones has a vestigial and narrowly conical style [21,38]. As aforementioned, and also made obvious by its name, enantiostyly seems to be inherently dependent on the presence of a developed style [41,165], but not necessarily its length. The fact that the flowers of *Hanguana* are always unisexual creates a unique scenery, since enantiostyly would completely lose its purpose of reducing the geitonogamous selfing, due to the impossibility of self-pollination in such a flower. Thus, by definition, the flowers of *Hanguana* cannot be classified as enantiostylic due to being unisexual and the absence of a style. Nonetheless, several species of *Hanguana* present non-terminal stigmas [166], which creates the same floral asymmetry as the one described for enantiostylous flowers (pers. observ.). The stigmas in these flowers are oblique to laterally inserted in the ovary, being observable even in herbarium specimens. Thus, my working hypothesis is that this feature in *Hanguana* is homologous to, but more precisely derived from, an ancestrally enantiostylous flower. Unisexual flowers are often associated with miniaturisation, as observed in several groups of aquatic plants (e.g., Araceae, Cabombaceae, Ceratophyllaceae, and Hydatellaceae) or terrestrial plants (e.g., Amborellaceae, Casuarinaceae, Eriocaulaceae, Moraceae, Urticaceae, etc.) [118]. Nonetheless, exceptions are as common as the rule, and a direct correlation cannot be properly made. On the other hand, the occurrence of style polymorphism seems to be directly limited by floral size, with small flowers rarely presenting any kind [167]. By definition, unisexual flowers are not able to present any type of style polymorphism. Furthermore, the loss or reduction of the style seems to be a common byproduct of miniaturisation, with most diminutive flowers completely lacking styles or presenting extremely reduced styles (e.g., Acoraceae, Araceae, Hydatellaceae, Pandanaceae, Poaceae, etc.). Thus, it seems most likely that the loss of the style occurred concomitantly with the floral miniaturisation and dioecy (or at least with the appearance of unisexual flowers), hypothetically leading to a secondary loss of the enantiostyly in Hanguanceae. Floral ontogeny might be key to understanding and properly addressing enantiostyly in *Hanguana*.

**Figure 20:**
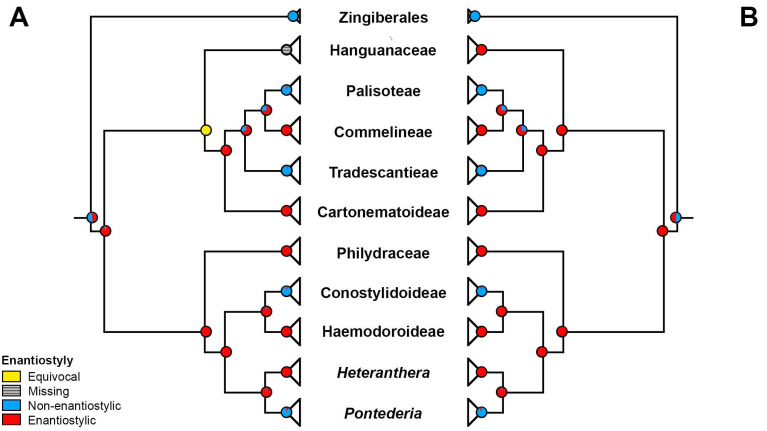
Simplified consensus tree for Commelinales showing the ancestral state reconstruction of enantiostyly (both reconstructed using Maximum Likelihood). (**A**), Mapping with Hanguanceae coded as missing. (**B**), Mapping with Hanguanaceae extrapolated as enantiostylic.

In the present morphological dataset, Hanguanaceae was coded as “?” (i.e., dubious) for the presence of enantiostyly. This causes the last shared ancestor of Commelinales to be reconstructed as most likely to have had enantiostylous flowers (Figure 20A). Alternatively, I have also coded Hanguanaceae as presenting enantiostylous pistillate flowers. In this second scenario, the last common ancestor of Commelinales is undoubtedly recovered as enantiostylous (Figure 20B), supporting the hypothesis of Givnish et al. [3] and Rudall & Bateman [168]. Finally, based on the rarity of enantiostyly in the Monocots [41], it would make much more evolutionary sense for the common ancestor of the order to be enantiostylous, with posterior reversions to non-enantiostylous flowers in several of its lineages, including Hanguanaceae. Thus, enantiostyly is inferred here as the ancestral state for Commelinales (Figure 20), supporting the hypotheses by Givnish et al. [3] and Rudall & Bateman [168].

### 3.9. Systematic and Phylogenetic Importance of Floral Anatomy for Commelinales

Up until the present study, Commelinales has been circumscribed based exclusively on molecular characters, with no obvious synapomorphies recovered for the group, in addition to the lack of morphological characterisation for the group. Thus, it seems unsurprising that most of the recovered synapomorphies in the present study are micromorphological. However, it does seem peculiar that these mostly relate to floral anatomy, which seems to contradict the massive variation in floral morphology observed in Commelinales (Figure 2, Figure 3, Figure 4, Figure 5, Figure 6, Figure 7, Figure 8, Figure 9, Figure 10, Figure 11, Figure 12, Figure 13, Figure 14, Figure 15 and Figure 16). Out of all floral anatomy characters, two main groups seem to be more systematically relevant: (1) tannin cells; and (2) cell inclusions. These are briefly discussed below, especially the need for further studies.

**Figure 21:**
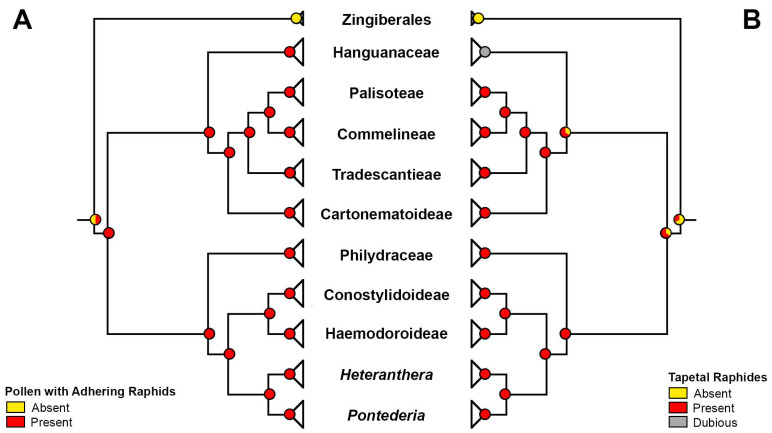
Simplified consensus tree for Commelinales showing the ancestral state reconstruction of floral tannins (both reconstructed using Maximum Likelihood). (**A**), Receptacle tannins. (**B**), Perianth tannins.

Tannins are secondary phenolic compounds produced in plants for chemical defence, due to their special properties, such as precipitating alkaloids, gelatines, and other proteins [169,170]. These are widespread in several plant lineages and organs, with their distribution, morphology and chemical structure with great systematic potential [171,172,173,174]. Tannins and their distribution have been mostly investigated in leaves and other vegetative organs [174]. Alternatively, floral tannins have been mostly investigated in Basal Angiosperms and Eudicots [173,175] and only sparsely reported in the Monocots [171,172,173,175]. Of the few records in the Monocots, most are restricted to members of the Commelinids, and the only records in Lilioid Monocots are for Asparagales (Orchidaceae) [176,177]. In the Commelinid Monocots, floral tannins are known to occur exclusively in Arecales (Arecaceae) [178,179,180,181] and Commelinales (Commelinaceae [28,132,133,182], this study, Haemodoraceae [19,20,25,52], Philydraceae [19,20,25,52], and Pontederiaceae [19,20,25,52,183]). Most specifically, receptacle and perianth with tannin cells (characters **529** and **531**) seem to be uncommon in Angiosperms, being recorded for only a few families but mostly in eudicots (e.g., Dilleniaceae [184], Urticaceae [185], Balsaminaceae, Marcgraviaceae and Tetrameristaceae [186], Melastomataceae [187], and Adoxaceae [188]), being otherwise found in the Monocots only in Arecales and Commelinales (see aforementioned references). It stands out that Hanguanaceae seems to be the only member of Commelinales that lacks floral tannins. Rudall et al. [38] investigated the floral anatomy of Hanguanaceae for the first time, highlighting several peculiar structures, including hairs, nectaries, and cell inclusions. Surprisingly enough, not a single reference is made to tannins, even though dark-staining and tannin-like inclusions are clearly observed in the receptacle and perianth of the analysed samples (see [38], their Figures 1, 2 and 12–14). Based on these secondary observations, I have coded Hanguanaceae in the present study as presenting tannin cells in both the receptacle and the perianth. Nonetheless, further studies of reliably identified samples of both staminate and pistillate flowers need to be conducted to test their potential systematic relevance within *Hanguana*. Furthermore, no studies have compared the morphology, chemical composition or ontogeny of these tannin cells in Arecales and Commelinales, in order to investigate their potential homology. Thus, based on the limited information currently available, the presence of receptacle and perianth tannin cells (characters **529** and **531**) is considered a potentially non-exclusive synapomorphy for Commelinales (Figure 21), shared in the Monocots only with Arecaceae and the distantly related Orchidaceae.

**Figure 22:**
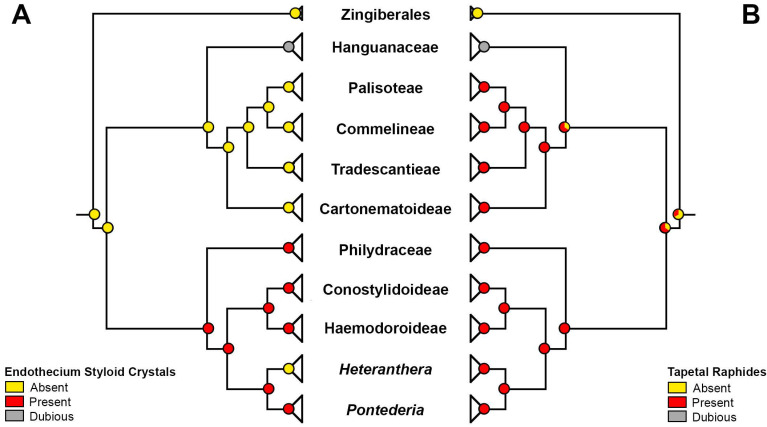
Simplified consensus tree for Commelinales showing the ancestral state reconstruction of two characters relating to the presence of androecium crystals (both reconstructed using Maximum Likelihood). (**A**), Endothecium styloid crystals. (**B**), Tapetal raphides.

Commelinales are particularly variable for all types of cell inclusions [53], with silica bodies having been previously recorded for all families of Commelinales [19,22,25,28,38,46,47,48,49,50,51,52,53,54,116,117,130,131,132,133,183,189,190,191]. These were investigated from an anatomical and descriptive perspective by Prychid et al. [53], who named the group the “Haemodoraceae and allied families” due to their exclusion of Hanguanaceae, since they believed the family was to be positioned in the Zingiberales [38]. This study gave special focus to the presence of tapetal raphides (character **544**), which were sometimes accompanied by endothecium styloid crystals (character **520**) [53]. Tapetum producing raphides and occasional styloid crystals is a unique character reported for Commelinaceae [53,189,190,191], Haemodoraceae [19,46,47,48,49,53], Philydraceae [2,53], and Pontederiaceae [52,53]. This character has the potential of being an exclusive synapomorphy for Commelinales, but further studies are needed to increase the sample in Commelinaceae (several genera not yet investigated) and include Hanguanaceae. Despite investigating the tapetum morphology of *Hanguana*, Rudall et al. [38] do not mention the presence of either raphides or styloid crystals. Further analysis of the images presented by Rudall et al. [38] has not been able to confirm or refute the absence of tapetal raphides and styloid crystals in Hanguanaceae. For this reason, *Hanguana* was coded in the present study as “?” (i.e., dubious) for characters **520** and **544** (Figure 22). Currently, the presence of endothecium styloid crystals (character **520**) is recognised as an exclusive synapomorphy for Pontederiineae (i.e., [Philydraceae [Haemodoraceae + Pontederiaceae]]) (Figure 21A). In contrast, the presence of tapetal raphides (character **544**) is recognised as an exclusive synapomorphy of Commelinales, despite its dubious occurrence in Hanguanaceae (Figure 21B).

**Figure 23:**
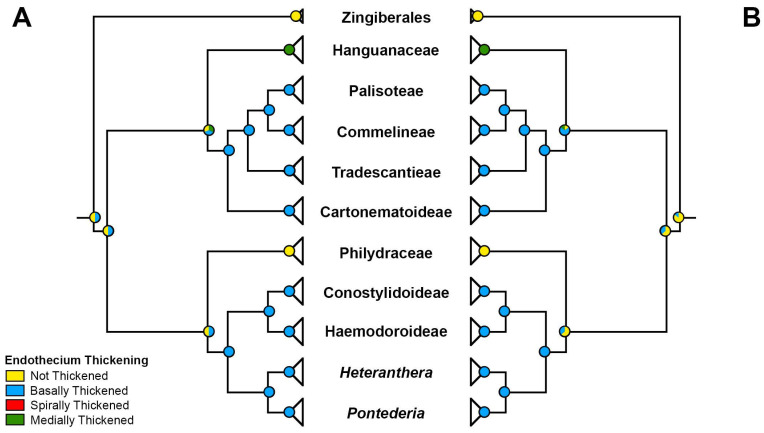
Simplified consensus tree for Commelinales showing the ancestral state reconstruction of endothecium thickening. (**A**), Parsimony reconstruction. (**B**), Maximum Likelihood reconstruction.

Finally, basally thickened endothecium (character **542**) represents the last recovered synapomorphy for Commelinales (Figure 23). Manning [192] previously investigated the endothecial patterns in Angiosperms, providing a very detailed summary of the occurrence of the known endothecium types in all flowering plants. For the Monocots, Manning [192] states that the U-shaped endothecium is the most common and most likely the symplesiomorphic type, with basal plates (i.e., basal thickenings) evolving several times independently in the group. Out of the five families of Commelinales, three were investigated (i.e., Commelinaceae, Haemodoraceae, and Pontederiaceae), being characterised as having U-shaped endothecium with basal thickening. For Zingiberales, the order was characterised as having U-shaped, helical, columnar, annular or pseudoannular endothecium, but consistently lacking a basal thickening. For Hanguanaceae, Rudall et al. [38] report the endothecium as being columnar and presenting medial thickenings, instead of basal. Finally, Haumann [22,23] reports the endothecium in Philydraceae to be either columnar or spiral, lacking basal thickenings. Thus, despite Manning [192] stating that U-shaped endothecium is most likely plesiomorphic for Monocots, the author clearly states that basal plates have evolved independently in several lineages, which is reconstructed here (Figure 23) as the ancestral character for Commelinales. Thus, it is recovered in the present analysis as synapomorphic for Commelinales, with a reversal in Philydraceae, and the unique columnar endothecium with a medial thickening recovered as synapomorphic for Hanguanaceae (Figure 23).

### 3.10. Morphological × Molecular Data in Commelinales

**Figure 24:**
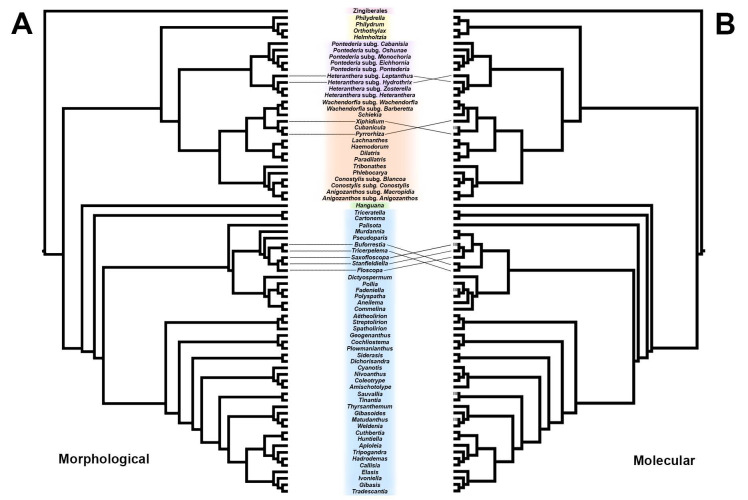
Comparison between morphology- and molecular-based phylogenetic hypotheses for Commelinales, reduced to genus-level. (**A**), Maximum Parsimony consensus tree based on the present dataset. (**B**), Maximum Likelihood tree from Zuntini et al. (2021) [44] based on a combined *matK*, *rbcL*, and *trnL-trnF* dataset. Genera with hatched branches not previously sampled in the molecular topology, thus, having their position extrapolated based on morphology.

Despite the high degree of homoplasy in morphological datasets for the order [25,28,114,115,119,144,193,194,195], they are still congruent with the molecular dataset, and therefore, informative and relevant for phylogenetic inference. Commelinales has been historically plagued by the discourse that morphological data is unfit and unreliable for phylogenetic assessments [3,25,28,114,115,119,193,194,195]. However, these articles readily choose to ignore the efforts and results of Simpson [19], who recovered a morphology-based topology for Haemodoraceae that is still greatly congruent with molecular hypotheses for the family. Additionally, Kress [196] also had similarly impressive results for the sister order Zingiberales using only morphology. Later on, Pellegrini [28] and Pellegrini et al. [25] had similarly positive and congruent results for the Tradescantia alliance (Commelinaceae) and Pontederiaceae, respectively. The topology recovered in the present study is greatly congruent with the two most well-sampled molecular phylogenetic hypotheses for Commelinales [41,44]. The present overall topology is essentially the same as the ones presented by Jesson & Barrett [41] and Zuntini et al. [44] (Figure 20). The observed incongruence between both data types is really minor, with the most drastic differences being observed in three points (Figure 24): (1) Pontederiaceae, between *Heteranthera* subg. *Leptanthus* and *H.* subg. *Hydrothrix*; (2) in Haemodoraceae, between the genera of tribe Xiphidieae; and (3) in Commelinaceae, between subtribes Murdanniinae s.str., Pseudoparidinae, Buforrestiinae, and Floscopinae. Of these, only the placement of *Pyrrorhiza* potentially questions the monophyly of Xiphidieae, since it is recovered as the first lineage of Wachendorfieae (Figure 19B). However, this dataset lacks *Cubanicula*, which is recovered here and on a much more well-sampled dataset using further markers (Hopper et al., in prep.) as sister to *Pyrrorhiza*, supporting its inclusion in Xiphidieae and not Wachendorfieae. In Pontederiaceae, the different position of the first two subgenera of *Heteranthera* between datasets is not surprising. *Heteranthera* subg. *Hydrothrix* has historically been recovered with distinct relationships within the genus: (1) as the first lineage; (2) as the second lineage; (3) as sister to *H.* subg. *Zosterella*; or (4) even nested within *H.* subg. *Zosterella*. Morphologically, *H.* subg. *Zosterella* does share several features with *H.* subg. *Hydrothrix*, which could help explain the last two phylogenetic placements. However, it is a clear morphological outlier in the genus, and this placement is not supported by morphology, nor do molecular data consistently support it. Thus, recognising *H.* subg. *Hydrothrix* as a distinct subgenus seems the most conservative and less disruptive option. Finally, the different relationship hypotheses between subtribes Murdanniinae s.str., Pseudoparidinae, Buforrestiinae, and Floscopinae are not very surprising. Despite being individually strongly and consistently supported in all datasets, their relationships are equally ill-supported based on both morphological and molecular data. This reinforces the need for further morphological and molecular studies to clarify these relationships.

Despite being the most well-sampled molecular phylogeny for Commelinales, Zuntini et al. [44] is far from being a good one. First and foremost, the study mostly focuses on the topology generated using Angiosperm 353, which is not only well known as not being a great bait kit in general, but it is also admittedly known as a very unreliable bait kit for Monocots, and even worse for Commelinid Monocots (A. Zuntini & W. Baker, pers. comm.). Additionally, Angiosperm 353 provides very inconsistent and unreliable recovery, as exemplified by *Triceratella*, where only 5 out of 353 regions were recovered. Another serious issue with Zuntini et al. [44] is that the study was carried out by botanists with no experience or familiarity with the order, which is aggravated by the chosen sampled specimens not being reliably identified. The taxonomic impediment in phylogenetic studies is consistently disregarded by most botanists, resulting in grotesque and very obviously inaccurate results [197]. As discussed by Almeida et al. [197], having reliably identified and sampled vouchers is imperative for scientifically accurate, reliable, and replicable phylogenetic results. Unfortunately, Zuntini et al. [44] do not meet these criteria. I have personally checked the identification of all vouchers cited by the authors, and most of their claimed “unexpected phylogenetic placements” are simply explained by misidentified samples. The remaining ones are explained by sample contamination and low-quality sequences, which were recklessly kept in their final analysis due to the huge amount of generated and analysed data (W. Baker, pers. comm.). Thus, the topology recovered by Zuntini et al. [44] using Angiosperm 353 needs to be considered with caution. However, the topology generated by their “expanded plastid dataset” (mostly retrieved from GenBank) provides a much more robust and reliable topology, with many fewer misidentified or contaminated sequences. This unsurprisingly results from them using data generated and curated by or in collaboration with specialists in Commelinales.

Regarding interfamilial relationships in Commelinales, numerous hypotheses have been proposed based on molecular data (Figure 1). Of these, the one that is more consistently recovered and with the strongest statistical support (Figure 1A) is also the only topology recovered using morphology (Figure 1A and Figure 4). As already discussed above (see interfamilial relationships in Commelinales), this provides a strong argument in favour of the inclusion of morphological data in phylogenetic analyses, at least in situations where molecular data alone are unable to resolve relationships. A very similar scenario can be observed in the sister order Zingiberales, where molecular data alone are unable to resolve the relationships between its families, while morphology is not only greatly congruent but also recovers a much more strongly supported and well-resolved topology [2,196].

#### 3.10.1. Philydraceae

Only two previous studies present a significant sampling of Philydraceae [12,44]. In Saarela et al. [12], *Philydrella* is recovered as sister to the rest of the family, followed by *Philydrum*, *Helmholtzia*, and *Orthothylax*, while Zuntini et al. [44] recovered a yellow-flowered clade (i.e., *Philydrella* and *Philydrum*) sister to a white-flowered clade (i.e., *Helmholtzia* and *Orthothylax*). The present study fully supports the topology of Saarela et al. [12], which indicates open and seasonally flooded environments, an aquatic life form (vs. rheophytic), and yellow flowers are not only ancestral for Philydraceae, but also support these traits as ancestral to the whole order. Currently, only three of eight species of Philydraceae have never been sampled in molecular studies (i.e., *Philydrum cochinchinensis*, *Philydrella minima*, and *Helmholtzia novoguineensis*).

#### 3.10.2. Pontederiaceae

Pontederiaceae has been extensively phylogenetically investigated, using morphological data, molecular data and/or a combination of both [25], and references therein. Molecular and morphological data are greatly congruent and complement each other by providing better support and further resolution for different parts of the tree. The present study confirms that by not only recovering an almost entirely congruent topology to all previous molecular ones, but also highlighting the importance of morphology in solving phylogenetic relationships that molecular data have been unable to solve on their own (e.g., *Heteranthera* subg. *Hydrothrix*).

#### 3.10.3. Haemodoraceae

In Haemodoraceae, the incongruence between molecular and morphological data is limited to species-level relationships, especially between monospecific genera. As aforementioned, the placement of *Pyrrorhiza* changes slightly between the morphological and molecular datasets, but also between different molecular datasets. However, morphology agrees with the expanded molecular dataset, making this incongruence an artefact caused by limited sampling and fewer plastidial regions in the analysis. For the other monospecific genera that suffer from phylogenetic incongruence (i.e., *Barberetta*, *Blancoa*, and *Macropidia*), morphology does not consistently support their placement as sister to instead of nested within their sister genera (i.e., *Wachendorfia*, *Conostylis*, and *Anigozanthos*, respectively). However, these small incongruences only highlight the relevance of morphology when making taxonomic decisions. Retaining these monospecific genera as independent actually hampers the taxonomy of the family, instead of simplifying it as argued by taxonomists who are defenders of recognising monospecific satellite genera. For instance, despite the strong molecular support for *Blancoa* as sister to *Conostylis* s.str., morphology inconsistently and weakly recovers a monophyletic *Conostylis* s.str., supported by a single exclusive synapomorphy (character **528**—pendulous-peltate placenta). This strongly argues against maintaining both genera as independent. The case of *Anigozanthos* s.lat. and *Wachendorfia* s.lat. are similar, where morphology does not support the recognition of *Macropidia* as independent from *Anigozanthos* s.str. and *Barberetta* as independent from *Wachendorfia* s.str. In the few trees in which *Macropidia* is recovered as sister to *Anigozanthos* s.str., only three non-exclusive synapomorphies support that relationship. Alternatively, for the few trees that recover *Barberetta* as sister to *Wachendorfia* s.str., only nine non-exclusive synapomorphies support that relationship. For all three genera, their strict sense circumscription has no statistical support in the morphological dataset.

A molecular phylogeny for *Conostylis* s.lat. was presented by Hopper et al. [65], where most of the infrageneric classification proposed by Macfarlane et al. [198] was recovered as non-monophyletic. According to the authors, they were unable to give any morphological support for the recovered topology. Nonetheless, the morphology-based topology recovered in the present study is considerably similar to the molecular one. I have recovered the same four main lineages, despite some minor incongruences, highlighting the importance of morphology-based phylogenies for large and taxonomically complex genera.

#### 3.10.4. Hanguanaceae

Hanguanaceae has only two published molecular hypotheses [44,199]. Both articles have a very limited species sampling, but recover congruent topologies to the morphology-based topology presented here. However, at least a few dozen species of *Hanguana* remain undescribed, and the family’s taxonomy has suffered from a lack of standardised terminology and an overreliance on fruit and seed characters (completely ignoring floral characters, especially for staminate plants). Combined with the lack of studies on the macro- and micro-morphology of these plants, the present morphological matrix is admittedly flawed and should be significantly improved with the addition of recently described and yet undescribed species, as well as detailed investigations of systematically relevant characters for Commelinales that have been overlooked for Hanguanaceae.

#### 3.10.5. Commelinaceae

When comparing the present topology for Commelinaceae with previous molecular hypotheses for the family [44,114,115,119,120,121,122,123,124], it is possible to notice that the topology is almost the same between all articles and datasets, with only a few incongruences between them. Historically contentious placements in the phylogeny of Commelinaceae include those of *Palisota*, Cochliostematinae, Callisiinae, and between subtribes Murdanniinae, Pseudoparidinae, Buforrestiinae, and Floscopinae. *Palisota* is resolved here as sister to tribe Commelineae, providing morphological support to recent molecular studies [44,122,123,124]. Cochliostematinae is recovered as monophyletic, but more importantly, not as sister to Dichorisandrinae, as sometimes recovered based on molecular data [44,114,115,119,120,121,122,123,124]. Finally, the relationship between subtribes Murdanniinae, Pseudoparidinae, Buforrestiinae and Floscopinae remains, unfortunately, unresolved. However, they are confirmed as strongly monophyletic.

Regarding infrageneric relationships, some genera merit attention. *Palisota* has a preliminary molecular phylogeny based solely on *rbcL* [122] in which the authors recovered the genus organised in two clades. The same topology was recovered in the present study. However, further phylogenetic studies are currently underway (E. Bidault, pers. comm.), which will include an almost complete species sampling for the genus using several plastidial markers, and seem to show a slightly distinct and more complicated phylogeny for the genus. Prior to this study, the phylogenetic relationships within *Aneilema* had only been investigated using molecular data [70]. These authors recover a paraphyletic *Aneilema*, due to *A. brasiliense* being recovered as sister to *Polyspatha* and due to *Rhopalephora* being nested within *Aneilema* s.str. Furthermore, of the seven sections proposed by Faden [29], *A.* sect. *Amelina* was recovered as polyphyletic due to *A. johnstonii* being recovered as sister to all species of *Aneilema* s.str., and *A. gillettii* being nested in *A.* sect. *Pedunculosa*. *Aneilema* sect. *Lamprodithyros* is also rendered as paraphyletic due to *A. indehiscens* subsp. *keniense* Faden being nested in *A.* sect. *Brevibarbata*. Added to that, none of the recovered relationships between the proposed sections of *Aneilema* was deemed, by Kelly & Evans [70], to make any morphological sense. The present morphology-based hypothesis for *Aneilema* lends strong morphological support for the results of Kelly & Evans [70] by being almost completely congruent, in addition to being further resolved, and more statistically well-supported than the molecular one. For *Commelina*, the present study confirms that *Tapheocarpa* is nested within it and that it should be reduced to synonymy. However, relationships within *Commelina* remain poorly supported and, thus, preliminary. However, a phylogenetic analysis of *Commelina* is currently being carried out (Pellegrini et al., in prep.), sampling over a third of the currently recognised species. This study recovers a different and very unexpected topology that seems to indicate a South American origin to this African-centred genus. Furthermore, due to how peculiar the morphology of *Commelina* is, even when compared to closely related genera such as *Aneilema*, the inclusion of several taxonomically relevant characters for *Commelina* would have added unnecessary noise and missing data. Thus, I chose to include only a minimal sampling of *Commelina* species, as well as to not include most *Commelina*-specific characters.

## 4. Materials and Methods

### 4.1. Taxon Sampling and OTU

The dataset is represented by 400 taxa, 398 of Commelinales and two species of Zingiberales as the outgroup, represented by *Zingiber officinale* L. (Zingiberaceae) and *Costus pulverulentus* L. (Costaceae). The choice of Zingiberales as the outgroup seems obvious, since the order is consistently recovered as sister to Commelinales [1,2,3,4,5,6,7,8,9,10,11,12,13]. The ingroup sampling aimed to reflect, as best as possible, the morphological and ecological diversity and variation observed within each genus and lineage. This initial sampling was complemented by adding the type species for all infrafamilial ranks currently accepted in Commelinales. This nomenclatural approach to sampling was made to ensure the obtained result could be properly assessed from a taxonomic and systematic perspective. Finally, the present study samples 398 taxa of Commelinales (ca. 40% of ca. 978), including all 59 genera currently accepted for the order [14]. Out of this total, the ingroup is represented by ca. 31% of the accepted Commelinaceae species (244 spp., plus one subspecies, out of ca. 800), ca. 76% of Haemodoraceae (92 spp., plus 5 subspecies, out of ca. 120), ca. 43% of Hanguanaceae (10 spp. out of ca. 23), 100% of Philydraceae (i.e., eight spp.), and 100% of Pontederiaceae (47 spp.) (Table S1).

Since one of the present study’s main objectives is to investigate and test the monophyly of all supraspecific ranks in Commelinales, individual species or infraspecific taxa are treated as the OTU. Therefore, the only a priori assumptions of monophylly for the present study are those for Commelinales and of our terminals (i.e., species or infraspecific taxa). This ensures that the investigated units all descended from a most recent common ancestor and represent distinct evolutionary entities, without biasing or forcing the monophylly of any taxonomic rank above the OTU. It also decreases the chances of analysis of artefacts caused by polyphyletic OTU, which could result in inaccurate and misleading evolutionary reconstructions [200,201].

### 4.2. Character Selection, Coding, and Scoring

Around 150 characters used in the present analysis were originally selected and coded by previous studies [19,25,28,40,69,114,132,193,196]. However, around 450 characters were coded and scored, or had their scoring and coding modified and/or updated, especially for the present study, for the first time. Character coding followed the recommendations of Sereno [202] for morphological phylogenies. Primary homology hypotheses [203] were proposed for root, stem, leaf, inflorescence architecture, floral, fruit, seed, seedling, palynological, anatomical, cytological, and phytochemical characters. A total of 600 discrete macro- and micromorphological, ecological, palynological, anatomical, cytological, and phytochemical characters were scored, being treated as unordered and equally weighted. Out of the 600 characters, 410 were macromorphological, 12 were ecological, 33 were palynological, 15 were for seedling morphology, 110 were anatomical, four were cytological, and 16 were phytochemical (Table S2). The complete morphological matrix is provided in Table S3.

### 4.3. Data Collection, Morphology, and Terminology

Specimens from the following herbaria were analyzed: AD, AAU, ALCB, ASU, B, BA, BAF, BBS, BHCB, BHZB, BKL, BLH, BM, BOL, BOTU, BR, BRI, BRIT, BRLU, C, CAL, CANB, CAS, CASAT, CAY, CBG, CEN, CEPEC, CESJ, CGE, CGMS, CICY, CIIDIR, CLF, CM, CNMT, COI, COL, COR, CORD, CR, CTES, CVRD, DR, DS, E, EA, EAC, ESA, F, FB, FCAB, FCQ, FLOR, FMB, FR, FURB, G, GBH, GENT, GH, GMUF, GOET, GUA, HA, HAL, HAMAB, HAS, HB, HBG, HBR, HDCF, HEM, HERBAM, HNMN, HRB, HRCB, HSTM, HUAP, HUCS, HUEFS, HUFSJ, HULE, HUPG, HURB, IAC, IAN, IBE, IBUG, ICN, INB, INPA, IPA, J (incl. BNRH), JAR, JBSD, JOI, K, KANU, KEP, KYO, L, LE, LG, LISC, LIL, LL, LOJA, LP, LPB, M, MA, MAPR, MBM, MBML, MEL, MEXU, MG, MICH, MIN, MO, MPUC, MVM, MY, NBG, NBYC, NDG, NEU, NGCPR, NH, NHA, NO, PB, PERTH, NBG, NSW, NU (incl. NPB, PCE, and UNDH), NX, NY, OS, P, PACA, PERTH, PH, PMSP, PORT, PR, PRC, PRE, QMEX, R, RB (incl. GUA), RFA, RFFP, S, SAR, SCP, SI, SMU, SP, SPF, SPSF, SRGH, TANG, TEFH, TEX, U, UAMIZ, UC, UCR, UEC, UFRN, UJAT, UMO, UMSA, UNA, UNDH, UPCB, UPRRP, US, USF, USZ, V, VDB, VEN, VIC, VIES, VRJ, VT, W, WAG, WSY, WU, XAL, and ZT (herbaria acronyms according to Thiers, continuously updated [204]). The morphology for all investigated taxa is based on a combination of cultivation, fresh, spirit and herbarium samples, complemented by field annotations, observations of wild and cultivated specimens, photographs, and all available literature. I have personally observed and/or collected over 2/3 of the investigated taxa either in cultivation or during several fieldtrips around the globe, from 2008 to the present. I have studied at least two specimens for each taxon, with the most representative one chosen as the voucher specimen (Table 1).

### 4.4. Terminology

The indumentum and shape terminology follow Radford et al. [205]; inflorescence terminology and morphology follow Weberling [206,207], Panigo et al. [69], and Pellegrini and Horn [24]; fruit terminology follows Spjut [208]; seed terminology follows Faden [29]; anatomical terminology follows Tomlinson [130,131,132,133,178,179,209]; and general morphology for each of the families follows Faden [29,30] and Pellegrini [28] for Commelinaceae, Simpson [19,20] for Haemodoraceae, Bayer et al. [21] for Hanguanaceae, Hamann [22,23] for Philydraceae, and Pellegrini et al. [25] for Pontederiaceae. Finally, whenever necessary, terminology is standardised in the present study to ensure consistency.

### 4.5. Phylogenetic Analysis

Data were entered into a matrix of characters per taxa using the software Mesquite 3.20 [210] (Table S3). All characters were treated as unweighted and unordered. MP analysis was performed using PAUP* 4 [211], with a heuristic search with 10,000 random taxon additions and TBR branch swapping. CI, RI, HI, and RC were used to assess the degree of homoplasy in the dataset, using character optimisation of ACCTRAN [212]. Statistical support for each branch of the cladogram was evaluated with BS analyses with 1000 random addition replications. The search parameters used to estimate the bootstrap values were the same as those of the initial heuristic search. BA was conducted with mixed models and unlinked parameters, using MrBayes 3.1.2 [213]. MCMC was performed using two simultaneous independent runs with four chains each (one cold and three heated), saving one tree every 1000 generations, for a total of ten million generations. I excluded as ‘burn-in’ trees from the first two million generations, and tree distributions were checked for a stationary phase of likelihood. PP of clades were based on the majority-rule consensus, using the remaining trees, calculated with MrBayes 3.1.2. Posteriorly, Mesquite 3.20 was used to reconstruct the ancestral character states, while WinClada ver. 1.0000 [214] was used to trace the synapomorphic characters over the consensus tree.

## 5. Conclusions

This is the first morphological phylogenetic hypothesis for an order of Monocots, and only the third for an order of flowering plants (i.e., Nymphaeales [215,216,217,218,219], Zingiberales [40,130,196]). However, this is the largest morphological dataset ever compiled for land plants. This monumental effort and dataset produced an almost completely congruent topology to the available molecular data, debunking the claim that morphological characters are unreliable for phylogenetic inferences in plants, especially in Commelinales [44,194]. Homoplasy is an unavoidable part of phylogenetic analyses, be they molecular or morphological, and needs to be embraced by botanical phylogeneticists, instead of being demonised. Without morphology, classification systems and taxonomy become relative and pointless, since recognised ranks and taxa are no longer diagnosable. Instead of the current trend of diminishing morphology, botanists need to embrace and revisit it. The current dogmatic and blind trust in molecular data can cause obviously unreliable molecular results to be taken at face value, without any critical thinking [197]. For instance, in Commelinales, the topology recovered by Zuntini et al. [44] is greatly congruent to the one recovered in the present and previous studies. These authors claim that most genera recognised in Commelinales are non-monophyletic, based on their results. Nonetheless, taxonomic incongruences in their topology generally result from misidentified samples or low-quality sequences. Finally, the present study empirically proves that the genera of Commelinales can be easily circumscribed and differentiated from each other by a reliable combination of morphological characters, which are further supported by molecular evidence.

## Figures and Tables

**Figure 17:**
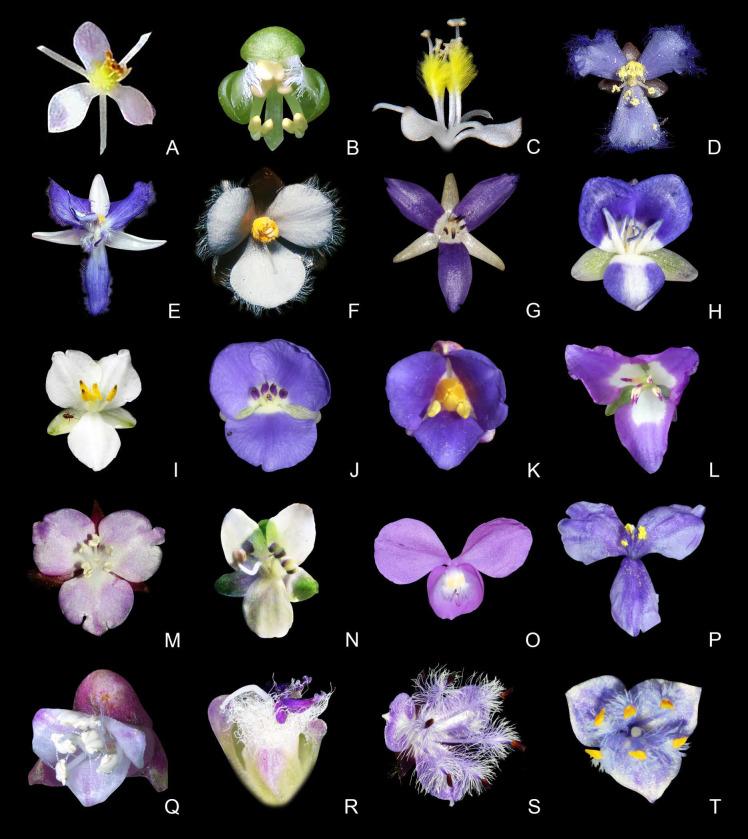
Floral morphology of Commelinaceae. (**A**), *Spatholirion calcicola* K.Larsen & S.S.Larsen; (**B**), *Aëtheolirion stenolobium* Forman; (**C**), *Streptolirion volubile* Edgew.; (**D**), *Geogenanthus rhizanthus* (Ule) G.Brückn; (**E**), *Cochliostema odoratissimum* Lem.; (**F**), *Plowmanianthus panamënsis* Faden & C.R.Hardy; (**G**), *Dichorisandra acaulis* Cogn.; (**H**), *D. hexandra* (Aubl.) C.B.Clarke; (**I**), *D. incurva* Mart.; (**J**), *D. penduliflora* Kunth; (**K**), D. thyrsiflora J.C.Mikan; (**L**), *D. radicalis* Nees & Mart.; (**M**), *Siderasis fuscata* (Lodd.) H.E.Moore; (**N**), *S. zorzanellii* M.Pell. & Faden; (**O**), *Nivoanthus synantherus* (H.Perrier) M.Pell.; (**P**), *Coleotrype natalensis* C.B.Clarke; (**Q**), *Amischotolype hispida* (A.Rich.) D.Y.Hong; (**R**), *A. microphylla* (Y.Wan) C.K.Lee et al.; (**S**), *Cyanotis ciliata* (Blume) Bakh. f.; (**T**), *C. repens* Faden & D.M.Cameron. A by T. Thitimetharoch, B by T. Sando, C by J. Lundberg, D by D. Scherberich, E by R. Aguilar, F by C.R. Hardy, G–H, J–M, P & T by M.O.O. Pellegrini, I by G. Shimizu, N by J.P. Zorzanelli, O by C. Rakotavao, Q & S by R. Schneider, and R by E. Barbier.

**Figure 18:**
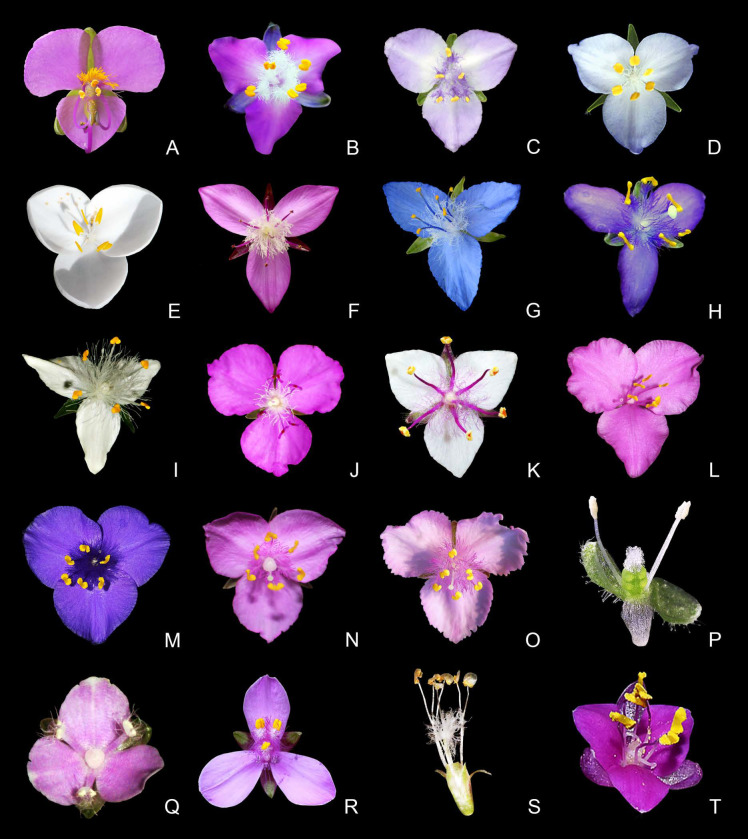
Floral morphology of Commelinaceae. (**A**), *Tinantia erecta* (Jacq.) Fenzl; (**B**), *Thyrsanthemum floribundum* (M.Martens & Galeotti) Pichon; (**C**), *Gibasoides laxiflora* (C.B.Clarke) D.R.Hunt; (**D**), *Matudanthus nanus* (M.Martens & Galeotti) D.R.Hunt; (**E**), *Weldenia candida* Schult. f.; (**F**), *Elasis hirsuta* (Kunth) D.R.Hunt; (**G**), *Ivoniella guatemalensis* (C.B.Clarke ex Donn.Sm.) M.Pell.; (**H**), *Gibasis matudae* D.R.Hunt; (**I**), *Tradescantia fluminensis* Vell.; (**J**), *T. commelinoides* Schult. f.; (**K**), *T. ambigua* Mart. ex Schult. & Schult. f.; (**L**), *T. sillamontana* Matuda; (**M**), *T. ohiensis* Raf.; (**N**), *Huntiella navicularis* (Ortgies) M.Pell. (**O**), *Cuthbertia ornata* Small; (**P**), *Aploleia monandra* (Sw.) H.E.Moore; (**Q**), *Tripogandra triandra* (Kunth) M.Pell. & Handlos; (**R**), *T. diuretica* (Mart.) Handlos; (**S**), *Callisia repens* (Jacq.) L.; (**T**), *Hadrodemas warszewiczianum* (Kunth & C.D.Bouché) H.E.Moore. A by E. Barbier, B & D by A. García, C by A. Jonker, E by S. Cross, F by A. Kay, G by P. Acevedo-Rodríguez, H by M. Costea and I. García-Ruiz, I, K, L–M & Q–S by M.O.O. Pellegrini, and T by C. Willemsen.

**Table 1:** All recovered exclusive (**bold**) and relevant homoplastic synapomorphic characters for all clades or taxa from order to family.

Rank	Taxon	Statistical Support (BS/PP)	Synapomorphies
order	Commelinales	100/1	**Closed leaf-sheaths with a suture scar (37:1)**, **accessory bracts with primordial inflorescence buds (91:1)**, secondary inflorescence branches pedunculate (101:1), **flowers enantiostylous (142:1)**, **flowers with three septal nectaries (157:1)**, **outer antesepalous stamen present (250:1)**, **inner antepetalous stamen present (253:1), pollen released with adhering raphides (343:1)**, **pollen grains sulcate (354:1)**, **pollen grains with rounded tectal elements (366:1)**, **fruits dry (416:1)**, seed testa scrobiculate to rugose (440:1), **seedlings with rhizoids (458:1)**, **stomata with terminal and lateral neighbouring cells equal in size (508:1)**, **leaf mesophyll with calcium oxalate raphides (517:1)**, **tannin cells in the floral receptacle (529:1) and perianth (531:1)**, **endothecium basally thickened (542:1)**, **tapetum with raphides (544:1)**, **seed coat bitegmic (570:1)**, and **copious endosperm (578:1)**
suborder	Pontederiineae/unifacial clade	99/1	**Ptyxis conduplicate (27:2)**, leaves unifacial (28:1) and **equitant (40:2)**, perianth tubular (164:1), **perianth whorls fused together (165:1)**, outer perianth whorl petaloid (168:1) and membranous (202:0), pollen exine verrucate (369:6), **seeds ovoid or fusiform to barrel-shaped (432:5)**, **primary root sinuate to spirally-coiled (456:2)**, **cotyledon assimilating (463:1) with bifacial hyperphyll (466:1)**, **primary leaves bifacial and ribbon-like (469:2)**, **bean-shaped starch grains present (471:1)**, stomata with 2 neighbouring cells (507:0), **xylem and phloem alternate or circular phloem with central xylem or xylem abaxial (514:1)**, **endothecium with styloid crystals (520:1)** and granular (533:1) and fibrillar tannin cells (534:1), ovary wall (546:1) and septae with tannin cells (552:1), **ovules multiseriate (554:2)**, **placenta 2-flanged (557:2) and with sclereids (558:1)**, **embryo ca. as long as the seed (576:2)**, **endosperm helobial-chalazal (577:2)**, **haploid chromosome count x = 7 (580:2)**, the presence of pro-anthocyanins (585:1), **diferulic acids (588:1)**, and **p-coumaric acids (600:1)**
family	Philydraceae	99/1	Bracteoles spathaceous (127:5), flowers campanulate (164:3), **perianth pseudotetramerous (184:2)**, inner antesepalous and antepetalous stamens absent (251–253:0), filament fused with the inner perianth lobes (257:2) and flattened (261:4), **seeds with dactyliform projections arranged in longitudinal striae (443:2)**, **chalazal cap enlarged (445:1)**, perianth with abundant tannin cells (535:2), endothecium not thickened (542:0), **tapetum glandular (543:1)**, ovary aposeptalous (548:0), placentation intrusive-parietal (555:2), and haploid chromosome count x = 8 (580:3)
superfamily	Pontederiorae/Haemodoraceae + Pontederiaceae	70/1	Septal nectaries present (156:1), **hypanthium present (161:1)**, when present perianth with nectar guides consisting of spots, blurs or gradients (214:1), **when present nectar guides yellow (215:3)**, stamens dimorphic (249:1), **pollen inner layer papillate or baculate (359:1)**, **lacking an infratectum (363:0)**, **tectum baculate (364:1)**, **sulcal membrane verrucate (374:5)**, **seed testa longitudinally winged or crested (440: 5)**, **stomata neighbouring cells with oblique divisions (506:0)**, **seed coat with calcium oxalate crystals (573:2)**, and **phenylphenalenones present (590:1)**
family	Haemodoraceae	81/1	Roots sand-binding (11:1) and with **arachnoid hairs (12:2)**, **leaf-blade fibrous and coriaceous (47:3)**, **inflorescence secondary branches a branched cyme (95:2)**, outer perianth whorl succulent or fleshy (202:2), when present nectar guides orange to red (215:4) and **consisting of three spots (216:2)**, ovary inferior (390:0)/ovary late-inferior (390:3) *, seeds wider than long or as wide as long (431:1), **seedlings with unifacial primary leaves (469:0**), root pith sclerified (474:1), vascular bundles in the stems with a fibrous layer (477:1), uniseriate macrohairs absent (483:0), multiseriate tapering hairs present (491:1), placenta unflanged (557:0), and **chelidonic acid present (587:1)**.
family	Pontederiaceae	99/1	**Leaves dimorphic (26:1)**, **ptyxis conduplicate-involute (27:3)**, **leaves late-bifacial (28:2)**, ligule present (29:1), immature leaves submerged (42:0), **mature leaves always produced (63:2)**, inflorescence deflexed at post-anthesis and in fruit (83:1), basal bract spathaceous (84:2), inflorescence with secondary branches long-pedunculate (101:1, 102:1), contracted axis (106:0) and ebracteate (111:0), bracteoles absent (127:0), septal nectaries interlocular (158:1), perianth whorls fused forming a conspicuous tube (166:1), persistent perianth longer than the fruit (204:2), **nectar guide on the posterior perianth lobes (213:1)**, filaments curved (274–277:1) and pubescent (279:1, 284:1, 290:1, 295:1) with glandular macrohairs (281:0, 286:0, 292:0, 297:0), **pollen grains bisulcate (355:2)**, **style pubescent with glandular macrohairs (403:2)**, **superior ovary anthocarp present (411:1)**, **cotyledon sheath ligulate (465:3)**, **xylem abaxial and phloem adaxial near the margin of the blades plus xylem and phloem alternate near the centre of the blade (514:2)**, leaf mesophyll (521:1) and floral receptacle (527:1) aerenchymatous, perianth lacking fibrillar tannin cells (534:0), perianth with moderate homogenous and/or granular tannin cells (535:1), **perianth aerenchymatous (536:1)**, ovary walls lacking silica crystals (545:0), **ovary walls aerenchymatous (547:1)**, ovary hemiseptalous (548:1), and ovary septae lacking tannin cells (552:0)
suborder	Commelinineae/bifacial clade	71/0.99	Leaves subpetiolate (43:1), flowers without display torsion (144:0), **inner perianth whorl deliquescent at post-anthesis (177:2) ♦**, perianth whorls similar in shape to each other (208:0), androecium actinomorphic (248:0), filaments free from the perianth (257:0), antepetalous filaments equal in length (270:1), seeds wider than long or as wide as long (431:1), **seeds with lateral wings restricted to the margin of the longer seed axis (435:1) †**, **cotyledon non-chlorophyllate (462:1)**, **seedlings with at least the first primary leaf modified into a cataphyll (468:1)**, **silica bodies present in the leaves (470:1)**, **leaf mesophyll with raphide canals (518:1)**, **staminal primordia originating from the petal primordia (540:1)**, ovules orthotropous (560:0) and pleurotropous (561:2), **seed coat sclerified (572:1)**, and **haploid chromosome count x = 29 (581:1) †**
family	Hanguanaceae	100/1	**Plants dioecious (4:1)**, stems fibrous (20:0), **leaf-sheaths with scarious margins (36:1)**, inflorescences with two to several secondary branches per node (99:1), flowers clustered (137:1) and all unisexual (140:3), perianth homochlamydeous (167:0), **inner perianth whorl sepaloid (169:0)**, **perianth remaining herbaceous at post-anthesis (177:3)**, outer perianth whorl herbaceous (202:3), medial and lateral sepals rhomboid to orbicular (224:3, 225:3), petals green (237:4, 246:4), stamens dimorphic (249:1), anther latrorse (337:0), pollen inaperturate (354:0), **nectariferous scales present (375:1)**, **pistilodes with nectariferous lobes (386:1)**, ovary locules 1-ovulate (395:1, 396:1), **style absent in pistillate flowers (397:0)**, fruits fleshy (416:0), subglobose to globose (417:2), lustrous (424:1) and indehiscent (425:0), seeds ventrally concave or wedge-shaped (437:4), testa smooth (440:0), **seedlings with primary root brown (457:1)**, uniseriate macrohairs absent (483:0), **multiseriate fruticose hairs present (493:1)**, leaf mesophyll lacking calcium oxalate raphides (517:0), **endothecium medially thickened (542:3)**, tapetum raphides absent (544:0), **ovary locules with mucilage-secreting colleter hairs (553:1)**, **placenta blanket-like (556:5)**, **seed coat with two layers of crossing fibres (574:1)**, **seeds tenuinucellate (579:1)**, and **haploid chromosome count x = ca. 85 (581:1)**
family	Commelinaceae	91/1	Roots sand-binding (11:1), internodes with a leaf-opposed line of uniseriate hairs (23:1), **nodes swollen (24:1)**, leaf-sheaths closed asymmetric (35:2), sepals free (217:0), filaments thin (260:0), outer antesepalous filament pubescent (279:1), seedling collar present (459:1), coleoptile present (465:1), vessels in both the roots and stems (472:1), **stems with a nodal vascular plexus (475:1)**, perianth with moderate tannin cells (535:1), **outer seed tegmen thin and sloughing off (571:1)**, **seed coat with silica crystals (573:1)**, **embryo of the Xyris-Scirpus or grass type (575:1)**, and **presence of acylated cyanidin 3,7,3′-triglycoside anthocyanins (586:0)**

Character numbers and states are presented between brackets, separated by a colon, i.e., “character name (character number:state number)”. Character and state numbers as presented in Table S2. BS—bootstrap support; PP—posterior probability. * treated as an exclusive synapomorphic when coded a posteriori as “390. Gynoecium, ovary, position: inferior (0); superior (1); half-inferior (2); late-inferior (3)”. ♦ Coding artefact, actually represents an exclusive synapomorphy for Commelinaceae. † coding artefact.

**Table 2:** Summary of recovered exclusive (**bold**) and relevant homoplastic synapomorphic characters for all clades or taxa below family and above genus in Philydraceae and Haemodoraceae.

Rank	Clade/Taxon	Statistical Support (BS/PP)	Synapomorphies
–	*Philydrum* + *Orthothylax* + *Helmholtzia* s.str.	82/0.53	9(1), 20(0), 22(0), 78(1), 134(1), **139(5)**, **187(1)**, **188(1)**, 369(7), 391(1), 407(3), 417(2), **432(7)**, **440(7)**, 559(1), 563(1)
–	*Orthothylax* + *Helmholtzia* s.str.	91/0.92	127(2), 180(1), **187(2)**, 202(1), 257(0), 261(0), 311(1), 394(2), 398(0), 418(0), 422(1), 424(1), 548(1), 555(0), 580(7)
subfamily	Haemodoroideae	97/1	16(1), 243(0), 250(0), 251(0), 257(0), 260(0), 272(0), 273(2), 402(1), 407(3), 417(2), 432(0), 436(0), 437(0), **440(8)**, **457(2)**, 463(0), **465(2)**, 467(0), **495(1)**, **496(1)**, 515(1), 529(0), 531(0), 558(0)
–	Xiphidieae + Wachendorfieae	87/1	127(2), 139(1), 144(0), 157(0), **158(0)**, **164(2)**, **166(2)**, 198(2), 201(0), 205(0), 208(1), 212(1), 234(3), 236(2), 237(0), 245(2), 246(0), **357(4)**, 359(1), 390(1), **393(2)**, 418(0), **421(1)**, 424(1)
tribe	Xiphidieae	87/1	127(4), 156(0), 164(3), 180(1), **210(1)**, 260(1), 311(1), 319(0), 320(0), 344(1), **392(4)**, 399(1), 422(1), **443(1)**, 491(0), 558(1)
–	*Cubanicula* + *Pyrrorhiza*	52/0.98	398(1), 417(0), 433(2), 447(1), 556(1)
tribe	Wachendorfieae	88/1	11(0), 12(0), 138(1), **159(1)**, **166(3)**, **176(1)**, 197(2), 204(1), 224(4), 243(3), 276(1), 277(1), 313(0), 378(0), 407(1), **417(5)**, 436(1), 437(1), 580(7)
tribe	Haemodoreae	94/1	**96(1)**, 145(0), 153(0), 200(1), 217(0), **392(1)**, 433(2), 434(1), 439(1), **440(9)**, 556(1), 580(3)
–	*Lachnanthes* + *Haemodorum*	95/1	2(1), 76(1), 130(0), 158(1), 197(0), **202(5)**, 234(0), 237(4), 246(4), 257(1), 358(0), 401(0), 402(0), **404(1)**, **417(7)**
subfamily	Conostylidoideae	82/1	41(0), 142(0), **173(1)**, 248(0), 249(0), 269(1), 270(1), 271(1), **354(2)**, 369(4), 387(1), **392(2)**, 401(0), **492(1)**, 522(1)
tribe	Tribonantheae	100/1	2(1), 12(0), 17(2), 22(0), 47(4), 48(6), 49(1), 59(2), 90(0), 92(0), 98(0), 100(2), 101(0), 102(1), 106(0), 111(0), 127(4), 131(1), 135(0), 136(0), 145(0), 164(0), 217(0), **264(2)**, 302(1), 303(1), 305(1), 307(1), 309(1), 311(1), 317(1), 318(1), **356(1)**, **390(2)**, **392(5)**, **432(8)**, 440(2), 477(0), 521(1)
tribe	Conostylideae	67/0.95	5(1), 9(1), 41(2), 55(2), 78(1), 139(1), 224(1), 225(1), 237(0), 246(0), 319(0), 320(0), **361(1)**, **399(0)**, 431(0), **494(1)**
(tribe)	*Anigozanthos* s.lat. + *Conostylis* s.lat./Conostylideae s.str.	63/–	146(1), 166(1), 237(3), 246(3), 580(1)

Character numbers are presented followed by the states between brackets, i.e., “character number (state number)”. Character and state numbers as presented in Table S2. BS—bootstrap support; PP—posterior probability.

**Table 3:** Summary of recovered exclusive (**bold**) and homoplastic synapomorphic characters for all clades at genus rank for Philydraceae, Pontederiaceae, Haemodoraceae, and Hanguanaceae.

Family	Clade/Taxon	Statistical Support (BS/PP)	Synapomorphies
Philydraceae	*Philydrella*	100/1	2(1), 11(1), 17(2), 47(2), 48(6), 62(2), 90(0), 98(0), 146(1), **186(2)**, **189(2)**, **258(1)**, 260(0), 274(1), 303(1), 328(4), 335(2), 399(1)
Philydraceae	*Philydrum*	96/0.99	47(4), 49(1), 190(1), 328(3), 342(1), 417(6), **441(1)**, 521(1), 527(1)
Philydraceae	*Orthothylax*	100/1	**177(5)**, 180(2), **186(0)**, 391(0), 394(3), 419(0)
Philydraceae	*Helmholtzia* s.str.	99/1	**6(3)**, 114(2), 164(6), 166(1), **185(1)**, **191(0)**, **194(1)**, **257(3)**, 311(2), 344(0), 416(0), 425(0), **441(2)**, 548(2), 580(8)
Pontederiaceae	*Heteranthera*	96/1	6(1), **11(2)**, 19(0), 40(1), 41(0), 92(0), 98(0), 135(0), 141(1), 156(0), 214(0), 224(4), 236(0), 242(0), 245(2), 252(0), 253(0), **254(1)**, 271(2), 272(2), 273(0), 344(1), 398(1), 399(1), 401(0), **407(6)**, 520(0), **528(0)**, 555(2)
Pontederiaceae	*Pontederia*	99/1	**31(0)**, 39(0), 62(0), 66(1), **69(1)**, **70(1)**, 73(1), 76(1), 77(0), **94(1)**, 142(0), **143(2)**, 153(2), 164(4), 170(1), **177(0)**, 203(1), 206(0), 241(1), 243(3), 255(1), 260(0), 282(2), 287(2), 317(1), 318(1), 353(1), 402(1), **412(1)**, **414(1)**, **537(1)**, **549(2)**, 564(2), 580(7)
Haemodoraceae	*Xiphidium*	98/1	48(1), 80(1), 95(0), 333(1), 416(0), 421(0), 425(0), 431(2), **432(6)**, 437(3), 440(6)
Haemodoraceae	*Cubanicula*	100/1	5(1), 196(1), 199(0), 206(2), 208(2), 243(3), 277(2), **313(1)**, 422(0), 424(0)
Haemodoraceae	*Pyrrorhiza*	100/1	17(2), 101(0), 127(5), **130(6)**, 139(2), 142(0), 145(0), 166(0), 198(1), 212(0), 234(0), 236(1), 245(1), 253(0), 491(1)
Haemodoraceae	*Schiekia*	99/1	16(0), **276(3)**, 302(1), **332(1)**, 357(0), **358(2)**, 369(7), 371(1), **377(1)**, **432(9)**, 440(1), 561(2)
Haemodoraceae	*Wachendorfia* s.lat.	91/1	2(1), 17(2), **48(3)**, 106(0), 136(0), 139(2), 139(2), 202(0), 205(2), 225(2), 237(3), 246(3), 395(1), **513(1)**, 515(0)
Haemodoraceae	*Barberetta*	100/1	5(1), 25(1), 47(1), 95(0), 102(1), 166(0), 176(0), 217(0), 225(4), 234(4), **236(3)**, 243(4), 245(3), 394(3), 420(1), 425(0), 426(0), 432(4), 440(0), 477(0), 491(0), 555(4), **556(6)**
Haemodoraceae	*Wachendorfia* s.str.	–/–	138(0), 196(1), 198(0), 206(2), 215(0), 224(2), 313(1), 396(1), 437(0)
Haemodoraceae	*Dilatris* s.lat.	98/1	164(5), 224(1), 225(1), 243(1), 277(1), 313(0), 395(1), 396(1), 450(1), 515(0), **523(1)**, 580(5)
Haemodoraceae	*Dilatris* s.str.	97/1	48(1), 77(0), 146(1), 152(2), 202(0), **229(2)**, 237(3), **238(2)**, 246(3), **247(3)**, 417(1), **418(3)**, 432(1)
Haemodoraceae	*Paradilatris*	99/1	5(2), 139(1), 180(2), **207(1)**, **407(8)**, **411(2)**, **425(5)**
Haemodoraceae	*Lachnanthes*	100/1	11(0), 12(0), 25(1), 47(4), 92(0), 146(1), 197(2), 200(2), 311(2), 322(1), **334(1)**, **425(6)**, 472(1), 495(0), 521(1), 561(2)
Haemodoraceae	*Haemodorum*	99/1	100(3), 127(2), **131(6)**, 249(0), 319(2), 320(2), 515(0)
Haemodoraceae	*Tribonanthes*	100/1	2(1), 12(0), 17(2), 22(0), 47(4), 48(6), 49(1), 59(2), 90(0), 92(0), 98(0), 100(2), 101(0), 102(1), 106(0), 111(0), 127(4), 131(1), 135(0), 136(0), 145(0), 164(0), 217(0), **264(2)**, 302(1), 303(1), 305(1), 307(1), 309(1), 311(1), 317(1), 318(1), **356(1)**, **390(2)**, **392(5)**, **432(8)**, 440(2), 477(0), 521(1)
Haemodoraceae	*Phlebocarya*	99/1	81(1), 156(0), 164(5), 173(0), 180(1), 313(0), 335(2), **336(2)**, 337(2), **338(2)**, **362(1)**, 394(0), 395(1), 396(1), 425(0), 426(0), 548(0), 555(4), **556(7)**, **561(0)**
Haemodoraceae	*Anigozanthos* s.lat.	95/1	9(0), 55(0) 139(3), 158(1), **174(1)**, 187(3), 208(2), **229(5)**, 248(1), 255(1), 260(0), 399(2), **556(3)**
Haemodoraceae	*Macropidia*	100/1	**175(1)**, 234(4), 237(8), 243(4), 246(8), 395(1), 396(1), **426(2)**
Haemodoraceae	*Anigozanthos* s.str.	–/–	106(0), 311(1), 387(2)
Haemodoraceae	*Conostylis* s.lat.	72/1	78(0), 153(0), 164(6), 478(1), **504(1)**, **505(1)**, 580(3)
Haemodoraceae	*Blancoa*	100/1	139(2), 153(3), **160(1)**, 164(1), 180(4), 197(2), 455(1), 515(1)
Haemodoraceae	*Conostylis* s.str.	–/–	5(0), 92(0), 100(3), 101(0), 106(0), 311(4), **556(2)**, 580(0)
Hanguanaceae	*Hanguana*	100/1	**4(1)**, 20(0), **36(1)**, 43(1), 99(1), 137(1), 140(3), 167(0), **169(0)**, **177(3)**, 197(0), 201(1), **202(3)**, 224(3), **225(3)**, 237(4), 246(4), 249(1), 337(0), 354(0), 366(0), **375(1)**, **386(1)**, 395(1), 396(1), **397(0)**, 416(0), 417(2), 424(1), 425(0), 437(4), 440(0), **457(1)**, 483(0), **493(1)**, 517(0), **542(3)**, 544(0), **553(1)**, **556(5)**, 574(1), **579(1)**, **581(2)**

Character numbers are presented followed by the states between brackets, i.e., “character number (state number)”. Character and state numbers as presented in Table S2. BS—bootstrap support; PP—posterior probability.

**Table 4:** Summary of recovered exclusive (**bold**) and relevant homoplastic synapomorphic characters for all clades or taxa below family and above genus in Commelinaceae.

Rank	Clade/Taxon	Statistical Support (BS/PP)	Synapomorphies
subfamily	Cartonematoideae	99/1	2(1), 17(0), 46(0), 48(1), 50(0), 89(0), 90(0), 130(1), 145(0), 153(1), 200(2), 203(1), 204(2), **221(2)**, 237(3), 246(3), 297(1), 311(2), 313(0), 335(1), 336(1), 338(1), **341(2)**, 407(3), 450(1), **461(1)**, **483(4)**, 507(0), **516(1)**, **519(1)**, 585(1)
tribe	Cartonemateae	94/1	17(2), 27(0), 47(1), 136(0), 146(1), 197(5), 279(0), 333(1), 395(2), 396(2), 418(2), 432(3), **433(1)**, 518(0)
tribe	Triceratelleae	100/1	1(1), 3(0), 19(0), 21(1), 41(0), 79(1), 81(2), 116(0), 234(3), 243(3), 261(4), 284(1), 290(1), 295(1), **436(2)**, 437(4), 440(3), 448(0), 497(1), 554(1)
subfamily	Commelinoideae	67/1	5(1), 14(1), 44(1), 56(1), 106(1), 117(1), 140(1), 142(0), 180(1), 236(1), 245(1), 248(1), **281(3)**, 282(1), 298(1), 303(1), 305(1), 307(1), 318(1), 419(0), 432(1), 440(2), 455(0), **460(2)**, **476(0)**, **479(1)**, 580(5), 582(2)
–	Palisoteae + Commelineae	–/0.92	61(0), 127(2), 139(3), 145(1), 152(1), 249(1), 271(0), 272(0), 273(2), 281(4), 318(2), 345(0), 353(0), 424(1), **464(0)**, 467(2), 508(0), **565(5)**, **586(2)**
tribe	Palisoteae	100/1	**40(3)**, 47(1), 117(0), 129(0), 138(1), 140(3), 152(2), 153(1), 167(0), 168(1), 200(1), 201(1), 202(0), 208(0), 224(4), 225(4), 234(4), 235(2), 243(4), 244(1), 282(2), 283(1), 284(1), 286(4), 287(2), 288(1), 307(0), 347(0), 352(0), **353(2)**, 399(2), **403(4)**, 416(0), 417(1), 422(1), 425(0), 432(0), 437(1), 440(0), 448(0), 450(1), **473(1)**, **483(5)**, **485(1)**, 591(1)
tribe	Commelineae	99/1	14(0), 56(0), 60(1), **128(3)**, 131(2), **132(1)**, 142(1), 198(0), 205(0), 226(1), **228(1)**, 269(0), 270(2), 271(3), 278(1), 279(0), 309(1), 337(0), 344(1), **351(0)**, 366(0), 371(1), 373(1), **374(3)**, 378(0), **427(1)**, 446(1), 447(1), 453(1), 454(3), **488(1)**, **507(2)**, 560(1), 580(2)
(subtribe)	Murdanniinae s.lat.	–/–	7(1), 92(0), 139(2), 199(0), 232(1), 234(0), 272(1), 345(1), **357(3)**, **367(2)**, **372(0)**, **380(1)**, 387(0), 432(4), 444(1), **467(1)**, **480(0)**, 597(1)
subtribe	Murdanniinae s.str.	100/1	2(1), 5(0), 27(0), 44(2), 47(2), 48(1), 50(0), 59(1), 60(0), 99(1), 117(0), 131(1), 180(3), 205(1), 226(0), 243(0), 248(0), 269(1), 270(1), 271(2), 272(2), 273(0), 286(0), 287(1), 292(0), 293(1), 317(2), 349(0), 353(0), **481(4)**, 498(1), **525(1)**, 580(5), 592(1)
subtribe	Pseudoparidinae	100/1	14(1), 59(0), 61(1), 82(2), 92(2), 154(0), 206(2), 234(2), **236(4)**, 237(0), **245(4)**, 246(0), 251(0), 269(2), 278(0), 304(1), 310(1), 328(4), 331(4), 387(2), 425(0), 554(1)
–	Pseudoparidinae + Buforrestiinae + Floscopinae	–/–	11(0), 130(0), 132(0), 152(2), 199(2), 206(0), 260(1), 318(1), 351(1)
–	Buforrestiinae + Floscopinae	53/0.58	7(0), 54(1), 78(1), 91(0), 112(0), 116(0), 128(0), 129(0), 140(0), 153(2), 201(1), 204(1), 208(2), 241(1), 243(1), 244(1), 319(1), **346(0)**, 352(0)
subtribe	Buforrestiinae	72/1	43(1), **58(1)**, 106(0), 130(5), 142(0), 199(1), 243(3), 275(1), 276(1), **317(4)**, 447(0), **486(1)**
subtribe	Floscopinae	95/1	17(0), 50(0), 90(0), 117(0), 127(1), 139(3), 152(1), 180(2), 242(0), 260(0), 389(1), 399(2), **449(1)**, 580(6)
–	*Saxofloscopa* + *Stanfieldiella*	96/1	62(2), 92(2), 138(1), 146(2), 150(1), 201(0), 241(0), 244(0), 387(1), 391(2), **480(1)**, **582(0)**
subtribe	Commelininae	98/1	17(0), 41(0), 43(1), 54(1), 146(2), 219(1), 231(1), 237(0), 246(0), 249(2), 275(1), 276(1), **313(3)**, 319(2), **374(4)**, 395(0), 396(1), 398(0), 417(2), 439(1), **481(6)**, **483(3)**
–	Pollia clade + Commelina clade	98/1	21(1), 46(2), 79(1), 99(1), 116(0), 202(0), 206(0), 222(1), 236(0), 241(1), 273(0), **278(2)**, 336(1), 337(2), 394(3), 399(2), **402(2)**, 425(1), **566(1)**
–	Pollia clade	79/1	25(1), 41(1), 145(1), 148(1), **149(1)**, 150(1), 197(3), **234(5)**, 242(4), 243(4), **406(1)**
–	*Fadeniella* + *Polyspatha*	97/1	27(0), **93(1)**, 132(0), 142(0), 208(3), 244(1), 256(1), 417(0), 418(0)
–	Commelina clade	66/0.99	90(0), 130(0), 208(3), 235(1), 237(1), 344(0), **382(1)**, 396(2), 417(1), 580(3)
tribe	Tradescantieae	58/1	7(1), 11(0), **45(1)**, 46(2), 50(2), 202(2), 224(0), 232(2), **286(3)**, **292(3)**, 293(1), 295(1), 297(3), 317(1), 320(1), 391(1), 436(0), 439(1), 453(2), 454(2), 484(1), **499(1)**, 554(1), **581(3)**
subtribe	Streptoliriinae	99/1	2(1), 19(0), 43(1), 59(3), 81(2), 90(0), 114(2), **116(2)**, **139(4)**, **140(2)**, 145(0), **201(2)**, 224(2), 225(2), 234(4), 235(2), 243(4), 244(1), 284(1), 287(1), 290(1), 309(1), 319(1), 451(2), **506(3)**, 508(2)
–	*Aëtheolirion* + *Streptolirion*	94/1	8(1), 21(2), 41(0), 44(0), 46(3), **52(1)**, 60(1), 102(1), **114(3)**, 237(0), 246(0), 335(1), 336(1), 338(1), 391(0), 417(4), 418(2), 580(1)
–	Cochliostematinae + Dichorisandrinae + Cyanotinae s.lat. + Tradescantia alliance	–/1	15(3), 78(1), 92(0), 106(0), 180(3), 198(2), 199(2), 236(0), 245(0), 282(2), 298(2), 303(0), 305(0), **369(3)**, 373(1), 407(3), 446(1), 447(1), **538(1)**, 560(1), **580(9)**, 582(4)
subtribe	Cochliostematinae	100/1	9(1), 46(1), 48(1), 62(2), 79(1), 81(1), 88(1), 145(1), **152(3)**, 205(2), 206(0), 226(1), 227(1), **230(5)**, 249(2), 252(0), **263(1)**, 269(2), 270(0), 271(3), 272(0), 273(2), 282(4), 298(4), 328(3), **329(3)**, **331(3)**, 335(1), 336(1), 338(1), 349(0), **369(8)**, 387(0), 444(1)
–	*Cochliostema* + *Plowmanianthus*	95/1	14(0), 19(0), 21(1), 22(1), 44(2), 45(2), 50(1), 208(2), 219(1), 246(0), 256(2), 283(1), 299(1), 313(0), 347(0)
–	Dichorisandrinae s.str. + Cyanotinae s.lat. + Tradescantia alliance	–/0.83	7(0), 20(2), 47(2), 51(1), 60(1), 61(0), 115(0), 242(2), 317(0), 318(0), 320(0), 337(0), 357(1), 419(1), **504(2)**, **539(1)**, 592(1)
subtribe	Dichorisandrinae s.str.	58/0.99	50(1), 92(2), 117(0), 148(1), 219(1), 224(1), 225(1), **242(3)**, 279(0), 295(0), 304(1), 306(1), 308(1), 310(1), 311(2), 450(1), 484(0), **541(1)**, **542(2)**
–	Cyanotinae s.lat.+ Tradescantia alliance	84/1	14(0), 15(1), 17(0), 41(0), 59(1), 101(0), 140(0), 145(0), 202(1), 248(0), 290(1), 393(1), 395(2), 396(2), 398(0), 399(2), 451(2), **509(1)**, 554(0), 580(0), **582(3)**
subtribe	Cyanotinae s.lat.	99/1	7(1), 21(1), 79(1), 81(1), **84(3)**, 89(0), 99(1), 100(3), **127(3)**, 130(1), 131(1), 139(2), 163(1), 204(2), 223(2), 224(2), 225(2), 227(1), 231(1), 234(3), 239(1), 243(3), 293(2), 307(0), 333(1), 439(0), 481(5), 561(1), 568(1), 580(4), 597(1)
(subtribe)	*Nivoanthus* + *Coleotrype* s.str. + *Amischotolype* s.lat./Coleotrypinae	–/–	43(1), 46(1), 50(1), 59(3), 88(1), 224(4), 255(2), 257(2), 282(4), 293(4), 298(4), 431(0), 444(1)
–	*Coleotrype* s.str. + *Amischotolype* s.lat.	–/–	41(1), 197(2), 224(5), **229(7)**, 303(1), 305(1), 307(1), 309(1), 333(0), 450(1)
(subtribe)	Tradescantia alliance/Tradescantiinae s.lat.	71/1	11(1), 61(2), 78(0), **128(1)**, 146(1), 180(1), 198(1), 199(1), **221(1)**, 287(0), 303(1), 305(1), 309(1), 317(2), 318(2), 319(2), 320(2), 343(1), **369(2)**, **374(1)**, 391(0), 419(0), **452(3)**, 465(0), 467(2)
subtribe	Tinantiinae	96/1	1(1), 47(0), 91(0), 98(0), 116(0), **119(1)**, 127(2), 142(1), 145(1), 152(1), 219(1), 226(1), 249(2), 275(1), 276(1), 278(1), 290(0), 355(1), 357(0), **373(2)**, 580(8), 582(2), 583(1)
–	Thyrsantheminae + Tradescantia clade	–/–	27(0), **45(0)**, 146(2), 236(1), 237(0), 245(1), 246(0), 282(1), 287(1), 298(1), 369(4), **385(0)**, 402(0), 417(2), 437(0), 440(0), 453(0), 454(0)
subtribe	Thyrsantheminae	98/1	2(1), **13(1)**, 14(1), **17(3)**, 22(1), **48(5)**, 50(0), 53(1), 59(2), 78(1), 101(1), 112(0), 129(0), 139(2), 223(2), **278(4)**, 391(2), 393(0), 564(1), 597(1)
–	*Gibasoides* + *Matudanthus* + *Weldenia*	87/1	5(2), **18(1)**, 44(2), 62(2), 102(1), 140(1), 180(3), 237(2), 246(2), 257(1), 467(0)
–	*Matudanthus* + *Weldenia*	89/1	89(0), 101(0), 280(1), 282(0), 283(1), 285(1), 287(0), 288(1), 291(1), 293(0), 294(1), 296(1), 298(0), 299(1), **304(9)**, **306(9)**, 308(9), 310(9), 311(1), 393(1), 417(4)
–	Tradescantia clade	99/1	40(0), 45(2), 54(1), **84(1)**, 90(0), 98(1), **100(1)**, **107(1)**, **108(1)**, **110(1)**, 134(0), 154(2), 164(0), 204(2), 304(1), 306(1), 308(1), 310(1), 317(0), 318(0), 319(0), 320(0), **330(1)**, **331(1)**, 374(0), 387(1), 401(0), 439(0), 446(0), 481(1), 531(0), 560(0), 592(0)
subtribe	Callisiinae	76/1	9(1), 48(1), 102(1), 232(0), 236(0), 245(0), 271(0), 273(2), 344(0), **432(2)**, **437(2)**, **447(2)**, **481(3)**, 500(1), **565(4)**
–	*Cuthbertia* + *Huntiella*	86/1	14(1), 50(0), 51(0), 54(0), 61(0), **122(2)**, 180(2), 221(0), 237(2), 328(4), 329(4), 330(4), 331(4), 407(1), 568(1)
–	*Aploleia* + *Hadrodemas* + *Callisia* s.tr. + *Tripogandra* s.lat.	92/1	19(0), 56(0), 78(1), 248(1), 272(0), 274(1), 275(1), 276(1), 277(1), 279(0), 282(2), 283(2), 284(0), 287(2), 288(2), **289(1)**, 290(0), 293(2), 294(2), 295(0), 298(2), 299(2), **300(1)**, 369(1), 387(0), 398(1), 440(1), 484(0)
–	*Hadrodemas* + *Callisia* s.tr. + *Tripogandra* s.lat.	71/1	76(1), 79(1), 134(2), 145(1), 236(1), 245(1), 278(1), 324(1), 325(1), 326(1), 327(1), 357(0), **482(0)**, 497(1), **512(1)**
–	*Hadrodemas* + *Callisia* s.tr.	51/–	5(2), 22(1), 46(1), 50(0), 60(0), 81(1), 89(0), 127(2), 163(1), 202(2), 224(2), 225(2), 328(1), 329(1), 398(0)
subtribe	Tradescantiinae s.str.	91/1	62(2), 99(1), 127(2), **151(2)**, 280(1), 283(1), 285(1), 288(1), 291(1), 294(1), 296(1), 299(1), **304(3)**, **306(4)**, **308(4)**, **310(4)**, 324(1), 325(1), 326(1), 327(1), 328(1), 329(1), 444(1), 453(1), 454(1), **480(3)**, 504(0), **510(0)**, **511(2)**
–	*Elasis* s.str. + *Ivoniella* + *Gibasis*	60/–	20(1), 21(1), 61(0), 79(1), 98(2), 100(2), 101(1), 107(0), 110(0), 234(0), 243(0), 391(2), 417(0), 564(1)
–	*Ivoniella* + *Gibasis*	62/1	27(1), **109(0)**, **489(1)**, **524(1)**

Character numbers are presented followed by the states between brackets, i.e., “character number (state number)”. BS—bootstrap support; PP—posterior probability.

**Table 5:** Summary of recovered exclusive (**bold**) and homoplastic synapomorphic characters for all clades at genus rank for Commelinaceae.

Family	Genus	Statistical Support (BS/PP)	Synapomorphies
Commelinaceae	*Cartonema*	94/1	17(2), 27(0), 47(1), 136(0), 146(1), 197(5), 279(0), 333(1), 395(2), 396(2), 418(2), 432(3), **433(1)**, 518(0)
Commelinaceae	*Triceratella*	100/1	1(1), 3(0), 19(0), 21(1), 41(0), 79(1), 81(2), 116(0), 234(3), 243(3), 261(4), 284(1), 290(1), 295(1), **436(2)**, 437(4), 440(3), 448(0), 497(1), 554(1)
Commelinaceae	*Palisota*	100/1	**40(3)**, 47(1), 117(0), 129(0), 138(1), 140(3), 152(2), 153(1), 167(0), 168(1), 200(1), 201(1), 202(0), 208(0), 224(4), 225(4), 234(4), 235(2), 243(4), 244(1), 282(2), 283(1), 284(1), 286(4), 287(2), 288(1), 307(0), 347(0), 352(0), **353(2)**, 399(2), **403(4)**, 416(0), 417(1), 422(1), 425(0), 432(0), 437(1), 440(0), 448(0), 450(1), **473(1)**, **483(5), 485(1)**, 591(1)
Commelinaceae	*Murdannia* s.lat.	100/1	2(1), 5(0), 27(0), 44(2), 47(2), 48(1), 50(0), 59(1), 60(0), 99(1), 117(0), 131(1), 180(3), 205(1), 226(0), 243(0), 248(0), 269(1), 270(1), 271(2), 272(2), 273(0), 286(0), 287(1), 292(0), 293(1), 317(2), 349(0), 353(0), **481(4)**, 498(1), **525(1)**, 580(5), 592(1)
Commelinaceae	*Pseudoparis*	100/1	14(1), 59(0), 61(1), 82(2), 92(2), 154(0), 206(2), 234(2), **236(4)**, 237(0), **245(4)**, 246(0), 251(0), 269(2), 278(0), 304(1), 310(1), 328(4), 331(4), 387(2), 425(0), 554(1)
Commelinaceae	*Buforrestia*	100/1	7(1), 54(0), 79(1), 81(1), 88(1), 112(1), 129(1), 131(1), 153(0), 200(2), 203(1), 206(2), 208(3), 237(0), 242(2), 246(0), 261(2), **268(2)**, 274(1), 277(1)
Commelinaceae	*Tricarpelema* s.str.	98/1	78(0), 140(1), 180(3), 204(0), 224(0), 225(0), 234(1), 308(9), 310(9), **317(5)**, 336(1), 337(1), 387(2), **487(1)**, 496(1)
Commelinaceae	*Saxofloscopa*	100/1	5(2), 40(0), 47(2), 48(1), 140(1), 498(1)
Commelinaceae	*Stanfieldiella*	79/0.98	41(0), 50(1), **154(5)**, 243(0)
Commelinaceae	*Floscopa*	100/1	5(0), 47(1), 131(0), **304(4)**, **310(4)**, 328(2), 331(2), 357(0), 394(1), 395(0), 417(3), 425(1), 432(1), 440(3), 446(0), 481(1), 560(0), 583(1)
Commelinaceae	*Dictyospermum*	99/1	11(0), 91(0), 112(0), 128(0), 140(0), 144(1), 245(0), 253(0), 256(1), 260(1), **274(2)**, **275(3)**, **276(2)**, 308(4), **313(4)**, 395(1), **401(2)**, 427(0), 440(1)
Commelinaceae	*Pollia* s.str.	100/1	43(0), 47(1), **53(2)**, 100(2), 102(1), 206(1), 241(0), **243(5)**, 249(1), 394(2), 395(4), 396(4), 422(1), **423(1)**, 425(0), 427(0), 453(0), 454(0), 580(0), 592(1), 597(1)
Commelinaceae	*Fadeniella*	100/1	1(1), 21(0), 78(1), 79(0), 152(2), 153(4), 367(1)
Commelinaceae	*Polyspatha*	99/1	10(1), 22(1), 40(0), 41(0), 99(0), 114(2), 140(0), 204(2), 232(2), **382(2)**, 440(3)
Commelinaceae	*Commelina* s.lat.	100/1	7(1), 10(1), 40(0), 54(0), **57(1)**, 81(2), 84(2), 92(0), 98(1), 100(3), 102(1), 106(0), 111(0), 127(0), 134(0), 154(2), 225(2), 246(1), 308(5), **379(1)**, **380(2)**, 395(1), 396(3), 424(0), 444(1), 580(6), **586(1)**, 592(1)
Commelinaceae	*Aneilema* s.lat.	75/1	17(1), 20(2), 21(0), 60(0), 79(0), 99(0), 130(1), 202(1), **218(1)**, 222(0), 232(0), 367(1), 569(1)
Commelinaceae	*Aëtheolirion*	100/1	145(1), **154(6)**, 180(0), 198(2), 224(5), 249(2), 252(0), 270(0), 273(0), 283(2), 284(0), 299(2), 303(0), 305(0), 309(0), 425(2), 431(0), 434(1), 440(0), 506(1), 507(1)
Commelinaceae	*Streptolirion*	99/1	1(1), 3(0), 5(2), 14(0), 78(1), 79(1), 88(1), 395(2), 396(2), 446(1), 560(1)
Commelinaceae	*Spatholirion*	83/1	**111(2)**, 127(0), 148(1), 168(1), 248(0), 337(0), 403(1), 419(1), 450(1)
Commelinaceae	*Geogenanthus*	99/1	54(1), 59(0), 82(2), 100(3), **146(3)**, **151(3)**, 199(1), 222(1), 507(3), 508(2)
Commelinaceae	*Cochliostema*	100/1	**5(3)**, 9(0), 47(1), 88(0), 91(0), 92(2), 99(1), 100(2), 114(2), 127(0), 138(1), 139(2), 140(0), 142(1), 144(1), **229(6)**, 234(3), 243(3), 262(1), 273(0), 284(1), 391(0), 440(0), **444(2)**, 446(0), 450(1), 560(0)
Commelinaceae	*Plowmanianthus*	95/1	48(4), 61(1), 98(0), 141(1), 197(2), 237(0), 251(0), 387(2), **567(3)**
Commelinaceae	*Dichorisandra*	97/1	20(1), 47(0), 127(2), **130(3)**, 202(0), 238(1), 242(4), 247(1), 260(1), 307(0), **311(3)**, 321(1), 333(1), 335(1), 336(1), 337(1), 338(1), 391(0), 424(1), **567(1)**, 597(1)
Commelinaceae	*Siderasis*	96/1	19(0), 48(1), 303(1), 305(1), 309(1), 317(1), 318(1), 319(1), 320(1), 328(4), 329(4), 330(4), 331(4), **369(9)**, 418(0), 481(1), 568(1)
Commelinaceae	*Cyanotis* s.lat.	100/1	1(1), 10(1), 27(0), 40(0), 60(0), 76(1), 164(0), 232(0), **261(3)**, 287(2), **340(0)**, **400(1)**, 440(4), **446(2)**, 453(0), 454(0), **483(2)**, 538(0), **560(2)**, **565(6)**, 591(0)
Commelinaceae	*Nivoanthus*	100/1	7(0), 14(1), 47(0), 79(0), 142(1), 145(1), 170(1), 180(0), 208(2), 219(1), 226(1), **227(2)**, 243(2), 248(1), 249(2), **256(4)**, 280(1), 285(1), 291(1), 296(1), 311(2), 313(0), 321(1), 322(1), **324(2)**, **328(6)**, **329(6)**, **330(6)**, **331(6)**, 454(3), 561(2), 592(0), 597(0), 599(1)
Commelinaceae	*Coleotrype* s.str.	83/1	46(0), 53(1), 59(2), 136(1), 208(0), 223(1), 249(1), 401(0), **425(7)**, 440(1), **451(3)**, 481(1), **567(2)**, 569(0)
Commelinaceae	*Amischotolype* s.lat.	94/1	21(0), **95(1)**, 164(0), 180(2), 200(1), 202(2), 204(0), 225(0), 227(0), 231(0), 239(0), 255(0), 282(1), 287(1), 293(1), 298(1), 424(1), 431(1), 444(0), 452(1), **511(1)**
Commelinaceae	*Sauvallia*	100/1	21(1), 44(3), 48(1), 84(2), 89(0), 90(0), 115(1), 135(0), 202(0), 234(3), 243(3), 394(1), 425(1)
Commelinaceae	*Tinantia*	95/1	43(1), 51(0), 101(1), 106(1), **119(1)** *, 140(1), 180(3), 206(0), 208(2), 248(1), 269(0), 270(2), 271(3), 272(2), 273(2), 335(1), 336(1), 337(1), 338(1), 373(2) *, 395(4), 396(4)
Commelinaceae	*Thyrsanthemum* s.str.	100/1	**15(5)**, 76(1), 92(2), 106(1), 139(3), 146(1), **149(2)**, **220(2)**, **223(3)**, 224(4), 225(4), 249(1), 328(4), 329(4), 300(4), 331(4), **374(2)**, 387(0), 418(2), 437(3), 450(1), 453(2), 454(2)
Commelinaceae	*Gibasoides*	100/1	–
Commelinaceae	*Matudanthus*	100/1	19(0), 40(0), 44(1), 48(0), 135(1), **151(4)**
Commelinaceae	*Weldenia*	100/1	41(1), 46(0), 60(0), 61(0), 62(1), 146(0), 163(1), **168(2)**, 217(1), 219(1), 224(4), 225(4), 227(1), 231(1), 237(0), 246(0), 256(3), **259(2)**, 279(0), 284(0), 290(0), 295(0), 357(0), 369(0), 391(0), 395(4), 396(4), 484(0)
Commelinaceae	*Cuthbertia*	98/1	2(1), **12(1)**, 20(1), 46(0), 59(2), **230(1)**, 246(2), 507(0), 580(1)
Commelinaceae	*Huntiella*	72/1	60(0), 89(0), **93(2)**, 127(2), **130(4)**, 134(2), 223(2), 599(1)
Commelinaceae	*Aploleia*	98/1	2(1), 10(1), 21(1), 202(0), 222(1), 303(0), 305(0), 307(0), 309(0), 311(2), 330(0), 331(0), **397(1)**, 440(4), **562(0)**, 565(0), 569(0), 580(2)
Commelinaceae	*Hadrodemas*	100/1	25(1), 40(1), 41(1), 44(2), 51(0), 56(1), 80(1), 98(2), **110(2)**, 148(1), 180(2), 236(0), 237(2), 245(0), 246(2), 249(2), 357(1), 369(4), 401(1), 402(1)
Commelinaceae	*Callisia* s.str.	99/1	9(0), 116(0), **130(2)**, 139(2), 140(1), 145(0), 197(0), **202(4)**, 223(2), 224(4), 225(4), 235(2), 244(1), 248(0), 274(0), 275(0), 276(0), 277(0), 278(0), 304(2), 306(2), 308(2), 310(2), 328(2), 329(2), 330(2), 331(2), 391(1), 395(1), 396(1), 407(4), 565(0), 569(0), 580(1)
Commelinaceae	*Tripogandra* s.lat.	99/1	7(1), 19(1), 20(0), 47(0), 48(0), 61(0), 99(1), **114(0)**, **120(2)**, **122(3)**, 144(1), 146(1), 234(1), 243(1), 249(1), 260(1), 295(1), 304(0), 306(0), 313(0), 317(1), 318(1), 319(1), 320(1), 335(1), 336(1), 337(1), 338(1), 369(5), 484(1), 500(0), 580(3), 599(1)
Commelinaceae	*Tradescantia*	60/1	40(1), 44(2), 114(2), 116(0), 130(0), **133(1)**, 154(0), **360(1)**, **481(2)**, 509(0), **565(1)**, 580(1), 582(2), **595(1)**, **598(1)**
Commelinaceae	*Elasis* s.str.	100/1	8(1), 10(1), 19(0), 76(1), 78(1), 81(1), 100(3), 139(2), 154(1), 180(2), 224(1), 225(1), 237(2), 246(2), 282(0), 287(0), 293(0), 298(0), 303(0), 305(0), 307(0), 309(0), **316(2)**, **317(3)**, **318(3)**, **319(3)**, **320(3)**, 324(0), 325(0), 326(0), 327(0), 328(0), 329(0), 330(0), 331(0), 393(0)
Commelinaceae	*Ivoniella*	100/1	47(0), 62(1), **77(3)**, **100(4)**, 180(3), 202(0), 237(1), 246(1), 580(2)
Commelinaceae	*Gibasis*	69/1	9(1), 21(0), 41(1), 61(2), 98(1), 102(1), **103(1)**, **154(3)**, **282(3)**, **287(3)**, **293(3)**, **298(3)**, **304(8)**, **306(8)**, **308(8)**, **310(8)**, 369(1), 454(0), 545(1), **565(7)**

Character numbers are presented followed by the states between brackets, i.e., “character number (state number)”. BS—bootstrap support; PP—posterior probability. * recovered as synapomorphic for the Tinantiinae due to missing data, but actually synapomorphic only to *Tinantia*.

**Table 6:** Proposed infra-ordinal classification for Commelinales, including all recognised taxa down to genus.

Suborder	Superfamily	Family	Subfamily	Tribe	Subtribe	Genus
**Commelinineae Engl.**	—	**Commelinaceae Mirb.**	**Cartonematoideae Faden ex G.C.Tucker**	Cartonemateae Faden & D.R.Hunt	—	*Cartonema* R.Br.
				Triceratelleae Faden & D.R.Hunt	—	*Triceratella* Brenan
			**Commelinoideae Eaton**	Commelineae Dumort.	Commelininae C.K.Lee et al.	*Aneilema* R.Br., *Commelina* Plum. ex L., *Dictyospermum* Wight, *Fadeniella* M.Pell., *Pollia* Thunb., and *Polyspatha* Benth.
					Buforrestiinae M.Pell.	*Buforrestia* C.B.Clarke and *Tricarpelema* J.K.Morton
					Floscopinae M.Pell.	*Floscopa* Lour., *Saxofloscopa* M.Pell., and *Stanfieldiella* Brenan
					Murdanniinae M.Pell. & Faden ex C.K.Lee et al.	*Murdannia* Royle
					Pseudoparidinae M.Pell.	*Pseudoparis* H.Perrier
				Palisoteae M.Pell. & Faden ex Zuntini & Frankel	—	*Palisota* Rchb. ex Endl.
				Tradescantieae Meisn.	Streptoliriinae Faden & D.R.Hunt	*Aëtheolirion* Forman, *Spatholirion* Ridl., and *Streptolirion* Edgew.
					Cochliostematinae M.Pell. & Faden ex C.K.Lee et al.	*Cochliostema* Lem., *Geogenanthus* Ule, and *Plowmanianthus* Faden & C.R.Hardy
					Dichorisandrinae Faden & D.R.Hunt	*Dichorisandra* J.C.Mikan and *Siderasis* Raf. emend. M.Pell. & Faden
					Cyanotinae Faden & D.R.Hunt	*Amischotolype* Hassk., *Coleotrype* C.B.Clarke, *Cyanotis* D.Don, and *Nivoanthus* M.Pell.
					Tinantiinae M.Pell. ex Z.H.Feng	*Sauvallia* C.Wright ex Hassk. and *Tinantia* Scheidw.
					Thyrsantheminae D.R.Hunt ex Faden & D.R.Hunt	*Gibasoides* D.R.Hunt, *Matudanthus* D.R.Hunt, *Thyrsanthemum* Pichon, and *Weldenia* Schult. f.
					Tradescantiinae Rohw.	*Elasis* D.R.Hunt, *Gibasis* Raf., *Ivoniella* M.Pell., and *Tradescantia* Ruppius ex L. emend. M.Pell.
					Callisiinae M.Pell.	*Aploleia* Raf., *Callisia* Loefl., *Cuthbertia* Small, *Hadrodemas* H.E.Moore, *Huntiella* M.Pell., and *Tripogandra* Raf. emend. M.Pell. & Handlos
	—	**Hanguanaceae Airy Shaw**	—	—	—	*Hanguana* Blume
**Pontederiineae Engl.**	—	**Philydraceae Link**	—	—	—	*Helmholtzia* F.Muell., *Orthothylax* (Hook. f.) Skottsb., *Philydrella* Caruel, and *Philydrum* Banks ex Gaertn.
	**Pontederiorae M.Pell.**	**Haemodoraceae R.Br.**	**Conostylidoideae T.D.Macfarl. & Hopper**	Conostylideae Benth.	—	*Anigozanthos* Labill., *Conostylis* R.Br., and *Phlebocarya* R.Br.
				Tribonantheae T.D.Macfarl. & Hopper	—	*Tribonanthes* Endl.
			**Haemodoroideae Arn.**	Haemodoreae Dumort.	—	*Dilatris* P.J.Bergius, *Haemodorum* Sm., *Lachnanthes* Elliott, and *Paradilatris* (Hopper ex J.C.Manning) Hopper
				Wachendorfieae Dumort.	—	*Schiekia* Meisn. and *Wachendorfia* Burm. ex L.
				Xiphidieae Dumort.	—	*Cubanicula* Hopper et al., *Pyrrorhiza* Maguire & Wurdack, and *Xiphidium* Aubl.
		**Pontederiaceae Kunth**	—	—	—	*Heteranthera* Ruiz & Pav. and *Pontederia* L.

## Data Availability

The original contributions presented in this study are included in the article/Supplementary Materials. Further inquiries can be directed to the corresponding author.

## Supplementary Materials

The following supporting information can be downloaded at https://www.mdpi.com/article/10.3390/plants15111738/s1, Figure S1. Full WinClada tree for Commelinales; Supplementary Table S1. List of voucher specimens for the morphological matrix; Supplementary Table S2. List of morphological characters and their respective states used in the phylogenetic analysis; Supplementary Table S3. Morphological matrix for Commelinales (separate .xlsv file); Supplementary Table S4. Historical ordinal placement of the families previously placed in Commelinales and currently placed elsewhere. Supplementary Table S5. Historical ordinal placement of families currently accepted in Commelinales; Supplementary List S1. Linear arrangement for the proposed infra-ordinal classification for Commelinales.

## Abbreviations

The following abbreviations are used in this manuscript:

ACCTRAN	Accelerated Transformation Optimisation
APES	School of Animal, Plant & Environmental Sciences
BA	Bayesian Analysis
BS	Bootstrap Support
CAPES	Coordenação de Aperfeiçoamento de Pessoal de Nível Superior—Brasil
CI	Consistency Index
HI	Homoplasy Index
MCMC	Markov Chain Monte Carlo
MP	Maximum Parsimony
OTU	Operational Taxonomic Unit
PhD	Philosophy Doctor
PP	Posterior Probability
RC	Rescaled Consistency Index
RI	Retention Index
TBR	Tree Bisection–Reconnection
USP	Universidade de São Paulo
WITS	University of the Witwatersrand
