# Supplementary material for: Evolution, Systematics and Classification of Commelinales (Commelinids, Monocots) Based on a Giant Morphological Taxon-Character Matrix
Source: Plants (Basel). 2026 Jun 3;15(11):1738. doi: 10.3390/plants15111738 (PMC13259461; doi:10.3390/plants15111738)
Supplement: Supplementary file 1 [file plants-15-01738-s001.zip › Supplementary files.pdf]

plants

MDPI

Article

Evolution, systematics and classification of Commelinales (Commelinids, Monocots) based on a giant morphological taxon-character matrix

Marco O.O. Pellegrini [1,2]

[1] University of the Witwatersrand, School of Animal, Plant & Environmental Sciences, C.E. Moss Herbarium, Johannesburg, South Africa; e-mail: Marco.Pellegrini@wits.ac.za
[2] Royal Botanic Gardens, Kew, Richmond, Surrey, UK; e-mail: M.Pellegrini@kew.org

Abstract

Despite being strongly recovered as monophyletic by molecular studies, Commelinales completely lacks any morphological support or circumscription. It is also the Monocot order that suffered the most striking changes across different classification systems, with its type-family, Commelinaceae, being the only consistent member since its proposition. The order currently consists of Commelinaceae, Haemodoraceae, Hanguanaceae, Philydraceae and Pontederiaceae, presenting a Pantropical distribution and great ecological and morphological diversity. Based on extensive field, cultivation, ecological, herbarium, botanical illustration and literature research, I present the first morphological phylogeny for Commelinales, based on an extensive 600-character matrix, sampling almost a third of the species in the order. All five families are recovered as monophyletic, with 49 of the 59 currently recognised genera also recovered as monophyletic. The MP and BA topologies are greatly congruent with the available molecular hypotheses for Commelinales, highlighting the importance of morphology in understanding the systematics of plant groups. Almost all genera are morphologically supported by at least one exclusive synapomorphy. Thus, based on a combination of morphological and molecular data, *Aneilema*, *Callisia*, *Coleotrype*, *Elasis*, *Thyrsanthemum*, *Tricarpelema* and *Tripogandra* (Commelinaceae) are recircumscribed to represent monophyletic genera. Five new genera of Commelinaceae are described, in addition to the reestablishment of *Aploleia*, *Cuthbertia*, *Gibasoides* and *Hadrodemas* (Commelinaceae), and *Orthotylax* (Philydraceae). The circumscription of *Anigozanthos* (Haemodoraceae) is broadened to include *Macropidia*, *Conostylis* (Haemodoraceae) is broadened to include *Blancoa*, and *Wachendorfia* (Haemodoraceae) is broadened to include *Barberetta*. Finally, I propose an updated classification for Commelinales, recognising two suborders, one superfamily, five families, four subfamilies, 10 tribes, 13 subtribes (four of them newly described here), and 64 genera (five of them newly described here).

Keywords: Commelinaceae; Haemodoraceae; Hanguanaceae; Philydraceae; Pontederiaceae; taxonomy; nomenclature

Supplementary files

Supplementary Figures

Supplementary Figure S1

Figure S1. Full WinClada tree for Commelinales (separate .tif file).

Supplement- Full WinClada tree

Supplementary Tables

Supplementary Table S1

Supplementary Table S1. List of voucher specimens for the morphological matrix.

Family	Taxon	Voucher
Costaceae	*Costus pulverulentus* C.Presl	Donnell 4973 (US)
Zingiberaceae	*Zingiber officinale* Roscoe	Canfield 290 (US)
Hanguanaceae	*Hanguana bakoënsis* Siti Nurfazilah et al.	Malcom S.100599 (SAR)
Hanguanaceae	*Hanguana bogneri* Tillich & E.Sill	Bogner 94/2211 (M)
Hanguanaceae	*Hanguana exultans* Siti Nurfazilah et al.	Siti Nurfazilah HA-55 (KEP)
Hanguanaceae	*Hanguana loi* Mohd Fahmi et al.	Chai S34089 (K)
Hanguanaceae	*Hanguana major* Airy Shaw	Chew RSNB4233 (K)
Hanguanaceae	*Hanguana malayana* (Jack) Merr.	Curtis s.n. (SING 0203818)
Hanguanaceae	*Hanguana nitens* Siti Nurfazilah et al.	Siti Nurfazilah HA-48 (KEP)
Hanguanaceae	*Hanguana pantiensis* Siti Nurfazilah et al.	Siti Nurfazilah HA-56 (KEP)
Hanguanaceae	*Hanguana podzolica* Siti Nurfazilah et al.	Siti Nurfazilah HA-50 (KEP)
Hanguanaceae	*Hanguana stenopoda* Siti Nurfazilah et al.	Siti Nurfazilah HA-60 (KEP)
Commelinaceae	*Aëtheolirion stenolobium* Forman	Thitimetharoch 579 (US)
Commelinaceae	*Amischotolype glabrata* Hassk.	Cameron s.n. (US 00520209)
Commelinaceae	*Amischotolype gracilis* (Ridl.) I.M.Turner	Bartlett 7312 (US)
Commelinaceae	*Amischotolype hispida* (Less & A.Rich.) D.Y.Hong	Robinson 1831 (US)
Commelinaceae	*Amischotolype hookeri* (Hassk.) H.Hara	Kress 98-6247 (US)
Commelinaceae	*Amischotolype microphylla* (Y.Wan) C.K.Lee. et al.	Thitimetharoch 458 (US)
Commelinaceae	*Amischotolype monosperma* (C.B.Clarke) I.M.Turner	Bogner 1811 (US)
Commelinaceae	*Amischotolype neoscandens* Idrees	Thitimetharoch 410 (US)
Commelinaceae	*Amischotolype ramosa* (D.Y.Hong) C.K.Lee. et al.	Henry 12204a (US)
Commelinaceae	*Amischotolype rostrata* (Hassk.) Duist.	Hallier 533/71 (L)
Commelinaceae	*Aneilema acuminatum* R.Br.	Faden 1/94 (US)
Commelinaceae	*Aneilema aequinoctiale* (P.Beauv.) G.Don	Faden 290 (US)
Commelinaceae	*Aneilema beniniense* (P.Beauv.) Kunth	Faden 87/2 (US)
Commelinaceae	*Aneilema biflorum* R.Br.	Pellegrini 218 (RB)
Commelinaceae	*Aneilema brasiliense* C.B.Clarke	Amaral 7/99 (US)
Commelinaceae	*Aneilema calceolus* Brenan	Faden 77/565 (US)
Commelinaceae	*Aneilema clarkei* Rendle	Faden 77/629 (US)
Commelinaceae	*Aneilema gillettii* Brenan	Friis 1044 (US)
Commelinaceae	*Aneilema grandibracteolatum* Faden	Glover 408 (K)
Commelinaceae	*Aneilema hockii* De Wild.	Faden 96/9 (US)
Commelinaceae	*Aneilema johnstonii* K.Schum.	Faden 97/8 (US)
Commelinaceae	*Aneilema leiocaule* K.Schum.	Bidgood 440 (US)
Commelinaceae	*Aneilema neocaledonicum* Schltr.	MacKee 2170 (US)
Commelinaceae	*Aneilema pedunculosum* C.B.Clarke	Bidgood 4196 (US)
Commelinaceae	*Aneilema petersii* (Hassk.) C.B.Clarke	Luke 2495 (US)
Commelinaceae	*Aneilema rendlei* C.B.Clarke	Faden 27/85 (US)

Commelinaceae	*Aneilema scaberrimum* Blume	Reinwardt s.n. (L 0820741)
Commelinaceae	*Aneilema somaliense* C.B.Clarke	Faden 74/939 (US)
Commelinaceae	*Aneilema taylorii* C.B.Clarke	Faden 74/371 (US)
Commelinaceae	*Aneilema umbrosum* (Vahl) Kunth	Faden 74/2 (US)
Commelinaceae	*Aneilema vitiense* Seem.	Seemann 643 (K)
Commelinaceae	*Aneilema zebrinum* Chiov.	Faden 77/311 (US)
Commelinaceae	*Buforrestia candolleana* C.B.Clarke	Pires 52215 (US)
Commelinaceae	*Buforrestia mannii* C.B.Clarke	Faden 87/5 (US)
Commelinaceae	*Buforrestia obovata* Brenan	Straub 145 (US)
Commelinaceae	*Callisia ciliata* Kunth	Prance 16762 (US)
Commelinaceae	*Callisia cordifolia* (Sw.) E.S.Anderson & Woodson	Donnell 414 (US)
Commelinaceae	*Callisia elegans* Alexander ex H.E.Moore	Molina 25165 (US)
Commelinaceae	*Callisia filiformis* (M.Martens & Galeotti) D.R.Hunt	Sobral-Leite 814 (RB)
Commelinaceae	*Callisia fragrans* (Lindl.) Woodson	Purpus 10668 (US)
Commelinaceae	*Callisia gentlei* Matuda	Adams 230 (K)
Commelinaceae	*Callisia gracilis* (Kunth) D.R.Hunt	Dorr 8571 (US)
Commelinaceae	*Callisia graminea* (Small) G.C.Tucker	Nash 879 (US)
Commelinaceae	*Callisia insignis* C.B.Clarke	Moore 1516 (US)
Commelinaceae	*Callisia hintoniorum* B.L.Turner	Hinton 25725 (US)
Commelinaceae	*Callisia laui* (D.R.Hunt) D.R.Hunt	Lau s.n. (K 000434009)
Commelinaceae	*Callisia graminea* fo. *leucantha* (Lakela) G.C.Tucker	Lakela 32048 (USF)
Commelinaceae	*Callisia macdougallii* Miranda	McDougal s.n. (US no. 1981074)
Commelinaceae	*Callisia micranta* (Torr.) D.R.Hunt	Traverse 1016 (US)
Commelinaceae	*Callisia monandra* (Sw.) Schult. & Schult. f.	Pellegrini 430 (RB)
Commelinaceae	*Callisia multiflora* (M.Martens & Galeotti) Standl.	Matuda 1992 (US)
Commelinaceae	*Callisia navicularis* (Orteges) D.R.Hunt	Hinton 18492 (US)
Commelinaceae	*Callisia ornata* (Small) G.C.Tucker	Small 9054 (US)
Commelinaceae	*Callisia repens* (Jacq.) L.	Pellegrini 284 (RB)
Commelinaceae	*Callisia rosea* (Vent.) D.R.Hunt	Harper 1314 (US)
Commelinaceae	*Callisia soconuscensis* Matuda	Hinton 11587 (US)
Commelinaceae	*Callisia tehuantepecana* Matuda	Tores 1021 (US)
Commelinaceae	*Callisia warszewicziana* (Kunth & C.D.Bouché) D.R.Hunt	Knunth BH 60-511 (US)
Commelinaceae	*Cartonema baileyi* F.M.Bailey	Bailey s.n. (BRI-AQ0512720)
Commelinaceae	*Cartonema brachyantherum* Benth.	Fitzalan s.n. (K 000854172)
Commelinaceae	*Cartonema parviflorum* Hassk.	s.leg. s.n. (L 0041670)
Commelinaceae	*Cartonema phylidroides* F.Muell.	Preiss 2228 (P)
Commelinaceae	*Cartonema spicatum* R.Br.	Brown s.n. (K 000854169)
Commelinaceae	*Cartonema tenue* Caruel	Dixon 1085 (US)
Commelinaceae	*Cartonema trigonospermum* C.B.Clarke	Fryxell 4122 (US)
Commelinaceae	*Cochliostema odoratissimum* Lem.	Knapp 1834 (US)
Commelinaceae	*Cochliostema velutinum* Read	Cuatrecasas 21438 (US)
Commelinaceae	*Coleotrype baronii* Baker	Nusbaumer 926 (US)
Commelinaceae	*Coleotrype brueckneriana* Mildbr.	Schlieben 1910 (BR)
Commelinaceae	*Coleotrype goudotii* C.B.Clarke	Goudot s.n. (G00018437)
Commelinaceae	*Coleotrype laurentii* K.Schum.	Bidault 4223 (P)
Commelinaceae	*Coleotrype lutea* H.Perrier	Perrier 7278 (P)
Commelinaceae	*Coleotrype madagascarica* C.B.Clarke	Boivin 2010 (P)
Commelinaceae	*Coleotrype natalensis* C.B.Clarke	Goldblatt 6587 (US)
Commelinaceae	*Coleotrype synanthera* H.Perrier	Perrier 19019 (P)
Commelinaceae	*Coleotrype udzungwaensis* Faden & Layton	Luke 7913 (K)
Commelinaceae	*Coleotrype vermigera* H.Perrier	Perrier 13039 (P)
Commelinaceae	*Commelina africana* L.	Faden 77/287 (US)

Commelinaceae	*Commelina benghalensis* L.	Pellegrini 233 (RB)
Commelinaceae	*Commelina calandrinioides* (F.Muell.) Zuntini & Frankel	Gulliver s.n. (K 000854161)
Commelinaceae	*Commelina communis* L.	Faden 76/29 (US)
Commelinaceae	*Commelina congesta* C.B.Clarke	Porembski 1154 (US)
Commelinaceae	*Commelina erecta* L.	Pellegrini 471 (RB)
Commelinaceae	*Commelina imberbis* Ehrenb. ex Hassk.	Faden 2003/024 (US)
Commelinaceae	*Commelina paludosa* Blume	Faden 76/206 (US)
Commelinaceae	*Commelina purpurea* C.B.Clarke	Faden 94/1 (US)
Commelinaceae	*Commelina reptans* Brenan	Faden 94/2 (US)
Commelinaceae	*Cyanotis axillaris* (L.) D.Don	Faden 76/196 (US)
Commelinaceae	*Cyanotis ciliata* (Blume) Bakh. f.	Merrill 8154 (US)
Commelinaceae	*Cyanotis beddomei* (Hook. f.) Erhardt et al.	Cultivated (US 01276642)
Commelinaceae	*Cyanotis speciosa* (L. f.) Hassk.	Faden 97/3 (US)
Commelinaceae	*Cyanotis vaga* (Lour.) Schult. f.	Gajurel 301 (US)
Commelinaceae	*Cyanotis villosa* Schult.	Faden 76/267 (US)
Commelinaceae	*Dichorisandra acaulis* Cogn.	Pellegrini 328 (RB)
Commelinaceae	*Dichorisandra amabilis* J.R.Grant	Funk 10992 (US)
Commelinaceae	*Dichorisandra hexandra* (Aubl.) C.B.Clarke	Pellegrini 484 (RB)
Commelinaceae	*Dichorisandra glabrescens* (Seub.) Aona & M.C.E.Amaral	Forzza 5514 (RB)
Commelinaceae	*Dichorisandra incurva* Mart.	Wängler 1598 (RB)
Commelinaceae	*Dichorisandra leucophatalmos* Hook.	Pellegrini 458 (RB)
Commelinaceae	*Dichorisandra marantoides* Aona & Faden	Pellegrini 492 (RB)
Commelinaceae	*Dichorisandra nana* Aona & M.C.E.Amaral	Jardim 4220 (RB)
Commelinaceae	*Dichorisandra odorata* Aona & M.C.E.Amaral	Santos-Lima 14183 (RB)
Commelinaceae	*Dichorisandra paranaënsis* D.Maia et al.	Pellegrini 469 (RB)
Commelinaceae	*Dichorisandra penduliflora* Kunth	Pellegrini 375 (RB)
Commelinaceae	*Dichorisandra procera* Mart.	Pellegrini 464 (RB)
Commelinaceae	*Dichorisandra picta* Lodd.	Costa 284 (RB)
Commelinaceae	*Dichorisandra tejucensis* Mart.	Forzza 2722 (RB)
Commelinaceae	*Dichorisandra thyrsiflora* J.C.Mikan	Pellegrini 219 (RB)
Commelinaceae	*Dichorisandra radicalis* Nees & Mart	Pellegrini 459 (RB)
Commelinaceae	*Dictyospermum conspicuum* (Blume) Hassk.	Thitimetharoch 568 (US)
Commelinaceae	*Dictyospermum humile* (Warb.) J.K.Morton	Merrill 10459 (US)
Commelinaceae	*Dictyospermum montanum* Wight	Faden 76/210 (US)
Commelinaceae	*Dictyospermum ovalifolium* Wight	Faden 76/474 (US)
Commelinaceae	*Dictyospermum ovatum* Hassk.	Thitimetharoch 426 (US)
Commelinaceae	*Elasis guatemalensis* (C.B.Clarke ex Donn.Sm.) M.Pell.	Heyde 3519 (US)
Commelinaceae	*Elasis hirsuta* (Kunth) D.R.Hunt	Bonpland (2160) (P)
Commelinaceae	*Floscopa africana* (P.Beauv.) C.B.Clarke	Faden 87/11 (US)
Commelinaceae	*Floscopa aquatica* Hua	Fosberg 40431 (US)
Commelinaceae	*Floscopa clarkeana* Kuntze	Schunke 8578 (US)
Commelinaceae	*Floscopa flavida* C.B.Clarke	Faden 96/188 (US)
Commelinaceae	*Floscopa glabrata* (Kunth) Hassk.	Pellegrini 450 (RB)
Commelinaceae	*Floscopa peruviana* Hassk. ex C.B.Clarke	Medeiros 20176 (RB)
Commelinaceae	*Floscopa tanneri* Brenan	Bidgood 2590 (US)
Commelinaceae	*Floscopa scandens* Lour.	Faden 76/447 (US)
Commelinaceae	*Floscopa yunnanensis* D.Y.Hong	Wang 80888 (A)
Commelinaceae	*Geogenanthus ciliatus* G.Brückn.	Killip 29347 (US)
Commelinaceae	*Geogenanthus poeppigii* (Miq.) Faden	Daly 7921 (US)
Commelinaceae	*Geogenanthus rhizanthus* (Ule) G.Brückn.	Plowman 4076 (US)
Commelinaceae	*Gibasis chihuahuensis* (Standl.) Rohweder	Hinton 13944 (US)
Commelinaceae	*Gibasis consobrina* D.R.Hunt	Pringle 6723 (US)
Commelinaceae	*Gibasis geniculata* (Jacq.) Rohweder	Pellegrini 338 (RB)

Commelinaceae	*Gibasis gypsophyla* B.L.Turner	Hinton 22082 (GBH)
Commelinaceae	*Gibasis hintoniorum* B.L.Turner	Hinton 23013 (GBH)
Commelinaceae	*Gibasis karwinskyana* (Schult. & Schult. f.) Rohweder	Pringle 9250 (US)
Commelinaceae	*Gibasis linearis* (Benth.) Rohweder	Palmer 319 (US)
Commelinaceae	*Gibasis matudae* D.R.Hunt	Smith 3913 (US)
Commelinaceae	*Gibasis oaxacana* D.R.Hunt	Hunt 8175 (K)
Commelinaceae	*Gibasis pauciflora* (Urb. & Ekman) D.R.Hunt	Eggers 7361 (US)
Commelinaceae	*Gibasis pellucida* (M.Martens & Galeotti) D.R.Hunt	Pellegrini 5 (RFA)
Commelinaceae	*Gibasis puclhella* (Kunth) Raf.	Matuda 21459 (US)
Commelinaceae	*Gibasis triflora* (M.Martens & Galeotti) D.R.Hunt	Hunt 8130 (K)
Commelinaceae	*Gibasis venustula* (Kunth) D.R.Hunt	Gaona 436 (US)
Commelinaceae	*Gibasoides laxiflora* (C.B.Clarke) D.R.Hunt	Smith 3818 (US)
Commelinaceae	*Matudanthus nanus* (M.Martens & Galeotti) D.R.Hunt	Frame 259 (US)
Commelinaceae	*Murdannia burchellii* (C.B.Clarke) M.Pell.	Burchell 8165 (K)
Commelinaceae	*Murdannia edulis* (Stokes) Faden	Faden 2009/013 (US)
Commelinaceae	*Murdannia engelsii* M.Pell. & Faden	Engels 3474 (RB)
Commelinaceae	*Murdannia gardneri* (Seub.) G.Brückn.	Gardner 4021 (K)
Commelinaceae	*Murdannia japonica* (Thunb.) Faden	Ramamoorthy 335 (US)
Commelinaceae	*Murdannia keisak* (Hassk.) Hand.-Mazz.	Faden 1/04 (US)
Commelinaceae	*Murdannia nudiflora* (L.) Brenan	Faden 77?151 (US)
Commelinaceae	*Murdannia paraguayensis* (C.B.Clarke ex Chodat) G.Brückn.	Hassler 5083 (G)
Commelinaceae	*Murdannia schomburgkiana* (Kunth) G.Brückn.	Schomburgk 842 (B)
Commelinaceae	*Murdannia semifoliata* (C.B.Clarke) G.Brückn.	Moore 541 (BM)
Commelinaceae	*Murdannia sepalosa* (Engl.) C.K.Lee et al.	Faden 74/504 (US)
Commelinaceae	*Palisota albertii* L.Gentil	SI Greenhouse (US no. 1991-071x)
Commelinaceae	*Palisota ambigua* (P.Beauv.) C.B.Clarke	Faden 87/10 (US)
Commelinaceae	*Palisota barteri* Hook.	Faden 86/57 (US)
Commelinaceae	*Palisota bogneri* Brenan	Le Testu 7520 (US)
Commelinaceae	*Palisota brachythyrsa* Mildbr.	Harris 2433 (US)
Commelinaceae	*Palisota bracteosa* C.B.Clarke	Faden 86/48 (US)
Commelinaceae	*Palisota mannii* C.B.Clarke	Faden 86/16 (US)
Commelinaceae	*Palisota thollonii* Hua	Harris 5616 (US)
Commelinaceae	*Plowmanianthus dressleri* Faden & C.R.Hardy	Hardy 236 (US)
Commelinaceae	*Plowmanianthus grandifolius* subsp. *grandifolius* Faden & C.R.Hardy	Hardy 140 (US)
Commelinaceae	*Plowmanianthus grandifolius* subsp. *robustus* C.R.Hardy & Faden	Hardy 200 (US)
Commelinaceae	*Plowmanianthus panamensis* Faden & C.R.Hardy	Hardy 243 (US)
Commelinaceae	*Plowmanianthus perforans* Faden & C.R.Hardy	Vásquez 34244 (US)
Commelinaceae	*Plowmanianthus peruvianus* C.R.Hardy & Faden	Hardy 122 (US)
Commelinaceae	*Pollia hasskarlii* R.S.Rao	Thitimetharoch 411 (US)
Commelinaceae	*Pollia japonica* Thunb.	Charette 1837 (US)
Commelinaceae	*Pollia thyrsiflora* (Blume) Steud.	Merrill 9268 (US)
Commelinaceae	*Polyspatha hirsuta* Mildbr.	Faden 87/13 (US)
Commelinaceae	*Polyspatha oligospatha* Faden	Poulsen 1275 (US)
Commelinaceae	*Polyspatha paniculata* Benth	Faden 86/32 (US)
Commelinaceae	*Pseudoparis cauliflora* H.Perrier	Perrier de la Bâthie 16764 (P)
Commelinaceae	*Pseudoparis monandra* H.Perrier	Gautier 4179 (US)
Commelinaceae	*Pseudoparis tenera* (Baker) Faden	Humbert 48416 (P)
Commelinaceae	*Pseudoparis* sp. nov. ined.	Perrier de la Bâthie 7296 (P)
Commelinaceae	*Sauvallia blainii* Wright ex Hassk.	Wright 3729 (US)
Commelinaceae	*Siderasis albofasciata* M.Pell.	Pellegrini 337 (RB)

Commelinaceae	*Siderasis almeidae* M.Pell. & Faden	Pellegrini 493 (RB)
Commelinaceae	*Siderasis fuscata* (Lodd.) H.E.Moore	Pellegrini 217 (RB)
Commelinaceae	*Siderasis medusoides* M.Pell. & Faden	Fiaschi 3489 (SPF)
Commelinaceae	*Siderasis spectabilis* M.Pell. & Faden	Melo-Filho 1172 (R)
Commelinaceae	*Siderasis zorzanellii* M.Pell. & Faden	Zorzanelli 969 (RB)
Commelinaceae	*Spatholirion calcicola* K.Larsen & S.S.Larsen	Larsen 43586 (US)
Commelinaceae	*Spatholirion decumbens* Fukuoka & N.Kurosaki	Fukuoka T-96210 (KYO)
Commelinaceae	*Spatholirion elegans* (Cherfils) C.Y.Wu	Pételot 4829 (P)
Commelinaceae	*Spatholirion longifolium* (Gagnep.) Dunn	Bartholomew 948 (US)
Commelinaceae	*Spatholirion ornatum* Ridl.	Kerr 3617 (P)
Commelinaceae	*Spatholirion puluongense* Aver.	Averyanov 3957 (LE)
Commelinaceae	*Stanfieldiella axillaris* J.K.Morton	Slayback 94-8 (US)
Commelinaceae	*Stanfieldiella brachycarpa* (Gilg & Ledermann ex Mildbr.) Brenan	Hall 091/93 (US)
Commelinaceae	*Stanfieldiella imperforata* (C.B.Clarke) Brenan	Faden 86/39 (US)
Commelinaceae	*Stanfieldiella oligantha* (Mildbr.) Brenan	Faden 97/14 (US)
Commelinaceae	*Streptolirion lineare* Fukuoka & N.Kurosaki	Peng 20537 (US)
Commelinaceae	*Streptolirion volubile* Edgew.	Henry 4170 (US)
Commelinaceae	*Thyrsanthemum floribundum* (M.Martens & Galeotti) Pichon	Galeotti 4952 (K)
Commelinaceae	*Thyrsanthemum goldianum* D.R.Hunt	Hinton 13082 (K)
Commelinaceae	*Thyrsanthemum longifolium* (M.Martens & Galeotti) M.Pell. & Espejo	Galeotti 4942a (BR)
Commelinaceae	*Tinantia anomala* C.B.Clarke	Tharp 44104 (US)
Commelinaceae	*Tinantia erecta* (Jacq.) Fenzl	Pellegrini 315 (RB)
Commelinaceae	*Tinantia leiocalyx* C.B.Clarke	Hinton 12227 (US)
Commelinaceae	*Tinantia pringlei* (S.Watson) Rohweder	Bartlett 10462 (US)
Commelinaceae	*Tradescantia cerinthoides* Kunth	Pellegrini 445 (RB)
Commelinaceae	*Tradescantia crassula* Link & Otto	Pellegrini 439 (RB)
Commelinaceae	*Tradescantia fluminensis* Vell.	Pellegrini 48 (RB)
Commelinaceae	*Tradescantia tenella* Kunth	Pellegrini 431 (RB)
Commelinaceae	*Tradescantia commelinoides* Schult. & Schult. f.	Breedlove 12239 (US)
Commelinaceae	*Tradescantia praetermissa* M.Pell.	Mandon 1237 (K)
Commelinaceae	*Tradescantia spathacea* Sw.	Pellegrini 499 (RB)
Commelinaceae	*Tradescantia zanonia* (L.) Sw.	Pellegrini 412 (RB)
Commelinaceae	*Tradescantia zebrina* Heynh. ex Bosse	Pellegrini 406 (RB)
Commelinaceae	*Tradescantia ambígua* Mart.	Fraga 3654 (RB)
Commelinaceae	*Tradescantia andrieuxii* C.B.Clarke	Tores 132 (US)
Commelinaceae	*Tradescantia boliviana* (Hassk.) J.R.Grant	Mandon 1239 (K)
Commelinaceae	*Tradescantia crassifolia* Cav.	Rose 216 (US)
Commelinaceae	*Tradescantia brevifolia* (Torr.) Rose	Bigelow 1500-a (NY)
Commelinaceae	*Tradescantia orchidophylla* Rose & Hemsl.	Jones 467 (US)
Commelinaceae	*Tradescantia pallida* (Rose) D.R.Hunt	Palmer s.n. (US 00091625)
Commelinaceae	*Tradescantia pygmaea* D.R.Hunt	Rose 2095 (US)
Commelinaceae	*Tradescantia hirsutiflora* Bush	Faden 76/21 (US)
Commelinaceae	*Tradescantia ohiensis* Raf.	Pellegrini 512 (RB)
Commelinaceae	*Tradescantia pinetorum* Greene	Greene s.n. (US 00044946)
Commelinaceae	*Tradescantia virginiana* L.	Faden 87/1a (US)
Commelinaceae	*Tricarpelema africanum* Faden	Keating 90-11 (US)
Commelinaceae	*Tricarpelema brevipedicellatum* Faden	Evrard 1178 (P)
Commelinaceae	*Tricarpelema chinense* D.Y.Hong	Tang 23593 (PE)
Commelinaceae	*Tricarpelema giganteum* (Hassk.) H.Hara	Long 1110 (US)
Commelinaceae	*Tricarpelema glanduliferum* (J.Joseph & R.S.Rao) R.S.Rao	Harder 5415 (US)

Commelinaceae	*Tricarpelema philippense* (Panigrahi) Faden	Ramos 22082 (US)
Commelinaceae	*Tricarpelema pumilum* (Hallier f.) Faden	Poulsen 187 (K)
Commelinaceae	*Tricarpelema xizangense* D.Y.Hong	Tibet-MacArthur 2050 (US)
Commelinaceae	*Triceratella drummondii* Brenan	Drummond 5780 (K)
Commelinaceae	*Tripogandra amplexans* Handlos	Hinton 9260 (US)
Commelinaceae	*Tripogandra disgrega* (Kunth) Woodson	Sanders 11287 (US)
Commelinaceae	*Tripogandra diuretica* (Mart.) Handlos	Pellegrini 4 (RFA)
Commelinaceae	*Tripogandra glandulosa* (Seub.) Rohweder	Pellegrini 298 (RB)
Commelinaceae	*Tripogandra grandiflora* (Donn.Sm.) Woodson	Sanders 9829 (US)
Commelinaceae	*Tripogandra multiflora* (Sw.) Raf.	Swartz s.n. (BM 000578859)
Commelinaceae	*Tripogandra serrulata* (Vahl) Handlos	Faden 76/181 (US)
Commelinaceae	*Weldenia volcanica* (Benth.) M.Pell. & Espejo	Hartweg s.n. (K 000363199)
Commelinaceae	*Weldenia candida* Schult. f.	Karwinsky 272 (M 0244244)
Philydraceae	*Helmholtzia acorifolia* F.Muell.	Kress 92-3505 (US)
Philydraceae	*Helmholtzia novoguineensis* (K.Krause) Skottsb.	Hoogland 11068 (US)
Philydraceae	*Orthothylax glaberrimus* (Hook. f.) Skottsb.	Constable 22483 (US)
Philydraceae	*Philydrella drummondii* L.G.Adams	Morrison s.n. (CANB 139276)
Philydraceae	*Philydrella pygmaea* subsp. *minima* L.G.Adams	Orchard 4341 (CANB)
Philydraceae	*Philydrella pygmaea* subsp. *pygmaea* (R.Br.) Caruel	Pritzel 471 (US)
Philydraceae	*Philydrum conchinchinense* (Lour.) M.Pell.	Fosberg 37834 (US)
Philydraceae	*Philydrum lanuginosum* Banks & Sol. ex Gaertn.	Johnson 20418 (US)
Haemodoraceae	*Anigozanthos bicolor* Endl. subsp. *bicolor*	Preiss 1417 (P)
Haemodoraceae	*Anigozanthos bicolor* subsp. *decrescens* Hopper	Hopper 773 (PERTH)
Haemodoraceae	*Anigozanthos bicolor* subsp. *exstans* Hopper	Hopper 4169 (PERTH)
Haemodoraceae	*Anigozanthos bicolor* subsp. *minor* (Benth.) Hopper	Hopper 2510 (PERTH)
Haemodoraceae	*Anigozanthos flavidus* DC.	Hopper 809 (PERTH)
Haemodoraceae	*Anigozanthos gabrielae* Domin	Hopper 806 (PERTH)
Haemodoraceae	*Anigozanthos humilis* subsp. *chrysanthus* Hopper	Hopper 2612 (PERTH)
Haemodoraceae	*Anigozanthos humilis* Lindl. subsp. *humilis*	Hopper 749 (PERTH)
Haemodoraceae	*Anigozanthos kalbarriensis* Hopper	George 9604 (PERTH)
Haemodoraceae	*Anigozanthos manglesii* D.Don subsp. *manglesii*	Hopper 757 (PERTH)
Haemodoraceae	*Anigozanthos manglesii* subsp. *quadrans* Hopper	Hopper 1393 (PERTH)
Haemodoraceae	*Anigozanthos onycis* A.S.George	Hopper 4241 (PERTH)
Haemodoraceae	*Anigozanthos preissii* Endl.	Preiss 1413a (P)
Haemodoraceae	*Anigozanthos pulcherrimus* Hook.	George 3221 (PERTH)
Haemodoraceae	*Anigozanthos rufus* Labill.	Hopper 819 (PERTH)
Haemodoraceae	*Anigozanthos viridis* Endl.	Preiss 1415 (P)
Haemodoraceae	*Barberetta aurea* Harv.	Bolus 8709 (K)
Haemodoraceae	*Blancoa canescens* Lindl.	Spjut 6992 (US)
Haemodoraceae	*Conostylis aculeata* R.Br. subsp. *aculeata*	Hopper 703 (PERTH)
Haemodoraceae	*Conostylis aculeata* subsp. *spinuligera* (F.Muell. ex Benth.) Hopper	Hopper 1177 (PERTH)
Haemodoraceae	*Conostylis albescens* Hopper	Hopper 1247 (PERTH)
Haemodoraceae	*Conostylis androstemma* F.Muell.	Crisp 6709 (US)
Haemodoraceae	*Conostylis angustifolia* Hopper	Hopper 276 (PERTH)
Haemodoraceae	*Conostylis argentea* (J.Green) Hopper	Hopper 66 (PERTH)
Haemodoraceae	*Conostylis canteriata* Hopper	Hopper 5185 (PERTH)
Haemodoraceae	*Conostylis caricina* Lindl. subsp. *caricina*	Green 488 (US)
Haemodoraceae	*Conostylis crassinerva* J.Green. subsp. *crassinerva*	Hopper 388 (PERTH)
Haemodoraceae	*Conostylis dielsii* W.Fitzg. subsp. *dielsii*	Hopper 430 (PERTH)
Haemodoraceae	*Conostylis dielsii* subsp. *teres* Hopper	Hopper 442 (PERTH)
Haemodoraceae	*Conostylis drummondii* Benth.	Hopper 55 (PERTH)
Haemodoraceae	*Conostylis festucacea* Endl. subsp. *festucacea*	Hopper 19 (PERTH)

Haemodoraceae	*Conostylis festucacea* subsp. *filifolia* (F.Muell.) Hopper	Hopper 458 (PERTH)
Haemodoraceae	*Conostylis lepidospermoides* Hopper	Hopper 1149 (PERTH)
Haemodoraceae	*Conostylis micrantha* Hopper	Hopper 2468 (PERTH)
Haemodoraceae	*Conostylis misera* Endl.	Hopper 129 (PERTH)
Haemodoraceae	*Conostylis neocymosa* Hopper	Hopper 445 (PERTH)
Haemodoraceae	*Conostylis pauciflora* Hopper subsp. *pauciflora*	Hopper 131 (PERTH)
Haemodoraceae	*Conostylis pauciflora* subsp. *Euryrhipis* Hopper	Hopper 4894 (PERTH)
Haemodoraceae	*Conostylis phathyrantha* Diels	Hopper 568 (PERTH)
Haemodoraceae	*Conostylis prolifera* Benth.	Pritzel 634 (US)
Haemodoraceae	*Conostylis pusilla* Endl.	Hopper 473 (PERTH)
Haemodoraceae	*Conostylis resinosa* Hopper	Hopper 318 (PERTH)
Haemodoraceae	*Conostylis seminuda* Hopper	Hopper 2621 (PERTH)
Haemodoraceae	*Conostylis seorsiflora* F.Muell. subsp. *seorsiflora*	Hopper 79 (PERTH)
Haemodoraceae	*Conostylis setigera* subsp. *dasys* Hopper	Hopper 4707 (PERTH)
Haemodoraceae	*Conostylis setigera* R.Br. subsp. *setigera*	Pritzel 790 (US)
Haemodoraceae	*Conostylis setosa* Lindl.	Green 1726 (US)
Haemodoraceae	*Conostylis stylidoides* F.Muell.	Green 428 (US)
Haemodoraceae	*Conostylis teretifolia* subsp. *planescens* Hopper	Hopper 27 (PERTH)
Haemodoraceae	*Conostylis tomentosa* Hopper	Hopper 5184 (PERTH)
Haemodoraceae	*Conostylis vaginata* Endl.	Hopper 679 (PERTH)
Haemodoraceae	*Cubanicula xanthorrhizos* (C.Wright ex Griseb.) Hopper et al.	Wright 3259 (US)
Haemodoraceae	*Dilatris corymbose* P.J.Bergius	Manning 3129 (NBG)
Haemodoraceae	*Dilatris ixioides* Lam.	Barker 3318 (NBG)
Haemodoraceae	*Dilatris pillansii* W.F.Barker	Pillans 3701 (BOL)
Haemodoraceae	*Paradilatris paniculata* (L. f.) M.Pell.	Bolus 8389 (BOL)
Haemodoraceae	*Paradilatris viscosa* (L. f.) Hopper	Barker 690 (NBG)
Haemodoraceae	*Haemodorum brevicaule* F.Muell.	Chippendale NT 4477 (PERTH)
Haemodoraceae	*Haemodorum brevisepalum* Benth.	Koch N69 (PERTH)
Haemodoraceae	*Haemodorum coccineum* R.Br.	White 8715 (US)
Haemodoraceae	*Haemodorum corymbosum* Vahl	MacKee 8276 (US)
Haemodoraceae	*Haemodorum discolor* T.D.Macfarl.	MacFarlane 1658 (PERTH)
Haemodoraceae	*Haemodorum distichophyllum* Hook.	Davis 1440 (MEL)
Haemodoraceae	*Haemodorum ensifolium* F.Muell.	Gardner 1374 (PERTH)
Haemodoraceae	*Haemodorum laxum* R.Br.	Morrison s.n. (US 00592008)
Haemodoraceae	*Haemodorum paniculatum* Lindl.	Spjut 7192 (US)
Haemodoraceae	*Haemodorum simplex* Lindl.	Pritzel 840 (US)
Haemodoraceae	*Haemodorum spicatum* R.Br.	Morrison s.n. (US 00592017)
Haemodoraceae	*Haemodorum subvirens* F.Muell.	Mueller s.n. (K 000846218)
Haemodoraceae	*Lachnanthes caroliniana* (Lam.) Dandy	Cronquist 5446 (US)
Haemodoraceae	*Macropidia fuliginosa* (Hook.) Druce	Pritzel 443 (US)
Haemodoraceae	*Phlebocarya ciliata* R.Br.	Pritzel 99 (US)
Haemodoraceae	*Phlebocarya filifolia* (F.Muell.) Benth.	Cranfield 1668b (PERTH)
Haemodoraceae	*Phlebocarya pilosissima* (F.Muell.) Benth.	Pritzel 15 (US)
Haemodoraceae	*Phlebocarya teretifolia* (T.D.Macfarl.) M.Pell.	Gardner 9394 (PERTH)
Haemodoraceae	*Pyrrorhiza neblinae* Maguire & Wurdack	Maguire 37108 (US)
Haemodoraceae	*Schiekia orinocensis* (Kunth) Meisn.	Humboldt 843 (P)
Haemodoraceae	*Schiekia silvestris* (Maas & Stoel) Hopper et al.	Prance 15864 (US)
Haemodoraceae	*Schiekia timida* M.Pell. et al.	Forzza 8562 (RB)
Haemodoraceae	*Tribonanthes australis* Endl.	Hickman 2067 (PERTH)
Haemodoraceae	*Tribonanthes brachypetala* Lindl.	Hickman 2022 (PERTH)
Haemodoraceae	*Tribonanthes elongate* E.J.Hickman & Hopper	Hickman 2073 (PERTH)
Haemodoraceae	*Tribonanthes keigheryi* E.J.Hickman & Hopper	Hickman 2065 (PERTH)

Haemodoraceae	*Tribonanthes longipetala* Lindl.	Hickman 2021 (PERTH)
Haemodoraceae	*Tribonanthes monantha* E.J.Hickman & Hopper	Hickman 2048 (PERTH)
Haemodoraceae	*Tribonanthes minor* M.Lyons & Keighery	Hickman 2079 (PERTH)
Haemodoraceae	*Tribonanthes porphyria* E.J.Hickman & Hopper	Hickman 2025 (PERTH)
Haemodoraceae	*Tribonanthes purpurea* T.D.Macfarl. & Hopper	Hickman 2083 (PERTH)
Haemodoraceae	*Tribonanthes uniflora* Lindl.	Hickman 2084 (PERTH)
Haemodoraceae	*Tribonanthes variabilis* Lindl.	Hickman 2053 (PERTH)
Haemodoraceae	*Tribonanthes violacea* Endl.	Hickman 2090 (PERTH)
Haemodoraceae	*Wachendorfia brachyandra* W.F.Barker	Barker 1096 (NBG)
Haemodoraceae	*Wachendorfia multiflora* (Klatt) J.C.Manning & Goldblatt	Barker 4600 (NBG)
Haemodoraceae	*Wachendorfia paniculata* Burm.	Pillans 9138 (BOL)
Haemodoraceae	*Wachendorfia thyrsiflora* Burm.	Pillans 8086 (BOL)
Haemodoraceae	*Xiphidium caeruleum* Aubl.	Perdiz 2376 (RB)
Haemodoraceae	*Xiphidium pontederiiflorum* M.Pell. et al.	Daly 5142 (US)
Pontederiaceae	*Heteranthera callifolia* Rchb. ex Kunth	Hepper 3683 (K)
Pontederiaceae	*Heteranthera catharinensis* C.N.Horn & M.Pell.	Smith 13919 (US)
Pontederiaceae	*Heteranthera dubia* (Jacq.) MacMill.	Horn 410 (UNA)
Pontederiaceae	*Heteranthera gardneri* (Hook. f.) M.Pell.	Gardner 1863 (K)
Pontederiaceae	*Heteranthera limosa* (Sw.) Willd.	Assunção 721 (RB)
Pontederiaceae	*Heteranthera longirachilla* D.J.Sousa & Giul.	Hage 1438 (MBM)
Pontederiaceae	*Heteranthera lutea* (H.Perrier) M.Pell.	Perrier de la Bâthie 7178 (P)
Pontederiaceae	*Heteranthera mexicana* S.Watson	Palmer 1324 (K)
Pontederiaceae	*Heteranthera multiflora* (Griseb.) C.N.Horn	Lorentz 310 (UNA)
Pontederiaceae	*Heteranthera oblongifolia* Mart. ex Schult & Schult. f.	Araújo 38 (RB)
Pontederiaceae	*Heteranthera peduncularis* Benth.	Hartweg 226 (K)
Pontederiaceae	*Heteranthera pumila* M.Pell. & C.N.Horn	Pellegrini 495 (RB)
Pontederiaceae	*Heteranthera reniformis* Ruiz & Pav.	Pellegrini 457 (RB)
Pontederiaceae	*Heteranthera rotundifolia* (Kunth) Griseb.	Walter 6644 (RB)
Pontederiaceae	*Heteranthera seubertiana* Solms	Horn 525 (UNA)
Pontederiaceae	*Heteranthera spicata* C.Presl	Haynes 8618 (UNA)
Pontederiaceae	*Heteranthera zosterifolia* Mart.	Fontana 8316 (RB)
Pontederiaceae	*Pontederia meyeri* (A.G.Schulz) M.Pell. & C.N.Horn	Prance 26220 (NY)
Pontederiaceae	*Pontederia paniculata* Spreng.	Machado 574 (RB)
Pontederiaceae	*Pontederia paradoxa* Mart.	Harley 21401 (K)
Pontederiaceae	*Pontederia crassipes* Mart.	Martius 60 (M)
Pontederiaceae	*Pontederia azurea* Sw.	Martinelli 18669 (RB)
Pontederiaceae	*Pontederia diversifolia* (Vahl) M.Pell. & C.N.Horn	Harley 10248 (RB)
Pontederiaceae	*Pontederia heterosperma* (Alexander) M.Pell. & C.N.Horn	Smith 2290 (NY)
Pontederiaceae	*Pontederia natans* P.Beauv.	s.leg. s.n. (US 00763681)
Pontederiaceae	*Pontederia africana* (Solms) M.Pell. & C.N.Horn	Schweinfurth 2296 (K)
Pontederiaceae	*Pontederia australasica* (Ridl.) M.Pell. & C.N.Horn	Allen 81 (K)
Pontederiaceae	*Pontederia brevipetiolata* (Verdc.) M.Pell. & C.N.Horn	Espírito Santo 2777 (K)
Pontederiaceae	*Pontederia cyanea* (F.Muell.) M.Pell. & C.N.Horn	Leichhardt s.n. (K 000873493)
Pontederiaceae	*Pontederia elata* (Ridl.) M.Pell. & C.N.Horn	Haniff 1208 (K)
Pontederiaceae	*Pontederia hastata* L.	Hermann s.n. (BM 000621681)
Pontederiaceae	*Pontederia korsakovii* (Regel & Maack) M.Pell. & C.N.Horn	Maack s.n. (K 000873544)
Pontederiaceae	*Pontederia plantaginea* Roxb.	Wallich 5096 (K)
Pontederiaceae	*Pontederia vaginalis* Burm. f.	Boeea 8471 (US)
Pontederiaceae	*Pontederia valida* (G.X.Wang & Nagam.) M.Pell. & C.N.Horn	Wong 901001 (KYO)
Pontederiaceae	*Pontederia cordata* L.	Barton s.n. (PH 00038346)

Pontederiaceae	*Pontederia ovalis* Mart.	Pellegrini 474 (RB)
Pontederiaceae	*Pontederia parviflora*	Forzza 8440 (RB)
Pontederiaceae	*Pontederia rotundifolia* L. f.	Alvarenga 952 (RB)
Pontederiaceae	*Pontederia sagittata* C.Presl	Catharino 342 (RB)
Pontederiaceae	*Pontederia subovata* (Seub.) Lowden	Gardner 4022 (NY)
Pontederiaceae	*Pontederia triflora* (Endl. ex Seub.) G.Agostini et al.	Hunt 5919 (K)

Supplementary Table S2

Supplementary Table S2. List of morphological characters and their respective states used in the phylogenetic analysis.

No.	Character: state (state no.)
**1.**	Plant, life cycle, duration: perennial (0); annual (1)
**2.**	Plant, life cycle, perennial, duration of aerial shoots: overwintering (0); dying off during the dry/cold/fire season (1)
**3.**	Plant, clonal reproduction: absent (0); present (1)
**4.**	Plants, sexual expression: monoecious (0); dioecious (1)
**5.**	Habitat, substrate: aquatic or growing in damp environments (0); terrestrial (1); rupicolous (2); epiphytic (3)
**6.**	Habitat, aquatic, emergence: paludal or emergent (0); mostly to completely submersed or rooted-floating (1); free-floating (2); rheophytic (3)
**7.**	Habit, growth, branching, type: sympodial (0); monopodial (1)
**8.**	Habit, growth type: herb (0); vine (1)
**9.**	Habit, growth form: solitary (0); mat/cluster/clump-forming (1)
**10.**	Habit, base: definite (0); indefinite (1)
**11.**	Roots, surface: non-binding (0); sand-binding (1); water-binding/mucilaginous (2)
**12.**	Roots, pubescence: glabrous to pilose (0); lanate (1); arachnoid (2)
**13.**	Roots, function: non-contractile (0); contractile (1)
**14.**	Roots, type: thin (0); tuberous (1); stilt (2)
**15.**	Roots, tuberous, shape: thin and cylindrical (0); spindle-like (1); with a fusiform enlargement at base (2); with a fusiform enlargement at mid-length or apex (3); with a napiform enlargement at base (4); outer ones with a fusiform enlargement at mid-length or apex, the inner ones spindle-like (5); with a globose and sclerified enlargement at apex (6)
**16.**	Roots/Stems, internal colouration: pale (0); yellow to orange to red to maroon (1); vinaceous to purple to black (2)
**17.**	Stems, underground system: absent (0); rhizome (1); corm (2); tuberized stem (3); bulb (4)
**18.**	Stems, underground system, crass or tuberized stem, development: short (0); elongate (1)
**19.**	Stems, posture: prostrate (0); erect to fruticose (1)
**20.**	Stems, consistency: fibrous (0); herbaceous (1); crass (2)
**21.**	Stems, branching: unbranched or branched only at base (0); branched throughout (1); branched in the upper 1/2 or 1/3 (2)
**22.**	Stems, internodes, elongation: elongated (0); contracted (1)
**23.**	Stems, internodes, leaf-opposite line of uniseriate hairs, presence: absent (0); present (1)
**24.**	Stems, node, swelling: slender (0); swollen (1)
**25.**	Stems, secondary branches, flagelliform-shoots, presence: absent (0); present (1)
**26.**	Leaves, dimorphic: absent (0); present (1)
**27.**	Leaves, ptyxis: supervolute to convolute (0); involute (1); conduplicate (2); conduplicate-involute, enclosing the petiole of the preceding leaf (3)
**28.**	Leaves, blade: bifacial (0); unifacial (1); late bifacial (2)
**29.**	Leaves, sheath, projection (ligule): absent (0); present (1)
**30.**	Leaves, sheath, projection (ligule), fusion: free (0); fused to the sheath (1)
**31.**	Leaves, sheath, projection (ligule), shape: truncate (0); flabellate (1); 2-lobed to 2-parted (2); multi-parted (3)

32. Leaves, sheath, projection (ligule), relative length to the sheath: shorter than the sheath or ca. as long as the sheath (0); clearly longer than the sheath (1)
33. Leaves, sheath, projection (ligule), 2-lobed to 2-parted or multiparted, lobes shape: filiform (0); quadrate (1); oblong (2); triangular (3)
34. Leaves, sheath, projection (ligule), consistency: membranous and hyaline (0); chartaceous and semi-opaque to opaque (1)
35. Leaves, sheaths, type: open (0); closed symmetric (1); closed asymmetric (2)
36. Leaves, sheaths, margin: entire (0); scarious (1)
37. Leaves, sheaths, closed, suture scar, presence: absent (0); present (1)
38. Leaves, sheaths, closed, splitting open at maturity: absent (0); present (1)
39. Leaves, immature, duration: early deciduous (0); late deciduous (1); persistent (2)
40. Leaves, immature, phyllotaxy: distichously-alternate (0); spirally-alternate/rosette (1); equitant (2); pseudo-whorled (3)
41. Leaves, immature, distribution: evenly distributed (0); congested at base/apex (1); equitant (2)
42. Leaves, immature, environment: submerged (0); floating or emerse (1)
43. Leaves, immature, insertion: sessile (0); subpetiolate (1)
44. Leaves, immature, blade, posture: pendulous (0); patent to slightly recurved (1); ascending to erect (2); appressed to the soil (3)
45. Leaves, immature, blade, development, lower leaves on the stem: scale-like, sometimes with an open sheath (0); reduced (1); expanded (2)
46. Leaves, immature, blade, shape: ribbon-like or linear to ensiform or loriform (0); oblong to elliptic (1); lanceolate to ovate (2); spathulate to obovate to rotund or cordate (3)
47. Leaves, immature, blade, consistency: membranous (0); chartaceous (1); crass (2); fibrous and coriaceous (3); spongy (4)
48. Leaves, immature, blade, architecture: flat (0); falcate to conduplicate (1); bifacial plicate (2); unifacial plicate (3); bullate (4); cannulate (5); acicular or subterete to terete (6); twisted (7); conduplicate-keeled (8)
49. Leaves, immature, blade, fistulous, presence: absent (0); present (1)
50. Leaves, immature, blade, base, shape: amplexicaulous to truncate (0); cuneate (1); obtuse to rounded to cordate (2)
51. Leaves, immature, blade, base, symmetry: symmetric (0); asymmetric (1)
52. Leaves, immature, blade, base, posterior divisions: absent (0); present (1)
53. Leaves, immature, blade, margin, architecture: flat (0); repandous (1); only the pseudopetiole repandous (2)
54. Leaves, immature, blade, margin, prickle hairs, presence: absent (0); present (1)
55. Leaves, immature, blade, margin, Conostylis-type hairs, type: absent (0); stiff, rigid or tooth-like, patent or upright bristles (1); fine or soft appressed bristles or simple hairs (2); fine and patent bristles (3); stiff bristles clasping the blade (4); plumose (5); papillose (6)
56. Leaves, immature, blade, margin, uniseriate hairs, presence: absent (0); present (1)
57. Leaves, immature, blade, margin, papillae, presence: absent (0); present (1)
58. Leaves, immature, blade, margin, star-shaped idioblast, presence: absent (0); present (1)
59. Leaves, immature, blade, apex, shape: round to obtuse (0); acute (1); acuminate or apiculate to mucronate (2); caudate (3)
60. Leaves, immature, blade, adaxial side, secondary veins, impression: inconspicuous (0); conspicuous (1)
61. Leaves, immature, blades, adaxial side, variegation, presence: absent (0); longitudinal silver/white/light green stripes (1); brown to vinaceous blotches (2); base of leaves turning pink when in flower (3)
62. Leaves, immature, blades, abaxial side, colouration: white (0); green (1); vinaceous to purple to maroon (2)
63. Leaves, mature, production: never produced (0); produced only when flowering (1); always produced (2)
64. Leaves, mature, phyllotaxy: distichously-alternate (0); spirally-alternate (1)

65. Leaves, mature, distribution: distributed along the stem (0); congested (1)
66. Leaves, mature, environment: floating (0); emerse (1)
67. Leaves, mature, petiole, length in relation to the blade: shorter than the blade (0); ca. the same length as or longer than the blade (1)
68. Leaves, mature, petiole, insertion, colouration in relation to the rest of the blade: concolourous (0); conspicuously discolourous (1)
69. Leaves, mature, pulvinus: absent (0); present (1)
70. Leaves, mature, blade, consistency: membranous (0); chartaceous to coriaceous (1)
71. Leaves, mature, blade, overall shape: linear to tapered (0); elliptic to lanceolate to ovate (1); obovate (2); cordate to reniform (3); hastate to sagittate (4); rotund (5)
72. Leaves, mature, blade, posture: pendulous (0); patent or floating (1); erect (2)
73. Leaves, mature, blade, base, posterior divisions: absent (0); present (1)
74. Leaves, mature, blade, base, posterior divisions, apex: round (0); acuminate (1)
75. Leaves, mature, blade, venation, thickened midvein: absent (0); present (1)
76. Synflorescence, leaves, dimorphism, presence: absent (0); present (1)
77. Synflorescence, leaves, sheath: inflated (0); not inflated (1); splitting open with the development of the inflorescence (2); concealing the inflorescence (3); hyaline and bladeless (4)
78. Synflorescence, composition: solitary main florescence (0); main florescence with 1-several coflorescences (1)
79. Synflorescence, structure: terminal or restricted to the apex of the stem (0); mainly axillary to spike-like or at the base of the plant (1)
80. Synflorescence, vivipary, presence: absent (0); present (1)
81. Inflorescence, position: terminal or apparently so (0); axillary or at the base of the plant (1); leaf-opposed (2)
82. Inflorescence, at anthesis, posture: erect (0); pendulous (1); prostrate (2)
83. Inflorescence, post-anthesis/in fruit, posture: the same as during anthesis (0); deflexed (1)
84. Inflorescence, basal bract, development: leaf-like or bracteose (0); vestigial and tubular (1); spathaceous (2); bracteose and bicarinate/bidentate (3)
85. Inflorescence, basal bract, spathaceous, shape: cordate (0); ovate to triangular to broadly ovate to depressed ovate (1); cup-shaped (2)
86. Inflorescence, basal bract, base, connation: free (0); connate (1)
87. Inflorescence, basal bract, margin, posture: straight (0); revolute (1)
88. Inflorescence, emergence: not perforating the leaf-sheath (0); perforating the leaf-sheath (1)
89. Inflorescence, peduncle, development: sessile to subsessile (0); obviously pedunculate (1)
90. Inflorescence, peduncle, accessory bracts: absent (0); present (1)
91. Inflorescence, peduncle, accessory bracts, bud, development: simple buds (0); primordial inflorescence buds (1)
92. Inflorescence, main axis, development: abbreviated (0); basally elongated, apically abbreviated (1); elongated (2)
93. Inflorescence, main axis, developed, architecture: straight (0); zigzag (1); with a 90° torsion (2)
94. Inflorescence, main axis, internal consistency: solid (0); fistulose (1)
95. Inflorescence, secondary branches, type: monochasium (0); dichasium (1); branched cyme (2)
96. Inflorescence, secondary branches, branched cyme, number of branches: bifurcate (0); trifurcate (1)
97. Inflorescence, secondary branches, persistency: persistent (0); detaching at the end of the flowering season (1)
98. Inflorescence, secondary branches, number per main florescence: one (0); two (1); three to several or variable (2)
99. Inflorescence, secondary branches, number per node of the main florescence: one (0); two to several or variable (1)
100. Inflorescence, secondary branches, arrangement: alternate (0); subopposite to opposite (1); subverticillate to verticillate (2); fasciculate to glomerulate (3); side-by-side (4)
101. Inflorescence, secondary branches, peduncle, development: sessile (0); pedunculate (1)
102. Inflorescence, secondary branches, peduncle, pedunculate, length: short (0); long (1); very long (2)

103. Inflorescence, secondary branches, flowering axis, insertion: straight (0); geniculate (1)

104. Inflorescence, secondary branches, flowering axis, insertion, degree of reflection: 0° (0); 30–60° (1); 180° (2)

105. Inflorescence, secondary branches, axis, thickness: thin (0); stout to greatly swollen (1)

106. Inflorescence, secondary branches, axis, internodes, development: contracted (0); elongate (1); medial internode contracted, remaining ones elongate (2)

107. Inflorescence, secondary branches, fusion, to the main axis of the inflorescence: free (0); fused (1)

108. Inflorescence, secondary branches, fusion, to the peduncle of the cincinnus: free (0); fused (1)

109. Inflorescence, secondary branches, fusion, to the peduncle of the cincinnus, degree of fusion: fused only at base (0); completely fused (1)

110. Inflorescence, secondary branches, fusion, to each other: free (0); fused (1); partially fused (2)

111. Inflorescence, secondary branches, bracts, presence: absent (0); present (1); present only on lower cincinnus of the thyrse, remaining cincinni ebracteate (2)

112. Inflorescence, secondary branches, bracts, persistency: caduceus (0); persistent (1)

113. Inflorescence, secondary branches, bracts, posture: patent to ascending (0); deflexed (1)

114. Inflorescence, secondary branches, bracts, development: vestigial (0); bracteose (1); frondose (2); basal frondose, apical bracteose (3)

115. Inflorescence, secondary branches, bracts, frondose, aspect: leaf-like (0); spathaceous (1)

116. Inflorescence, secondary branches, bracts, similarity to each other: equal to subequal (0); unequal (1); the first markedly different from the rest (2)

117. Inflorescence, secondary branches, bracts, architecture: flat (0); complicate or canaliculate to folded (1)

118. Inflorescence, secondary branches, bracts, overlap: not overlapping (0); overlapping one another (1)

119. Inflorescence, secondary branches, bracts, fusion, to the secondary branch axis: free (0); fused (1)

120. Inflorescence, secondary branches, bracts, fusion, to each other: free (0); fused only at the base (1); completely fused/cup-shaped (2)

121. Inflorescence, secondary branches, bracts, base, inflation: non-saccate (0); saccate (1)

122. Inflorescence, secondary branches, bracts, apex: acute (0); with an apical gland (1); cleft or lobed (2); erose (3)

123. Inflorescence, secondary branches, bracts, apex, with an apical gland, shape: acute (0); filiform (1)

124. Inflorescence, secondary branches, bracts, supernumerary bracts: absent (0); present (1)

125. Inflorescence, secondary branches, anthesis pattern, direction: basal branches first (0); apical branches first (1)

126. Inflorescence, secondary branches, anthesis pattern, flowers: sequential (0); simultaneous (1)

127. Bracteoles, development: absent to vestigial (0); inconspicuous (1); conspicuous (2); bracteose to leaf-like and equal to the cincinni bracts (3); leaf-like but distinct from the cincinni bracts (4); spathaceous (5)

128. Bracteoles, arrangement: zig-zag (0); tightly imbricate in 1 whorl (1); tightly imbricate in 2 whorls (2); spirally-alternate or one-sided (3)

129. Bracteoles, persistency: caducous (0); persistent (1)

130. Bracteoles, consistency: membranous (0); herbaceous (1); paleaceous (2); scarious (3); crass (4); chartaceous to coriaceous (5); papyraceous (6)

131. Bracteoles, architecture: flat (0); complicate or canaliculate (1); cup-shaped (2); navicular (3); tubular (4); involving the cincinnus (5); margin crispate (6)

132. Bracteoles, base, conation: free (0); perfoliate (1)

133. Bracteoles, margin: entire (0); erose (1)

134. Bracteoles, aspect: hyaline (0); centre opaque, margin translucent (1); opaque (2)

135. Flowers, number, per main florescence: one (0); two (1); three to several (2)

136. Flowers, number, per secondary branch: one (0); two (1); three to several (2)

137. Flowers, arrangement, in the cincinnus: solitary (0); clustered (1); forming a pseudanthium (2)

138. Flowers, scent, presence: scentless (0); scented (1)

139. Flowers, buds, shape: globose (0); ovoid (1); ellipsoid or fusiform or oblongoid (2); obovoid (3); obpyriform (4); narrowly ovoid (5)

140. Flowers, sexual expression: all bisexual (0); occasionally unisexual, randomly distributed (1); lower cincinnus with bisexual remaining cincinni staminate (2); all unisexual (3)
141. Flowers, cleistogamy: absent (0); present (1)
142. Flowers, heterostyly, enantiostyly: absent (0); present (1)
143. Flowers, heterostyly, style morphs, number: monostylous (0); pseudomonostylous (1); tristylous (2)
144. Flowers, display angle: without torsion (0); with 60° torsion (1); resupinate (2)
145. Flowers, overall symmetry: actinomorphic (0); zygomorphic or asymmetric (1)
146. Flowers, pedicel, length: sessile to subsessile (0); ca. 1/2 the length of the floral bud or around the same length as the floral bud (1); longer than the floral bud (2); more than three times longer than the floral bud (3)
147. Flowers, pedicel, fusion to each other: free (0); fused (1)
148. Flowers, pedicel, thickness: thin (0); stout (1)
149. Flowers, pedicel, consistency, at post-anthesis: herbaceous (0); lignified (1); stout and fibrous (2)
150. Flowers, pedicel, apical gibbae, presence: absent (0); present (1)
151. Flowers, pedicel, pubescence, long hairs: absent (0); setose and eglandular (1); glandular (2); Geogenanthus-type glandular hair (3); with 3 longitudinal lines of acicular hairs (4)
152. Flowers, pedicel, posture at pre-anthesis: upright to erect (0); patent (1); deflexed (2); reflexed (3)
153. Flowers, pedicel, posture at anthesis: upright to erect (0); patent (1); geniculate (2); pendulous (3); geniculate (4)
154. Flowers, pedicel, posture at post-anthesis: decurved (0); upright to erect (1); recurved (2); laterally spreading (3); spirally-coiled (4); oblique (5); pendulous (6)
155. Flowers, pedicel, in fruit: persistent (0); persistent and greatly elongate (1); deciduous (2)
156. Flowers, septal nectaries, presence: absent (0); present (1)
157. Flowers, septal nectaries, number: two (0); three (1)
158. Flowers, septal nectaries, position: infralocular (0); interlocular (1); supralocular (2)
159. Flowers, septal nectaries, commissure slits, presence: absent (0); present (1)
160. Flowers, septal nectaries, supra-ovarian nectar pockets, presence: absent (0); present (1)
161. Flowers, hypanthium, presence: absent (0); present (1)
162. Flowers, hypanthium, supra-ovarian constriction, presence: absent (0); present (1)
163. Flowers, shape: flat (0); tubular (1)
164. Perianth, shape, tubular, type: infundibuliform (0); tubular (1); bilabiate (2); campanulate (3); hypocrateriform (4); rotate (5); urceolate (6)
165. Perianth, whorls, fusion between whorls: free from each other (0); fused (1)
166. Perianth, whorls, fusion between whorls, fused: basally fused (0); forming a conspicuous tube (1); the upper three tepals basally to medially fused (2); the upper five tepals basally to medially fused, the remaining one free (3)
167. Perianth, whorls, similarity to each other: homochlamydeous (0); heterochlamydeous (1)
168. Perianth, whorls, outer, aspect: sepaloid (0); petaloid (1); spathaceous (2)
169. Perianth, whorls, inner, aspect: sepaloid (0); petaloid (1)
170. Perianth, tube, curvature: straight (0); falcate (1)
171. Perianth, tube, collar-like base, presence: absent (0); present (1)
172. Perianth, tube, medial or sub-apical constriction, presence: absent (0); present (1)
173. Perianth, aestivation: imbricate (0); valvate (1)
174. Perianth, longitudinal splitting, presence: absent (0); present (1)
175. Perianth, longitudinal splitting, perianth lobes relative length to the tube: shorter than the tube (0); ca. as long to longer than the tube (1)
176. Perianth, apertures, presence: absent (0); present (1)
177. Perianth, at post-anthesis: coiled but apex withering or deliquescent (0); marcescent (1); entire inner whorl withering or deliquescent, outer whorl variable (2); herbaceous (3); coriaceous (4); succulent (5)
178. Perianth, at post-anthesis, coiling type: slightly spirally-coiled to patent (0); strongly spirally-coiled (1); involute (2)

179. Perianth, at post-anthesis, marcescent, lobes, becoming tooth-like, presence: absent (0); present (1)
180. Perianth, overall colour: green to yellow to orange (0); white to cream to grey or hyaline (1); pink to mauve (2); blue to lilac to purple (3); red to vinaceous to maroon or black (4)
181. Perianth, red or suffused with red at post-anthesis, presence: absent or perianth already red at anthesis (0); present (1)
182. Perianth, hypanthium/ovary, colouration in relation to the remaining of the perianth: equal to similar (0); different (1)
183. Perianth, hypanthium/ovary, colouration in relation to the remaining of the perianth, different, specific colouration: orange (0); red (1); vinaceous (2)
184. Perianth, merosity: dimerous (0); tetramerous (1); pseudotetramerous (2); trimerous (3)
185. Perianth, pseudotetramerous, outer lobes, connation: free (0); basally connate (1)
186. Perianth, pseudotetramerous, outer lobes, shape: broadly oblong (0); lanceolate or ovate (1); rhomboid (2)
187. Perianth, pseudotetramerous, outer lobes, architecture: flat (0); plicate (1); margin involute (2)
188. Perianth, pseudotetramerous, outer lobes, posture: patent (0); oblique to deflexed (1)
189. Perianth, pseudotetramerous, outer lobes, margin: entire (0); crenulate (1); erose (2)
190. Perianth, pseudotetramerous, outer lobes, pubescence: glabrous (0); pubescent (1)
191. Perianth, pseudotetramerous, inner lobes, shape: oblong to rectangular (0); linear-spathulate (1); spathulate to obovate (2); widely obtrullate (3)
192. Perianth, pseudotetramerous, inner lobes, architecture: straight or curved outwards (0); curved inwards (1); repandous (2)
193. Perianth, pseudotetramerous, inner lobes, margin: entire (0); dentate (1)
194. Perianth, pseudotetramerous, inner lobes, apex, shape: obtuse (0); tridentate (1)
195. Perianth, pseudotetramerous, inner lobes, pubescence: glabrous (0); basally pubescent (1)
196. Perianth, lobes, overall arrangement: 3+3 (0); 5+1 (1); 3+1 or 3+2+1 (2)
197. Perianth, lobes, posture: erect (0); patent (1); apex revolute to slightly recurved (2); deflexed (3); connivent (4); incurved (5); concave (6)
198. Perianth, lobes, outer whorl, length, relative to each other: medial lobe shorter than the laterals (0); all equal (1); medial lobe longer than the laterals (2)
199. Perianth, lobes, outer whorl, width, relative to each other: medial sepal narrower than the laterals (0); all equal to subequal (1); medial sepal broader than the lateral (2)
200. Perianth, lobes, outer whorl, size, length relative to the inner whorl: outer shorter than the inner (0); equal to subequal (1); outer longer than the inner (2)
201. Perianth, lobes, outer whorl, width, relative to the inner whorl: outer narrower than the paired inner lobes (0); outer equal or subequal to the paired inner lobes (1); outer broader than the paired inner lobes (2)
202. Perianth, lobes, outer whorl, consistency: membranous (0); chartaceous (1); succulent or fleshy (2); herbaceous (3); paleaceous (4); coriaceous (5)
203. Perianth, lobes, outer whorl, in fruit, development: persistent to slightly accrescent (0); obviously accrescent (1); caducous (2)
204. Perianth, lobes, outer whorl, in fruit, size relative to the fruit (superior ovary): smaller than the fruit (0); same size as the fruit (1); longer than the fruit (2)
205. Perianth, lobes, inner whorl, length, relative to each other: medial lobe shorter than the laterals (0); equal to subequal (1); medial lobe longer than the laterals (2)
206. Perianth, lobes, inner whorl, width, relative to each other: medial lobe narrower than the laterals (0); equal to subequal (1); medial lobe broader than the laterals (2)
207. Perianth, lobes, gland-dots, presence: absent (0); 2–3 apical dots (1); several distal dots (2)
208. Perianth, lobes, shape, between one another: all equal or subequal (0); equal to subequal in the same whorl (1); different in the same whorl (2)
209. Perianth, lobes, apex, glandular papillae, presence: absent (0); present (1)
210. Perianth, lobes, apex, dark-mucronate, presence: absent (0); present (1)
211. Perianth, lobes, central anterior lobe, base: flat, lacking projections or folds (0); with a basal fold (1); basal flanges (2)
212. Perianth, lobes, nectar guide, presence: absent (0); present (1)

213. Perianth, lobes, nectar guide, location: anterior lobes (0); posterior lobes (1); base of all lobes (2)

214. Perianth, lobes, nectar guide, morphology: bands (0); spots, blurs or gradients (1)

215. Perianth, lobes, nectar guide, colouration: maroon to atro-vinaceous to black (0); mauve to purple or blue (1); green (2); yellow (3); orange to red (4); lighter than the remaining lobe or white (5)

216. Perianth, lobes, nectar guide, spots, number: one (0); two (1); three (2)

217. Sepals, connation: free (0); connate in at least one point (1)

218. Sepals, subapical gland, presence: absent (0); present (1)

219. Sepals, calyx, symmetry: actinomorphic (0); zygomorphic (1)

220. Sepals, margin, integrity: entire (0); erose at apex (1); sparsely papillose at apex (2)

221. Sepals, margin, thickness: as thick as the remaining sepal (0); margin hyaline but as thick as the remaining sepal (1); membranous and much thinner than the remaining sepal (2)

222. Sepals, transparency: opaque (0); completely hyaline (1)

223. Sepals, ornamentation: absent (0); dorsal keel present in one sepal (1); dorsal keel present in all sepals (2); striated (3); dorsally 3-costate (4)

224. Sepals, medial sepal, shape: elliptic to broadly elliptic (0); lanceolate to ovate to triangular (1); obovate to spatulate (2); rhomboid to orbicular (3); linear to oblong (4); larger and strongly cucullate (5)

225. Sepals, lateral sepals, shape: elliptic to broadly elliptic (0); lanceolate to ovate to triangular (1); obovate to spatulate (2); rhomboid (3); linear to oblong (4)

226. Petals, corolla, symmetry: actinomorphic (0); zygomorphic (1)

227. Petals, connation: free (0); connate in at least one point (1); paired petals connate, medial fused to the stamens (2)

228. Petals, senescence: withering (0); deliquescent (1)

229. Petals, indumentum, adaxial side: glabrous (0); only base with glandular macrohairs (1); completely glandular macrohairs (2); base bearded with non-moniliform uniseriate hairs (3); with branched multiseriate hairs (4); with dendritic hairs (5); bearded with moniliform hairs at base (6); margin ciliate with papillose hairs (7)

230. Petals, margin, integrity: glabrous or entire (0); crenulate (1); fimbriate to erose (2); ciliate with non-moniliform hairs throughout (3); base barbate with moniliform hairs (4); barbate with moniliform hairs throughout or at apex (5)

231. Petals, paired petals, claw, presence: absent (0); present (1)

232. Petals, paired petals, claw, length: shorter than the blade (0); ca. the length of the blade (1); longer than the blade (2)

233. Petals, paired petals, claw, colour compared to the blade: concolourous (0); lighter or hyaline (1); darker (2)

234. Petals, paired petals, blade, overall, shape: elliptic (0); lanceolate to ovate or triangular (1); rhomboid to orbicular or reniform (2); spatulate to obovate (3); linear to oblong (4); cucullate-spathulate to cucullate-obovate (5); cucullate-reniform (6)

235. Petals, paired petals, blade, base, shape: cuneate to obtuse (0); rounded or auriculate (1); truncate (2)

236. Petals, paired petals, blade, apex, shape: obtuse to rounded (0); acute (1); acuminate (2); emarginate-mucronate (3); trilobed (4); emarginate (5)

237. Petals, paired petals, blade, overall colour: white to cream to grey or hyaline (0); blue (1); lilac to purple or pink to mauve (2); yellow (3); green (4); orange to apricot (5); red (6); vinaceous to maroon (7); black (8)

238. Petals, paired petals, colour, base: concolourous (0); white basal third (1); red to orange base (2)

239. Petals, medial petal, claw: absent (0); present (1)

240. Petals, medial petal, claw, colour compared to the blade: concolourous (0); lighter or hyaline (1)

241. Petals, medial petal, blade, architecture: similar to the paired petals (0); different from the paired petals (1)

242. Petals, medial petal, blade, architecture, different from the paired petals: involute (0); cucullate (1); cup- to boat- to slipper-shaped (2); revolute (3); flat (4)

243. Petals, medial petal, blade, overall, shape: elliptic to trullate (0); lanceolate to ovate or triangular (1); rhomboid to orbicular or reniform (2); spatulate to obovate (3); linear to oblong (4); cucullate-spathulate to cucullate-obovate (5)

244. Petals, medial petal, blade, base, shape: cuneate to obtuse (0); truncate (1)

245. Petals, medial petal, blade, apex, shape: obtuse to rounded (0); acute (1); acuminate (2); emarginate-mucronate (3); trilobed (4); emarginate (5)

246. Petals, medial petal, blade, overall colour: white to cream to grey or hyaline (0); blue (1); lilac to purple or pink to mauve (2); yellow (3); green (4); orange to apricot (5); red (6); vinaceous to maroon (7); black (8)

247. Petals, medial petal, colour, base: concolourous (0); white basal third (1); medially extending towards the apex of the petal (2); red to orange base (3)

248. Androecium, symmetry: actinomorphic (0); zygomorphic (1); asymmetric (2)

249. Androecium, stamens, diversity: monomorphic (0); dimorphic (1); unequal (2)

250. Androecium, stamens, outer antesepalous, presence: absent or microscopic (0); present (1)

251. Androecium, stamens, inner antesepalous, presence: absent or microscopic (0); present (1)

252. Androecium, stamens, outer antepetalous, presence: absent or microscopic (0); present (1)

253. Androecium, stamens, inner antepetalous, presence: absent or microscopic (0); present (1)

254. Androecium, filaments, insertion, position: straight (0); oblique (1)

255. Androecium, filaments, insertion, levels: one (0); two (1); three (2)

256. Androecium, filaments, connation: all free (0); inner antepetalous stamens conate with anterior ones or anterior conate (1); anterior connate (2); all connate (3); 5 connate and 1 free (4)

257. Androecium, filaments, fusion to the perianth: free (0); epitepalous (1); fused only with the petals/inner tepals (2); fused with the posterior outer perianth lobe and to the inner lobes (3); fused with the medial petal (4)

258. Androecium, filaments, fusion to the inner perianth lobes (pseudotetramerous), degree of fusion: basally fused (0); fused for the basal third or half (1)

259. Androecium, filaments, perianth-filament tube, presence: absent (0); tepalo-staminal (1); petalo-staminal (2)

260. Androecium, filaments, thickness: thin (0); stout (1)

261. Androecium, filaments, shape, transverse section: cylindrical throughout (0); basally inflated (1); medially inflated (2); apically inflated (3); flattened (4); medially concave (5)

262. Androecium, filaments, hood-like structure, presence: absent (0); present (1)

263. Androecium, filaments, hood-like structure, apex, shape: truncate (0); tubular (1)

264. Androecium, filaments, appendage, presence: absent (0); tooth-shaped (1); flat and thickened (2)

265. Androecium, filaments, appendage, flat and thickened, apex, shape and size: small and bifid (0); medium-sized and dactyliform to slightly petaloid (1); large and dentate/crested (2)

266. Androecium, filaments, appendage, flat and thickened, colouration: white to cream (0); yellow to orange (1)

267. Androecium, filaments, appendage, flat and thickened, projection in relation to the anther: below the anther (0); at the same level as the anther (1); above the anther (2)

268. Androecium, filaments, lower stamens held inside the cup-shaped medial petal: not held by the medial petal (0); only during the beginning of the anthesis (1); throughout the anthesis (2)

269. Androecium, filaments, relative size, within the same whorl, antesepalous: outer filament shorter, inner longer (0); equal (1); outer filament longer, inner shorter (2)

270. Androecium, filaments, relative size, within the same whorl, antepetalous: outer filament shorter, inner longer (0); equal (1); outer filament longer, inner shorter (2)

271. Androecium, filaments, relative size, between whorls: outer whorl shorter, inner whorl longer (0); equal (1); outer whorl longer, inner whorl shorter (2); whorls with heteromorphic stamens, thus not comparable (3)

272. Androecium, filaments, relative size, posterior stamens: antesepalous shorter, antepetalous longer (0); equal (1); antesepalous longer, antepetalous shorter (2)

273. Androecium, filaments, relative size, anterior stamens: antesepalous longer, antepetalous shorter (0); equal (1); antesepalous shorter, antepetalous longer (2)

274. Androecium, filaments, outer antesepalous stamen, curvature, at anthesis: straight (0); curved (1); descending-falcate (2)

275. Androecium, filaments, inner antesepalous stamens, curvature, at anthesis: straight (0); curved (1); twisted (2); descending-falcate (3)

276. Androecium, filaments, outer antepetalous stamen, curvature, at anthesis: straight (0); curved (1); descending-falcate (2); spirally-coiled (3)

277. Androecium, filaments, inner antepetalous stamens, curvature, at anthesis: straight (0); curved (1); twisted (2)

278. Androecium, filaments, curvature, at post-anthesis: straight (0); curved (1); coiled (2); apex spirally-coiled (3); flaccid and pointing outwards (4)

279. Androecium, filaments, outer antesepalous stamen, indumentum: absent (0); present (1)

280. Androecium, filaments, outer antesepalous stamen, indumentum, density: sparse (0); dense (1)

281. Androecium, filaments, outer antesepalous stamen, indumentum, hair, type: glandular macrohair (0); simple macrohair (1); hook hair (2); moniliform hair (3); uniseriate hairs with dumbbell-shaped cells (4)

282. Androecium, filaments, outer antesepalous stamen, indumentum, distribution: basal (0); up to the middle of the filament (1); apical (2); tufted at base and apex (3); the whole filament (4)

283. Androecium, filaments, outer antesepalous stamen, indumentum, length of the hair: shorter than 1/2 the length of the filament (0); ca. 1/2 the length of the filament (1); ca. as long as the filament (2)

284. Androecium, filaments, inner antesepalous stamens, indumentum: absent (0); present (1)

285. Androecium, filaments, inner antesepalous stamens, indumentum, density: sparse (0); dense (1)

286. Androecium, filaments, inner antesepalous stamens, indumentum, hair, type: glandular macrohair (0); simple macrohair (1); hook hair (2); moniliform hair (3); uniseriate hairs with dumbbell-shaped cells (4); brightly coloured macrohair (5)

287. Androecium, filaments, inner antesepalous stamens, indumentum, distribution: basal (0); up to the middle of the filament (1); apical (2); tufted at base and apex (3); the whole filament (4); upper half (5)

288. Androecium, filaments, inner antesepalous stamens, indumentum, length of the hair: shorter than 1/2 the length of the filament (0); ca. 1/2 the length of the filament (1); ca. as long as the filament (2)

289. Androecium, filaments, antesepalous stamens, indumentum, arrangement: evenly distributed (0); in a ring or single patch (1)

290. Androecium, filaments, outer antepetalous stamen, indumentum: absent (0); present (1)

291. Androecium, filaments, outer antepetalous stamen, indumentum, density: sparse (0); dense (1)

292. Androecium, filaments, outer antepetalous stamen, indumentum, hair, type: glandular macrohair (0); simple macrohair (1); hook hair (2); moniliform hair (3); uniseriate hairs with dumbbell-shaped cells (4); brightly-coloured macrohair (5)

293. Androecium, filaments, outer antepetalous stamen, indumentum, distribution: basal (0); up to the middle of the filament (1); apical (2); tufted at base and apex (3); the whole filament (4); upper half (5)

294. Androecium, filaments, outer antepetalous stamen, indumentum, length of the hair: shorter than 1/2 the length of the filament (0); ca. 1/2 the length of the filament (1); ca. as long as the filament (2)

295. Androecium, filaments, inner antepetalous stamens, indumentum: absent (0); present (1)

296. Androecium, filaments, inner antepetalous stamens, indumentum, density: sparse (0); dense (1)

297. Androecium, filaments, inner antepetalous stamens, indumentum, hair, type: glandular macrohair (0); simple macrohair (1); hook hair (2); moniliform hair (3); uniseriate hairs with dumbbell-shaped cells (4)

298. Androecium, filaments, inner antepetalous stamens, indumentum, distribution: basal (0); up to the middle of the filament (1); apical (2); tufted at base and apex (3); the whole filament (4)

299. Androecium, filaments, inner antepetalous stamens, indumentum, length of the hair: shorter than 1/2 the length of the filament (0); ca. 1/2 the length of the filament (1); ca. as long as the filament (2)

300. Androecium, filaments, antepetalous stamens, indumentum, arrangement: evenly distributed (0); in a ring or single patch (1); in 2 patches (2)

301. Androecium, connectives, fertile stamens, appendages, dorsal horn-like, presence: absent (0); present (1)

302. Androecium, connectives, fertile stamens, appendages, apical, presence: absent (0); eglandular (1); glandular (2)

303. Androecium, connectives, fertile stamens, development, outer antesepalous stamen: contracted (0); expanded (1)

304. Androecium, connectives, fertile stamens, shape, outer antesepalous stamen: oblong to elliptic (0); quadrangular to slightly curved (1); flabellate (2); rhomboid (3); dumbbell-shaped (4); saddle-shaped (5); cordate to sagittate to linear-tapered (6); shield-shaped (7); trapezoid to V-shaped (8); hastate (9); obdeltoid (10)

305. Androecium, connectives, fertile stamens, development, inner antesepalous stamens: contracted (0); expanded (1)

306. Androecium, connectives, fertile stamens, shape, inner antesepalous stamens, shape: oblong to elliptic (0); quadrangular to slightly curved (1); flabellate (2); rhomboid or sagittate (3); obdeltoid or dumbbell-shaped (4); saddle-shaped (5); cordate to hastate to linear-tapered (6); shield-shaped (7); trapezoid to V-shaped (8)

307. Androecium, connectives, fertile stamens, development, outer antepetalous stamen: contracted (0); expanded (1)

308. Androecium, connectives, fertile stamens, shape, outer antepetalous stamen: oblong to elliptic (0); quadrangular to slightly curved (1); flabellate (2); rhomboid or sagittate (3); obdeltoid or dumbbell-shaped (4); saddle-shaped (5); cordate to hastate to linear-tapered (6); shield-shaped (7); trapezoid to V-shaped (8); obsaddle-shaped (9); strongly convex and maroon-spotted (11)

309. Androecium, connectives, fertile stamens, development, inner antepetalous stamens: contracted (0); expanded (1)

310. Androecium, connectives, fertile stamens, development, inner antepetalous stamens: oblong to elliptic (0); quadrangular to slightly curved (1); flabellate (2); rhomboid or sagittate (3); obdeltoid or dumbbell-shaped (4); saddle-shaped (5); cordate to hastate to linear-tapered (6); shield-shaped (7); trapezoid to V-shaped (8); butterfly-shaped (9)

311. Androecium, anthers, size, relative to the filaments: 3–6 times shorter than the filaments (0); ca. the same length as the filaments (1); ca. 1–2 times longer than the filaments (2); 3–4 times longer than the filaments (3); anthers sessile to subsessile (4)

312. Androecium, anthers, 3–4 times longer than the filaments, overall colour: white to cream, with anther sacs apically pink to purple to blue (0); yellow (1)

313. Androecium, anthers, posture: connivent or all pointing towards the centre of the flower (0); all straight, pointing upwards or pointing outwards of the flower (1); 4 pointing towards the centre of the flower + 2 lower pointing outwards (2); posterior +/- straight, paired anterior curved inwards, medial anterior curved towards the centre of the flower (3); pointing downwards (4); connivent or pointing towards the centre of the flower, except for an odd straight stamen (5); reclined against the perianth lobes (6)

314. Androecium, anthers, indumentum: glabrous (0); barbate with moniliform hairs (1)

315. Androecium, anthers, indumentum, barbate with moniliform hairs, distribution: restricted to the margins (0); dorsally and along the margins (1)

316. Androecium, anthers, insertion, angle: straight (0); upwards-geniculate (1); outwards-geniculate (2)

317. Androecium, anthers, insertion, position, outer antesepalous stamen: basifixed (0); dorsifixed (1); basifixed-versatile (2); medifixed-versatile (3); apicifixed (4); sub-basifixed (5)

318. Androecium, anthers, insertion, position, inner antesepalous stamens: basifixed (0); dorsifixed (1); versatile (2); medifixed-versatile (3); basifixed-versatile (4)

319. Androecium, anthers, insertion, position, outer antepetalous stamen: basifixed (0); dorsifixed (1); versatile (2); medifixed-versatile (3); basifixed-versatile (4)

320. Androecium, anthers, insertion, position, inner antepetalous stamens: basifixed (0); dorsifixed (1); versatile (2); medifixed-versatile (3); basifixed-versatile (4)

321. Androecium, anthers, curvature, at anthesis: straight (0); falcate (1)

322. Androecium, anthers, curvature, at post-anthesis: straight (0); falcate to coiled (1)

323. Androecium, anthers, anther sacs, symmetry: symmetric (0); slightly asymmetric (1); strongly asymmetric (2)

324. Androecium, anthers, fertile stamens, anther sacs, position, outer antesepalous stamen: parallel (0); divergent (1); base divergent, apex parallel (2); base divergent, apex connivent (3); parallel but divergent at base and apex (4); parallel but connivent at base and apex (5)

325. Androecium, anthers, fertile stamens, anther sacs, position, inner antesepalous stamens: parallel (0); divergent (1); base divergent, apex connivent (2)

326. Androecium, anthers, fertile stamens, anther sacs, position, outer antepetalous stamen: parallel (0); divergent (1); base divergent, apex connivent (2)

327. Androecium, anthers, fertile stamens, anther sacs, position, inner antepetalous stamens: parallel (0); divergent (1); base divergent, apex connivent (2)

328. Androecium, anthers, fertile stamens, anther sacs, shape, outer antesepalous stamen: linear to elongate (0); elliptic (1); round (2); spirally-coiled (3); reniform to C-shaped (4); drip-shaped (5); coiled on both ends (6); hook-shaped (7)

329. Androecium, anthers, fertile stamens, anther sacs, shape, inner antesepalous stamens: linear to elongate (0); elliptic (1); round (2); spirally-coiled (3); reniform to C-shaped (4); drip-shaped (5); coiled on both ends (6)

330. Androecium, anthers, fertile stamens, anther sacs, shape, outer antepetalous stamen: linear to elongate (0); elliptic (1); round (2); spirally-coiled (3); reniform to C-shaped (4); drip-shaped (5); coiled on both ends (6)

331. Androecium, anthers, fertile stamens, anther sacs, shape, inner antepetalous stamens: linear to elongate or drip-shaped (0); elliptic (1); round (2); spirally-coiled (3); reniform to C-shaped (4); drip-shaped (5); coiled on both ends (6)

332. Androecium, anthers, persistency: all persistent (0); at least two consistently caducous (1)

333. Androecium, anthers, dehiscence: rimose (0); functionally poricidal or truly poricidal (1)

334. Androecium, anthers, dehiscence, rimose and rimose but functionally poricidal, opening direction: base to apex (0); apex to base (1)

335. Androecium, anthers, dehiscence, rimose and rimose but functionally poricidal, slit position, outer antesepalous stamen: latrorse (0); introrse (1); extrorse (2)

336. Androecium, anthers, dehiscence, rimose and rimose but functionally poricidal, slit position, inner antesepalous stamens: latrorse (0); introrse (1); extrorse (2)

337. Androecium, anthers, dehiscence, rimose and rimose but functionally poricidal, slit position, outer antepetalous stamen: latrorse (0); introrse (1); extrorse (2)

338. Androecium, anthers, dehiscence, rimose and rimose but functionally poricidal, slit position, inner antepetalous stamens: latrorse (0); introrse (1); extrorse (2)

339. Androecium, anthers, dehiscence, truly poricidal, pore, number: one (0); two (1)

340. Androecium, anthers, dehiscence, truly poricidal, pore, position: basal (0); apical (1)

341. Androecium, anthers, dehiscence, truly poricidal, pore, ornamentation: absent (0); one-sided wedge-like projection (1); two revolute projections, forming a rim around the pore (2); with a curved apical tube (3)

342. Androecium, anthers, pollen, release, number of grains: monads (0); tetrads (1)

343. Androecium, anthers, pollen, release, raphides: absent (0); with raphides adhering (1)

344. Androecium, anthers, pollen, colour: white to cream or similar to the petals (0); yellow to orange (1)

345. Androecium, anthers, pollen, production, outer antesepalous stamen: no pollen production (staminodial) (0); present (1)

346. Androecium, anthers, pollen, fertility, outer antesepalous stamen: sterile (0); fertile (1)

347. Androecium, anthers, pollen, production, inner antesepalous stamens: no pollen production (staminodial) (0); present (1)

348. Androecium, anthers, pollen, fertility, inner antesepalous stamens: sterile (0); fertile (1)

349. Androecium, anthers, pollen, production, outer antepetalous stamen, pollen production: no pollen production (staminodial) (0); present (1)

350. Androecium, anthers, pollen, fertility, outer antepetalous stamen: sterile (0); fertile (1)

351. Androecium, anthers, pollen, production, inner antepetalous stamens: no pollen production (staminodial) (0); present (1)

352. Androecium, anthers, pollen, fertility, inner antepetalous stamens: sterile (0); fertile (1)

353. Androecium, anthers, pollen, dimorphism: absent (0); in different whorls of polliniferous anthers (1); in the same whorl of polliniferous anthers (2)

354. Androecium, anthers, pollen, aperture, type: inaperturate (0); sulcate (1); porate (2)

355. Androecium, anthers, pollen, aperture, sulcate, number: monosulcate (0); monosulcate with tenuites (1); bisulcate (2)

356. Androecium, anthers, pollen, aperture, porate, number: 2–3-porate (0); 5–7-porate (1)

357. Androecium, anthers, pollen, aperture, transitional zone, ornamentation: elements equal (0); elements reduced (1); elements larger (2); elements closer to each other (3); apertural border (4)
358. Androecium, anthers, pollen, wall, structure: 1-layered (0); 2-layered (1); 3-layered (2)
359. Androecium, anthers, pollen, wall, inner layer, papillate or baculate: absent (0); present (1)
360. Androecium, anthers, pollen, wall, inner surface, striate-rugulate: absent (0); present (1)
361. Androecium, anthers, pollen, wall, hemispheric aperture walls, presence: absent (0); present (1)
362. Androecium, anthers, pollen, wall, hemispheric aperture walls, exine development: devoid of exine (0); flattened with scattered exine elements (1)
363. Androecium, anthers, pollen, infratectum, presence: absent (0); present (1)
364. Androecium, anthers, pollen, tectum, structure: tectate-columellate (0); baculate (1); channelled-tectate (2)
365. Androecium, anthers, pollen, tectum, tectate-columellate, type: tectate-perforate or semitectate (0); eutectate (1)
366. Androecium, anthers, pollen, tectum, tectal elements: acute (0); rounded (1)
367. Androecium, anthers, pollen, tectum, tectal elements, acute, ornamentation, type: spinulose (0); spinulose-rugose (1); tuberculate (2)
368. Androecium, anthers, pollen, tectum, tectal elements, acute, ornamentation, spinulose or spinulose-rugose, spacing: regular (0); irregular (1)
369. Androecium, anthers, pollen, tectum, tectal elements, rounded, ornamentation, type: areolate-rugulate (0); microclavate (1); coarsely areolate (2); rugulose to coarsely rugulose (3); insulate-cerebroid (4); verrucose-granulose (5); verrucate (6); reticulate to foveolate (7); micro verrucate (8); fossulate (9); domed areolate (10); irregularly microclavate (11)
370. Androecium, anthers, pollen, tectum, tectal elements, rounded, ornamentation, homogeneity: homogeneous (0); heterogeneous (1)
371. Androecium, anthers, pollen, tectum, microperforations, presence: absent (0); present (1)
372. Androecium, anthers, pollen, tectum, microperforations, density: sparse (0); dense (1)
373. Androecium, anthers, pollen, sulcal membrane, ornamentation, distinguishability from the tectum: equal (0); slightly different (1); obviously different (2)
374. Androecium, anthers, pollen, sulcal membrane, ornamentation, type: granular (0); coarsely granular-insulate (1); coarsely granular-ridged (2); tuberculate (3); spinulate (4); coarsely spinulate (5); verrucate (6); coarsely verrucate (7)
375. Androecium, staminodes, nectariferous scales, presence: absent (0); present (1)
376. Androecium, staminodes, nectariferous scales, margin: opaque (0); hyaline (1)
377. Androecium, staminodes, filiform staminodes adnate to the lower inner tepals, presence: absent (0); present (1)
378. Androecium, staminodes, antherodes, presence: all staminodes with antherodes (0); medial staminode with an enlarged antherode (1); medial staminode lacking the antherode or antherode much reduced (2); all staminodes lacking antherodes (3)
379. Androecium, staminodes, antherodes, reduced anther sacs, presence: absent (0); present (1)
380. Androecium, staminodes, antherodes, lobes, number: 2-lobed (0); 3-lobed (1); 4-lobed/X-shaped (2)
381. Androecium, staminodes, antherodes, lobes, insertion: sessile (0); stipitate (1)
382. Androecium, staminodes, antherodes, lobes, 2-lobed, shape: scarcely lobed (0); globose to subglobose or obovoid or transversally ellipsoid (1); ellipsoid, antherode V-shaped (2); horseshoe-shaped to curved (3); reniform (4); C-shaped (5)
383. Androecium, staminodes, antherodes, lobes, 3-lobed, shape: cordate (0); clove-shaped (1); sagittate to hastate (2)
384. Androecium, staminodes, antherodes, lobes, 4-lobed/X-shaped, shape: upper lobes larger (0); all lobes equal to subequal (1); lower lobes larger (2)
385. Gynoecium, pistilode, presence in staminate flowers: absent (0); present (1)
386. Gynoecium, pistilode, nectariferous lobes, presence: absent (0); present (1)
387. Gynoecium, pistil, relative length: shorter than the stamens (0); ca. the same length as the stamens (1); 1/2 times longer than the stamens (2); 1–2 times longer than the stamens (3)
388. Gynoecium, epigynial nectaries, presence: absent (0); present (1)

389. Gynoecium, stipe, presence: absent (0); present (1)
390. Gynoecium, ovary, position: inferior (0); superior (1); half-inferior (2); late inferior (3)
391. Gynoecium, ovary/hypanthium (inferior ovary), indumentum, uniseriate, macrohairs: absent (0); eglandular simple (1); glandular (2); hook (3); rugose (4); clavate (5)
392. Gynoecium, ovary/hypanthium (inferior ovary), indumentum, multiseriate, macrohairs: absent (0); tapering (1); dendritic (2); fruticose (3); pilate (4); branched (5)
393. Gynoecium, ovary/hypanthium (inferior ovary), indumentum, distribution: evenly distributed (0); restricted to or concentrated at the apex (1); restricted to septal ridges (2)
394. Gynoecium, ovary, locules, number: one (0); two (1); three (2); posterior locule reduced or aborted (3); pseudomonomerous (4)
395. Gynoecium, ovary, posterior locule, ovules, number: empty (0); one (1); two (2); three (3); four to several (4)
396. Gynoecium, ovary, anterior locules, ovules, number: empty (0); one (1); two (2); three (3); four to several (4)
397. Gynoecium, style, development: absent (0); inconspicuous to very short (1); short to elongate (2)
398. Gynoecium, style, thickness: slender (0); stout (1); flattened (2)
399. Gynoecium, style, base: conic-inflate (0); tapered (1); abrupt-cylindrical (2); abrupt-obconic (3)
400. Gynoecium, style, inflation: not inflated (0); inflated (1)
401. Gynoecium, style, curvature, at anthesis: straight (0); curved (1); descending-falcate (2)
402. Gynoecium, style, curvature, at post-anthesis: straight (0); curved (1); spirally-coiled (2)
403. Gynoecium, style, indumentum, uniseriate, macrohairs: absent (0); eglandular simple (1); glandular (2); clavate (3); branched (4); apically moniliform (5)
404. Gynoecium, style, in fruit: splitting with the valves (0); remaining intact at the columella (1)
405. Gynoecium, stigma, position: terminal (0); oblique (1)
406. Gynoecium, stigma, posture: pointing upwards or straight (0); pointing downwards (1)
407. Gynoecium, stigma, shape: triparted (0); capitate or trilobed to subtrilobed (1); punctate (2); truncate to capitulate (3); penicilliform (4); cup- or funnel-shaped (5); unevenly trilobed (6); trifid (7); tuberculate (8)
408. Gynoecium, stigma, apex, tuff of silky hairs, presence: absent (0); present (1)
409. Gynoecium, stigma, apex, three protuberances, presence: absent (0); present (1)
410. Gynoecium, stigma, sessile, projection: same level as the ovary (0); prominent (1)
411. Fruit, anthocarp, presence: absent (0); present, ovary superior (1); present, ovary inferior (2)
412. Fruit, anthocarp, superior ovary, development: thin (0); thickened to hardened (1)
413. Fruit, anthocarp, superior ovary, coiled, enclosing the fruit: loosely (0); tightly but free from the fruit (1); tightly and fused to the fruit (2)
414. Fruit, anthocarp, superior ovary, longitudinal projections: absent (0); ridges (1); crests (2)
415. Fruit, anthocarp, superior ovary, ornamentation, type: smooth (0); sinuate (1); toothed (2); echinate (3)
416. Fruits, consistency: fleshy (0); dry (1)
417. Fruits, outline shape: ellipsoid to oblongoid (0); oblanceoloid to obovoid or dolabriform (1); subglobose to globose (2); cordate (3); linear-oblongoid to linear to cylindrical (4); trigonous (5); ovoid or lageniform (6); strongly trilobed (7)
418. Fruits, apex: sunken (0); emarginate to truncate or round or acute to slightly apiculate (1); rostrate (2); aristate (3)
419. Fruits, wall, thickness: thin (0); thick and hardened (1)
420. Fruits, wall, ornamentation: smooth (0); tuberculate (1); echinate (2)
421. Fruit, septal ridges, thickening: thin (0); thickened (1)
422. Fruits, overall colour: dull-coloured (0); bright-coloured (1)
423. Fruits, colouration, type: chemical (0); structural (1)
424. Fruits, surface: opaque (0); lustrous (1)
425. Fruits, dehiscence: indehiscent or irregular (0); 2-valved (1); partially 3-valved (2); 3-valved (3); lateral (4); 6-valved (5); denticidal (6); ejaculatory (7)
426. Fruits, dry, type: achene (0); capsule (1); coccarium (2)

427. Fruits, dry, constriction between the seeds: not constricted (0); constricted (1)
428. Fruits, fleshy, colour: white to pale yellow to light green (0); pink to red to orange (1); blue to purple to black (2)
429. Fruits, fleshy, stigma, sessile, projection: same level as the ovary (0); prominent (1)
430. Seeds, dimorphism, presence: absent or all subequal (0); present in the same locule (1); seeds from the dorsal locule different from the ventral locules (2)
431. Seeds, overall shape: longer than wide (0); wider than long or as wide as long (1); ca. as long as wide (2)
432. Seeds, outline, shape: circular (0); elliptic to oblong or rectangular (1); triangular or tetrahedral (2); ovate to kidney-shaped (3); polygonal (4); ovoid or fusiform to barrel-shaped (5); cuboid (6); bottle-shaped (7); irregular and angled (8); deltoid (9)
433. Seeds, outline, compression: absent (0); lateral (1); dorsiventral (2)
434. Seeds, lateral wings, presence: absent (0); present (1)
435. Seeds, lateral wings, distribution: radiate (0); restricted to the margin of the longer axis of the seed (1)
436. Seeds, dorsal side, shape: flattened (0); rounded or acute (1); conical (2)
437. Seeds, ventral side, shape: flattened (0); rounded to acute (1); ridged (2); depressed (3); concave or wedge-shaped (4)
438. Seeds, ventral side, concave or wedge-shaped, projection, shape: absent (0); 1-lobed (1)
439. Seeds, lateral side: uncleft (0); cleft towards the embryotega (1)
440. Seeds, testa, ornamentation, type: smooth (0); reticulate to foveolate (1); scrobiculate to rugose (2); ridged to costate (3); striate (4); longitudinally winged or crested (5); verrucose to tuberculate (6); spirally-striate (7); with dactyliform projections (8); scabrid (9)
441. Seeds, testa, ornamentation, spirally striate, additional ornamentation: smooth (0); tuberculate (1); with dactyliform projections (2)
442. Seeds, testa, ornamentation, longitudinally winged, ornamentation between the longitudinal wings: transversally striate (0); reticulate (1); foveolate (2)
443. Seeds, testa, ornamentation, with dactyliform projections, distribution: evenly distributed (0); restricted to the margin (1); arranged in longitudinal striae with short dactyliform projections (2)
444. Seeds, testa, deposition: absent (0); farinose (1); sticky/mucilaginous (2); coat of fused farinose granules (3)
445. Seeds, chalazal cap, development: reduced (0); enlarged (1)
446. Seeds, embryotega, position: dorsal (0); semidorsal to semilateral to lateral (1); apical (2)
447. Seeds, embryotega, development: inconspicuous (0); prominent (1); prominent but obscured by a deep depression (2)
448. Seeds, embryotega, micropillar scar: absent (0); present (1)
449. Seeds, embryotega, colour, relative to the remaining testa: evenly coloured (0); distinctively lighter than the testa or white (1)
450. Seeds, accessory tissues, presence: none (0); aril (1); lateral appendage (2)
451. Seeds, accessory tissues, aril, colouration: hyaline (0); white (1); orange to red (2); tan coloured and papery (3)
452. Seeds, accessory tissues, aril, development: covering only its seed (0); expanded fused to the neighbouring seed's aril, forming a sole dispersal unit per locule (1); obscuring the hilum (3)
453. Seeds, hilum, shape: punctate to elliptic (0); linear (1); C-shaped (2)
454. Seeds, hilum, length, relative to 1/2 of the seed's total length: shorter (0); equal (1); slightly longer (2); ca. the same length as the seed (3)
455. Seedlings, primary root, branching: unbranched (0); freely-branching (1)
456. Seedlings, primary root, shape: straight (0); turnip-shaped (1); sinuate to spirally-coiled (2)
457. Seedlings, primary root, colouration: white (0); brown (1); yellow or orange or red or violet (2)
458. Seedlings, rhizoids, presence: absent (0); present (1)
459. Seedlings, collar, presence: absent (0); present (1)
460. Seedlings, collar, morphology: inconspicuous (0); thick ring (1); umbrella-like (2)
461. Seedlings, mesocotyl, presence: absent (0); present (1)
462. Seedlings, cotyledon, chlorophyll production: chlorophyllate (0); non-chlorophyllate (1)

463. Seedlings, cotyledon, function: haustorial (0); assimilating (1)
464. Seedlings, cotyledon, sheath, development: inconspicuous (0); conspicuous (1)
465. Seedlings, cotyledon, sheath, projection: absent (0); coleoptile (1); lobes (2); ligule (3)
466. Seedlings, cotyledon, hyperphyll, morphology: cylindrical (0); bifacial (1); leaf-like with an apical ventral cone (2)
467. Seedlings, cotyledon, middle part, presence: absent (0); short (1) long (2)
468. Seedlings, primary leaves, development: all blades expanded (0); at least the first modified into a cataphyll (1)
469. Seedlings, primary leaves, type: unifacial (0); bifacial leaf-like (1); bifacial ribbon-like (2)
470. Anatomy, silica bodies, in the leaves, presence: absent (0); present (1)
471. Anatomy, bean-shaped starch grains, presence: absent (0); present (1)
472. Anatomy, vessels, distribution: restricted to the roots (0); roots and stems (1)
473. Anatomy, roots, mucilage canals, presence: absent (0); present (1)
474. Anatomy, roots, pith, sclerification: non-sclerified (0); sclerified (1)
475. Anatomy, stems, nodal vascular plexus, presence: absent (0); present (1)
476. Anatomy, stems, cortex, morphology: narrow and lacking vascular tissue (0); expanded and with vascular tissue (1)
477. Anatomy, stems, vascular bundles, fibrous layer, presence: absent (0); present (1)
478. Anatomy, stems, vascular bundles, fibrous layer, development: partially enclosing the bundles (0); completely enclosing the bundles (1)
479. Anatomy, hairs, uniseriate, glandular microhairs, presence: absent (0); present (1)
480. Anatomy, hairs, uniseriate, glandular microhairs, basal cell, morphology: lenticular and not wedged between epidermal cells (0); lenticular and wedged (1); with a short neck (2); with a conspicuous neck (3)
481. Anatomy, hairs, uniseriate, glandular microhairs, medial cell, morphology: cylindrical and narrower than the distal (0); cylindrical and as wide as the distal (1); cylindrical, wider and with thicker wall than the distal (2); barrel-shaped (3); ellipsoid (4); fusiform (5); clavate (6)
482. as wide as the middle cell (1); wider than the middle cell (2)
483. Anatomy, hairs, uniseriate, macrohairs, type: absent (0); exclusively simple uniseriate or glandular (1); flagelliform (2); hook (3); clavate (4); rugose (5)
484. Anatomy, hairs, uniseriate, macrohairs, moniliform, presence: absent (0); present (1)
485. Anatomy, hairs, uniseriate, macrohairs, branched (Palisota type), presence: absent (0); present (1)
486. Anatomy, hairs, uniseriate, macrohairs, star-shaped idioblasts, presence: absent (0); present (1)
487. Anatomy, hairs, uniseriate, macrohairs, star-shaped idioblasts, arm, morphology: long with rounded apex (0); short with acute apex (1)
488. Anatomy, hairs, uniseriate, macrohairs, papillae: absent (0); present (1)
489. Anatomy, hairs, uniseriate, macrohairs, prickle-hairs, basal cell: not swollen (0); swollen (1)
490. Anatomy, hairs, uniseriate, macrohairs, falcate-hairs: absent (0); present (1)
491. Anatomy, hairs, multiseriate, tapering, presence: absent (0); present (1)
492. Anatomy, hairs, multiseriate, branched (Conostylidoideae type), presence: absent (0); present (1)
493. Anatomy, hairs, multiseriate, fruticose (Hanguana type), presence: absent (0); present (1)
494. Anatomy, hairs, multiseriate, dendritic, presence: absent (0); present (1)
495. Anatomy, hairs, multiseriate, pilate, presence: absent (0); present (1)
496. Anatomy, hairs, multiseriate, basal cell-rosette, presence: absent (0); present (1)
497. Anatomy, stem, cuticle, ornamentation: smooth (0); ribbed (1)
498. Anatomy, leaf epidermis, cuticle, ornamentation: smooth (0); striated to ridged (1)
499. Anatomy, leaf epidermis, surface, cell, morphology: not-domed (0); domed (1)
500. Anatomy, leaf epidermis, warts, presence: absent (0); present (1)
501. Anatomy, leaf epidermis, warts, cell wall thickened: absent (0); present (1)
502. Anatomy, leaf epidermis, warts, arrangement: non-continuous (0); continuous resembling striate thickenings (1)
503. Anatomy, leaf epidermis, cell layer, number: one (0); two or more (1); two layers at margin (2)

504. Anatomy, leaf epidermis, cell wall, thickness: thin (0); evenly thick (1); lenticular thickened (2)
505. Anatomy, leaf epidermis, cell wall, morphology: absent (0); paradermic invaginations (1); transversely lobed (2)
506. Anatomy, leaf epidermis, stomata, neighbouring cells, division: oblique (0); parallel (1)
507. Anatomy, leaf epidermis, stomata, neighbouring cells, number: 2-celled (0); 4-celled (1); 6-celled, terminal cells small (2); 6-celled, terminal cells large (3)
508. Anatomy, leaf epidermis, stomata, neighbouring cells, terminal cells, size in comparison to the lateral cells: smaller (0); equal (1); larger (2)
509. Anatomy, leaf epidermis, silica crystals, in specialised cells, presence: absent (0); present (1)
510. Anatomy, leaf epidermis, silica crystals, in specialised cells, cell walls: thin (0); thickened (1)
511. Anatomy, leaf epidermis, silica crystals, in specialised cells, cell morphology: enlarged (0); deep and wedged between regular epidermal cells (1); arranged in longitudinal bands or solitary and wedged, but at the same level as regular epidermal cells (2)
512. Anatomy, leaf epidermis, silica crystals, in specialised cells, silica type 2, presence: absent (0); present (1)
513. Anatomy, leaf epidermis, bulliform cells, presence: absent (0); present (1)
514. Anatomy, leaf, vascular bundles, organisation: xylem abaxial, phloem adaxial (0); xylem and phloem alternate or circular phloem with central xylem or xylem abaxial (1); xylem abaxial and phloem adaxial near the margin of the blades plus xylem and phloem alternate near the centre of the blade (2)
515. Anatomy, leaf, vascular bundles, contact with the epidermis: absent (0); present (1)
516. Anatomy, leaf, mesophyll, palisade cells, shape: unlobed (0); elaborately lobed (1)
517. Anatomy, leaf, mesophyll, calcium oxalate raphides, presence: absent (0); present (1)
518. Anatomy, leaf, mesophyll, calcium oxalate raphides, inside raphid canals, presence: absent (0); present (1)
519. Anatomy, leaf, mesophyll, calcium oxalate raphides, inside raphid canals, distribution: evenly distributed throughout the blades (0); restricted to along the veins of the blades (1)
520. Anatomy, perianth, styloid crystals, presence: absent (0); present (1)
521. Anatomy, leaf, mesophyll, aerenchyma, presence: absent (0); present (1)
522. Anatomy, leaf, mesophyll, tannin cells, presence: absent (0); present (1)
523. Anatomy, leaf, mesophyll, mucilage cells, presence: absent (0); present (1)
524. Anatomy, leaf, mesophyll, longitudinal bundles: diffuse (0); with fibrous extensions (1)
525. Anatomy, leaf, mesophyll, marginal mechanical tissue, presence: absent (0); present (1)
526. Anatomy, leaf, mesophyll, marginal fibre caps, presence: absent (0); present (1)
527. Anatomy, flower, receptacle, aerenchyma, presence: absent (0); present (1)
528. Anatomy, flower, receptacle, aerenchyma, concentration: sparse (0); dense (1)
529. Anatomy, flower, receptacle, tannin cells: absent (0); present (1)
530. Anatomy, flower, perianth, tube, epidermal unicellular gland, presence: absent (0); present (1)
531. Anatomy, flower, perianth, tannin cells: absent (0); present (1)
532. Anatomy, flower, perianth, tannin cells, homogeneous, presence: absent (0); present (1)
533. Anatomy, flower, perianth, tannin cells, granular, presence: absent (0); present (1)
534. Anatomy, flower, perianth, tannin cells, fibrillar, presence: absent (0); present (1)
535. Anatomy, flower, perianth, tannin cells, distribution: sparse (0); moderate (1); abundant (2)
536. Anatomy, flower, perianth, aerenchyma, presence: absent (0); present (1)
537. Anatomy, flower, perianth, aerenchyma, density: sparse (0); dense (1)
538. Anatomy, flower, perianth, inner whorl/petals, cuticular surface, deposition, type: mucilage (0); epicuticular layer of wax (1)
539. Anatomy, flower, perianth, inner whorl/petals, cuticular surface, deposition, ornamentation: smooth (0); striate (1)
540. Anatomy, flower, androecium, stamens primordia, ontogeny, origin: same as the petals (0); distinct (1)
541. Anatomy, flower, androecium, anthers, wall, at maturity: 2-layered (0); more than 2 layers (1)

542. Anatomy, flower, androecium, anthers, endothecium, type: not thickened (0); basally thickened (1); spirally thickened (2); medially thickened (3)
543. Anatomy, flower, androecium, anthers, tapetum, type: glandular (0); amoeboid (1); invasive non-syncytial (2)
544. Anatomy, flower, androecium, anthers, tapetum, raphides: absent (0); present (1)
545. Anatomy, flower, gynoecium, ovary, wall, silica crystals: absent (0); present (1)
546. Anatomy, flower, gynoecium, ovary, wall, tannin cells: absent (0); present (1)
547. Anatomy, flower, gynoecium, ovary, wall, aerenchyma: absent (0); present (1)
548. Anatomy, flower, gynoecium, ovary, septae, conation: aposeptalous (0); hemiseptalous (1); synseptalous (2)
549. Anatomy, flower, gynoecium, ovary, septae, septal commissures: absent (0); present throughout (1); apical (2)
550. Anatomy, flower, gynoecium, ovary, septae, epithelial cells: absent (0); present (1)
551. Anatomy, flower, gynoecium, ovary, septae, epithelial cells, density: sparse (0); medium to dense (1)
552. Anatomy, flower, gynoecium, ovary, septae, tannin cells: absent (0); present (1)
553. Anatomy, flower, gynoecium, ovary, locules, mucilage-secreting colleter hairs, presence: absent (0); present (1)
554. Anatomy, flower, gynoecium, ovary, placenta, ovules, arrangement: uniseriate (0); partially biseriate to biseriate (1); multiseriate (2)
555. Anatomy, flower, gynoecium, ovary, placenta, position: axile (0); pendule (1); intrusive-parietal (2); axile-parietal (3); basal (4)
556. Anatomy, flower, gynoecium, ovary, placenta, shape: linear (0); clavate (1); pendulous-peltate or inclinate-peltate (2); straight-peltate (3); hemispheric (4); globose (5); blanket-like (6); ribbon-like (7)
557. Anatomy, flower, gynoecium, ovary, placenta, flanges: unflanged (0); slightly 2-flanged (1); 2-flanged (2)
558. Anatomy, flower, gynoecium, ovary, placenta, sclereids: absent (0); present (1)
559. Anatomy, flower, gynoecium, ovary, placenta, tannin cells: absent (0); present (1)
560. Anatomy, flower, gynoecium, ovary, ovule, orientation: orthotropous (0); hemianatropous to anatropous (1); campylotropous (2)
561. Anatomy, flower, gynoecium, ovary, ovule, non-orthotropous, position: epitropous (0); hypotropous (1); pleurotropous (2)
562. Anatomy, flower, gynoecium, style, stylar canal, presence: absent (0); present (1)
563. Anatomy, flower, gynoecium, style, tannin cells: absent (0); present (1)
564. Anatomy, flower, gynoecium, stigma, surface: dry (0); wet (1)
565. Anatomy, flower, gynoecium, stigma, papillae, type: A (0); B or B+E (1); C (2); D (3); E (4); F (5); G (6); C+E (7)
566. Anatomy, flower, gynoecium, stigma, papillae, epidermis, folded, presence: absent (0); present (1)
567. Anatomy, flower, gynoecium, stigma, papillae, cell number: unicellular (0); all multicellular (1); only the marginal multicellular (2); the marginal moniliform (3)
568. Anatomy, flower, gynoecium, stigma, papillae, distribution: evenly distributed across (0); restricted to the margin (1)
569. Anatomy, flower, gynoecium, stigma, papillae, length: longer than 1μm (0); equal or shorter than 1μm (1); equal or shorter than 0.5μm (2)
570. Anatomy, seed, coat, type: testal (0); bitegmic (1)
571. Anatomy, seed, coat, outer tegmen: thick and persistent (0); thin and sloughing off (1)
572. Anatomy, seed, coat, sclerified, presence: absent (0); present (1)
573. Anatomy, seed, coat, crystals, presence: absent (0); silica (1); calcium oxalate (2)
574. Anatomy, seed, coat, two layers of crossing fibres, presence: absent (0); present (1)
575. Anatomy, seed, embryo, type: Trillium (0); Xyris-Scirpus or grass (1)
576. Anatomy, seed, embryo, relative size: shorter than 1/2 the length of the seed (0); ca. 1/2 the length of the seed (1); ca. as long as the seed (2)
577. Anatomy, seed, endosperm, type: nuclear (0); helobial (1); helobial-chalazal (2)
578. Anatomy, seed, endosperm, concentration/amount: scanty (0); copious (1)

579. Anatomy, seed, nucellus, parietal layer: crassinucellate (0); tenuinucellate (1)
580. Chromosomes, number, up to 19: n= 4–5 (0); n= 6 (1); n=7 (2); n= 8 (3); n= 9 (4); n= 10 (5); n= 11 (6); n= 14, 15 or 16 (7); n= 17 (8); n= 19 (9)
581. Chromosomes, number, above 19: n=26 (0); n= 29 (1); n= ca. 85 (2); n= variable due to Robertsonian Translocations (3)
582. Chromosomes, size: ≤ 2μm (0); ca. 3μm (1); > 3μm ≤ 5μm (2); > 5μm < 10μm (3); ≥ 10μm (4)
583. Chromosomes, karyotype, homogeneity: unimodal (0); bimodal (1)
584. Chromosomes, complements, symmetry: symmetric (0); at least some asymmetric (1)
585. Phytochemistry, pro-anthocyanins, presence: absent (0); present (1)
586. Phytochemistry, anthocyanin, type: acylated cyanidin 3,7,3′-triglycoside (0); commelinin (1); 3-glycoside (2); eichhornin (3); delphinidin (4)
587. Phytochemistry, acids, chelidonic acid, presence: absent (0); present (1)
588. Phytochemistry, acids, presence: absent (0); diferulic acids (1); syringic acids (2)
589. Phytochemistry, cyanogenic compounds, presence: absent (0); present (1)
590. Phytochemistry, phenylphenalenones, presence: absent (0); present (1)
591. Phytochemistry, C-glycosides, presence: absent (0); present (1)
592. Phytochemistry, flavanols, presence: absent (0); present (1)
593. Phytochemistry, flavonoids, apigenin, presence: absent (0); present (1)
594. Phytochemistry, flavonoids, luteolin, presence: absent (0); present (1)
595. Phytochemistry, flavonoids, hidroxiluteolin, presence: absent (0); present (1)
596. Phytochemistry, flavonoids, isorhamnetin, presence: absent (0); present (1)
597. Phytochemistry, flavonoids, quercetin, presence: absent (0); present (1)
598. Phytochemistry, flavones, 6-hidroxiluteolin, presence: absent (0); present (1)
599. Phytochemistry, phenolic and sulphate derivatives, presence: absent (0); present (1)
600. Phytochemistry, p-coumaric acids, presence: absent (0); present (1)

Supplementary Table S3

Supplementary Table S3. Morphological matrix for Commelinales (separate .xlsv file).

Matrix_Commelinales_410x600

Supplementary Table S4

Supplementary Table S4. Historical ordinal placement of the families previously placed in Commelinales and currently placed elsewhere.

Classification	Bromeliaceae	Dasypogonaceae	Eriocaulaceae	Flagellariaceae	Mayacaceae	Rapateaceae	Restionaceae	Xyridaceae
Berchtold & Presl (1820)	Alliales	Juncales[1]	Restionales[2]	—	Commelinales[3]	—	Restionales	Restionales[2]
Reichenbach (1828)	Caulo-Acroblastae	Phyllo-Acroblastae[4]	Caulo-Acroblastae[3]	Caulo-Acroblastae[3]	Caulo-Acroblastae[3]	Caulo-Acroblastae[3]	Caulo-Acroblastae[3]	Caulo-Acroblastae[3]
Dumortier (1829)	Bromeliales	**Commelinales**	**Commelinales**	Liliales	**Commelinales**[3]	Juncales	Juncales	**Commelinales**
Endlicher (1836)	Ensatae	Coronariae[1]	**Enantioblastae**[†]	Coronariae[1]	**Enantioblastae**[5,†]	Coronariae[1]	**Enantioblastae**[†]	**Enantioblastae**[†]
Eichler (1890)	Ensatae	Coronariae[1]	**Enantioblastae**[†]	Coronariae[1]	**Enantioblastae**[5,†]	Coronariae[1]	**Enantioblastae**[†]	**Enantioblastae**[†]
Lindley (1846)	Narcissales	Xyridales[5]	Glumales	Xyridales[3]	Xyridales	Part in Xyridales[5],	Glumales	Xyridales

						part in Juncales[1]		
Engler (1886)	**Farinosae**†	Liliiflorae[6]	**Farinosae**†	**Farinosae**†	**Farinosae**†	**Farinosae**†	**Farinosae**†	**Farinosae**†
Rendle (1904)	**Farinosae**†	Liliiflorae[6]	**Farinosae**†	**Farinosae**†	**Farinosae**†	**Farinosae**†	**Farinosae**†	**Farinosae**†
Hutchinson (1934)	Bromeliales	Agavales[7]	Eriocaulales*	**Commelinales**	**Commelinales**	Xyridales	Juncales	Xyridales
Cronquist (1968)	Bromeliales	Liliales[7]	Eriocaulales*	Restionales	**Commelinales**	**Commelinales**	Restionales	**Commelinales**
Thorne (1968)	**Commelinales**	Liliales[6]	**Commelinales**	**Commelinales**	**Commelinales**	**Commelinales**	**Commelinales**	**Commelinales**
Takhtajan (1969)	Bromeliales	Liliales[6]	Eriocaulales*	Restionales	**Commelinales**	**Commelinales**	Restionales	**Commelinales**
Thorne (1976)	**Commelinales**	Liliales[6]	**Commelinales**	**Commelinales**	**Commelinales**	**Commelinales**	**Commelinales**	**Commelinales**
Dahlgren (1980)	Bromeliales	Asparagales	Eriocaulales	Poales	**Commelinales**	Eriocaulales	Poales	Eriocaulales
Takhtajan (1980)	Bromeliales	Liliales[7]	Eriocaulales*	Restionales	**Commelinales**	**Commelinales**	Restionales	**Commelinales**
Cronquist (1981)	Bromeliales	Liliales[7]	Eriocaulales*	Restionales	**Commelinales**	**Commelinales**	Restionales	**Commelinales**
Dahlgren & Clifford (1982)	Bromeliales	Asparagales	Eriocaulales	Poales	**Commelinales**	Eriocaulales	Poales	Eriocaulales
Dahlgren & Rasmussen (1983)	Bromeliales	Asparagales	Eriocaulales	Poales	**Commelinales**	Eriocaulales	Poales	Eriocaulales
Dahlgren *et al.* (1985)	Bromeliales	Asparagales	**Commelinales**	Poales	**Commelinales**	**Commelinales**	Poales	**Commelinales**
G. Dahlgren (1989)	Bromeliales	Asparagales	**Commelinales**	Poales	**Commelinales**	**Commelinales**	Poales	**Commelinales**
Goldberg (1989)	Bromeliales	Liliales[7]	**Commelinales**	Juncales	**Commelinales**	**Commelinales**	Juncales	**Commelinales**
Thorne (1992a)	Bromeliales	Asparagales	**Commelinales**	Poales	**Commelinales**	**Commelinales**	Poales	**Commelinales**
Thorne (1992b)	Bromeliales	Liliales	**Commelinales**	Poales	**Commelinales**	**Commelinales**	Poales	**Commelinales**
Takhtajan (1997)	Bromeliales	Xanthorrhoeales	Eriocaulales*	Flagellariales*	Mayacales*	Rapateales*	Restionales	Xyridales
Kubitzki (1998)	Bromeliales	Dasypogonales	Xyridales	Poales	Xyridales	Xyridales	Poales	Xyridales
APG I (1998)	Poales	—	Poales	Poales	Poales	Poales	Poales	Poales
APG II (2003)	Poales	—	Poales	Poales	Poales	Poales	Poales	Poales
Thorne & Reveal (2007)	Bromeliales	Dasypogonales*	Xyridales	Poales	Xyridales	Bromeliales	Restionales	Xyridales
APG III (2009)	Poales	—	Poales	Poales	Poales	Poales	Poales	Poales

Takhtajan (2009)	Brome-liales	Dasy-pogonales*	Xyridales	Restion-ales	Xyridales	Xyridales	Restion-ales	Xyridales
APG IV (2016)	Poales	Arecales	Poales	Poales	Poales	Poales	Poales	Poales
Givnish *et al.* (2018)	Poales	Dasy-pogonales*	Poales	Poales	Poales	Poales	Poales	Poales
Shipunov (2022)	Poales	Arecales	Poales	Poales	Poales	Poales	Poales	Poales

[1]in Juncaceae, [2]in Restionaceae, [3]in Commelinaceae, [4]in Sarmentaceae (i.e., Asparagaceae), [5]in Xyridaceae, [6]in Liliaceae, [7]in Xanthorrhoeaceae, *single family order, †homotypic synonym of Commelinales, — unplaced.

Supplementary Table S5

Supplementary Table S5. Historical ordinal placement of families currently accepted in Commelinales.

Classification	Comme-linaceae	Haemo-doraceae	Hanguanaceae	Philydraceae	Pontederia-ceae
Berchtold & Presl (1820)	**Commelinales**	Alliales	—	—	—
Reichenbach (1828)	Caulo-Acroblas-tae	Part in Caulo-Acroblastae[1], part in Phyllo-Acroblas-tae[2]	—	Caulo-Acroblas-tae[1]	Caulo-Acroblas-tae[1]
Dumortier (1829)	**Commelinales**	Part in Narcis-sales[3], part in Liliales[4]	—	Philydrales*	Liliales
Endlicher (1836)	**Enantioblastae†**	Ensatae	Coronariae[5]	Coronariae	Coronariae
Eichler (1890)	**Enantioblastae†**	Ensatae	Coronariae[5]	Coronariae	Coronariae
Lindley (1846)	**Xyridales**	Narcissales	Juncales[5]	Xyridales	Liliales
Engler (1886)	**Farinosae†**	Liliiflorae	—	Farinosae	Farinosae
Rendle (1904)	**Farinosae†**	Liliiflorae	—	Farinosae	Farinosae
Hutchinson (1934)	**Commelinales**	Haemodorales	**Commelinales[6]**	Haemodorales	Liliales
Cronquist (1968)	**Commelinales**	Liliales	Liliales[7]	Liliales	Liliales
Thorne (1968)	**Commelinales**	Liliales[7]	Liliales[7]	**Commelinales**	**Commelinales**
Takhtajan (1969)	**Commelinales**	Liliales	Restionales	Liliales	Liliales
Thorne (1976)	**Commelinales**	Liliales[7]	Liliales[7]	**Commelinales**	**Commelinales**
Dahlgren (1980)	**Commelinales**	Haemodorales*	Asparagales	Philydrales*	Pontederiales
Cronquist (1981)	**Commelinales**	Liliales	Liliales	Liliales	Liliales
Takhtajan (1980)	**Commelinales**	Liliales	Liliales	Liliales	Liliales
Dahlgren & Clifford (1982)	**Commelinales**	Haemodorales*	Asparagales	Philydrales*	Pontederiales*
Dahlgren & Ras-mussen (1983)	**Commelinales**	Haemodorales*	Asparagales or Poales	Philydrales*	Pontederiales*
Dahlgren *et al.* (1985)	**Commelinales**	Haemodorales*	Asparagales	Philydrales*	Pontederiales*
G. Dahlgren (1989)	**Commelinales**	Haemodorales*	Hanguanales	Philydrales*	Pontederiales*
Goldberg (1989)	**Commelinales**	Iridales	Liliales[7]	Iridales	Liliales
Thorne (1992a)	**Commelinales**	Philydrales	Asparagales	Philydrales	Philydrales
Thorne (1992b)	**Commelinales**	Bromeliales	Liliales	Bromeliales	Bromeliales
Takhtajan (1997)	**Commelinales**	Haemodorales*	Hanguanales*	Philydrales*	Pontederiales*
Kubitzki (1998)	**Commelinales**	**Commelinales**	—	**Commelinales**	**Commelinales**

APG I (1998)	**Commelinales**	**Commelinales**	—	**Commelinales**	**Commelinales**
APG II (2003)	**Commelinales**	**Commelinales**	—	**Commelinales**	**Commelinales**
Thorne & Reveal (2007)	**Commelinales**	**Commelinales**	**Commelinales**	**Commelinales**	**Commelinales**
APG III (2009)	**Commelinales**	**Commelinales**	**Commelinales**	**Commelinales**	**Commelinales**
Takhtajan (2009)	**Commelinales**	**Commelinales**	**Commelinales**	**Commelinales**	**Commelinales**
APG IV (2016)	**Commelinales**	**Commelinales**	**Commelinales**	**Commelinales**	**Commelinales**
Givnish *et al.* (2018)	**Commelinales**	**Commelinales**	**Commelinales**	**Commelinales**	**Commelinales**
Shipunov (2022)	Zingiberales	Zingiberales	Zingiberales	Zingiberales	Zingiberales

[1]in Commelinaceae, [2]in Narcissaceae (i.e. Amaryllidaceae), [3]in Agavaceae, [4]as Xiphidiaceae, [5]in Juncaceae, [6]in Flagellariaceae, [7]in Liliaceae, *single family order, †homotypic synonym of Commelinales, — unplaced.

Supplementary Lists

A complete and detailed linear arrangement of the proposed infra-ordinal classification for Commelinales. The classification covers all names from order to genus, presented in a combination of alphabetical and phylogenetic arrangement.

Supplementary List S1

Supplementary List S1. Linear arrangement for the proposed infra-ordinal classification for Commelinales.

Commelinales Mirb. ex Bercht. & J.Presl, Přir. Rostlin: 267. 1820, as "*Commelinariae*"; Commelinales Dumort., Anal. Fam. PI.: 54. 1829, isonym. Type family. Commelinaceae Mirb., nom. cons. (*Commelina* Plum. ex L.).

= Enantioblastae Mart. ex Endl., Gen. Pl.: 119. 1836, **syn. nov.** Type family (designated here). Commelinaceae Mirb., nom. cons. (*Commelina* Plum. ex L.).

= Farinosae Engl., Bot. Gart. Breslau: 23: 1886, **syn. nov.** Type family (designated here). Commelinaceae Mirb., nom. cons. (*Commelina* Plum. ex L.).

= Haemodorales Mart., Consp. Regn. Veg.: 9. 1835. Type family. Haemodoraceae R.Br., nom. cons. (*Haemodorum* Sm.).

= Hanguanales R.Dahlgren ex Reveal, Novon 2: 239. 1992. Type family. Hanguanaceae Airy Shaw (*Hanguana* Blume).

= Philydrales Dumort., Anal. Fam. Pl.: 62. 1829. Type family. Philydraceae Link, nom. cons. (*Philydrum* Banks & Sol. ex Gaertn.).

= Pontederiales Mart., Consp. Regn. Veg.: 7. 1835. Type family. Pontederiaceae Kunth, nom. cons. (*Pontederia* L.).

I. Commelinineae Engl., Syllabus, ed. 2: 87. Syllabus, ed. 2: 87. 1898. Type family. Commelinaceae Mirb., nom. cons. (*Commelina* Plum. ex L.).

I.A. Commelinaceae Mirb., Hist. Nat. Pl. 8: 177. 1804, nom. cons. Type genus. *Commelina* Plum. ex L.

= Cartonemataceae Pichon, Notul. Syst. (Paris) 12: 219. 1946, nom. cons. Type genus. *Cartonema* R.Br.

= Ephemeraceae Batsch, Tab. Affin. Regni Veg.: 125. 1802, as "*Ephemera*", nom. rej., non Ephemeraceae Hampe, Flora 20: 285. 1837, nom. cons., Bryopsida. Type genus. *Ephemerum* Mill. (= *Tradescantia* Ruppius ex L. emend. M.Pell.).

= Tradescantiaceae Salisb., Trans. Linn. Soc. London 8: 9. 1834, as "*Tradescanteae*". Type genus. *Tradescantia* Ruppius ex L. emend. M.Pell.

I.A.1. Cartonematoideae Faden ex G.C.Tucker, J. Arnold Arbor. 70: 99. 1989; Cartonematoideae Faden ex Faden & D.R.Hunt, Taxon 40: 22. 1991, isonym. Type genus. *Cartonema* R.Br.

= Triceratelloideae Zuntini & Frankel, Amer. J. Bot. 108(7): 1086. 2021, **syn. nov.** Type genus. *Triceratella* Brenan.

I.A.1.i. Cartonemateae Faden & D.R.Hunt, Taxon 40: 23. 1991. Type genus. *Cartonema* R.Br.

I.A.1.i.a. *Cartonema* R.Br., Prodr.: 271. 1810. Type species. *Cartonema spicatum* R.Br.

I.A.1.ii. Triceratelleae Faden & D.R.Hunt, Taxon 40: 23. 1991. Type genus. *Triceratella* Brenan.

I.A.1.ii.a. *Triceratella* Brenan, Kirkia 1: 14. 1961. Type species. *Triceratella drummondii* Brenan.

I.A.2. Commelinoideae Eaton, Bot. Dict., ed. 4: 27. 1836, as "*Commelineae*". Type genus. *Commelina* Plum. ex L.

= Tradescantioideae G.Brückn., Bot. Jahrb. Syst. 61(Beibl. 137): 56. 1927, as "*Tradescantieae*". Type genus. *Tradescantia* Ruppius ex L. emend. M.Pell.

I.A.2.i. Palisoteae M.Pell. & Faden ex Zuntini & Frankel, Amer. J. Bot. 108(7): 1086. 2021; Palisoteae M.Pell. & Faden ex C.K.Lee, Fuse & M.N.Tamura, Bot. J. Linn. Soc. 198(2): 127. 2021, isonym. Type genus. *Palisota* Rchb. ex Endl., nom. cons.

= Palisotinae Faden & D.R.Hunt, Taxon 40: 23. 1991. Type genus. *Palisota* Rchb. ex Endl., nom. cons.

I.A.2.i.a. *Palisota* Rchb. ex Endl., Gen. Pl.: 125. 1836, nom. cons. Type species. *Palisota ambigua* (P.Beauv.) C.B.Clarke.

= *Duchekia* Kostel., Allg. Med.-Pharm. Fl. 1: 213. 1831. Type species. *Duchekia hirsuta* (Thunb.) Kostel. [≡ *Palisota hirsuta* (Thunb.) K.Schum.].

I.A.2.ii. Commelineae Dumort., Anal. Fam. Pl.: 55. 1829. Type genus. *Commelina* Plum. ex L.

= Pollieae C.B.Clarke, J. Bot. 18: 127. 1880. Type genus. *Pollia* Thunb.

= Anthericopsideae Pichon, Notul. Syst. (Paris) 12: 237. 1946. Type genus. *Anthericopsis* Engl. (= *Murdannia* Royle, nom. cons.).

= Pseudoparideae Pichon, Notul. Syst. (Paris) 12: 240. 1946. Type genus. *Pseudoparis* H.Perrier.

I.A.2.ii.a. Commelininae M.Pell. & Faden ex C.K.Lee, Fuse & M.N.Tamura, Bot. J. Linn. Soc. 198(2): 127. 2021. Type genus. *Commelina* Plum. ex L.

I.A.2.ii.a.(1). *Dictyospermum* Wight, Icon. Pl. Ind. Orient. 6: 29. 1853. Types species. *Dictyospermum montanum* Wight.

I.A.2.ii.a.(2). *Pollia* Thunb., Nov. Gen. Pl. 1: 11. 1781. Type species. *Pollia japonica* Thunb.

≡ *Dirtea* Raf., Fl. Tellur. 3: 69. 1836[1837], nom. superfl., **syn. nov.** Type species. *Dirtea japonica* (Thunb.) Raf. (≡ *Pollia japonica* Thunb.).

= *Aclisia* E.Mey. ex C.Presl, Reliq. Haenk. 1: 137. 1827. Type species. *Aclisia sorzogonensis* E.Mey. ex C.Presl. [= *Pollia secundiflora* (Blume) Bakh. f.].

= *Lamprocarpus* Blume ex Schult. & Schult. f., Syst. Veg. (ed. 15 bis) 7(2): 1615, 1726. 1830. Type species. *Lamprocarpus thyrsiflorus* (Blume) Blume ex Schult. & Schult. f. [≡ *Pollia thyrsiflora* (Blume) Steud.].

I.A.2.ii.a.(3). *Fadeniella* M.Pell., Plants [Basel] XXX(XXX)-XXX: 47. 2026. Type species. *Fadeniella brasiliensis* (C.B.Clarke) M.Pell. (≡ *Aneilema brasiliense* C.B.Clarke).

I.A.2.ii.a.(4). *Polyspatha* Benth., Niger Fl.: 543. 1849. Type species. *Polyspatha paniculata* Benth.

I.A.2.ii.a.(5). *Aneilema* R.Br., Prodr.: 270. 1810. Type species. *Aneilema biflorum* R.Br.

= *Amelina* C.B.Clarke, Commelyn. Cyrtandr. Bengal: 38. 1874. Type species. *Amelina wallichii* C.B.Clarke [= *Aneilema aequinoctiale* (P.Beauv.) Loudon].

= *Bauschia* Seub. ex Warm., Vidensk. Meddel. Naturhist. Foren. Kjøbenhavn 1872: 123. 1872. Type species. *Bauschia bracteolata* (Mart.) Seub. ex Warm. (≡ *Aneilema bracteolatum* Mart.).

= *Lamprodithyros* Hassk., Flora 46: 388. 1863. Type species. *Lamprodithyros petersii* Hassk. [≡ *Aneilema petersii* (Hassk.) C.B.Clarke].

= *Piletocarpus* Hassk., Flora 49: 212. 1866. Type species. *Piletocarpus protensus* (Wall. ex Wight) Hassk. [= *Aneilema protensum* (Wall. ex Wight) Thwaites].

= *Rhopalephora* Hassk., Bot. Zeitung (Berlin) 22: 58. 1864a. Type species. *Rhopalephora blumei* Hassk., nom. illeg. [≡ *Aneilema micranthum* (Vahl) Kunth].

– *Perosanthera* Fend, Sitzungsb. Akad. Wien. 50: 353. 1864, nom. nud.

I.A.2.ii.a.(6). *Commelina* Plum. ex L., Sp. Pl. 1: 40. 1753. Type species. *Commelina communis* L.

= *Allosperma* Raf., Fl. Tellur. 4: 122. 1836[1838]. Type species. *Allosperma tuberosa* (L.) Raf. (≡ *Commelina tuberosa* L.).

= *Allotria* Raf., Fl. Tellur. 3: 70. 1836[1837]. Type species. *Allotria scabra* Raf. (= *Commelina virginica* L.).

= *Ananthopus* Raf., Fl. Ludov.: 20–22. 1817. Type species (designated here). *Ananthopus clandestinus* Raf. (= *Commelina virginica* L.).

= *Athyrocarpus* Schltdl. ex Hassk., Flora 49: 212. 1866. Type species. *Commelina pallida* Humb. & Bonpl. ex Willd.

= *Commelinopsis* Pichon, Notul. Syst. (Paris) 12: 227. 1946. Type species. *Commelina persicariifolia* Redouté.

= *Disecocarpus* Hassk., Flora 49: 211. 1866. Type species. *Disecocarpus polygamus* (Roth) Hassk. (= *Commelina communis* L.).

= *Erxlebia* Medik., Hist. & Commentat. Acad. Elect. Sci. Theod.-Palat. 6(Phys.): 494. 1790. Type species. *Erxlebia fusiformis* Medik. (= *Commelina tuberosa* L.).

= *Eudipetala* Raf., Fl. Tellur. 3: 68. 1836 [1837]. Type species. *Eudipetala deficiens* (Hook.) Raf. (= *Commelina erecta* L.).

= *Hedwigia* Medik. Hist. & Commentat. Acad. Elect. Sci. Theod.-Palat. 6(Phys.): 495. 1790, nom. rej., non *Hedwigia* P.Beauv. Type species. *Hedwigia africana* (L.) Medik. (≡ *Commelina africana* L.).

= *Heterocarpus* Wight, Icon. Pl. Ind. Or. 6: 29. 1853. Type species (designated here). *Heterocarpus glaber* Wight (≡ *Commelina wightii* Raizada).

= Larnalles Raf., Fl. Tellur. 3: 70. 1836[1837]. Type species (designated here). *Larnalles dichotoma* Raf. (= *Commelina erecta* L.).

= *Lechea* Lour., Fl. Cochinch.: 34, 60, 1790, nom. illeg., non *Lechea* L. Type species. *Lechea chinensis* Lour. (≡ *Commelina loureiroi* Kunth).

= *Nephralles* Raf., Fl. Tellur. 3: 70. 1836[1837]. Type species. *Nephralles parviflora* Raf. (= *Commelina diffusa* Burm. f.).

= *Omphalotheca* Hassk., Bull. Congr. Int. Bot. Hort. Amsterdam 1865: 103. 1866. Type species. Not designated.

= *Ovidia* Raf., Fl. Tellur. 3: 68. 1836[1837]. Type species. *Ovidia gracilis* (Ruiz & Pav.) Raf. (= *Commelina diffusa* Burm. f.).

= *Phaeosphaerion* Hassk., Flora 49: 212. 1866. Type species. *Phaeosphaerion leiocarpum* (Benth.) Hassk. (≡ *Commelina leiocarpa* Benth.).

= *Spathodithyros* Hassk., Flora 49: 211. 1866. Type species. *Spathodithyros suffruticosus* (Blume) Hassk. (≡ *Commelina suffruticosa* Blume).

= *Tapheocarpa* Conran, Austral. Syst. Bot. 7: 585. 1994. Type species. *Tapheocarpa calandrinioides* (F.Muell.) Conran [≡ *Commelina calandrinioides* (F.Muell.) Zuntini & Frankel].

= *Trithyrocarpus* Hassk., Flora 49: 211. 1866. Type species (designated here). *Trithyrocarpus paleatus* (Hassk.) Hassk. (≡ *Commelina paleata* Hassk.).

– *Isanthina* Rchb. ex Steud., Nomencl. Bot. ed. 2, 1: 400. 1840, pro. syn.

I.A.2.ii.b. Buforrestiinae M.Pell., Plants [Basel] XXX(XXX)-XXX: 48–49. 2026. Type genus. *Buforrestia* C.B.Clarke.

I.A.2.ii.b.(1). *Buforrestia* C.B.Clarke in Candolle & Candolle, Monogr. Phan. 3: 120, 233. 1881. Type species. *Buforrestia mannii* C.B.Clarke.

I.A.2.ii.b.(2). *Tricarpelema* J.K.Morton, J. Linn. Soc., Bot. 59(380): 436. 1966. Type species. *Tricarpelema thomsonii* (C.B.Clarke) J.K.Morton [= *Tricarpelema giganteum* (Hassk.) H.Hara.].

I.A.2.ii.c. Floscopinae M.Pell., Plants [Basel] XXX(XXX)-XXX: 49–50. 2026. Type genus. *Floscopa* Lour.

I.A.2.ii.c.(1). *Floscopa* Lour., Fl. Cochinch. 1: 189, 192. 1790. Type species. *Floscopa scandens* Lour.

= *Dithyrocarpus* Kunth, Ber. Bekanntm. Verh. Königl. Preuss. Akad. Wiss. Berlin: 245. 1841. Type species. *Dithyrocarpus paniculatus* (Roxb.) Kunth. (= *Floscopa scandens* Lour.).

I.A.2.ii.c.(2). *Saxofloscopa* M.Pell., Plants [Basel] XXX(XXX)-XXX: 50–51. 2026. Type species. *Saxofloscopa africana* (Faden) M.Pell. (≡ *Tricarpelema africanum* Faden).

I.A.2.ii.c.(3). *Stanfieldiella* Brenan, Kew Bull. 14: 283. 1960. Type species. *Stanfieldiella imperforata* (C.B.Clarke) Brenan.

I.A.2.ii.d. Murdanniinae M.Pell. & Faden ex C.K.Lee, Fuse & M.N.Tamura, Bot. J. Linn. Soc. 198(2): 127. 2021. Type genus. *Murdannia* Royle, nom. cons.

I.A.2.ii.d.(1). ***Murdannia*** Royle, Ill. Bot. Himal. Mts. 1: 403, pl. 95, f. 3. 1839, nom. cons. Type species. *Murdannia scapiflora* (Roxb.) Royle [= *Murdannia edulis* (Stokes) Faden].

= *Dilasia* Raf., Fl. Tellur. 4: 122. 1836[1838], nom. rej. Type species. *Dilasia vaginata* (L.) Raf. [≡ *Murdannia vaginata* (L.) G.Brückn.].

= *Ditelesia* Raf., Fl. Tellur. 3: 69. 1836[1838], nom. rej. Type species. *Ditelesia nudiflora* (L.) Raf. [≡ *Murdannia nudiflora* (L.) Brenan].

= *Talipulia* Raf., Fl. Tellur. 2: 17. 1836[1838], nom. rej. Type species. *Talipulia malabarica* (L.) Raf. [= *Murdannia nudiflora* (L.) Brenan].

= *Streptylis* Raf., Fl. Tellur. 4: 122. 1836[1838], nom. rej. Type species. *Streptylis bracteolata* Raf. [= *Murdannia spirata* (L.) G.Brückn.].

= *Dichoespermum* Wight, Icon. Pl. Ind. Orient. 6: 31. 1853, as "*Dichaespermum*". Type species. *Dichoespermum lanceolatum* Wight [≡ *Murdannia lanceolata* (Wight) Kammathy].

= *Prionostachys* Hassk., Flora 49: 212. 1866. Type species. *Prionostachys ensifolia* Hassk. ex C.B. Clarke [= *Murdannia gigantea* (Vahl) G.Brückn.].

= *Anthericopsis* Engl., Pflanzenw. Ost-Afrikas C: 139. 1895. Type species. *Anthericopsis fischeri* Engl., nom. illeg. [≡ *Murdannia sepalosa* (C.B.Clarke) C.K.Lee et al.].

= *Gillettia* Rendle, J. Bot. 34: 55. 1896, **syn. nov.** Type species. *Gillettia sepalosa* (C.B.Clarke) Rendle [≡ *Murdannia sepalosa* (C.B.Clarke) C.K.Lee et al.].

= *Baoulia* A.Chev., Bull. Soc. Bot. France 8d: 217. 1912. Type species. *Baoulia tenuissima* A.Chev. [≡ *Murdannia tenuissima* (A.Chev.) Brenan].

= *Phaeneilema* G.Brückn., Bot. Jahrb. Syst. 61(Beibl. 137): 63. 1926, nom. illeg. Type species. *Phaeneilema sinicum* (Ker Gawl.) G.Brückn. [= *Murdannia sinica* (Ker Gawl.) G.Brückn.].

– *Aphylax* Salisb., Trans. Hort. Soc. London 1: 271. 1812, nom. nud. Type species. *Aphylax spiralis* (L.) Salisb. [≡ *Murdannia spirata* (L.) G.Brückn.].

I.A.2.ii.e. Pseudoparidinae M.Pell., Plants [Basel] XXX(XXX)-XXX: 54–55. 2026. Type genus. *Pseudoparis* H.Perrier.

I.A.2.ii.e.(1). ***Pseudoparis*** H.Perrier, Notul. Syst. (Paris) 5(3): 176. 1936. Type species. *Pseudoparis cauliflora* H.Perrier.

I.A.2.iii. Tradescantieae Meisn., Pl. Vasc. Gen.: Tab. Diagn. 406. Type genus. *Tradescantia* Ruppius ex L. emend M.Pell.

= Callisieae Pichon, Notul. Syst. (Paris) 12: 236. 1946. Type genus. *Callisia* Loefl.

= Cochliostemateae Pichon, Notul. Syst. (Paris) 12: 240. 1946. Type genus. *Cochliostema* Lem.

= Cyanoteae Pichon, Notul. Syst. (Paris) 12: 242. 1946. Type genus. *Cyanotis* D.Don, nom. cons.

= Dichorisandreae Dumort., Anal. Fam. Pl.: 55. 1829. Type genus. *Dichorisandra* J.C.Mikan, nom. cons.

= Geogenantheae Pichon, Notul. Syst. (Paris) 12: 240. 1946. Type genus. *Geogenanthus* Ule.

= Streptolirieae C.K.Lee, Fuse & M.N.Tamura, Bot. J. Linn. Soc. 198(2): 127. 2021, **syn. nov.** Type genus. *Streptolirion* Edgew.

= Zebrineae Small, Man. S.E. Fl.: 259. 1933; Zebrineae Pichon, Notul. Syst. (Paris) 12: 241. 1946, isonym. Type genus. Zebrina Schnizl. (= *Tradescantia* Ruppius ex L. emend M.Pell.).

I.A.2.iii.a. Streptoliriinae Faden & D.R.Hunt, Taxon 40: 25. 1991. Type genus. *Streptolirion* Edgew.

I.A.2.iii.a.(1). ***Spatholirion*** Ridl., J. Bot. 34: 329. 1896. Type species. *Spatholirion ornatum* Ridl.

I.A.2.iii.a.(2). ***Aëtheolirion*** Forman, Kew Bull. 16: 209. 1962. Type species. *Aëtheolirion stenolobium* Forman.

I.A.2.iii.a.(3). ***Streptolirion*** Edgew., Proc. Linn. Soc. Lond. 1: 254. 1845. Type species. *Streptolirion volubile* Edgew.

I.A.2.iii.b. Cochliostematinae M.Pell. & Faden ex C.K.Lee, Fuse & M.N.Tamura, Bot. J. Linn. Soc. 198(2): 127. 2021. Type genus. *Cochliostema* Lem.

I.A.2.iii.b.(1). ***Geogenanthus*** Ule, Repert. Spec. Nov. Regni Veg. 11: 524. 1913. Type species. *Geogenanthus wittianus* (Ule) Ule [= *Geogenanthus poeppigii* (Miq.) Faden].

= *Chamaeanthus* Ule, Verh. Bot. Vereins Prov. Brandenburg 50: 71. 1908[1909], nom. illeg. non *Chamaeanthus* Schltr. ex J.J.Sm. Type species. Chamaeanthus wittianus Ule [= *Geogenanthus poeppigii* (Miq.) Faden].

= *Uleopsis* Fedde, Just's Bot. Jahresber. 37(2): 77. 1911. Type species. *Uleopsis wittianus* (Ule) Fedde [= *Geogenanthus poeppigii* (Miq.) Faden].

I.A.2.iii.b.(2). ***Cochliostema*** Lem., Ill. Hort. 6: Misc. 70. 1859. Type species. *Cochliostema odoratissimum* Lem.

I.A.2.iii.b.(3). ***Plowmanianthus*** Faden & C.R.Hardy in Hardy & Faden, Syst. Bot. 29(2): 316–318. 2004. Type species. *Plowmanianthus perforans* Faden & C.R.Hardy.

I.A.2.iii.c. Dichorisandrinae Faden & D.R.Hunt, Taxon 40: 25. 1991. Type genus. *Dichorisandra* J.C.Mikan, nom. cons.

I.A.2.iii.c.(1). ***Dichorisandra*** J.C.Mikan, Del. Fl. Faun. Bras.: pl. 3. 1820. Type species. *Dichorisandra thyrsiflora* J.C.Mikan.

= *Stickmannia* Necker ex A.H.L.Jussieu, Dict. Sci. Nat. (ed. 2) 51: 1. 1827. Type species. *Stickmannia hexandra* (Aubl.) Kuntze [≡ *Dichorisandra hexandra* (Aubl.) C.B.Clarke].

= *Petaloxis* Raf., Fl. Tellur. 2: 83. 1836[1837]. Type species. *Petaloxis purpurea* Raf., nom. superfl. (≡ *Dichorisandra oxypetala* Hook.).

I.A.2.iii.c.(2). ***Siderasis*** Raf., Fl. Tellur. 3: 67. 1837, emend. M.Pell. & Faden, PhytoKeys 83: 6. 2017. Type species.

Siderasis acaulis Raf. [≡ *Siderasis fuscata* (Lodd.) H.E.Moore].

= *Pyrrheima* Hassk., Flora 52: 366. 1869, nom. illeg. Type species. *Pyrrheima loddigesii* Hassk., nom. illeg. [≡ *Siderasis fuscata* (Lodd.) H.E.Moore].

I.A.2.iii.d. Cyanotinae Faden & D.R.Hunt, Taxon 40: 24. 1991. Type genus. *Cyanotis* D.Don, nom. cons.

= Coleotrypinae Faden & D.R.Hunt, Taxon 40: 25. 1991. Type genus. *Coleotrype* C.B.Clarke.

I.A.2.iii.d.(1). ***Cyanotis*** D.Don, Prodr. Fl. Nepal.: 45. 1825, nom. cons. Type species. *Cyanotis barbata* D.Don.

= *Amischophacelus* R.S.Rao & Kammathy, J. Linn. Soc., Bot. 59(379): 305–306. 1966. Type species. *Amischophacelus axillaris* (L.) R.S.Rao & Kammathy [≡ *Cyanotis axillaris* (L.) D.Don ex Sweet].

= *Belosynapsis* Hassk., Flora 54: 259. 1871. Type species. *Belosynapsis kewensis* Hassk. [= *Cyanotis beddomei* (Hook. f.) Erhardt et al.].

= *Dalzellia* Hassk., Flora 48: 593. 1865. Type species. *Dalzellia vivipara* (Dalzell) Hassk. (≡ *Cyanotis vivipara* Dalzell).

= *Erythrotis* Hook. f., Bot. Mag. 101: t. 6150. 1875. Type species. *Erythrotis beddomei* Hook. f. [≡ *Cyanotis beddomei* (Hook. f.) Erhardt et al.].

= *Etheosanthes* Raf., Neogenyton 3. 1825, **syn. nov.** Type species. *Etheosanthes ciliata* (Blume) Raf. [≡ *Cyanotis ciliata* (Blume) Bakh. f.].

= *Siphostigma* Raf., Fl. Tellur. 2: 16. 1836[1837]. Type species. *Siphostigma cristata* (L.) Raf. [≡ *Cyanotis cristata* (L.) D.Don].

= *Zygomenes* Salisb., Trans. Hort. Soc. London 1: 271. 1812. Type species. *Zygomenes axillaris* (L.) Salisb. [≡ *Cyanotis axillaris* (L.) D.Don ex Sweet].

– *Cyanopogon* Welw. ex C.B.Clarke in Candolle & Candolle, Monogr. Phan. 3: 240, 258. 1881, pro. syn. Type species. Not designated.

– *Tonningia* Neck., Elem. Bot. 3: 165. 1790, nom. not validly publ., published in opera utiq. oppr.; Neck. ex A.Juss., Dict. Sci. Nat. (ed. 2) 54: 505. 1829. Type species (designated here). *Tonningia axillaris* (L.) Raf. [≡ *Cyanotis axillaris* (L.) D.Don ex Sweet].

I.A.2.iii.d.(2). ***Nivoanthus*** M.Pell., Plants [Basel] XXX(XXX)-XXX: 58–59. 2026. Type species. *Nivoanthus madagascaricus* (C.B.Clarke) M.Pell. (≡ *Coleotrype madagascarica* C.B.Clarke).

I.A.2.iii.d.(3). ***Coleotrype*** C.B.Clarke in Candolle & Candolle, Monogr. Phan. 3: 120, 238. 1881. Type species. *Coleotrype natalensis* C.B.Clarke.

I.A.2.iii.d.(4). ***Amischotolype*** Hassk., Flora 46: 391. 1863. Type species. *Amischotolype glabrata* Hassk.

= *Forrestia* A.Rich., Voy. Astrolabe 2: 1. 1834, nom. illeg., non Forrestia Raf. Type species. *Forrestia hispida* A.Rich. [≡ *Amischotolype hispida* (A.Rich.) D.Y.Hong].

= *Porandra* D.Y.Hong, Acta Phytotax. Sin. 12(4): 462, f. 1–8. 1974. Type species. *Porandra ramosa* D.Y.Hong [≡ *Amischotolype ramosa* (D.Y.Hong) C.K.Lee et al.].

I.A.2.iii.e. Tinantiinae M.Pell. ex Z.H.Feng, Plant 13(3): 145. 2025. Type genus. *Tinantia* Scheidw.

I.A.2.iii.e.(1). ***Sauvallia*** C.Wright ex Hassk., Anales Acad. Ci. Med. Habana 7: 608. 1871, as "*Sauvallea*". Type species. *Sauvallia blainii* C.Wright ex Hassk.

I.A.2.iii.e.(2). ***Tinantia*** Scheidw., Allg. Gartenzeitung 7: 365. 1839, nom. cons., non *Tinantia* Dumort., nom. rej., nec *Tinantia* M.Martens & Galeotti, nom. illeg. Type species. *Tinantia fugax* Scheidw. [= *Tinantia erecta* (Jacq.) Fenzl].

= *Commelinantia* Tharp, Bull. Torrey Bot. Club 49: 272. 1922. Type species. *Commelinantia anomala* (Torr.) Tharp. [≡ *Tinantia anomala* (Torr.) C.B.Clarke].

= *Pogomesia* Raf., Fl. Tellur. 3: 67. 1836[1837]. Type species. *Pogomesia undata* (Humb. & Bonpl. ex Willd.) Raf. [= *Tinantia erecta* (Jacq.) Fenzl].

I.A.2.iii.f. Thyrsantheminae D.R.Hunt ex Faden & D.R.Hunt, Taxon 40: 23. 1991. Type genus. *Thyrsanthemum* Pichon.

I.A.2.iii.f.(1). ***Thyrsanthemum*** Pichon, Notul. Syst. (Paris) 12: 224. 1946. Type species. *Thyrsanthemum floribundum* (M.Martens & Galeotti) Pichon.

I.A.2.iii.f.(2). ***Gibasoides*** D.R.Hunt, Kew Bull. 33(2): 331. 1978. Type species. *Gibasoides laxiflora* (C.B.Clarke) D.R.Hunt (≡ *Tradescantia laxiflora* C.B.Clarke).

I.A.2.iii.f.(3). ***Matudanthus*** D.R.Hunt, Kew Bull. 33(2): 333. 1978. Type species. *Matudanthus nanus* (M.Martens & Galeotti) D.R.Hunt.

I.A.2.iii.f.(4). ***Weldenia*** Schult. f., Flora 12: 1. 1829. Type species. *Weldenia candida* Schult. f.

I.A.2.iii.g. Tradescantiinae Rohw., Abh. Auslandsk. 61, Reihe C, Naturwiss. 18: 144. 1956. Type genus. *Tradescantia* Ruppius ex L. emend M.Pell.

= Gibasinae Rohw., Abh. Auslandsk. 61, Reihe C, Naturwiss. 18: 143. 1956. Type genus. *Gibasis* Raf.

I.A.2.iii.g.(1). ***Elasis*** D.R.Hunt, Kew Bull. 33(2): 332. 1978. Type species. *Elasis hirsuta* (Kunth) D.R.Hunt.

I.A.2.iii.g.(2). ***Ivoniella*** M.Pell., Plants [Basel] XXX(XXX)-XXX: 60–61. 2026. Type species. *Ivoniella guatemalensis* (C.B.Clarke ex Donn.Sm.) M.Pell. [≡ *Tradescantia guatemalensis* C.B.Clarke ex Donn.Sm.].

I.A.2.iii.g.(3). ***Gibasis*** Raf., Fl. Tellur. 2: 16. 1836[1837]. Type species. *Gibasis pulchella* (Kunth) Raf.

= *Heterachthia* Kunze, Bot. Zeitung (Berlin) 8: 1. 1850, nom. superfl., **syn. nov.** Type species. *Heterachthia pulchella* (Kunth) Kunze [≡ *Gibasis pulchella* (Kunth) Raf.].

I.A.2.iii.g.(4). *Tradescantia* Ruppius ex L., Species Plantarum 1: 288. 1753, emend. M.Pell., Phytokeys 98: 43. Type species. *Tradescantia virginiana* L.

≡ *Ephemerum* Mill., Gard. Dict. Abr., ed. 4.: 462. 1754, nom. superfl. Type species. *Ephemerum virginianum* (L.) Mill. (≡ *Tradescantia virginiana* L.).

= *Cymbispatha* Pichon, Not. Syst. 12: 224. 1946. Type species. *Tradescantia commelinoides* Schult. f.

= *Gonatandra* Schltdl., Linnaea 24: 659. 1851. Type species. *Gonatandra tradescantioides* Schltdl. [= *Tradescantia zanonia* (L.) Sw.].

= *Knowlesia* Hassk., Flora 49: 215. 1866. Type species. *Knowlesia spicata* (Knowles & Westc.) Hassk. (= *Tradescantia virginiana* L.).

= *Neomandonia* Hutch., Fam. Fl. Pl., Monocot. 2: 57. 1934. Type species. *Mandonia boliviana* Hassk. [≡ *Tradescantia boliviana* (Hassk.) J.R.Grant].

= *Neotreleasea* Rose, Contr. U.S. Natl. Herb. 8: 5. 1903, nom. superfl. ≡ *Treleasea* Rose, Contr. U.S. Natl. Herb. 5: 207. 1899, nom. illeg., non *Treleasia* Speg. Type species. *Tradescantia leiandra* var. *brevifolia* Torr. [≡ Tradescantia brevifolia (Torr.) Rose].

= *Sarcoperis* Raf., Fl. Tellur. 2: 16. 1837. Type species. *Sarcoperis bibracteata* (Cramer) Raf. [= Tradescantia zanonia (L.) Sw.].

= *Separotheca* Waterf., Rhodora 61: 138. 1959. Type species. *Zebrina pumila* Greene (≡ *Tradescantia pygmaea* D.R.Hunt).

= *Setcreasea* K.Schum. & Sydow, Just's Bot. Jahresber. 27(1): 452. 1901. Type. *Tradescantia brevifolia* (Torr.) Rose.

= *Skofitzia* Hassk. & Kanitz, Oesterr. Bot. Z. 22: 147. 1872 ≡ *Mandonia* Hassk., Flora 54: 260. 1871, nom. illeg, non *Mandonia* Wedd. Type species. *Tradescantia ambigua* Mart. ex Schult. & Schult. f.

= *Tropitria* Raf., Fl. Tellur. 3: 68. 1836[1837]. Type species. *Tropitria crassula* (Link & Otto) Raf. (≡ *Tradescantia crassula* Link & Otto).

= *Zanonia* Cramer., Disp. Syst.: 75. 1803, nom. illeg. Type species. *Zanonia bibracteata* Cramer., nom. illeg. [= *Tradescantia zanonia* (L.) Sw.].

= *Zebrina* Schnizl., Bot. Zeitung (Berlin) 7: 870. 1849. Type species. *Zebrina pendula* Schnizl. (= *Tradescantia zebrina* Heynh. ex Bosse).

I.A.2.iii.h. Callisiinae M.Pell., Plants [Basel] XXX(XXX)-XXX: 62–63. 2026. Type genus. *Callisia* Loefl.

I.A.2.iii.h.(1). *Cuthbertia* Small, Fl. S.E. U.S. 237, 1328. 1903 ≡ *Callisia* sect. *Cuthbertia* (Small) D.R.Hunt., Kew Bull. 41(2): 409. 1986. Type. *Callisia rosea* (Vent.) D.R.Hunt [≡ *Cuthbertia rosea* (Vent.) Small].

I.A.2.iii.h.(2). *Huntiella* M.Pell., Plants [Basel] XXX(XXX)-XXX: 63–64. 2026. Type species. *Huntiella navicularis*

(Ortgies) M.Pell. [≡ *Callisia navicularis* (Ortgies) D.R.Hunt].

I.A.2.iii.h.(3). ***Aploleia*** Raf., Fl. Tellur. 2: 17. 1836[1837]. Type. *Aploleia diffusa* Raf., nom. superfl. [≡ *Aploleia monandra* (Sw.) H.E.Moore].

= *Leiandra* Raf., Fl. Tellur. 2: 17. 1836[1837], **syn. nov.** Type species (designated here). *Leiandra cordifolia* (Sw.) Raf. [≡ *Aploleia cordifolia* (Sw.) M.Pell.].

= *Leptocallisia* (Benth. & Hook. f.) Pichon, Notul. Syst. (Paris) 12: 225, 237. 1946, nom. superfl. *Callisia* sect. *Leptocallisia* Benth. & Hook. f., Gen. Pl. 3: 854. 1883. Type species. *Callisia umbellata* Lam. [= *Aploleia monandra* (Sw.) H.E.Moore].

= *Tradescantella* Small, Fl. S.E. U.S.: 237–238, 1328. 1903, **syn. nov.** Type species. *Tradescantella floridana* (S.Watson) Small [= *Aploleia cordifolia* (Sw.) M.Pell.].

I.A.2.iii.h.(4). ***Hadrodemas*** H.E.Moore, Baileya 10: 134. 1963. Type species. *Hadrodemas warszewiczianum* (Kunth & C.D.Bouché) H.E.Moore [≡ *Callisia warszewicziana* (Kunth & C.D.Bouché) D.R.Hunt].

I.A.2.iii.h.(5). ***Callisia*** Loefl., Iter Hispan.: 305–306. 1758. Type species. *Callisia repens* (Jacq.) L. (≡ *Hapalanthus repens* Jacq.)

≡ *Hapalanthus* Jacq., Enum. Syst. Pl. 1, 12. 1760, nom. superfl. Type species. *Hapalanthus repens* Jacq. [≡ *Callisia repens* (Jacq.) L.].

= *Rectanthera* O.Deg., Fl. Hawaiiensis 1: Fam 62. 1932. Type species. *Rectanthera fragrans* (Lindl.) O.Deg. [≡ *Spironema fragrans* Lindl.].

= *Spironema* Lindl., Edwards's Bot. Reg. 26: Misc. 26. 1840, nom. illeg., non *Spironema* Raf. Type species. *Spironema fragrans* Lindl. [≡ *Callisia fragrans* (Lindl.) Woodson].

I.A.2.iii.h.(6). ***Tripogandra*** Raf., Fl. Tellur. 2: 16. 1836[1837], emend. M.Pell. & Handlos. Type species. *Tripogandra multiflora* (Sw.) Raf.

≡ *Heminema* Raf., Fl. Tellur. 2: 17. 1836[1837], nom. superfl. Type species. *Heminema multiflora* (Sw.) Raf. [≡ *Tripogandra multiflora* (Sw.) Raf.].

= *Descantaria* Schltdl., Linnaea 26: 140. 1853[1854]. Type species. *Descantaria cumanensis* (Kunth) Schltdl. [= *Tripogandra multiflora* (Sw.) Raf.].

= *Disgrega* Hassk., Flora 49: 215. 1866. Type species. *Disgrega mexicana* Hassk. ex C.B.Clarke, pro syn. [≡ *Tripogandra disgrega* (Kunth) Woodson].

= *Donnellia* C.B.Clarke, Bot. Gaz. 33(4): 261. 1902, nom. illeg., non *Donnellia* Austin. Type species. *Donnellia grandiflora* (Donn.Sm.) C.B.Clarke [≡ *Tripogandra grandiflora* (Donn.Sm.) Woodson].

= *Leptorhoeo* C.B.Clarke, Diagn. Pl. Nov. Mexic. 3: 55. 1880, **syn. nov.** Type species. *Leptorhoeo filiformis* (M.Martens

& Galeotti) C.B.Clarke [≡ *Tripogandra filiformis* (M.Martens & Galeotti) M.Pell. & Handlos].

= *Neodonnellia* Rose, Proc. Biol. Soc. Washington 19(22): 96. 1906. Type species. *Neodonnellia grandiflora* (Donn.Sm.) Rose [≡ *Tripogandra grandiflora* (Donn.Sm.) Woodson].

= *Phyodina* Raf., Fl. Tellur. 2: 16. 1836[1837], **syn. nov.** Type species. *Phyodina gracilis* (Kunth) Raf. [≡ *Tripogandra gracilis* (Kunth) M.Pell. & Handlos].

I.B. Hanguanaceae Airy Shaw, Kew Bull. 18: 260. 1964. Type genus. *Hanguana* Blume.

I.B.1. *Hanguana* Blume, Enum. Pl. Javae: 15. 1827. Type species. *Hanguana kassintu* Blume.

= *Susum* Blume ex Schult. & Schult. f., Syst. Veg. (ed. 15 bis) 7(2): XCV. 1830. Type species. *Susum anthelminthicum* Blume ex Schult. & Schult. f. [≡ *Hanguana anthelminthica* (Blume ex Schult. & Schult. f.) Masam.].

= *Veratronia* Miq., Fl. Ned. Ind. 3: 553 1859, nom. illeg. Type species. *Veratronia malayana* (Jack) Miq. [≡ *Hanguana malayana* (Jack) Merr.].

II. Pontederiineae Engl., Syllabus, ed. 2: 87. 1898. Type family. Pontederiaceae (*Pontederia* L.).

= Philydrineae Engl., Syllabus, ed. 2: 87. 1898. Type family. Philydraceae (*Philydrum* Banks & Sol. ex Gaertn.).

II.A. Philydraceae Link, Enum. Hort. Berol. Alt. 1: 5. 1821, nom. cons. Type genus. *Philydrum* Banks & Sol. ex Gaertn.

= Philydroideae Burnett, Outlines Bot.: 422. 1835, as "*Philydreae*" or rather "*Philydridae*". Type genus. *Philydrum* Banks & Sol. ex Gaertn.

= Philydreae M.Gómez, Noc. Bot. Sist.: 1893. Type genus. *Philydrum* Banks & Sol. ex Gaertn.

II.A.1. *Helmholtzia* F.Muell., Fragm. 5: 202. 1866. Type species. *Helmholtzia acorifolia* F.Muell.

II.A.2. *Orthothylax* (Hook. f.) Skottsb., Bot. Jahrb. Syst. 65: 264. 1932 ≡ *Philydrum* sect. *Orthothylax* Hook. f., Bot. Mag. 99: t. 6056. 1873. Type species. *Philydrum glaberrimum* Hook. f. [≡ *Orthothylax glaberrimus* (Hook. f.) Skottsb.].

II.A.3. *Philydrella* Caruel, Nuovo Giorn. Bot. Ital. 10: 91. 1878. Type species. *Philydrella pygmaea* (R.Br.) Caruel.

≡ *Hetaeria* Endl. Gen. Pl.: 133. 1836, nom. illeg. non *Hetaeria* Blume, Bijdr. Fl. Ned. Ind. 8: 409. 1825. Type species. *Hetaeria pygmaea* (R.Br.) Endl. [≡ *Philydrella pygmaea* (R.Br.) Caruel].

≡ *Pritzelia* F.Muell. Descr. Notes Papuan Pl. 1: 13. 1875, nom. illeg. non *Pritzelia* Walp., Repert. 2: 428. 1843, nec *Pritzelia* Schauer Flora 26: 407. 1843. Type species. *Pritzelia pygmaea* (R.Br.) F.Muell. ex Benth. [≡ *Philydrella pygmaea* (R.Br.) Caruel].

II.A.4. *Philydrum* Banks & Sol. ex Gaertn., Fruct. Sem. Pl. 1: 62. 1788. Type species. *Philydrum lanuginosum* Banks & Sol. ex Gaertn.

= *Garciana* Lour., Fl. Cochinch.: 14. 1790. Type species. *Garciana cochinchinensis* Lour. [≡ *Philydrum cochinchinense* (Lour.) M.Pell.].

II.B. Pontederiorae M.Pell., Plants [Basel] XXX(XXX)-XXX: 35. 2026. Type family. Pontederiaceae Kunth (*Pontederia* L.).

II.B.1. Haemodoraceae R.Br., Prodr.: 299. 1810, nom. cons. Type genus. *Haemodorum* Sm.

= Conostylidaceae Takht., Sist. Magnoliof. [Syst. Magnolioph.]: 313. 1987. Type genus. *Conostylis* R.Br.

= Dilatridaceae M.Roem., Handb. Allg. Bot. 3: 476. 1840, as "*Dilatrideae*". Type genus. *Dilatris* P.J.Bergius.

= Wachendorfiaceae Herb., Amaryllidaceae: 48. 1837. Type genus. *Wachendorfia* Burm. ex L.

= Xiphidiaceae Dumort., Anal. Fam. Pl.: 59, 61. 1829. Type genus. *Xiphidium* Aubl.

II.B.1.i. Haemodoroideae Arn., Botany: 133. 1832, as "*Haemodoreae*". Type genus. *Haemodorum* Sm.

= Wachendorfioideae Arn., Botany: 133. 1832, as "*Wachendorfieae*". Type genus. *Wachendorfia* Burm. ex L.

II.B.1.i.a. Haemodoreae Dumort., Anal. Fam. Pl.: 62. 1829. Type genus. *Haemodorum* Sm.

= Dilatrideae M.Roem., Handb. Allg. Bot. 3: 476. 1840. Type genus. *Dilatris* P.J.Bergius.

II.B.1.i.a.(1). ***Dilatris*** P.J.Bergius, Descr. Pl. Cap.: 9. 1767. Type species. *Dilatris corymbosa* P.J.Bergius.

II.B.1.i.a.(2). ***Paradilatris*** (Hopper ex J.C.Manning) Hopper in Hopper et al., Nuytsia 36: 169. 2025 ≡ *Dilatris* subg. *Paradilatris* Hopper ex J.C.Manning, S. African J. Bot. 113: 104. 2017. Type species. *Paradilatris viscosa* (L. f.) Hopper.

II.B.1.i.a.(3). ***Haemodorum*** Sm., Trans. Linn. Soc. London 4: 213. 1798. Type. *Haemodorum corymbosum* Vahl.

II.B.1.i.a.(4). ***Lachnanthes*** Elliott, Sketch Bot. S. Carolina 1: 47. 1816. Type species. *Lachnanthes tinctoria* (Walter ex J.F.Gmel.) Elliott [= *Lachnanthes caroliniana* (Lam.) Dandy].

= *Heritiera* J.F.Gmel., Syst. Nat. (ed. 13) 2(1): 113. 1791, nom. illeg., non *Heritiera* Aiton, nec *Heritiera* Retz. Type species. *Heritiera tinctorum* Walter ex J.F.Gmel. [= *Lachnanthes caroliniana* (Lam.) Dandy].

= *Camderia* Dumort., Anal. Fam. Pl.: 80. 1829, nom. superfl. Type species. *Heritiera tinctorum* Walter ex J.F.Gmel. [= *Lachnanthes caroliniana* (Lam.) Dandy].

– *Anonymos* Walter, Fl. Carol.: 37. 1788, nom. not validly publ.

– *Gyrotheca* Salisb., Trans. Hort. Soc. London 1: 327. 1812, nom. nud. Type species. *Gyrotheca tinctorum* (Walter ex J.F.Gmel.) Salisb. [= *Lachnanthes caroliniana* (Lam.) Dandy].

II.B.1.i.b. Wachendorfieae Dumort., Anal. Fam. Pl.: 61. 1829. Type genus. *Wachendorfia* Burm. ex L.

II.B.1.i.b.(1). ***Schiekia*** Meisn., Pl. Vasc. Gen. 2(12): 300. 1842. Type species. *Schiekia orinocensis* (Kunth) Meisn.

≡ *Troschelia* Klotzsch & M.R.Schomb. in Schomburgk MR, Reis. Br.-Guiana: 1066. 1849, nom. nud. Type species. *Troschelia orinocensis* (Kunth) Klotzsch & M.R.Schomb. [≡ *Schiekia orinocensis* (Kunth) Meisn.].

II.B.1.i.b.(2). ***Wachendorfia*** Burm. ex L., Syst. Nat., ed. 10. 2: 864. 1759. Type species. *Wachendorfia paniculata* Burm.

≡ *Pedilonia* C.Presl, Pedilonia Nov. Pl. Gen.: 1. 1829, nom. superfl. Type species. *Pedilonia violacea* C.Presl (= *Wachendorfia paniculata* Burm.).

= *Barberetta* Harv., Gen. S. Afr. Fl. Pl. (ed. 2): 377. 1868. Type species. *Barberetta aurea* Harv. [≡ *Wachendorfia aurea* (Harv.) M.Pell.].

– *Wachendorfia* Burm., Wachendorfia: 2. 1757, nom. nud. Type species. *Wachendorfia paniculata* Burm.

II.B.1.i.c. Xiphidieae Dumort., Anal. Fam. Pl.: 61. 1829. Type genus. *Xiphidium* Aubl.

II.B.1.i.c.(1). ***Cubanicula*** Hopper, J.E.Gut., E.J.Hickman, M.Pell. & R.J.Sm., PhytoKeys 169: 5. 2020. Type species. *Cubanicula xanthorrhizos* (C.Wright ex Griseb.) Hopper et al. (≡ *Xiphidium xanthorrhizon* C.Wright ex Griseb.).

II.B.1.i.c.(2). ***Pyrrorhiza*** Maguire & Wurdack, Mem. New York Bot. Gard. 9(3): 318. 1957. Type species. *Pyrrorhiza neblinae* Maguire & Wurdack.

II.B.1.i.c.(1). ***Xiphidium*** Loefl. ex Aubl., Hist. Pl. Guiane 1: 33, pl. 11. 1775. Type species. *Xiphidium caeruleum* Aubl.

= *Durandia* Boeckeler, Allg. Bot. Z. Syst. 2: 160, 173. 1896. Type species. *Durandia macrophylla* Boeckeler (= *Xiphidium caeruleum* Aubl.).

– *Tonduzia* Boeckeler ex Tonduz, Bull. Herb. Boissier 3: 464. 1895, nom. nud. Type species. *Tonduzia macrophylla* Boeckeler ex Tonduz (= *Xiphidium caeruleum* Aubl.).

– *Xiphidium* Loefl., Iter Hispan.: 179. 1758, nom. nud. Type species. *Xiphidium caeruleum* Aubl.

II.B.1.ii. **Conostylidoideae** Lindl., Veg. Kingd.: 153. 1846, as "*Conostyleae*"; Conostylidoideae T.D.Macfarl. & Hopper, Fl. Australia 45: 454. 1987, isonym. Type genus. *Conostylis* R.Br.

II.B.1.ii.a. **Tribonantheae** T.D.Macfarl. & Hopper, Fl. Australia 45: 454. 1987. Type genus. *Tribonanthes* Endl.

II.B.1.ii.a.(1). ***Tribonanthes*** Endl., Nov. Stirp. Dec.: 27. 1839. Type species. *Tribonanthes australis* Endl.

II.B.1.ii.b. **Conostylideae** Benth., Fl. Austral. 6: 417, 425. 1873, as "*Conostyleae*". Type genus. *Conostylis* R.Br.

= Anigozantheae Z.H.Feng, Plant 13(3): 149, **syn. nov.** Type genus. *Anigozanthos* Labill.

= Phlebocaryeae Meisn., Pl. Vasc. Gen.: Tab. Diagn. 396, Comm. 299. 1842, **syn. nov.** Type genus. *Phlebocarya* R.Br.

II.B.1.ii.b.(1). ***Anigozanthos*** Labill., Voy. Rech. Pérouse 1: 410. 1800. Type species. *Anigozanthos rufus* Labill.

≡ *Anigosia* Salisb., Trans. Hort. Soc. London 1: 327. 1812, nom. superfl. Type species. *Anigosia flavida* (DC.) Salisb. (≡ *Anigozanthos flavidus* DC.).

≡ *Schwaegrichenia* Spreng. Pl. Pugil. 2: 58. 1815, nom. superfl. Type species (designated here). *Schwaegrichenia flavida* (DC.) Spreng. (≡ *Anigozanthos flavidus* DC.).

= *Macropidia* J.Drumm. ex Harv., Hooker's J. Bot. Kew Gard. Misc. 7: 57. 1855. Type species. *Macropidia fumosa* J.Drumm. ex Harv., nom. superfl. (≡ *Anigozanthos fuliginosus* Hook.).

II.B.1.ii.b.(2). ***Conostylis*** R.Br., Prodr.: 300. 1810. Type species. *Conostylis aculeata* R.Br.

= *Androstemma* Lindl., Edwards's Bot. Reg. 23 App. (Swan River): xlvi. 1840. Type species. *Androstemma junceum* Lindl. (≡ *Conostylis androstemma* F.Muell.).

= *Blancoa* Lindl., Edwards's Bot. Reg.: 45. 1840 ≡ *Styloconus* Baill., Hist. Pl. 13: 75. 1894, nom. superfl. Type species. *Blancoa canescens* Lindl. [≡ *Conostylis canescens* (Lindl.) F.Muell.].

II.B.1.ii.b.(3). ***Phlebocarya*** R.Br., Prodr.: 301. 1810. Type species. *Phlebocarya ciliata* R.Br.

II.B.2. Pontederiaceae Kunth, Nov. Gen. Sp. 1, ed. qu.: 265. 4–11 Mai 1816, nom. cons. Type genus. *Pontederia* L.

≡ Pontederieae Dumort., Anal. Fam. Pl.: 61. 1829. Type genus. *Pontederia* L.

≡ Pontederoideae Eaton, Bot. Dict., ed. 4: 28. 1836. Type genus. *Pontederia* L.

= Eichhornieae O.Schwartz, Bot. Jahrb. Syst. 61(Beibl. 139): 32, 50. 1927. Type genus *Eichhornia* Kunth, nom. cons. (= *Pontederia* L.).

= Heterantheraceae J.Agardh, Theoria Syst. Pl.: 36. 1858. Type genus. *Heteranthera* Ruiz & Pav.

= Heterantthereae O.Schwartz, Bot. Jahrb. Syst. 61(Beibl. 139): 35, 50. 1927. Type genus. *Heteranthera* Ruiz & Pav.

II.B.2.i. *Heteranthera* Ruiz & Pav., Fl. Peruv. Prodr.: 9. 1794. Type species. *Heteranthera reniformis* Ruiz & Pav.

≡ *Heterandra* P.Beauv., Trans. Amer. Philos. Soc. 4: 175. 1799, nom. superfl. Type species. *Heterandra reniformis* (Ruiz & Pav.) P.Beauv. (= *Heteranthera reniformis* Ruiz & Pav.).

= *Buchosia* Vell., Fl. Flumin.: 33. 1829, "*Buch'osia*". Type species. *Buchosia aquatica* Vell. (= *Heteranthera reniformis* Ruiz & Pav.).

= *Eurystemon* Alexander, N. Amer. Fl. 19: 55. 1937. Type species. *Eurystemon mexicanum* (S.Watson) Alexander (≡ *Heteranthera mexicana* S.Watson).

= *Hydrothrix* Hook. f., Ann. Bot. (Oxford) 1: 89. 1887 ≡ *Hookerina* Kuntze, Revis. Gen. Pl. 2: 718. 1891, nom. superfl. Type species. *Hydrothrix gardneri* Hook. f. [≡ *Heteranthera gardneri* (Hook. f.) M.Pell.].

= *Leptanthus* Michx., Fl. Bor.-Amer. 1: 24. 1803. Type species. *Leptanthus ovalis* Michx., nom. superfl. [≡ *Heteranthera limosa* (Sw.) Willd.].

= *Lunania* Raf., Med. Fl. 2: 106. 1830, nom. illeg. non *Lunania* Hook., London J. Bot. 3: 317. 1844. Type species. *Lunania uniflora* Raf., nom. superfl. [≡ *Heteranthera limosa* (Sw.) Willd.].

= *Phrynium* Loefl. ex Kuntze, Revis. Gen. Pl. 3(3): 318. 1898, nom. illeg., non *Phrynium* Willd., Sp. Pl. Editio quarta 1: 1, 17. 1797. Type species. *Phrynium limosum* (Sw.) Kuntze [≡ *Heteranthera limosa* (Sw.) Willd.].

= *Schollera* Schreb., Gen. Pl. 785. 1791, nom. illeg., non *Schollera* Roth, Tent. Fl. Germ. 1: 165, 170. 1788. Type species. *Schollera graminea* (Michx.) Willd. ex A.Gray [= *Heteranthera dubia* (Jacq.) MacMill.].

= *Scholleropsis* H.Perrier, Notul. Syst. (Paris) 5: 158. 1936. Type species. *Scholleropsis lutea* H.Perrier [≡ *Heteranthera lutea* (H.Perrier) M.Pell.].

= *Triexastima* Raf., Fl. Tellur. 4: 121. 1836 [1838]. Type species. *Triexastima uniflora* Raf., nom. superfluous [≡ *Heteranthera limosa* (Sw.) Willd.].

II.B.2.ii. *Pontederia* L., Sp. Pl. 1: 288. 1753. Type species. *Pontederia cordata* L.

≡ *Michelia* Adans., Fam. Pl. 2: 201. 1763, nom. superfl. Type species. *Pontederia cordata* L.

≡ *Narukila* Adans., Fam. Pl. 2: 54. 1763, nom. superfl. Type species. *Narukila cordata* (L.) Nieuwl. (≡ *Pontederia cordata* L.).

≡ *Unisema* Raf. Med. Repos. 5: 352. 1808, nom. superfl. Type species. *Unisema obtusifolia* Raf., nom. illeg. (≡ *Pontederia cordata* L.).

= *Cabanisia* Klotzsch ex Schltdl., Abh. Naturf. Ges. Halle 6: 176. 1862. Type species. *Cabanisia caracasana* Klotzsch ex Schltdl., nom. illeg. (≡ *Pontederia paniculata* Spreng.).

= *Calcarunia* Raf., Med. Fl. 2: 106. 1830. Type species. *Calcarunia hastata* (L.) Raf., nom. not validly publ. (≡ *Pontederia hastata* L.).

= *Carigola* Raf., Fl. Tellur. 2: 10. 1837. Type species. *Carigola hastata* (L.) Raf. (≡ *Pontederia hastata* L.).

= Eichhornia Kunth, Enum. Pl. 4: 129. 1843. Type species. *Eichhornia azurea* (Sw.) Kunth. (≡ *Pontderia azurea* Sw.).

= *Gomphima* Raf., Fl. Tellur. 2: 10. 1837. Type species. *Gomphima vaginalis* (Burm. f.) Raf. (≡ *Pontederia vaginalis* Burm. f.).

= *Kadakia* Raf., Fl. Tellur. 2: 9. 1837. Type species. *Kadakia dilatata* (Buch.-Ham.) Raf. (= *Pontederia hastata* L.).

= *Leptosomus* Schltdl., Abh. Naturf. Ges. Halle 6: 174. 1862. Type species. *Leptosomus natans* (P.Beauv.) Schltdl. (≡ *Pontederia natans* P.Beauv.).

= *Limnostachys* F.Muell., Fragm. 1: 24. 1858. Type species. *Limnostachys cyanea* F.Muell. [≡ *Pontederia cyanea* (F.Muell.) M.Pell. & C.N.Horn].

= *Monochoria* C.Presl, Reliq. Haenk. 1(2): 127. 1827. Type species. *Monochoria hastifolia* C.Presl., nom. illeg. (≡ *Pontederia hastata* L.).

= *Piaropus* Raf., Fl. Tellur. 2: 81. 1837, nom. rej. Type species. *Piaropus mesomelas* Raf., nom. illeg. (≡ *Pontederia crassipes* Mart.).

= *Reussia* Endl., Gen. Pl.: 139. 1836. Type species. *Reussia triflora* Endl. ex Seub. [≡ *Pontederia triflora* (Endl. ex Seub.) G.Agostini et al.].

– *Pontederas* Hoffmanns., Verz. Pfl.: 137. 1824, orth. var.

– *Pontederaea* Kuntze, Revis. Gen. Pl. 2: 718. 1891, orth. var.

– *Umsema* Raf. Med. Repos. 5: 352 1808, orth. var.

– *Unisemma* D.A.Godron, in Orbigny CVD, Dict. Univ. Hist. Nat.: 761. 1848, orth. var.
